# Research on multi-defects classification detection method for solar cells based on deep learning

**DOI:** 10.1371/journal.pone.0304819

**Published:** 2024-06-21

**Authors:** Zhenwei Li, Shihai Zhang, Chongnian Qu, Zimiao Zhang, Feng Sun

**Affiliations:** School of Mechanical Engineering, Tianjin University of Technology and Education, Tianjin, China; Mohanlal Sukhadia University, INDIA

## Abstract

Solar cells are playing a significant role in aerospace equipment. In view of the surface defect characteristics in the manufacturing process of solar cells, the common surface defects are divided into three categories, which include difficult-detecting defects (mismatch), general defects (bubble, glass-crack and cell-crack) and easy-detecting defects (glass-upside-down). Corresponding to different types of defects, the deep learning model with different optimization methods and a classification detection method based on multi-models fusion are proposed in the paper. In the proposed model, in order to solve the mismatch problem between the default anchor boxes size of YOLOv5s model and the extreme scale of the battery mismatch defect label boxes, the K-means algorithm was adopted to re-cluster the dedicated anchor boxes for the mismatch defect label boxes. In order to improve the comprehensive detection accuracy of YOLOv5s model for the general defects, the YOLOv5s model was also improved by the methods of image preprocessing, anchor box improving and detection head replacing. In order to ensure the recognition accuracy and improve the detection speed for easy-detecting defects, the lightweight classification network MobileNetV2 was also used to classify the cells with glass-upside-down defects. The experimental results show that the proposed optimization model and classification detection method can significantly improve the defect detection precision. Respectively, the detection precision for mismatch, bubble, glass-crack and cell-crack defects are up to 95.64%, 91.8%, 93.1% and 98.0%. By using lightweight model to train the glass-upside-down defect dataset, the average classification accuracy reaches 100% and the detection speed reaches 13.29 frames per second. The comparison experiments show that the proposed model has a great improvement in detection accuracy compared with the original model, and the defect detection speed of lightweight classification network is improved more obviously, which confirms the effectiveness of the proposed optimization model and the multi-defect classification detection method for solar cells defect detection.

## 1 Introduction

Solar cells are the core components of photovoltaic power generation system in aerospace equipment. The key factors which affect the photoelectric conversion efficiency and service life of photovoltaic power generation system include cell material and structure, manufacturing process, surface defects, etc [1]. In 2015, the photoelectric conversion efficiency of gallium arsenide cells independently developed by China Guodian Photovoltaic company reached 34.5% [2]. Photovoltaic has the advantages of high conversion efficiency, strong radiation resistance and good high temperature resistance, which make it suitable for application in aerospace field. However, defects such as cell-crack, mismatch, bubble, glass-crack and glass-upside-down often occur in the production process of gallium arsenide cells, which seriously affect the photoelectric conversion efficiency and the service life of solar cells. Therefore, production enterprises mostly use manual detection method to reduce the safety risks caused by defective products. But manual detection method has some problems, such as strong subjectivity, inconsistent standards, high cost, low efficiency, and fatigue etc.

In recent years, the solar cells defect detection method based on deep learning has the characteristics of high precision, fast speed, and strong robustness, and has achieved certain application effects. For example, Tang et al. [3] used Generative Adversarial Network (GAN) to generate many high-resolution datasets of cell defects, Convolutional Neural Network (CNN) was used to achieve the accurate classification of solar cell defects based on the datasets. Akarm et al. [4] realized the accurate identification of photovoltaic module defects by training the lightweight network from initial state, and the average accuracy reached 98.67%. On the other hand, the average accuracy reached 99.23% by using transfer learning method to train the model. Fan et al. [5], aiming at the micro-crack defect target of polycrystalline silicon solar cells, proposed a detection method that enhanced the micro-crack defect feature through image preprocessing and used ResNet50 network as the backbone network to extract the defect feature. Through testing the industrial micro-crack defect dataset, it was found that the detection accuracy rate of this method could reach 98.29%. It also proved that transfer learning method was very helpful to improve the accuracy of model detection. Zubair et al. [6] proposed a deep learning method to quickly identify and locate defects in electroluminescence images of silicon solar cells, which solved the problem that the efficiency of manual detection cannot meet the needs of the manufacturing industry. Tian et al. [7] proposed a fusion detection model based on VGG(Visual Geometry Group) classification network and U-net ++ segmentation network. First, an improved VGG16 network was used to extract the electroluminescence image features of solar cells to detect the defects. Then U-net ++ network is used to segment the defects of solar cells. The test results shown that the defect detection accuracy of improved VGG16 model reaches 95.2%, and the average MIoU (Mean Intersection over Union) value of U-Net++ defect segmentation model reaches 95.5%. In summary, relevant scholars have introduced deep learning methods into solar cells defect detection, and achieved good results that are difficult to by the conventional image analysis and processing methods.

The gallium arsenide solar cells studied in this paper are applied in aerospace field, its structure, shape, material, production process and defect characteristics are different from those of crystalline silicon cells, and it has a very low tolerance for cell defects. Considering the diversity and complexity of solar cells surface defects, the classification detection method is proposed for different defects, and the comprehensive studies of YOLOv5s model optimization and multi-model fusion application are carried out in the paper.

## 2 Solar cells defect detection system, datasets construction and defects feature analysis

**Fig 1:**
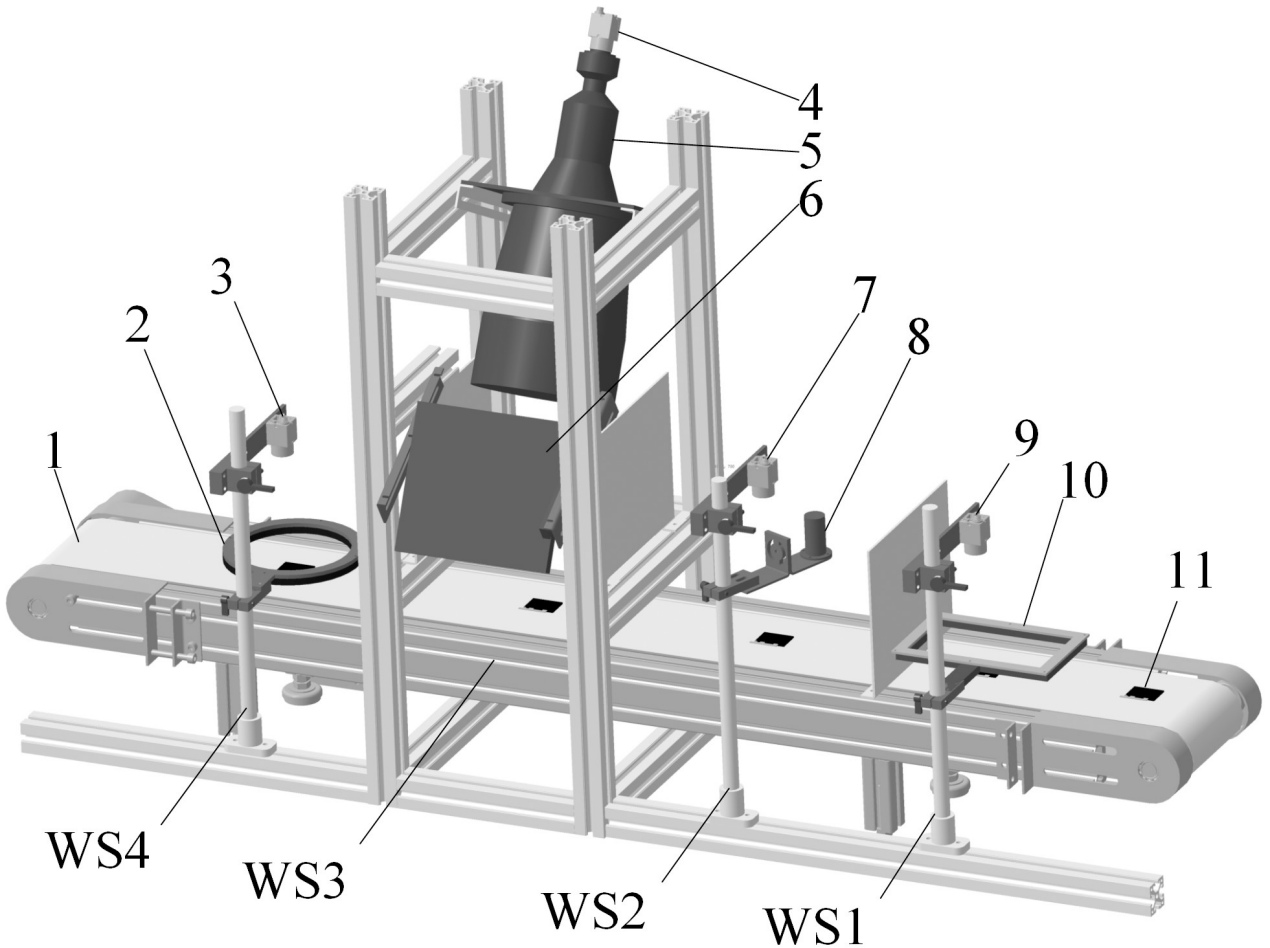
The defect detection system for solar cells. Notes: 1-conveyer belt, 2-the light source of WS4, 3-the camera of WS4, 4-the camera of WS3, 5-the telecentric, 6-the light source of WS3, 7-the camera of WS2, 8-the light source of WS2, 9-the camera of WS1, 10-the light source of WS1, 11-solar cell.

**Fig 2:**
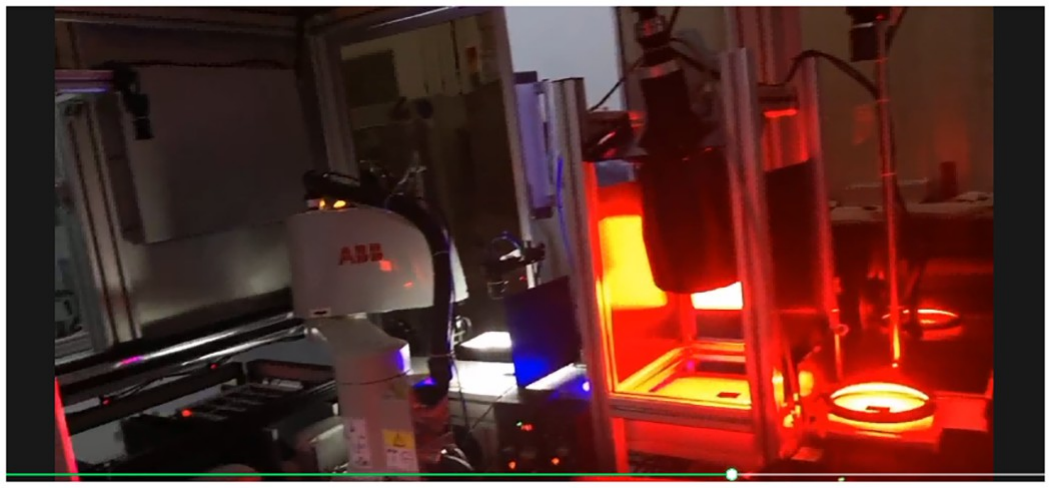
The field diagram of the image detection system.

Based on the field application requirements, The defect detection system for solar cells is built and shown in Fig 1. The solar cells will pass through four detection working stations (from WS1 to WS4) in sequence, in each station, a grayscale industrial camera with a resolution of 5496×3672 is adopted to collect the images of the solar cells. In order to better reflect different defects characteristics, the bar type white light source is used in WS1 and mainly used to detect the mismatch defects, the single emission blue light source is used in WS2 and mainly used to detect the cell-crack defects, the telecentric and panel type red light source are used in WS3 and mainly used to detect the bubble and glass-upside-down defects, the low angle circular white light source is used in WS4 and mainly used to detect the glass-crack defects. In fact, the same defects may be reflected at multi working stations, therefore it is also an important measure to improve the defects rate. The field diagram of the image detection system is shown in Fig 2.

**Fig 3:**
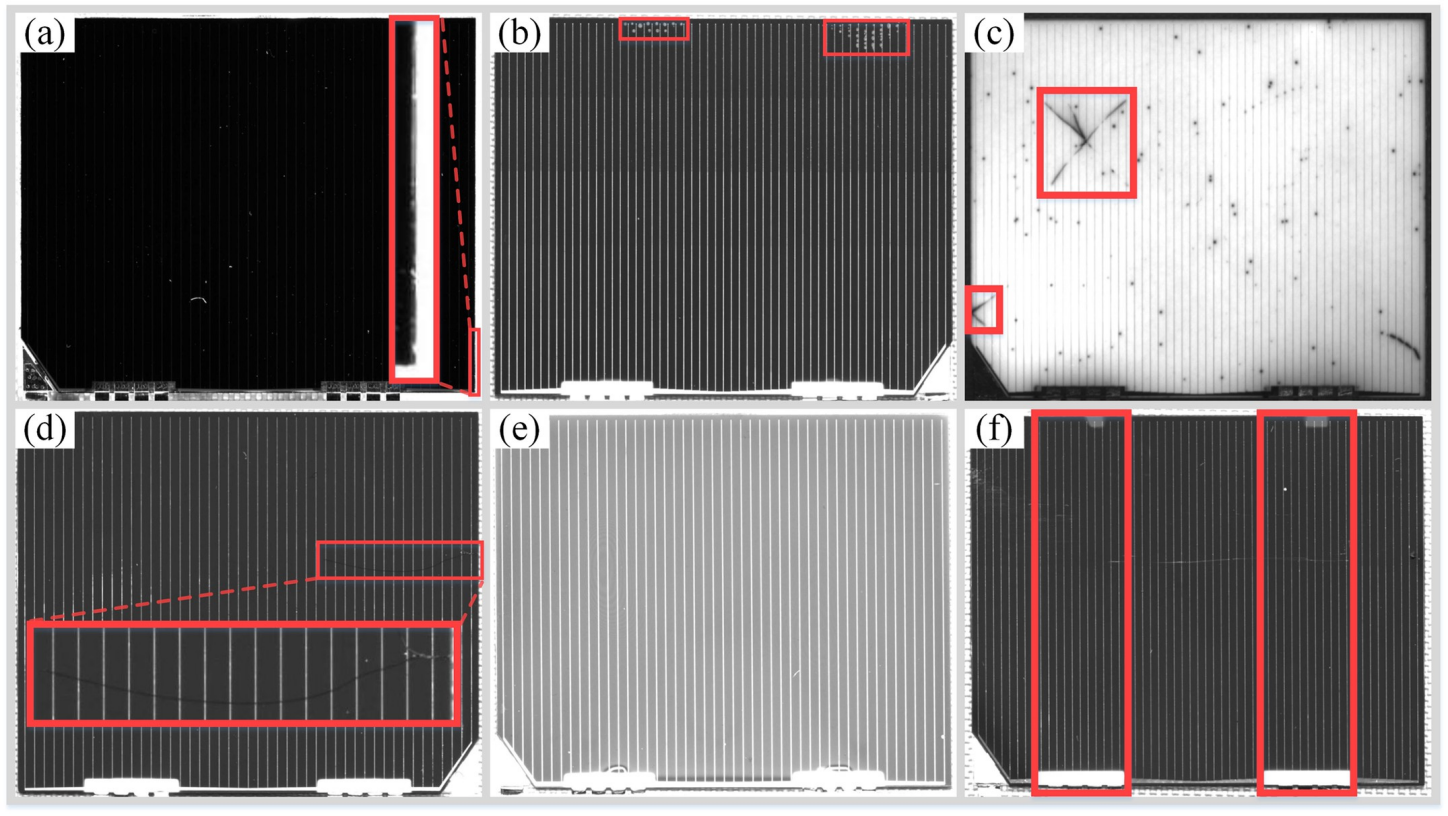
The 5 types defects of solar cells. Notes: (a) mismatch defect, (b) bubble defects, (c) cell-crack defects, (d) glass-crack defect, (e) glass-upside-down defect 1, (f) glass-upside-down defect 2.

Based on test rig as shown in Fig 1, the data set covering 5 types defects of solar cells is constructed, and the typical defect characteristics are shown in Fig 3.

The mismatch defects with the characteristics of narrow-long and blurred edges are shown as Fig 3(a). The large mismatch may be formed when the glass cover is attached on the cell (the paster process). Based on enterprise production standards, if the misaligned width exceeds 0.1mm will be taken as the mismatch defects.

The bubble defects with the characteristics of water droplets shape are shown in Fig 3(b). In the paster process, the bubble defects may be formed due to the low vacuum degree between glass cover and cell or the gas precipitation during the solidification process of the cover adhesive.

The cell-crack defects with the characteristics of one or more extension line from edge are shown in Fig 3(c). Compared to crystalline silicon cells, the thickness of gallium arsenide solar cells is thinner, during the cutting process, the gallium arsenide solar cells are more prone to appear crack due to the uneven pressure of mechanical equipment.

The glass-crack defects with the characteristics of divergent extension and hidden cracks are shown in Fig 3(d). The glass-crack defects usually caused by surface collision during the workpiece transfer process or improper blade force during the scraping glue process.

The glass-upside-down defects have two types of characteristics. One type of feature is shown in Fig 3(e), compared with the normal image of solar cells, the brightness of the defect images is higher due to the reflective differences on both sides of the glass cover under the same image acquisition environment. Another type of feature is shown in Fig 3(f), there is a high brightness clip print directly above the two interconnecting pieces.

In this paper, the five types of defects are divided into 3 categories based on the difficulty of defect detection. That is difficult-detecting defects (mismatch), general defects (bubble, glass-crack and cell-crack) and easy-detecting defects (glass-upside-down). Comprehensively considering of the detection accuracy and efficiency, different detection models and optimization methods are applied according to the 3 categories defects of solar cells.

Usually, in the supervised learning process, in order to prevent overfitting in the training process due to insufficient sample data, image enhancement is carried out on the existing data set to increase the number of defective sample images. During the image enhancement period, in order to preserve the hidden crack feature and the integrity of the small target as much as possible, the methods of geometric transformation and brightness adjustment are used for mismatch and glass-crack defects. Meanwhile, the methods of Gaussian blur, contrast enhancement and geometric transformation were used for cell-crack, bubble and glass-upside-down defects. The changes in the number of defect datasets before and after image enhancement are shown in Table 1.

**Table 1:** The defect datasets before and after image enhancement.

datasets	mismatch	cell-crack	bubble	Glass-crack	glass-upside-down	total
Number of original images	236	282	152	460	312	1442
Number of images after enhancement	708	846	1280	920	984	4738

In the paper, Intel^®^ Core^™^ i9-12900K processor and NVIDIA GeForce RTX3080Ti graphics processor are adopted in the experimental platform. The memory size is 32GB and the graphics memory size is 12GB. Pytorch-gpu deep learning framework is adopted, and CUDA10.1 and corresponding versions CUDnn are used for GPU-accelerated training.

## 3 YOLOv5s network structure

**Fig 4:**
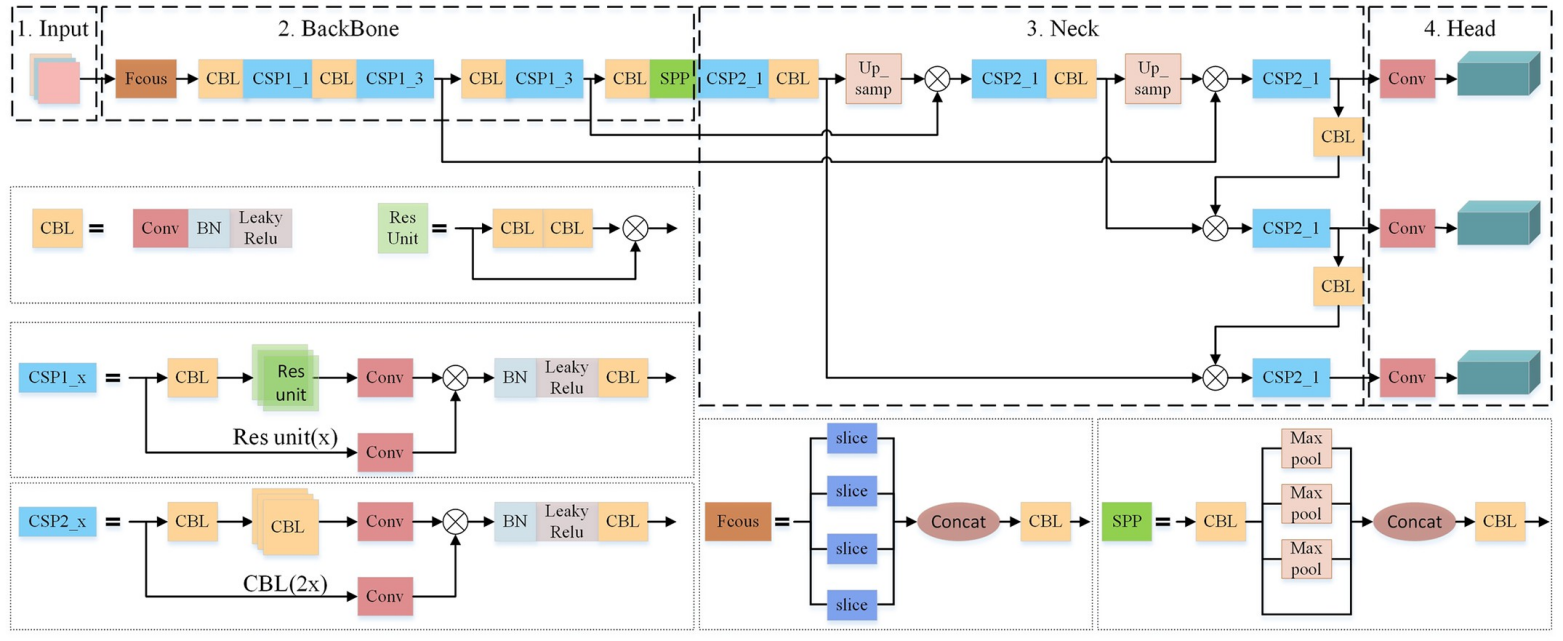
The structure of YOLOv5s model.

Several target detection models were compared and analyzed in the early stage of the paper for the solar cells defect detection. For example, Lu Sha and Huang Jing respectively verified the advantages of YOLOv5 algorithm in the field of surface defect detection of solar cells through experimental comparison [8, 9]. Therefore, YOLOv5s with the best comprehensive detection performance is taken as a base and core for analysis and optimization in the paper. The structure of YOLOv5s model is shown in Fig 4. The model is mainly composed of BackBone network for feature extraction, Neck network for feature fusion and Head network for target information prediction.

The backbone feature extraction network used in YOLOv5s is CSPDarknet (Cross Stage Partial Darknet). The core of CSPDarknet is the Residual network and the CSPnet network. The residual edge of the residual network solves the problem of network degradation by passing the input directly to the output by adopting a jump connection. CSPnet split the stack of the original residual blocks into two parts, the trunk part continues to perform the stacking of residual blocks. Another part is directly connected to the end after a small amount of processing, which forms a large residual edge to enhance the feature fusion capability and information transfer efficiency [10]. The backbone feature extraction network of YOLOv5s also applies the Spatial Pyramid Pooling (SPP) structure. Through pooling operations of different sizes on input feature images, information of different scales is merged to realize multi-scale feature extraction and enhance the model’s perception ability of different scales targets [11].

The Neck Network of YOLOv5s adopts FPN (Feature Pyramid Network) and PAN (Path Aggregation Network) structure for multi-scales feature fusion, which enables YOLOv5s to effectively integrate shallow edge information and deep semantic information, and improve the accuracy and robustness of defect detection [12].

In addition, YOLOv5s introduces the Focus network structure, which obtains four independent feature layers by taking a value every other pixel in the input image. Then these four feature layers are stacked so that the width and height information is concentrated in the channel information, which increases the sensitivity of the network to the width and height information. YOLOv5s uses SiLU (Sigmoid-weighted Linear Unit) as the activation function. SiLU has the characteristics of lower down bound, no upper bound, smooth, and non-monotonic. Compared with the ReLU activation function, SiLU performs better in the deep model [13].

## 4 The detect method for difficult-detecting defects of solar cells based on K-means clustering anchor boxes

**Fig 5:**
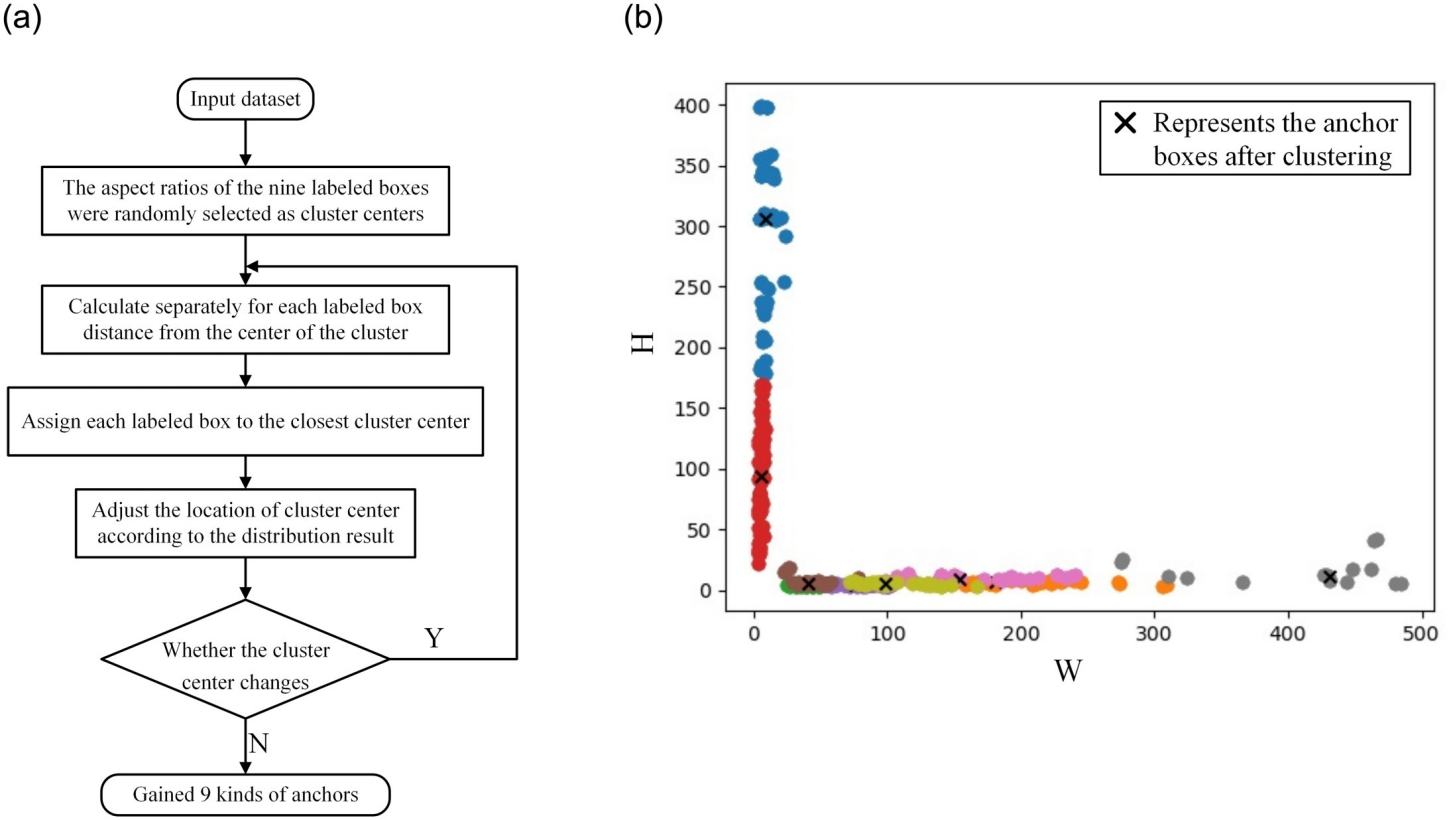
The steps of K-means algorithm and the visualization of the clustering result of mismatch defects. (a) The process of obtaining the optimal size of the anchor boxes by the K-means algorithm. (b) The visualization results of the clustering process for mismatch defects labeled boxes.

It can be seen from Fig 3(a), the mismatch defects are difficult to be detected due to its narrow and blurry edges characteristics. The default anchor boxes of YOLOv5s model are clustered based on the COCO datasets with 80 types, and there are many significant differences in scale between the default anchor boxes and the mismatch defect labeled boxes. Therefore, the paper uses the K-means algorithm to determine the optimal size of the anchor boxes to improve the detection accuracy of mismatch defects. The process of obtaining the optimal size of the anchor boxes by the K-means algorithm is shown in Fig 5(a).

Firstly, 9 cluster centers are randomly select from all the labeled boxes sizes, and the distances from other labeled boxes to the cluster centers are calculated separately. The calculation method of the clustering distances is shown in Eq 1.


D(b,c)=1−I(b,c) (1)

In Eq (1), *D* represents the distances from data sample points to the cluster centers, *c* represents the coordinates and width-height information of the cluster centers, *b* represents the coordinates and width-height information of the labeled boxes, *I* represents the intersection over union ratio between two boxes.

Finally, the labeled boxes are divided into the nearest cluster centers, and the coordinate means of each type data are calculated according to the current classification results, and then the new cluster centers can be obtained. If the new cluster center changes, the distances from the data sample to the cluster center are recalculated and the data samples are reclassified until the cluster center no longer changes. The mismatch defect dataset of solar cells is used to re-cluster the anchor boxes in the paper, and the visualization results of the clustering process are shown in Fig 5(b) (the input image resolution is set to [640,640]). In Fig 5(b), solid points represent the actual size of the labeled boxes for mismatch defects (the final clustering results are expressed by 9 colors respectively), × represents the size of the anchor boxes after clustered.

**Fig 6:**
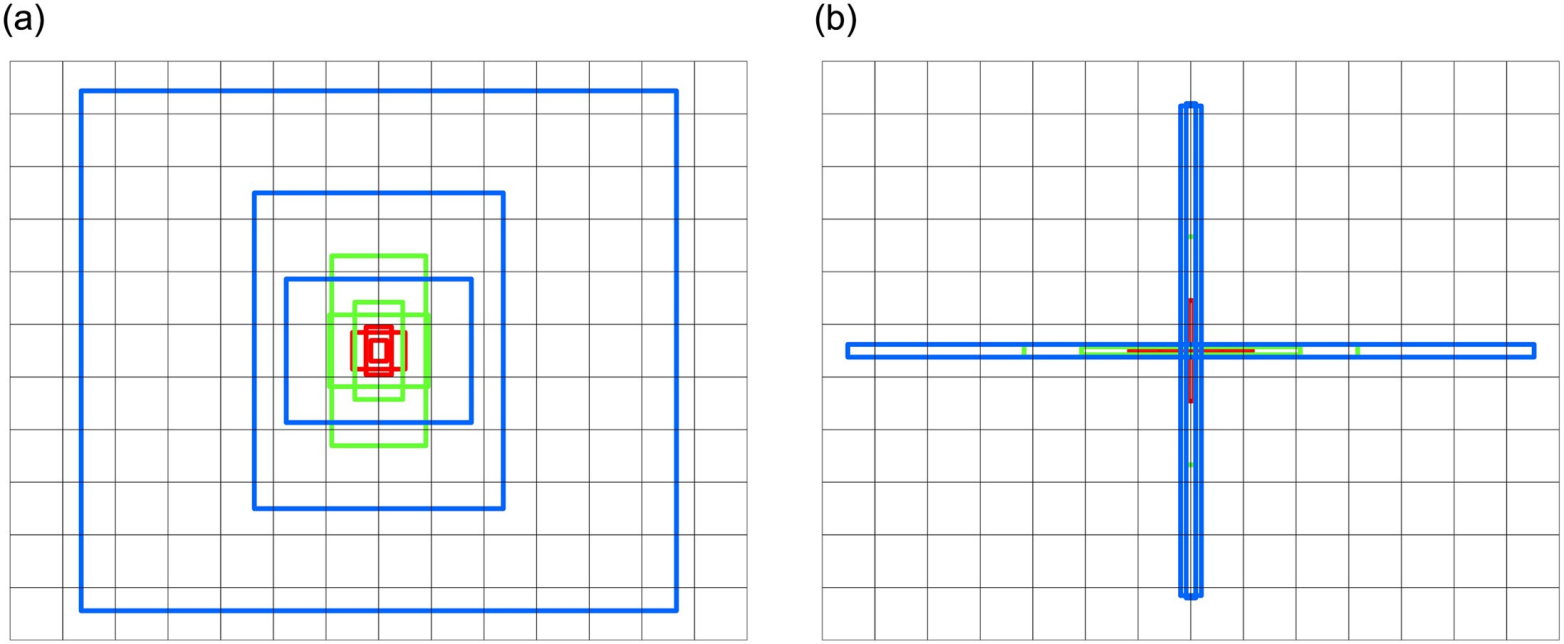
The comparison of 9 anchor boxes before and after clustered calculation. (a) The 9 anchor boxes before K-means clustered calculation. (b) The 9 anchor boxes after K-means clustered calculation.

The sizes of 9 anchor boxes before clustering calculation are [(10, 13), (16, 30), (33, 23)], [(30, 61), (62, 45), (59, 119)], [(116, 90), (156, 198), (373, 326)], and the sizes of 9 anchor boxes after clustering calculation are [(39, 4), (4, 63), (77, 3)], [(137, 5), (6, 143), (209, 8)], [(6, 310), (430, 8), (13, 307)]. The visual comparison of scale changes before and after anchor boxes clustered is shown in Fig 6.

It can be seen from Fig 6, the anchor boxes sizes after using K-means clustered are more adaptable to the characteristics of mismatch defects in solar cells, which is beneficial for solving the low recall rate problem in mismatch defect detection.

**Fig 7:**
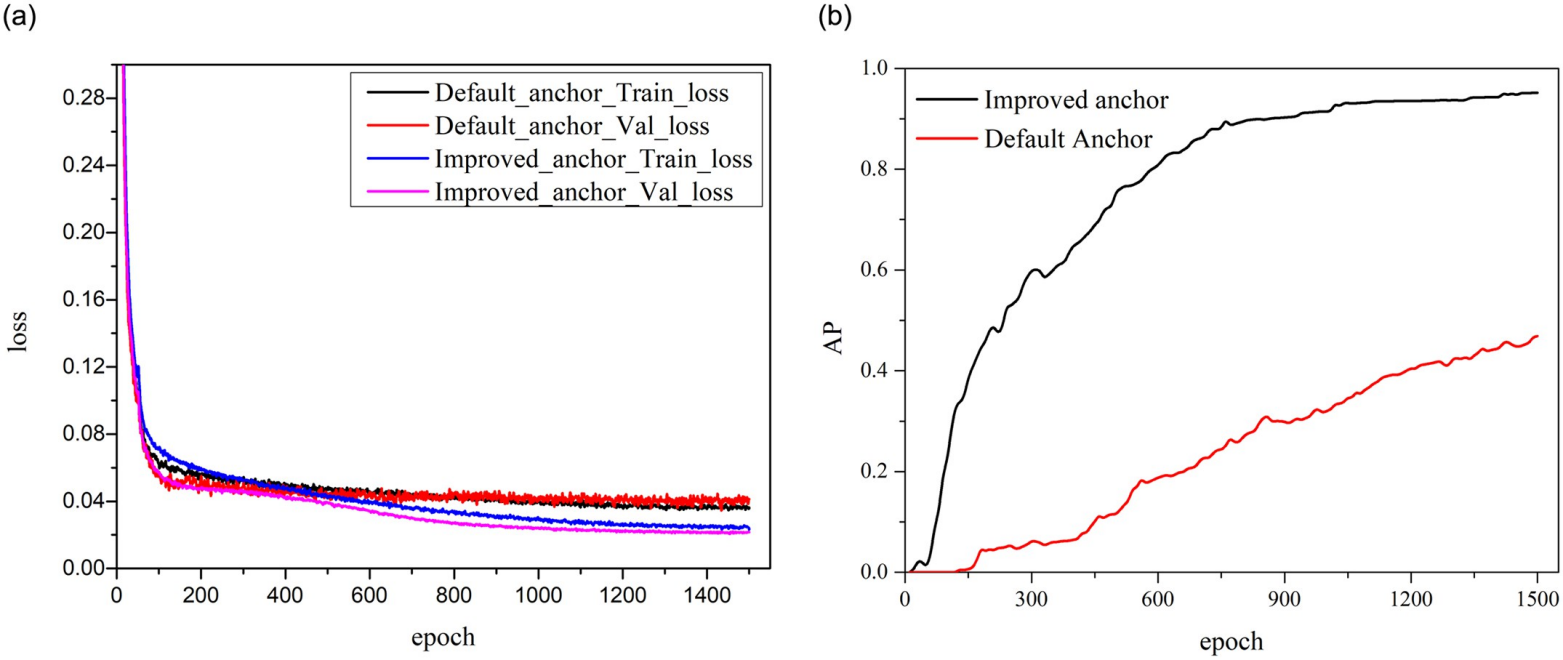
The loss and AP curves of default and improved anchor boxes. (a) The loss curves. (b) The AP curves.

For mismatch defect detection, several deep learning models were attempted in the early experimental stage, but the detection effects were unsatisfactory. To verify the detection effect for mismatch defects by using K-means clustering to improve anchor boxes, the YOLOv5s model is trained using the mismatch defects dataset. In the training phase, the input image resolution is [1280,1280], and the iterative epoch is set as 1500. The loss and average precision (AP) curves of default and improved anchor boxes are respectively shown in Fig 7.

It can be seen from Fig 7(a), the overfitting occurs at 800 epochs during the training process of YOLOv5s model using default anchor boxes, which cause the model to be unable to accurately learn the features of mismatch defects. Comparatively, the YOLOv5s model, using K-means clustering to improve anchor boxes, has a good convergence of the loss function during the training process. According to the variation curve of AP in Fig 7(b), the detection accuracy of the YOLOv5s model with improved anchor boxes increases faster as the iterations time increases. The detection accuracy indicators of the YOLOv5s model with default and improved anchor boxes are shown in Table 2, where the P (Precision) refers to the proportion of correct prediction among all prediction targets, the R (Recall rate) refers to the proportion of correct prediction among all real targets, the AP (Average Precision) refers to the area enclosed by P-R curve, the F1 is the harmonic average of P and R indicators.

**Table 2:** The detection accuracy indicators of the YOLOv5s model.

Method	AP(%)	R (%)	P (%)	F1
Using default anchor boxes	62.87	9.09	100.00	0.17
Using improved anchor boxes	95.64	74.75	97.37	0.85

**Fig 8:**
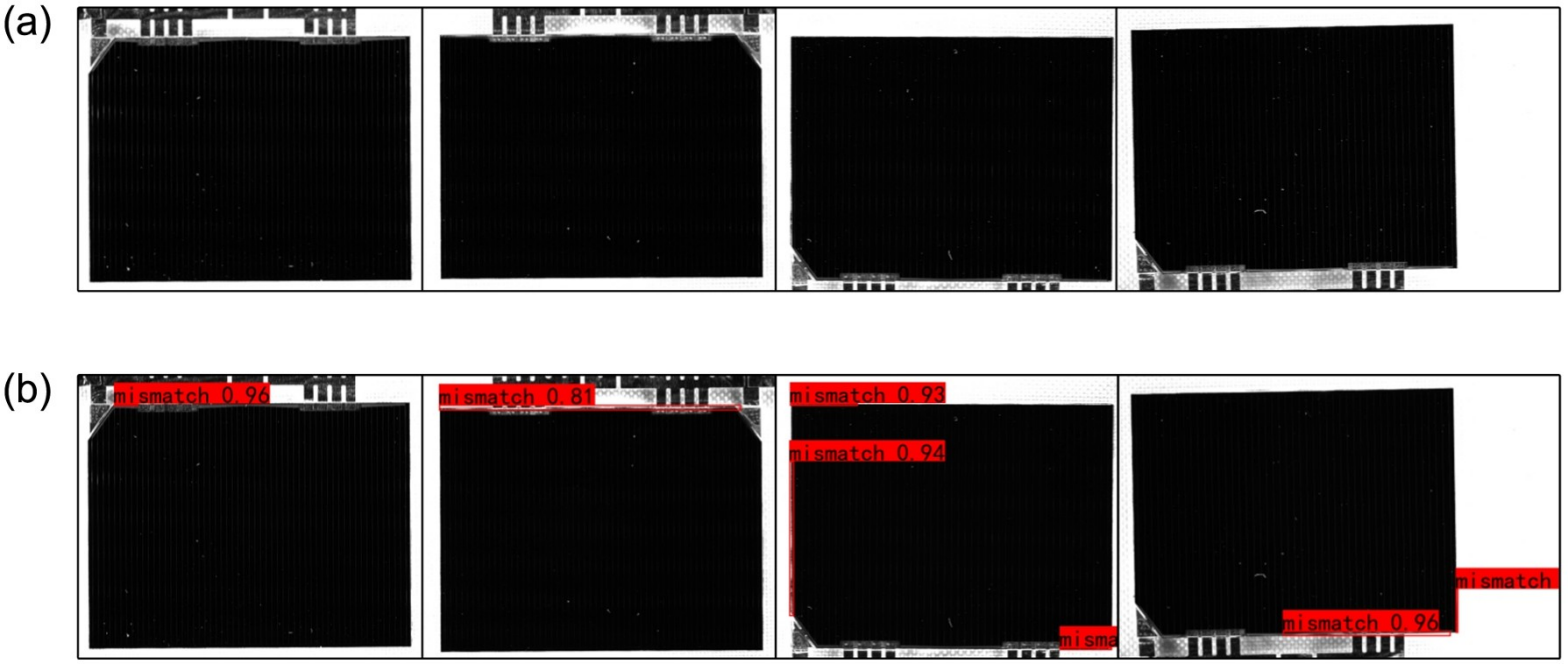
Test results of mismatch defects before and after the anchor boxes are improved. (a) The detection results of original YOLOv5 model. (b) The detection results of YOLOv5 model with improved anchor boxes.

The detection accuracy indicators in Table 2 further indicate: The recall rate of YOLOv5s model with anchor box improved by K-means is significantly improved. The AP indicator is increased by 32.77 percentage points and reach 95.64%. Before and after the anchor box is improved, the actual test results of YOLOv5s model are shown in Fig 8.

Fig 8 shows that the YOLOv5s model with default anchor boxes has a very low detection rate for mismatch defects, and the detection rate is only 11.1% in actual detection statistics. However, the detection rate is up to 93.3% in actual detection statistics by the YOLOv5s model with improved anchor boxes. It is evident that the problem of large miss detection rate of mismatch defects is largely solved by improving the default anchor boxes of the YOLOv5s model.

## 5 General defect detection based on improved decoupled head

The YOLOv5s algorithm employs a Coupled Head, wherein the fusion features directly pass through a convolutional layer to form the detection head, and the detection tasks of classification, localization and confidence are coupled in the detection head. Related studies show that the classification and regression tasks of target detection algorithms are mutual repulsive relation. In other words, the focus of classification and regression are different, which the classification pays greater attention to the texture content of the target, while regression focuses more on the edge information of the target [14]. The YOLOv5s detection head combines the classification and regression tasks for calculation, and the parameters are shared by classification and regression branches. Due to the defect classification task is relative ease, the precision of defect locations will be low when the task fusion algorithm is used for solar cells defect detection, and the slow convergence may occur during model training. Aim at above problem, the Decoupled Head method of YOLOX model is adopted to replace the Coupled Head structure in YOLOv5s so as to resolve the mutual interference problem between classification and regression tasks in solar cells defect detection. Meanwhile the convergence speed can be accelerated, and the precision of defect classification and localization can be improved.

**Fig 9:**
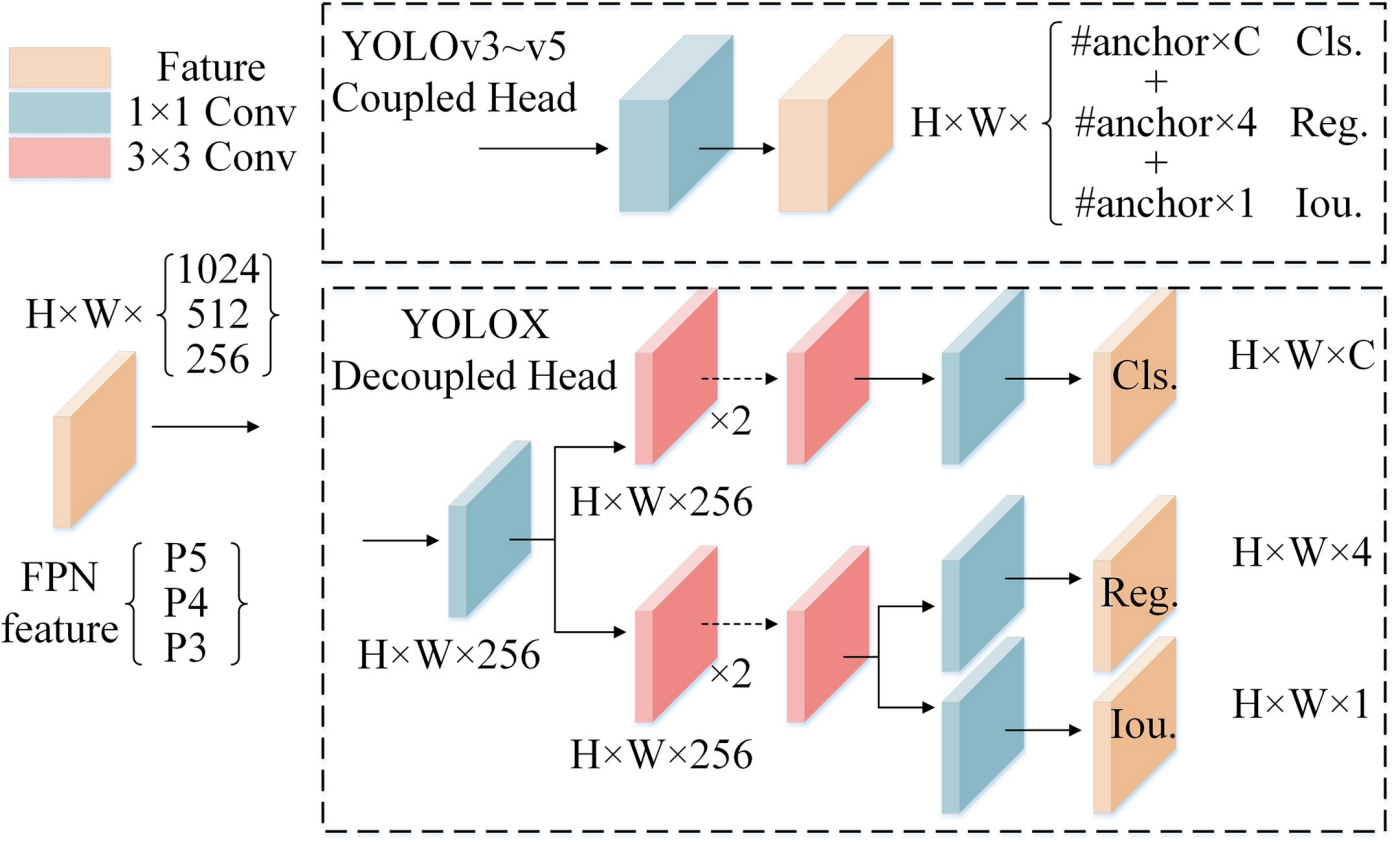
The structures of coupled head and decoupled head.

The structures of Coupled Head and Decoupled Head are illustrated in Fig 9. Comparing these two structures, the Coupled Head employs a 1×1 convolutional layer to obtain a feature tensor, which contains Cls (class prediction information), Reg (the position information of detection box) and Obj (the object whether or not is included in the bounding box). In contrast, the Decoupled Head extracts three feature tensors (labeled as P5, P4, and P3) from the Neck. The Decoupled Head is splits into two branches after passing through a 1×1 convolutional layer. The upper branch obtains a feature tensor with Cls information by two layers 3×3 convolutional operation and one layer 1×1 convolutional operation. The lower branch is further divided into two branches after two 3×3 layers convolutional operation. The upper branch obtains Reg information after one layer 1×1 convolutional operation, and the lower branch obtains the Obj information after one layer 1×1 convolutional operation. In the outputting feature tensors of Fig 9, C is the number of label categories, 4 represents the center coordinate(x, y), width(w) and height(h) information of predicted bounding box, and 1 represents the confidence level of the included objects.

Based on the Decoupled Head algorithm, the location and category information of solar cell defects is learned by different network branches respectively, and the generalization ability and robustness of the model can be improved.

Among the general defects of solar cells, the characteristics of hidden cracks of solar cell defects increases the identifying difficulty of model. To improve the average precision of general defect detection, the methods of Decoupled Head replacing, Image preprocessing, and anchor boxes improving are comprehensively used to optimize the YOLOv5s model.

**Fig 10:**
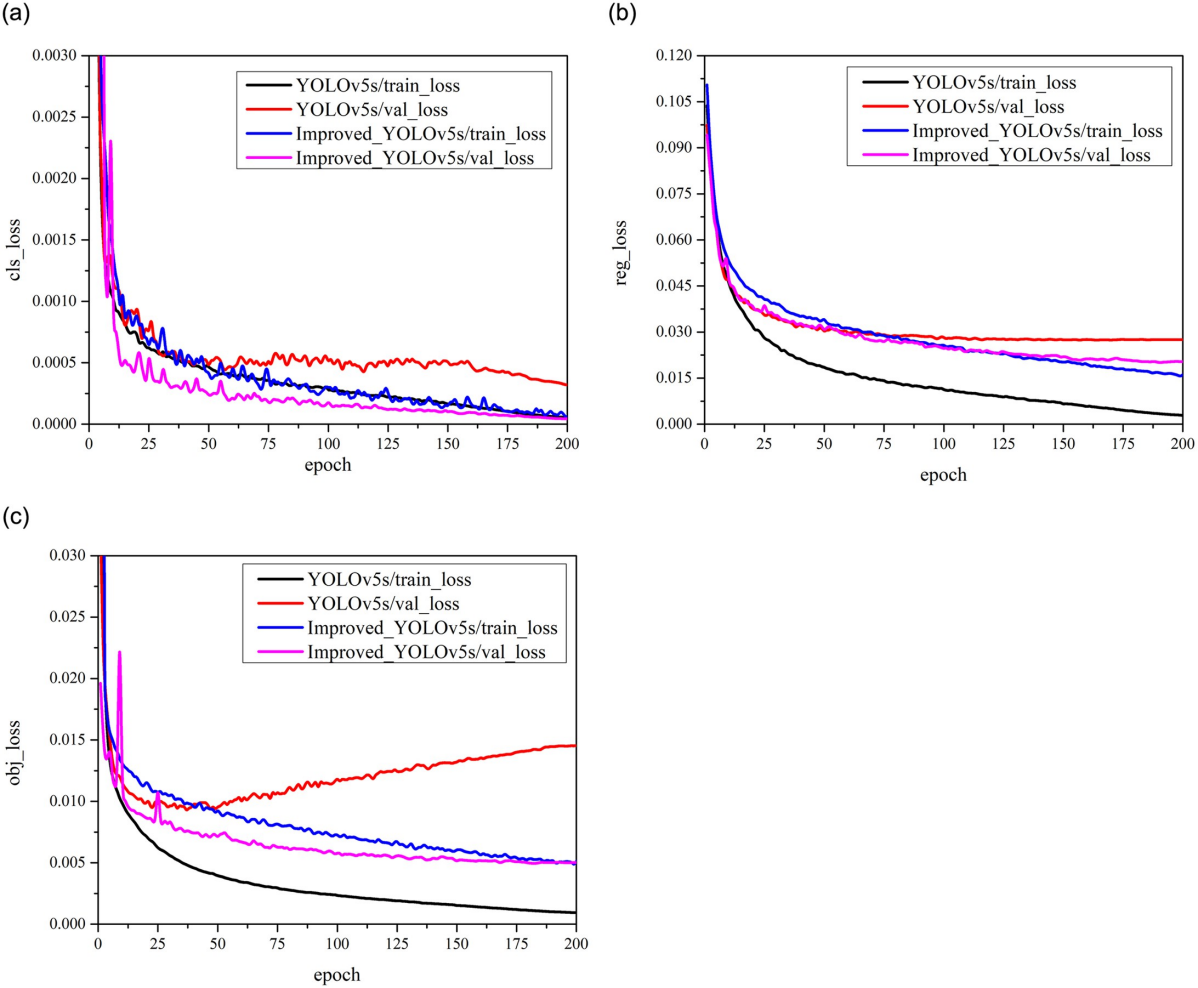
The variation process of various loss functions before and after model improvement. (a) Loss curves for predicting category information, Cls_loss. (b) Loss curves for prediction boxes information, Reg_loss. (c) Loss curves for predicting the inclusion or exclusion of objects, Obj_loss.

The resolution of the input images with general defects is set to [1280, 1280] and used to train model, and the number of epochs is set to 200. The Reg, Cls, and Obj loss curves of original YOLOv5s model and improved YOLOv5s model are shown in Fig 10. It can be seen from Fig 10 that the overfitting problem during the training process can be alleviated in the improved YOLOv5s model.

**Fig 11:**
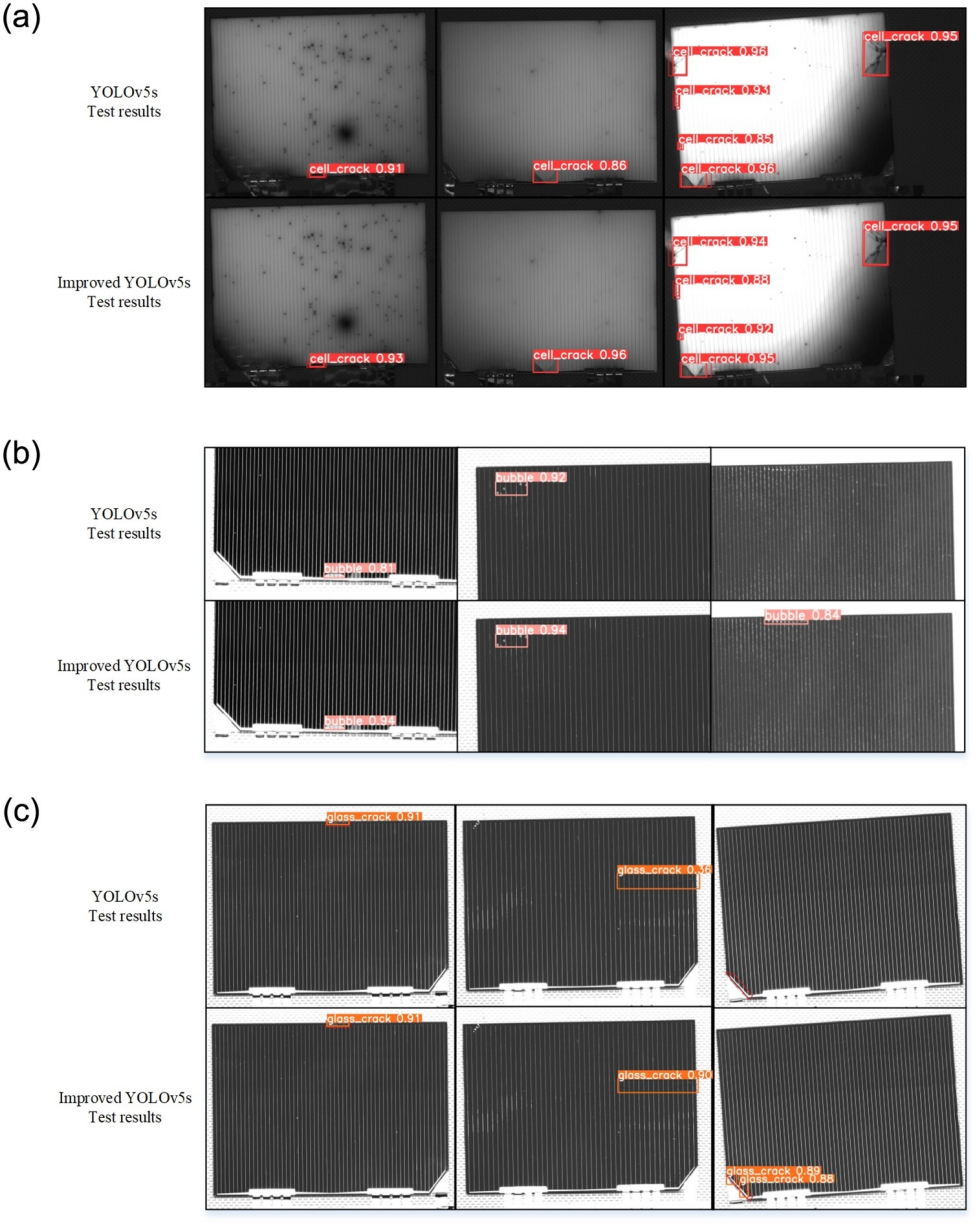
The testing effectiveness comparison for general defects before and after the model is improved. (a) The detection effectiveness comparison for cell-crack defects. (b) The detection effectiveness comparison for bubble defects. (c) The detection effectiveness comparison for glass-crack defects.

The test results for general defects by the original YOLOv5s model and improved YOLOv5s model are shown in Fig 11. Fig 11(a) and 11(b) show that the improved model demonstrates better overall detection performance for cell-crack and bubble defects, especially for detecting small defect features. Fig 11(c) shows that the original YOLOv5s model and improved YOLOv5s model have the equally excellent detection effect for the obvious glass-crack defects of solar cells. However, in the case of hidden crack defects, the improved YOLOv5s model exhibits a significant advantage.

The detection accuracy indicators of the original YOLOv5s model and improved YOLOv5s model for general defects are shown in Table 3. In Table 3, "Class" represents the category of defects, "Labels" indicates the number of predicted bounding boxes for defects, " mAP@0.5" denotes the AP mean value of all predicted categories when the IOU threshold is set to 0.5, and "mAP@0.5:.95" denotes the mean of 10 mAP values when the IOU threshold is respectively set to ‘0.5, 0.55,… 0.95’.

**Table 3:** Comparative experiments of general defect detection.

Method	Class	Labels	P	R	AP	mAP@.5	mAP@.5:.95
YOLOv5s	cell-crack	126	0.958	0.909	0.957	0.83	0.58
	bubble	174	0.869	0.76	0.824		
	glass-crack	62	0.886	0.629	0.708		
Improved-YOLOv5s	cell-crack	126	0.968	0.944	0.98	0.943	0.727
	bubble	174	0.901	0.885	0.918		
	glass-crack	62	0.965	0.901	0.931		

Comparing to the original YOLOv5s model, the detection accuracy of improved YOLOv5s model is higher for solar cells defect detection, and the detection accuracy indicators of P, R and AP corresponding to different defects both improved in varying degree. The indicators of " mAP@0.5" and "mAP@0.5:.95" are respectively increased 11.3 and 14.7 percentage points, which show the comprehensive detection effect of improved YOLOv5s model is better.

## 6 Easy-detecting defects identification based on the lightweight MobileNetV2 network

For glass-upside-down defects of solar cells, the detection results only need to identify whether or not the glass cover is pasted upside to down, and the defect localization is unnecessary. So the glass-upside-down defects identification belong to a typical binary classification problem. In practical applications, in order to reduce the inference time, the lightweight target classification networks are usually used to solve the binary classification problem. For comparative analysis, three types of models are used to test the identification effect for glass-upside-down defects in the paper.

The lightweight target classification network MobileNetV2 was introduced by the Google team in 2018. This model has the characteristics of small structural scale and high accuracy, which making it be a typical representative of target classification networks.

The deep residual network ResNet50 was developed by Microsoft Research Asia, which the residual learning was employed to increase network depth, and the problems of gradient vanishing and exploding in deep neural networks was effectively solved. ResNet50 model has achieved outstanding performance in various computer vision tasks.

For comparative analysis, the glass-upside-down defect dataset is used to respectively train MobileNetV2, ResNet50 and YOLOv5s models. The input image resolution for the MobileNetV2 and ResNet50 models was set to [224,224], while the input image resolution for the YOLOv5s model was set to [1280,1280]. The detection results of three models are presented in Table 4, where FPS represents the number of images detected per second during the inference process.

**Table 4:** Detection results of three models for glass-upside-down defects.

Model	AP	R	P	FPS(Pictures·s^-1^)
MobileNetV2	100%	100%	100%	13.29
ResNet50	99.46%	99.43%	99.49%	13.80
YOLOv5s	99.64%	97.9%	97.22%	1.36

From Table 4, it is evident that all three models exhibit high detection accuracy for glass-upside-down defects, which confirms the detectability of this defects. However, the target classification networks MobileNetV2 and ResNet50 have faster detection speeds and 10 times greater than YOLOv5s model. Furthermore, in comparison to ResNet50 and YOLOv5s models, MobileNetV2 model achieves higher classification accuracy. The comparative experiments demonstrate that the MobileNetV2 target classification network improve detection efficiency while ensuring accuracy for glass-upside-down defects of solar cells.

## 7 Design of classification detection scheme for solar cell defects

**Fig 12:**
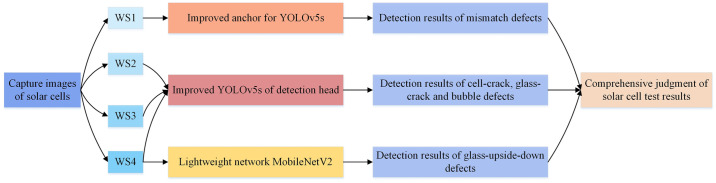
The classification detection scheme for solar cell defects.

In response to the diversity and complexity of solar cell defects, and based on above experiments analysis, a classification detection scheme for solar cell defects is designed and shown in Fig 12.

In the scheme depicted in Fig 12, the YOLOv5s model with improved anchor boxes is employed to real-time detect the mismatch defects of solar cells. The YOLOv5s model with Decoupled Head is used to real-time detect the cell-crack, glass-crack and bubble defects of solar cells. Furthermore, the lightweight target classification network MobileNetV2 is utilized to real-time identify glass-upside-down defects of solar cells. The detection results of the three models are synthesized to determine whether there are defects in the solar cells.

In order to verify the effectiveness of the proposed classification detection scheme, the model is compared with the original YOLOv5 in practical tests, and the statistical results are shown in Table 5.

**Table 5:** Detection result statistics of the classification detection scheme.

Model	Test Images	Successful Detection	Missed Detection	Accuracy
Original YOLOv5	593	457	136	77.07%
Classification detection model	508	459	49	90.35%

As can be seen from the actual test results in Table 5, the combined detection performance of the classification detection scheme improves significantly, and its actual test accuracy reaches 90.35%, which is an improvement of 13 percentage points compared to the original model.

## 8 Conclusion

In response to the diversity and complexity of solar cell defects, the deep learning classification detection scheme is proposed to improve the accuracy and overall detection speed for various defect categories. The main research conclusions are as follows:

1. Aim at the characteristics of the mismatch defects of solar cells, the K-means clustering algorithm is employed to re-cluster exclusive anchor boxes for mismatch defects, so as to improve the detection accuracy of the YOLOv5s model for mismatch defects and accelerates the convergence during model training. Experimental results demonstrate that the AP of YOLOv5s model with improved anchor boxes reach 95.64%, and the percentage has increased by 32.77 compared to the original model. More obviously, the recall of mismatch defects is greatly improved and the low detection rate problem is effectively resolved.
2. Aim at the characteristics of general defects of solar cells, the measures of coupled head replacement, image preprocessing techniques and re-clustering anchor boxes are comprehensively used to optimize the YOLOv5 model. Experimental results show that the AP value of improved model achieves reach 94.3%, and the percentage has increased by 11.3 compared to the original model.
3. Aim at the characteristics of the glass-upside-down defects of solar cells, the lightweight target classification network MobileNetV2 is employed to ensure detection accuracy while improving the detection speed. Experimental results demonstrate that the AP value of MobileNetV2 model reach 100%, and the detection speed reach 13.29 FPS.

## Supporting information

S1 Dataset (ZIP)
