# Supplementary figures and images for: Research on multi-defects classification detection method for solar cells based on deep learning (part 2 of 2)
Source: PLoS One. 2024 Jun 21;19(6):e0304819. doi: 10.1371/journal.pone.0304819 (PMC11192367; doi:10.1371/journal.pone.0304819)

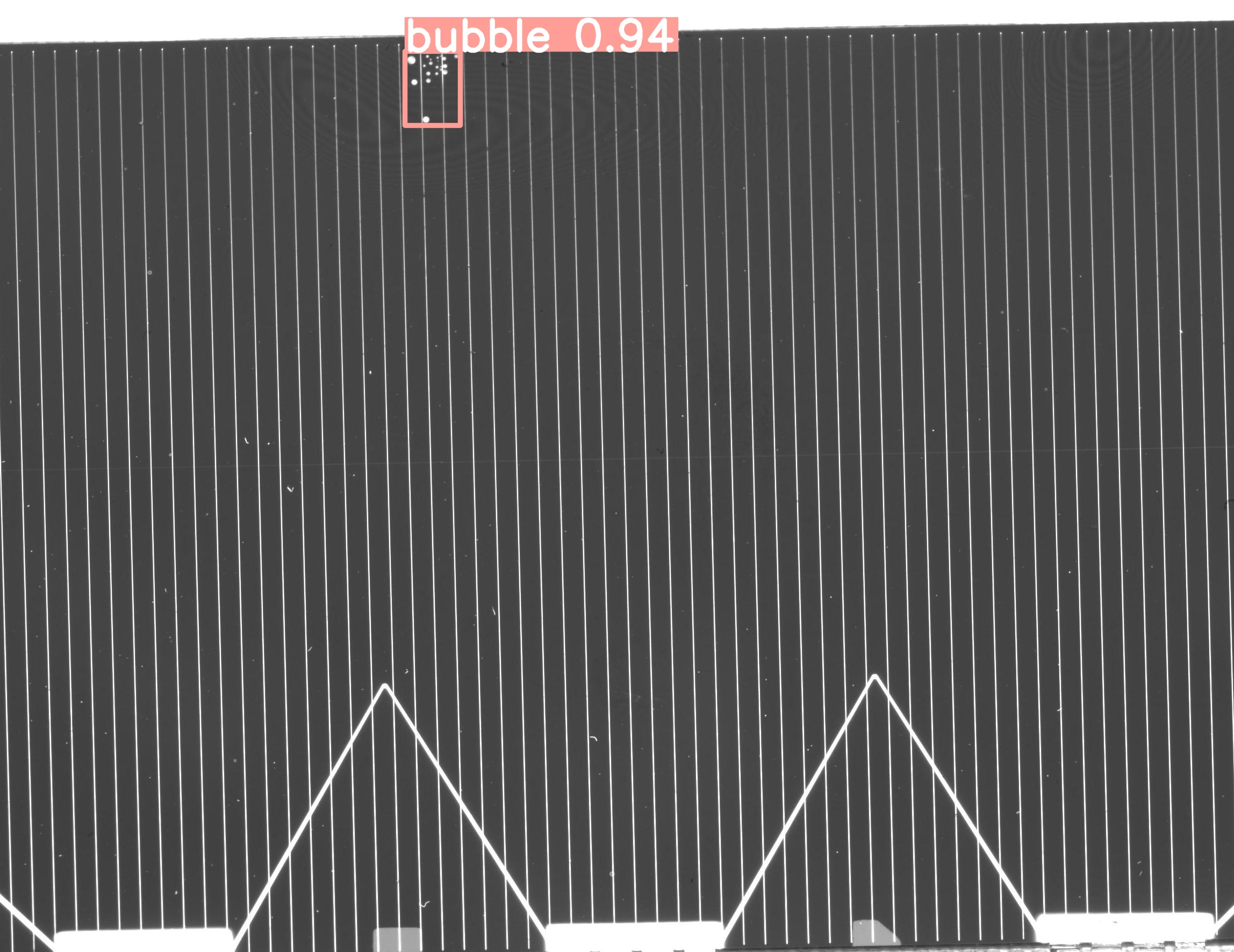

Supplement: S1 Dataset — (ZIP) [file pone.0304819.s001.zip › 6930.jpg]

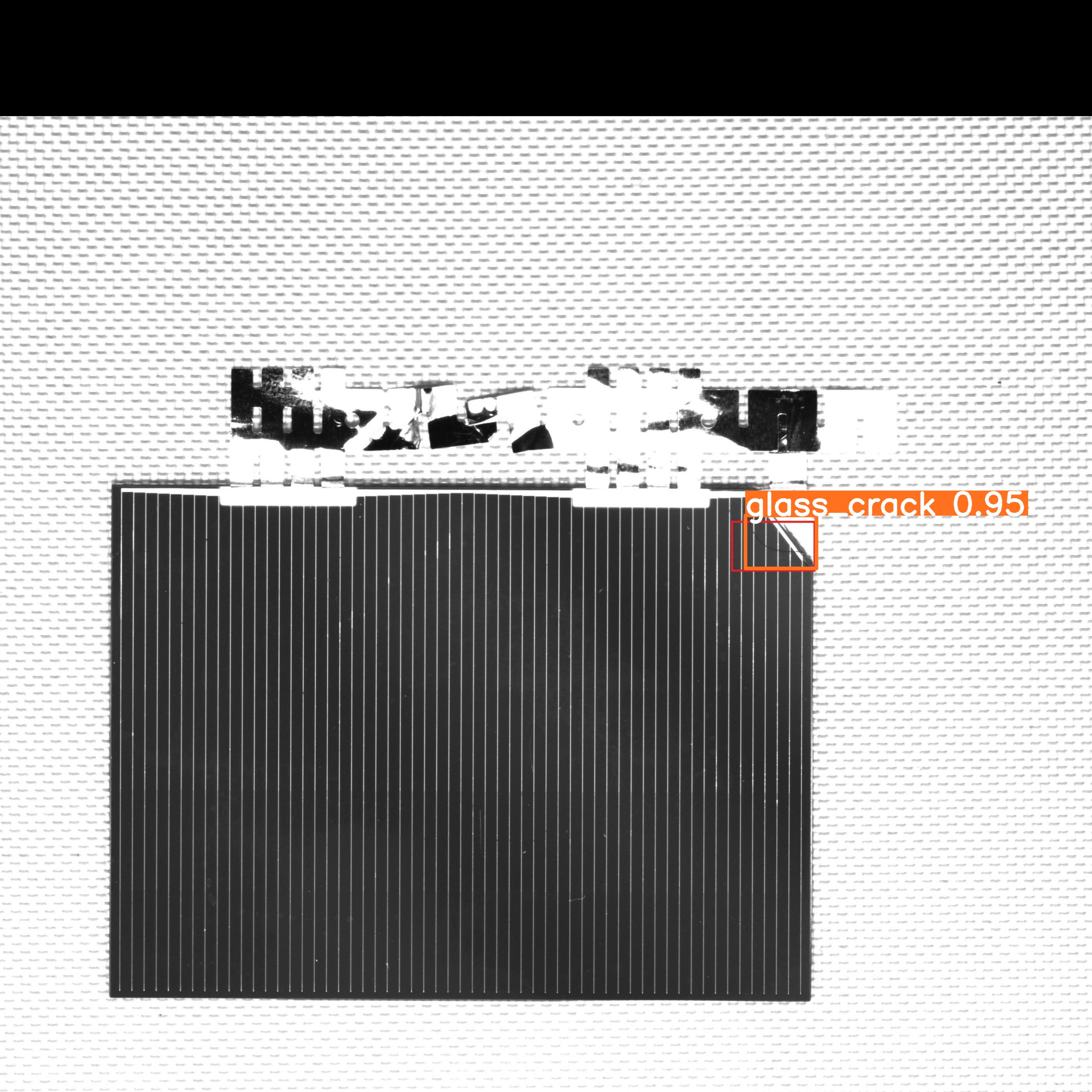

Supplement: S1 Dataset — (ZIP) [file pone.0304819.s001.zip › 7002.jpg]

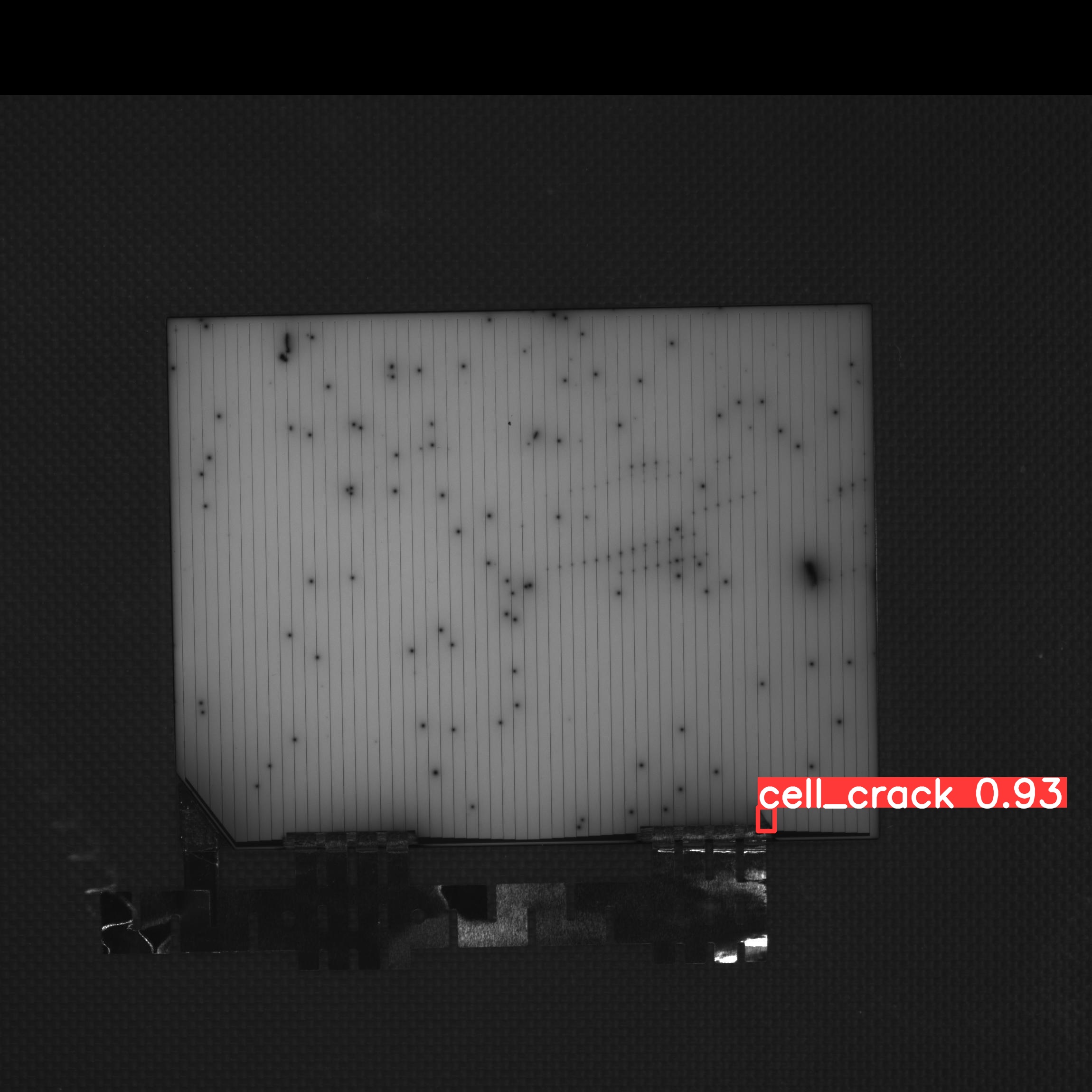

Supplement: S1 Dataset — (ZIP) [file pone.0304819.s001.zip › 7196.jpg]

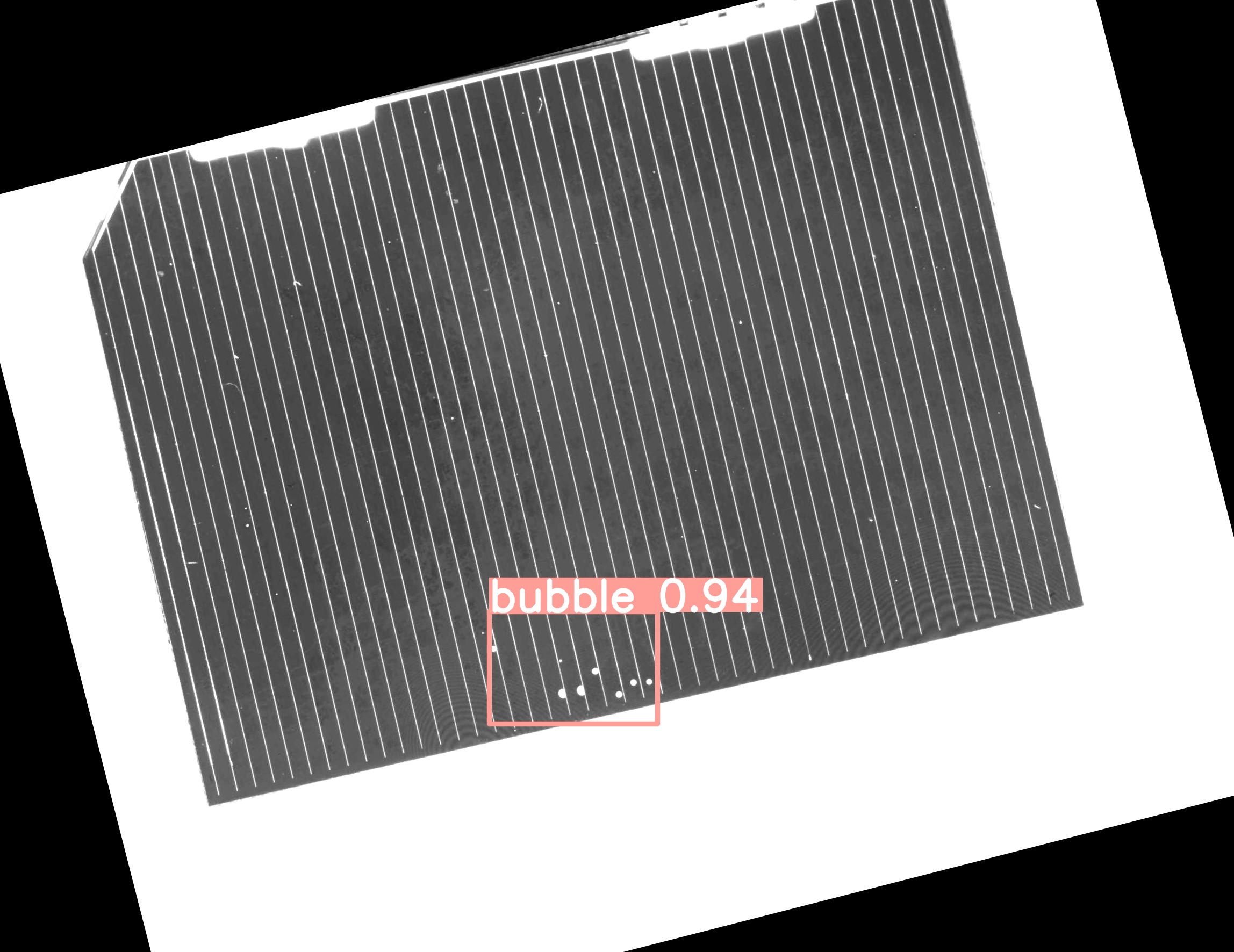

Supplement: S1 Dataset — (ZIP) [file pone.0304819.s001.zip › 7262.jpg]

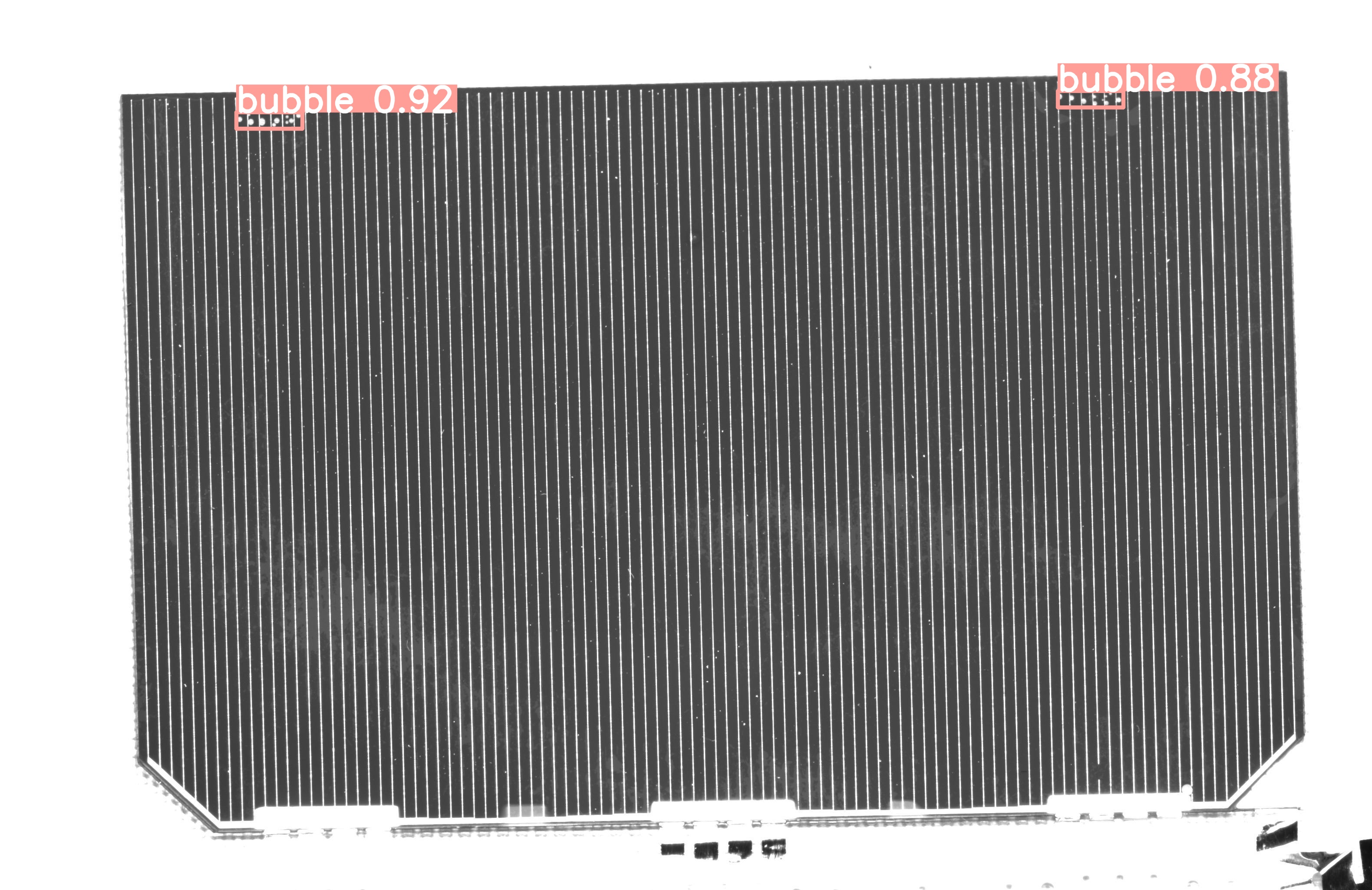

Supplement: S1 Dataset — (ZIP) [file pone.0304819.s001.zip › 7409.jpg]

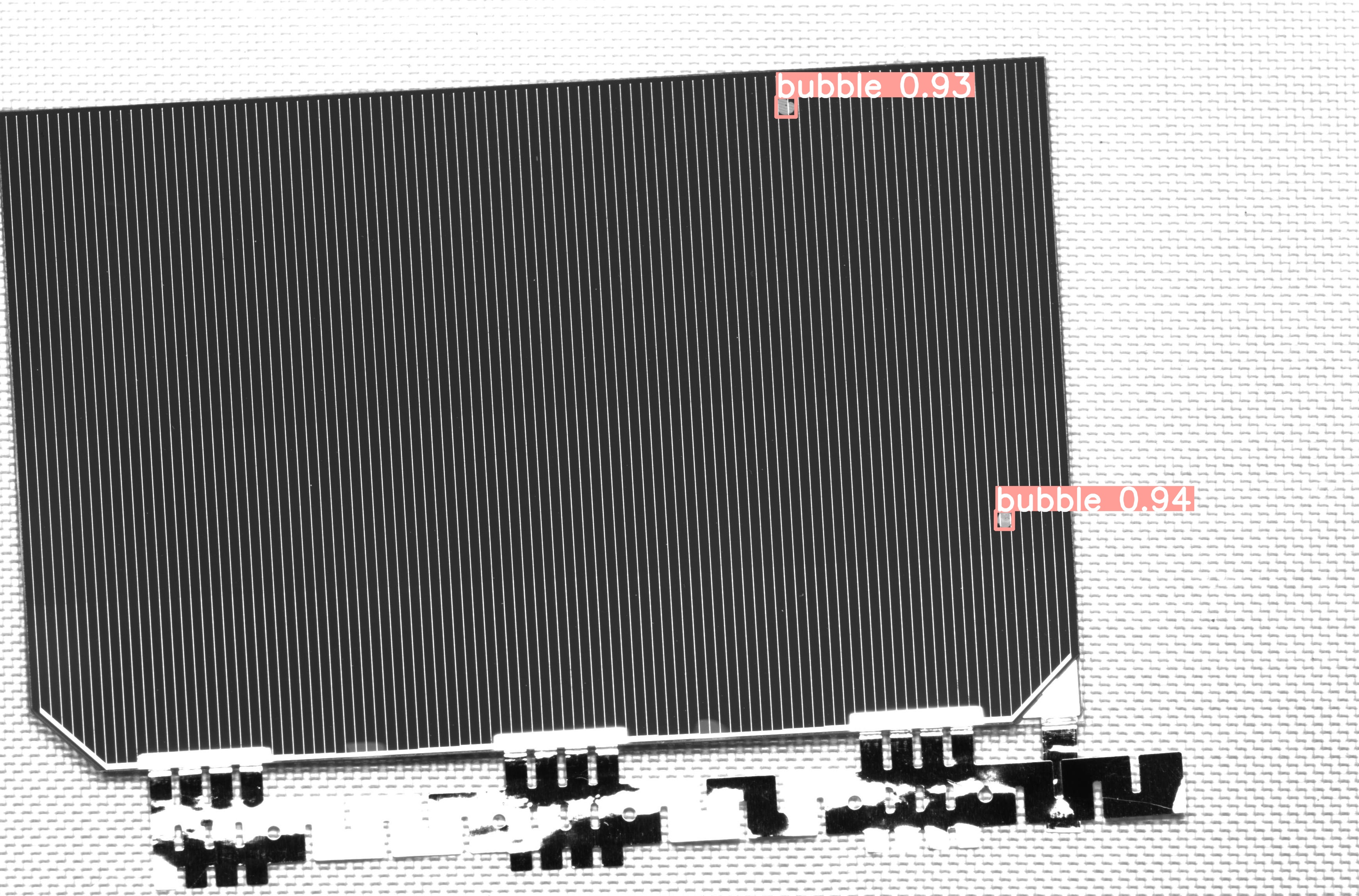

Supplement: S1 Dataset — (ZIP) [file pone.0304819.s001.zip › 7672.jpg]

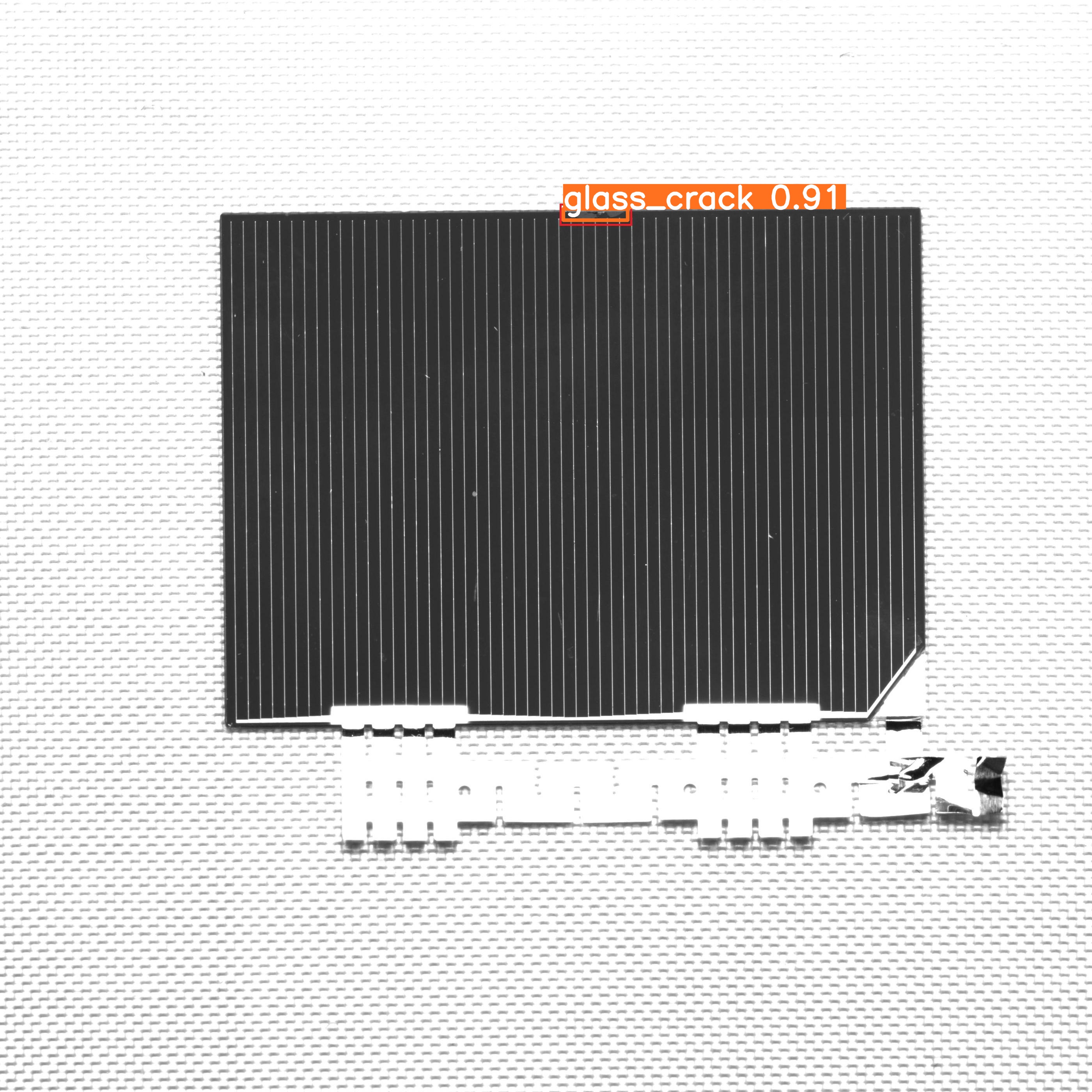

Supplement: S1 Dataset — (ZIP) [file pone.0304819.s001.zip › 7769.jpg]

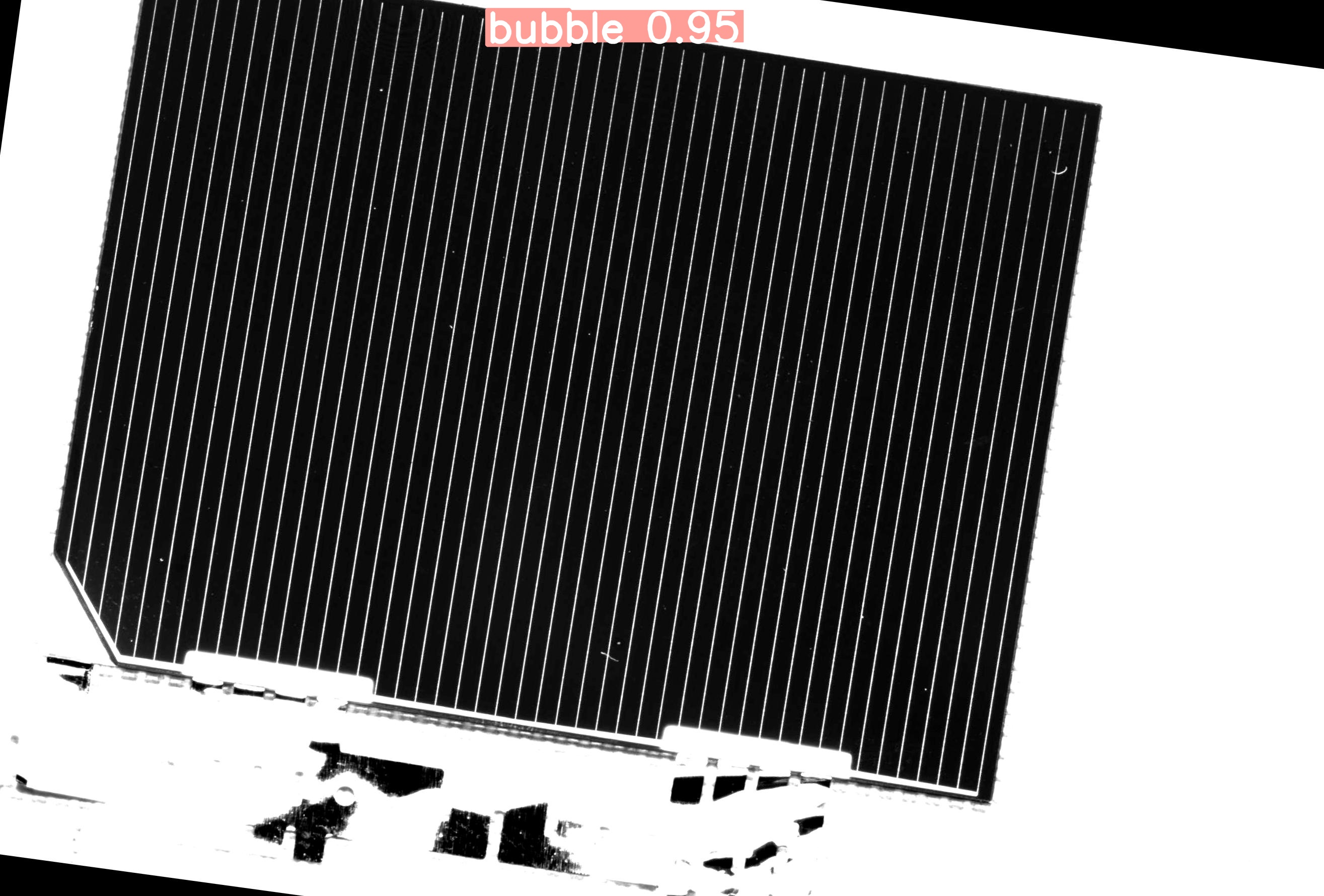

Supplement: S1 Dataset — (ZIP) [file pone.0304819.s001.zip › 7862.jpg]

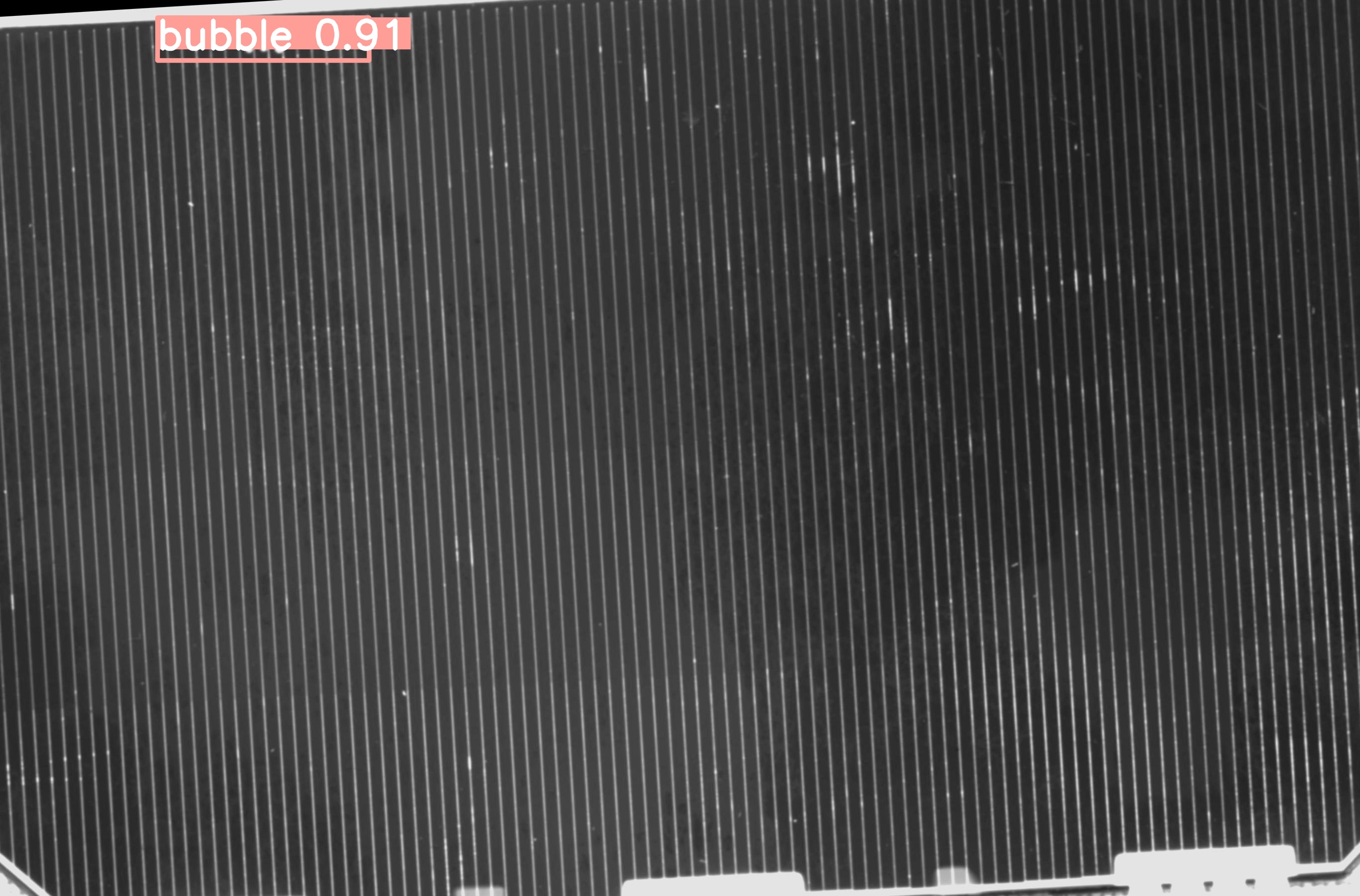

Supplement: S1 Dataset — (ZIP) [file pone.0304819.s001.zip › 8100.jpg]

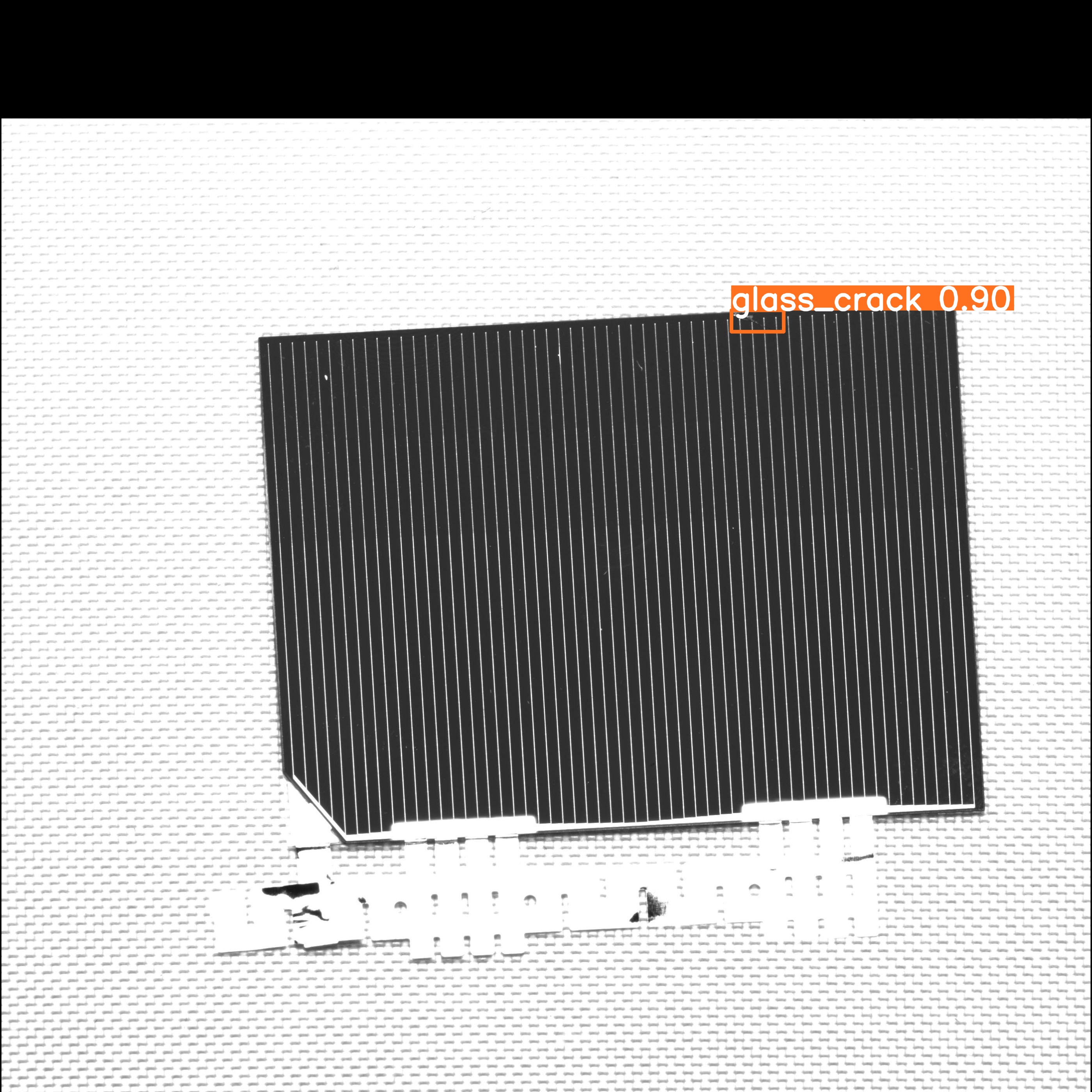

Supplement: S1 Dataset — (ZIP) [file pone.0304819.s001.zip › 8173.jpg]

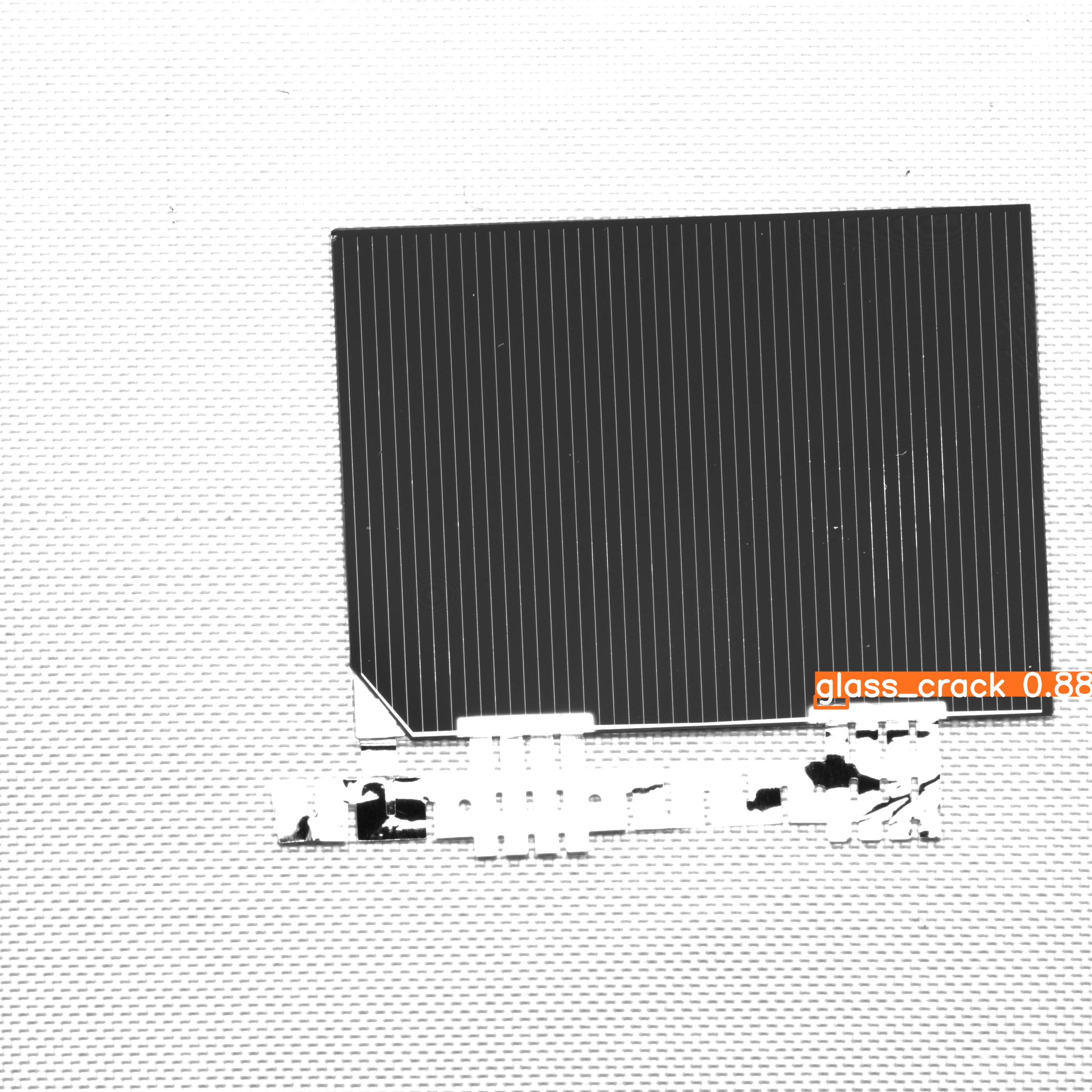

Supplement: S1 Dataset — (ZIP) [file pone.0304819.s001.zip › 8269.jpg]

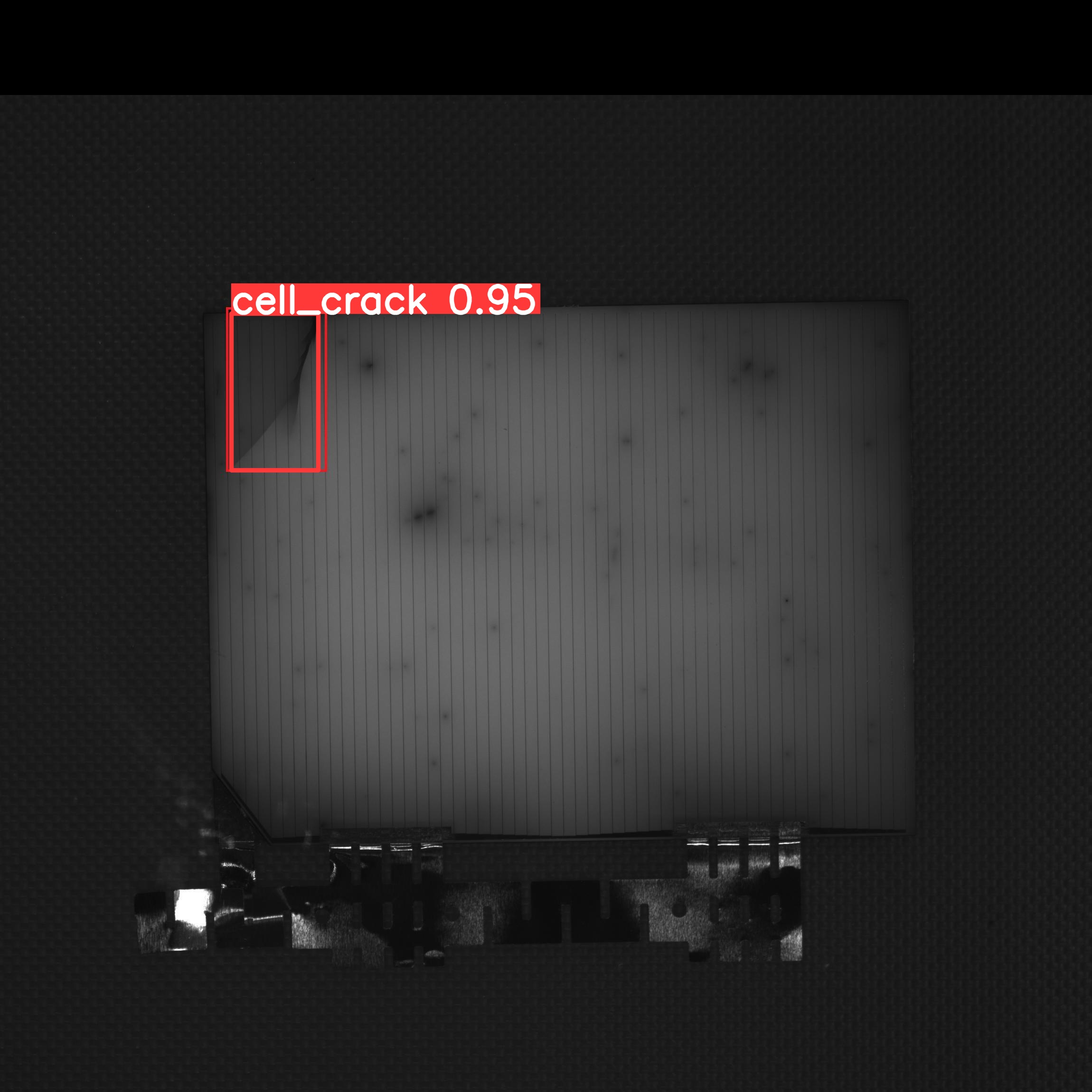

Supplement: S1 Dataset — (ZIP) [file pone.0304819.s001.zip › 8357.jpg]

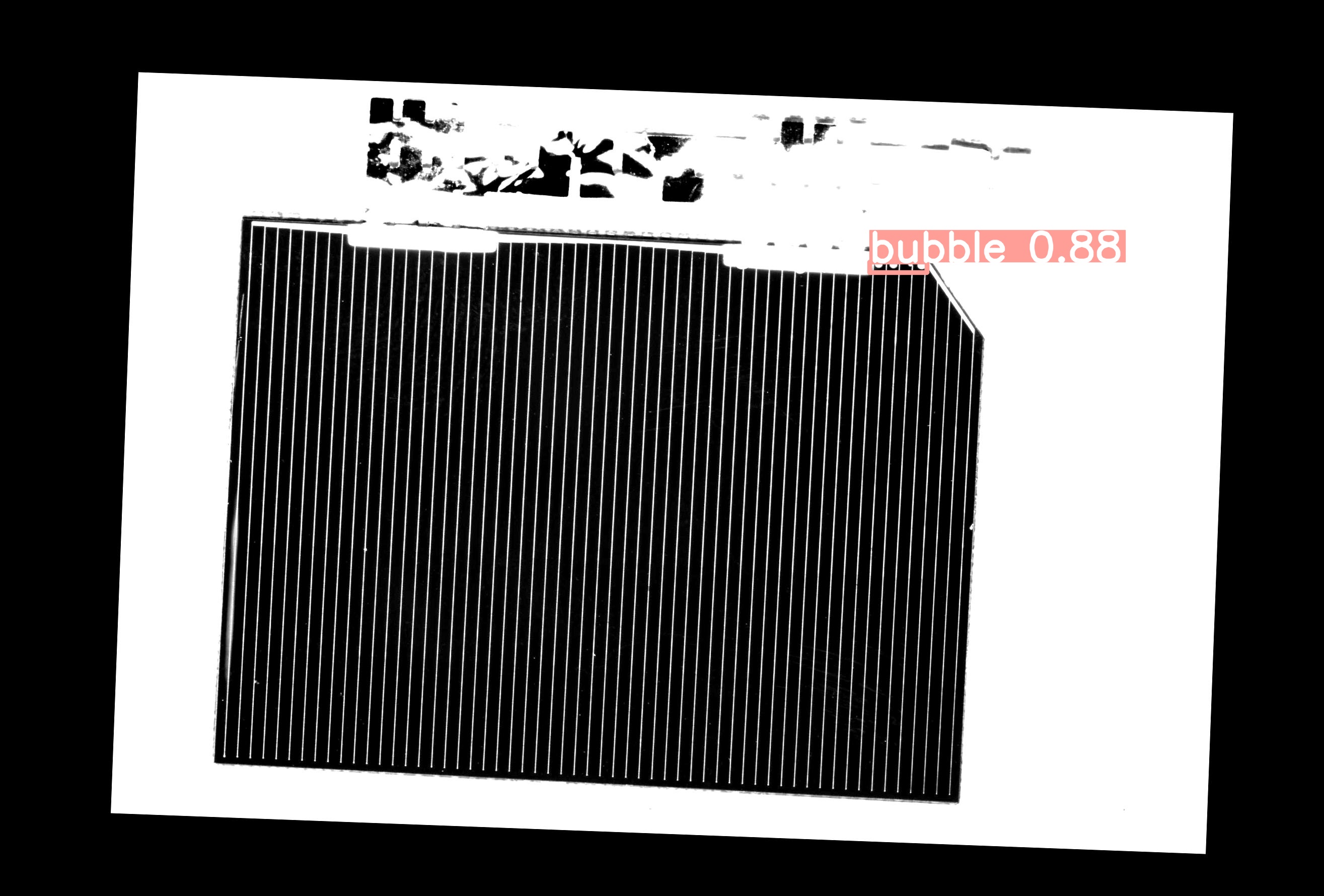

Supplement: S1 Dataset — (ZIP) [file pone.0304819.s001.zip › 8421.jpg]

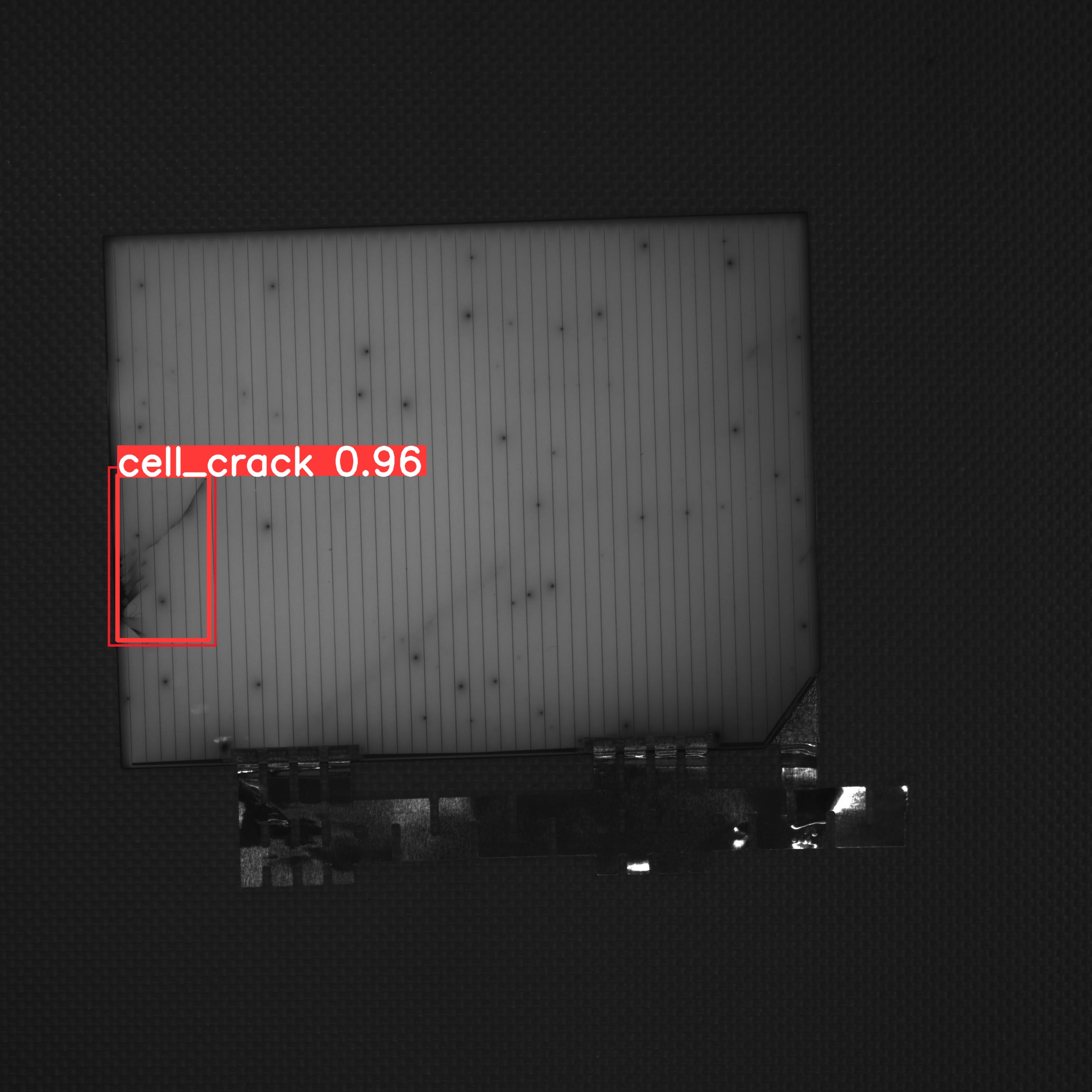

Supplement: S1 Dataset — (ZIP) [file pone.0304819.s001.zip › 8500.jpg]

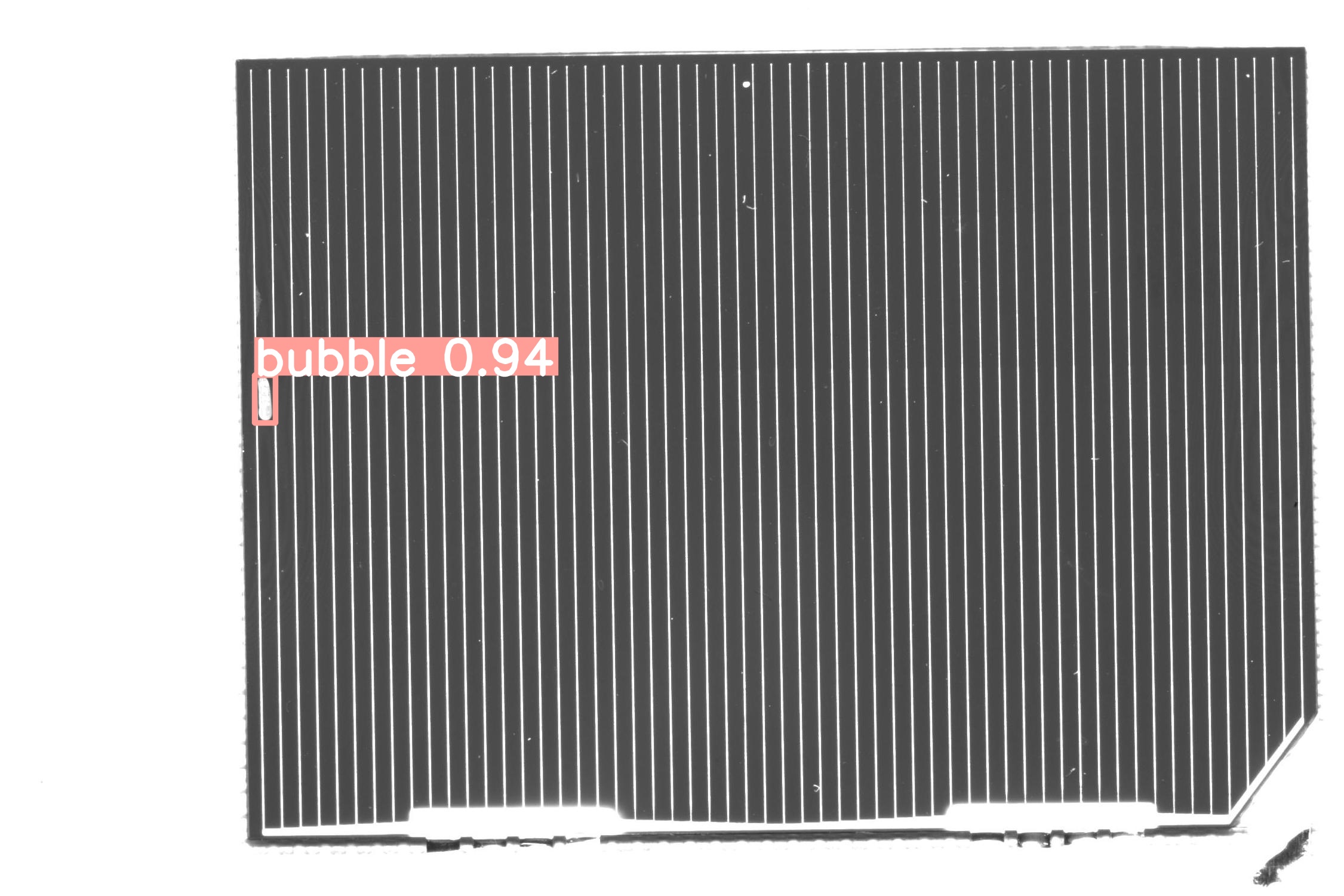

Supplement: S1 Dataset — (ZIP) [file pone.0304819.s001.zip › 8576.jpg]

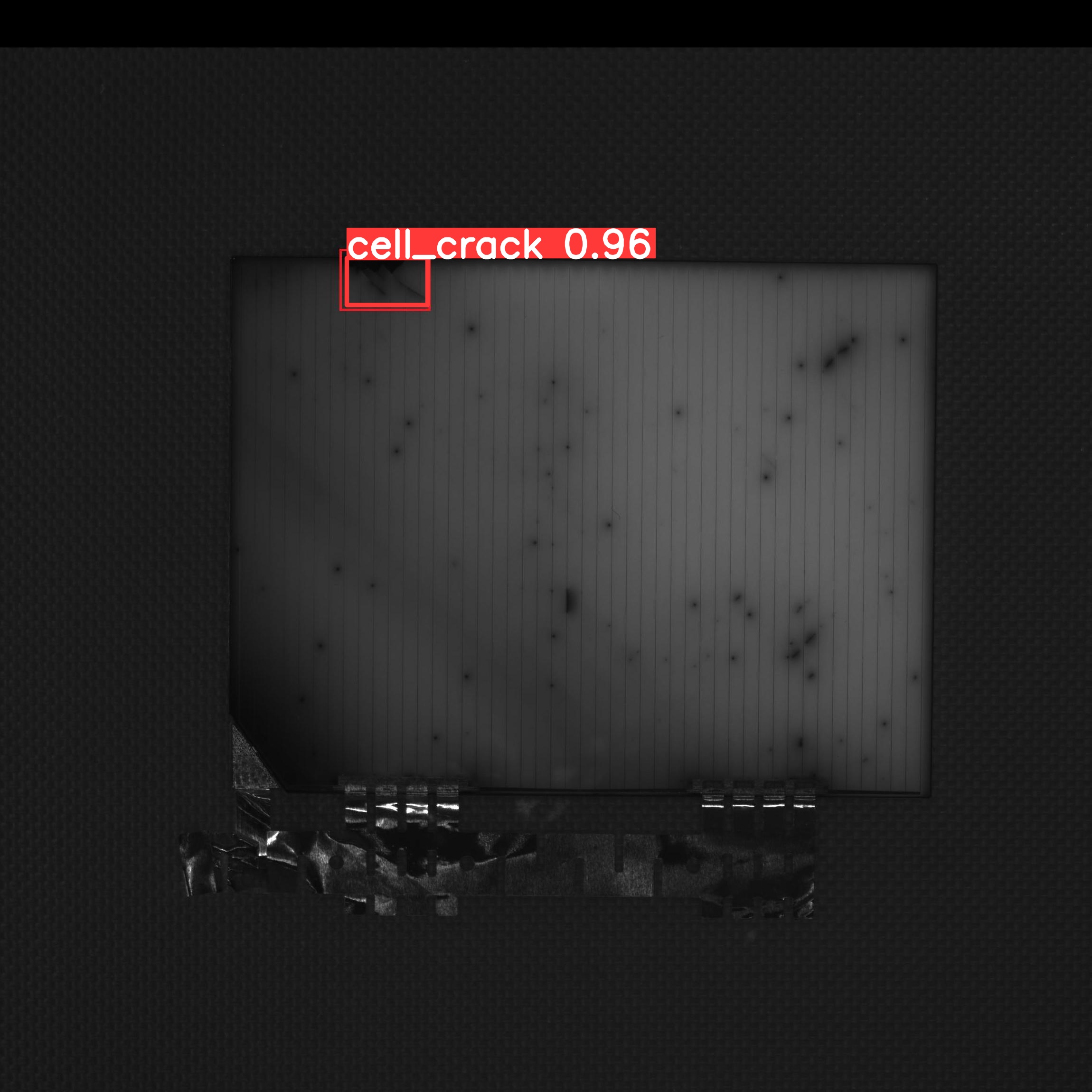

Supplement: S1 Dataset — (ZIP) [file pone.0304819.s001.zip › 8671.jpg]

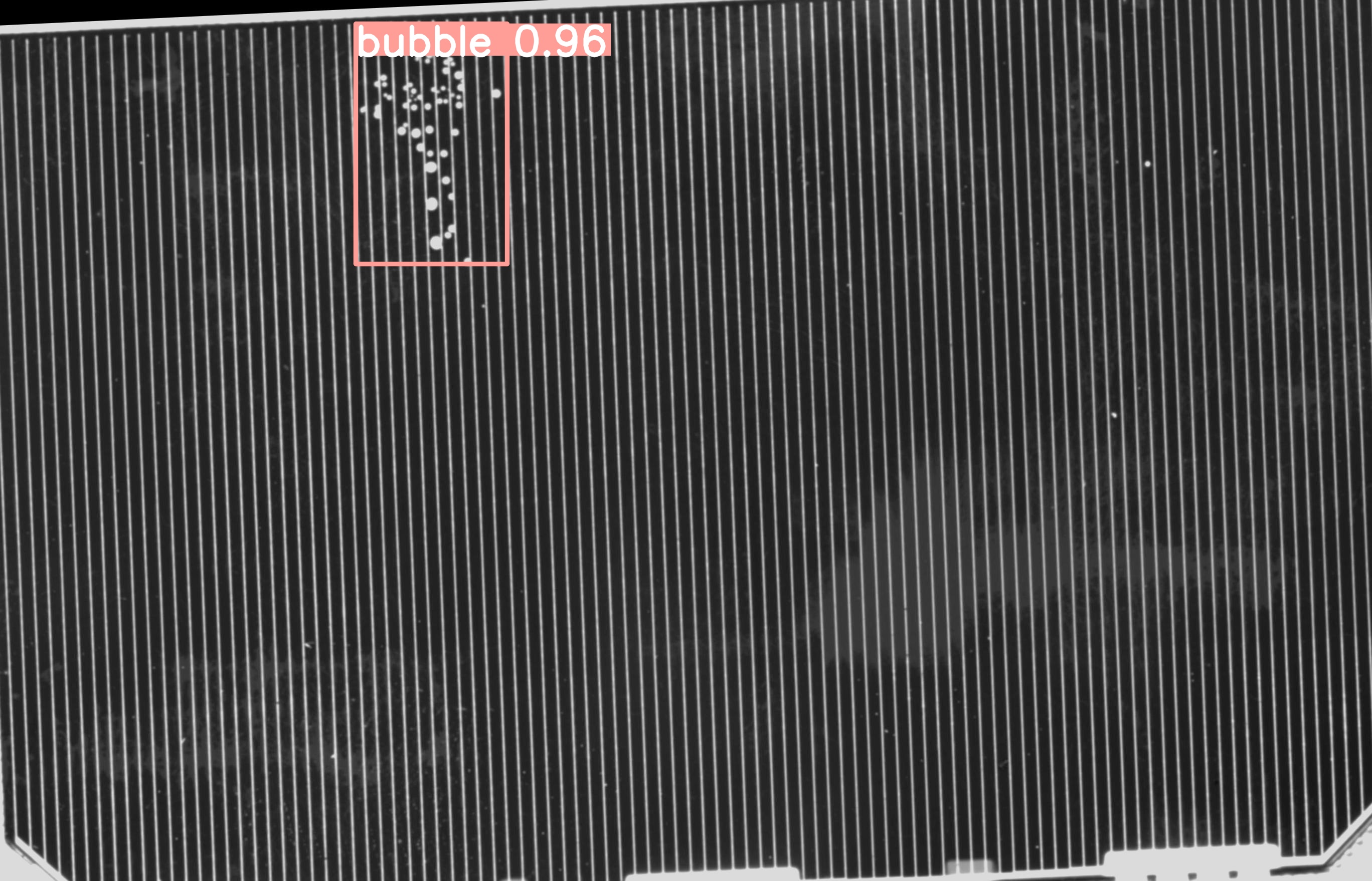

Supplement: S1 Dataset — (ZIP) [file pone.0304819.s001.zip › 8786.jpg]

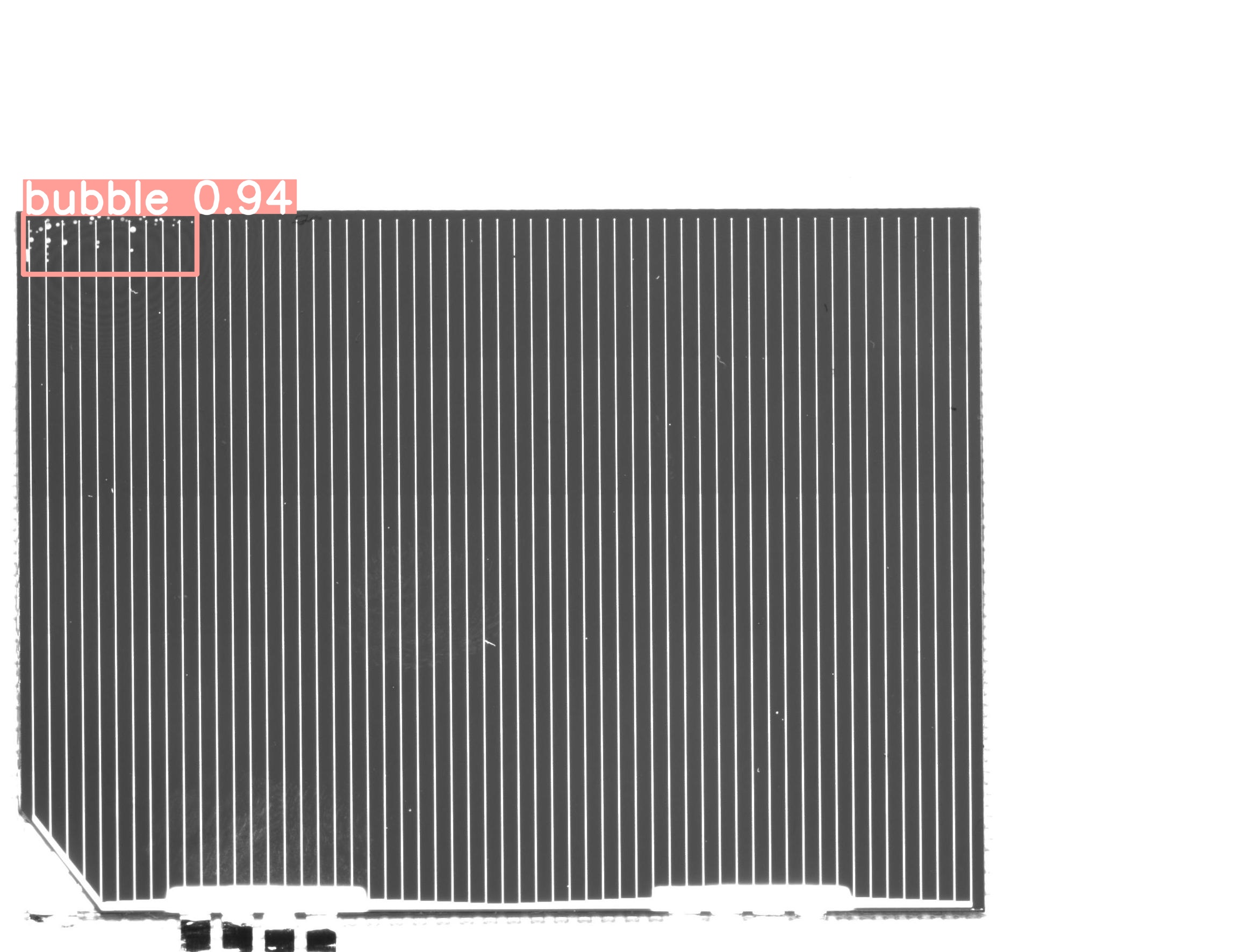

Supplement: S1 Dataset — (ZIP) [file pone.0304819.s001.zip › 8880.jpg]

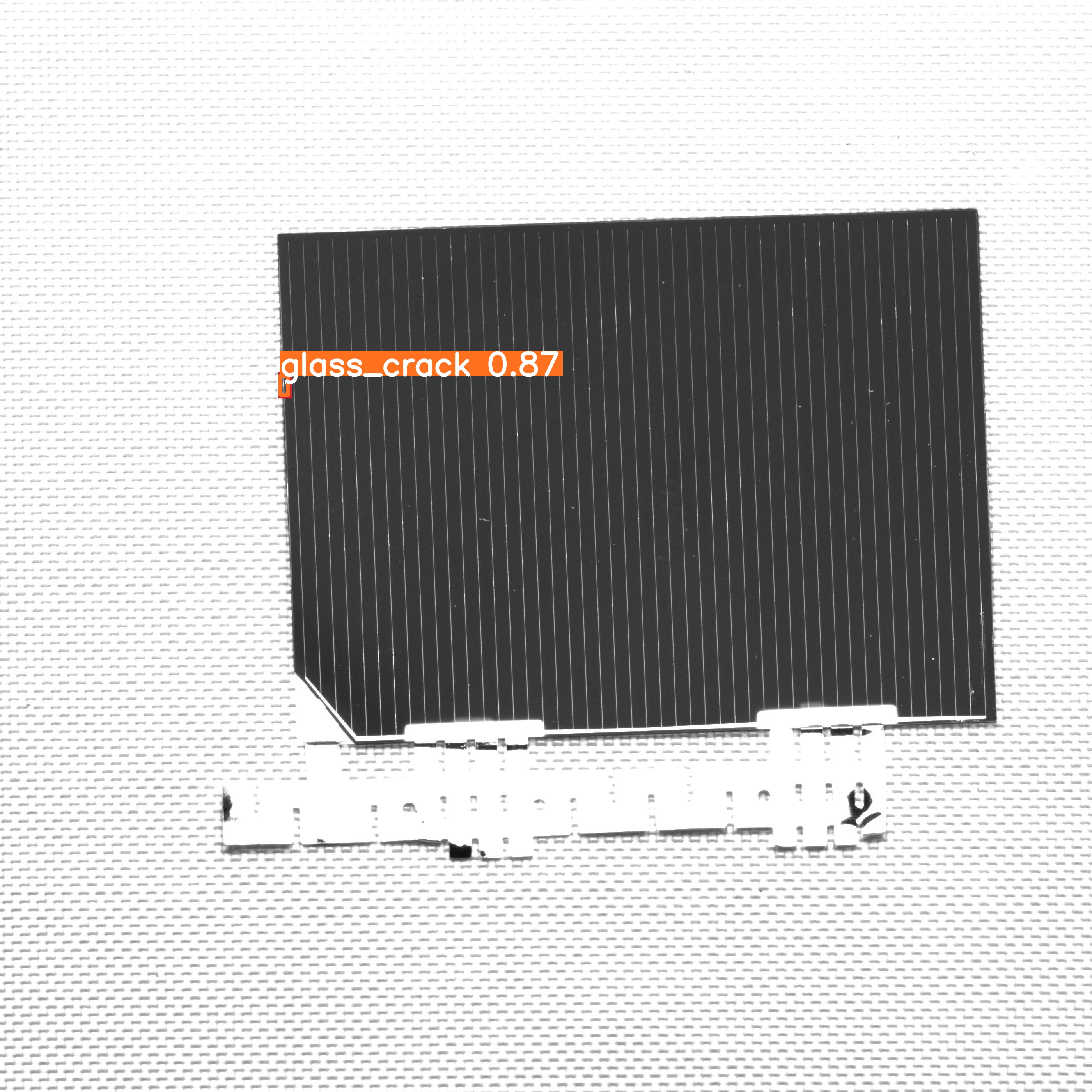

Supplement: S1 Dataset — (ZIP) [file pone.0304819.s001.zip › 9048.jpg]

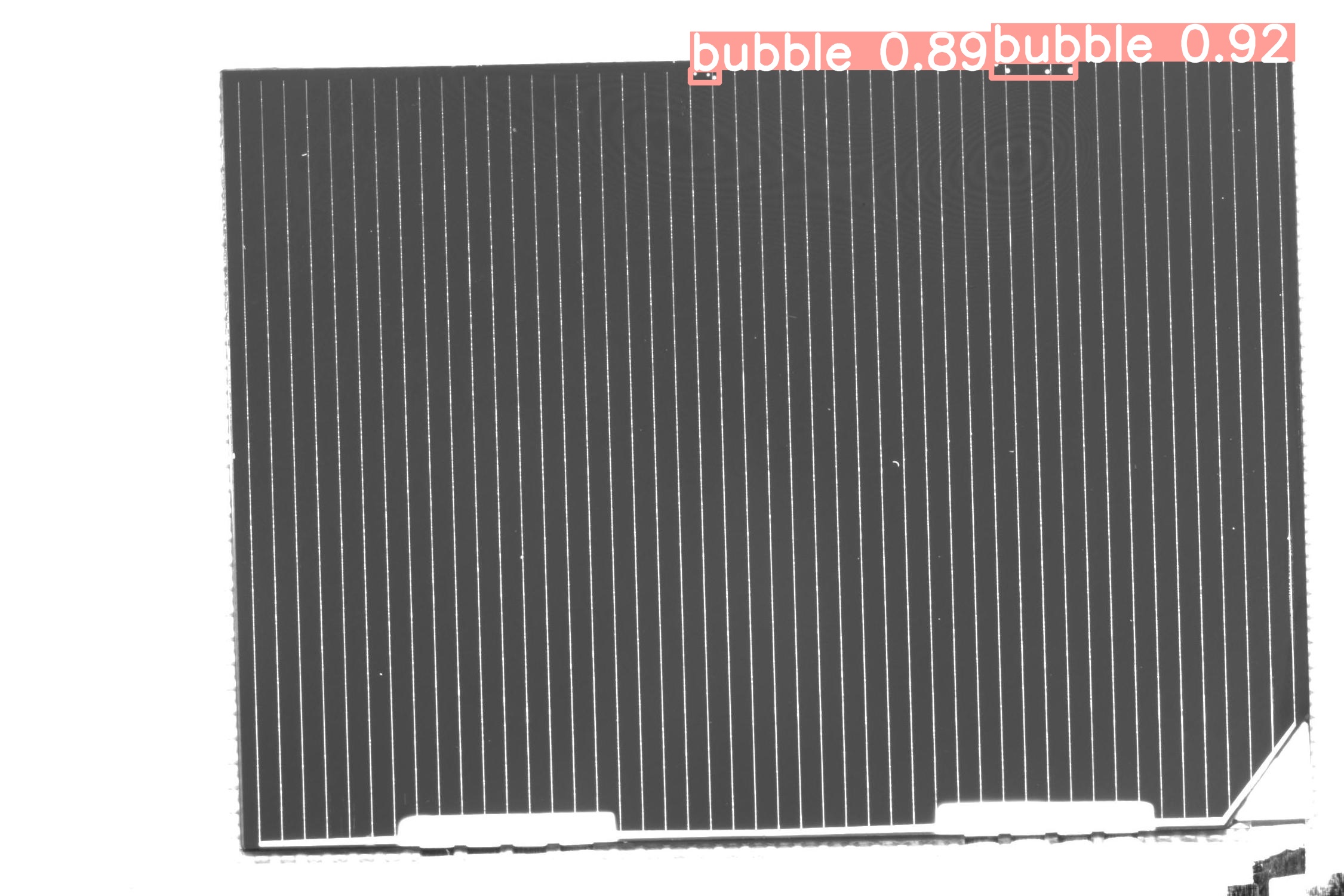

Supplement: S1 Dataset — (ZIP) [file pone.0304819.s001.zip › 9141.jpg]

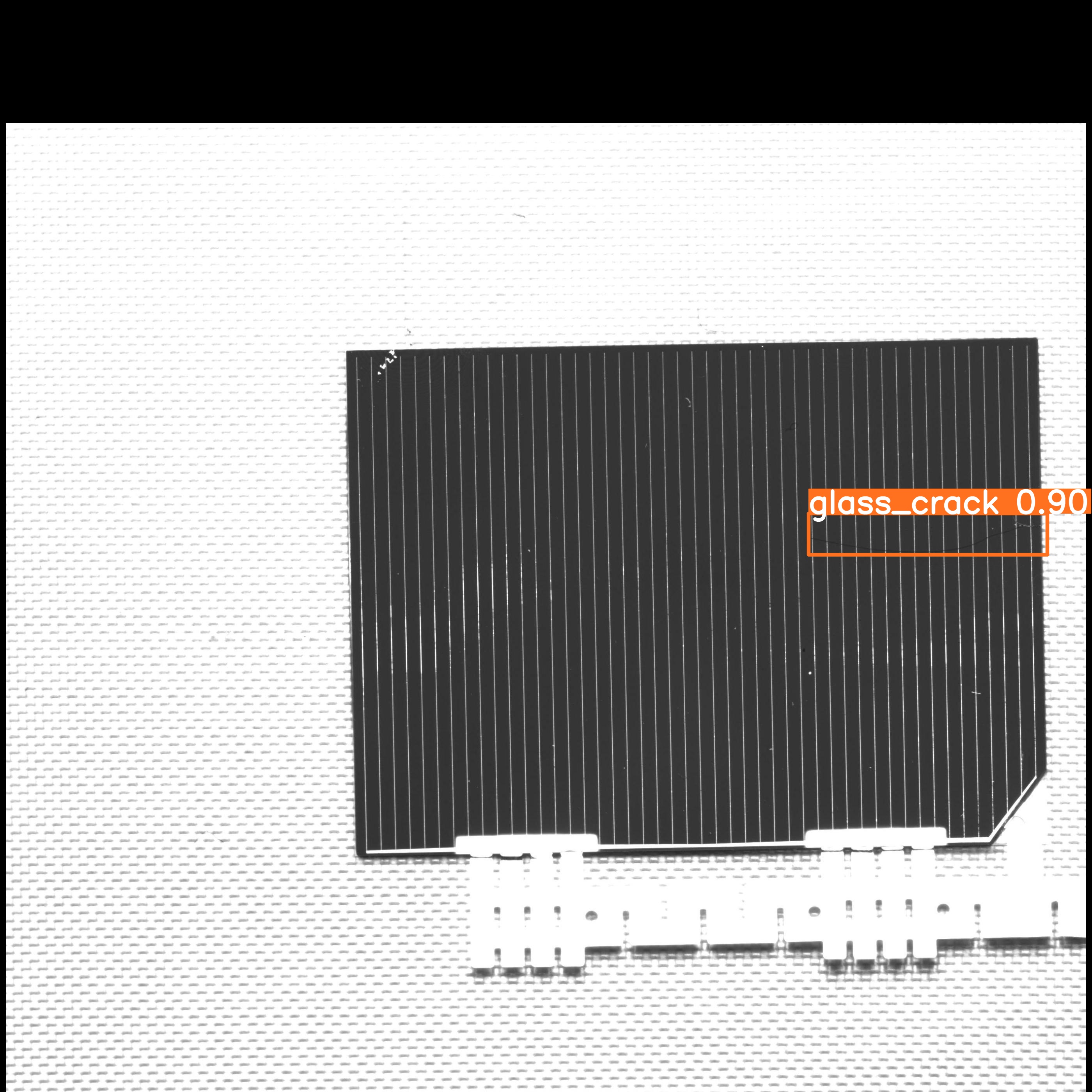

Supplement: S1 Dataset — (ZIP) [file pone.0304819.s001.zip › 9243.jpg]

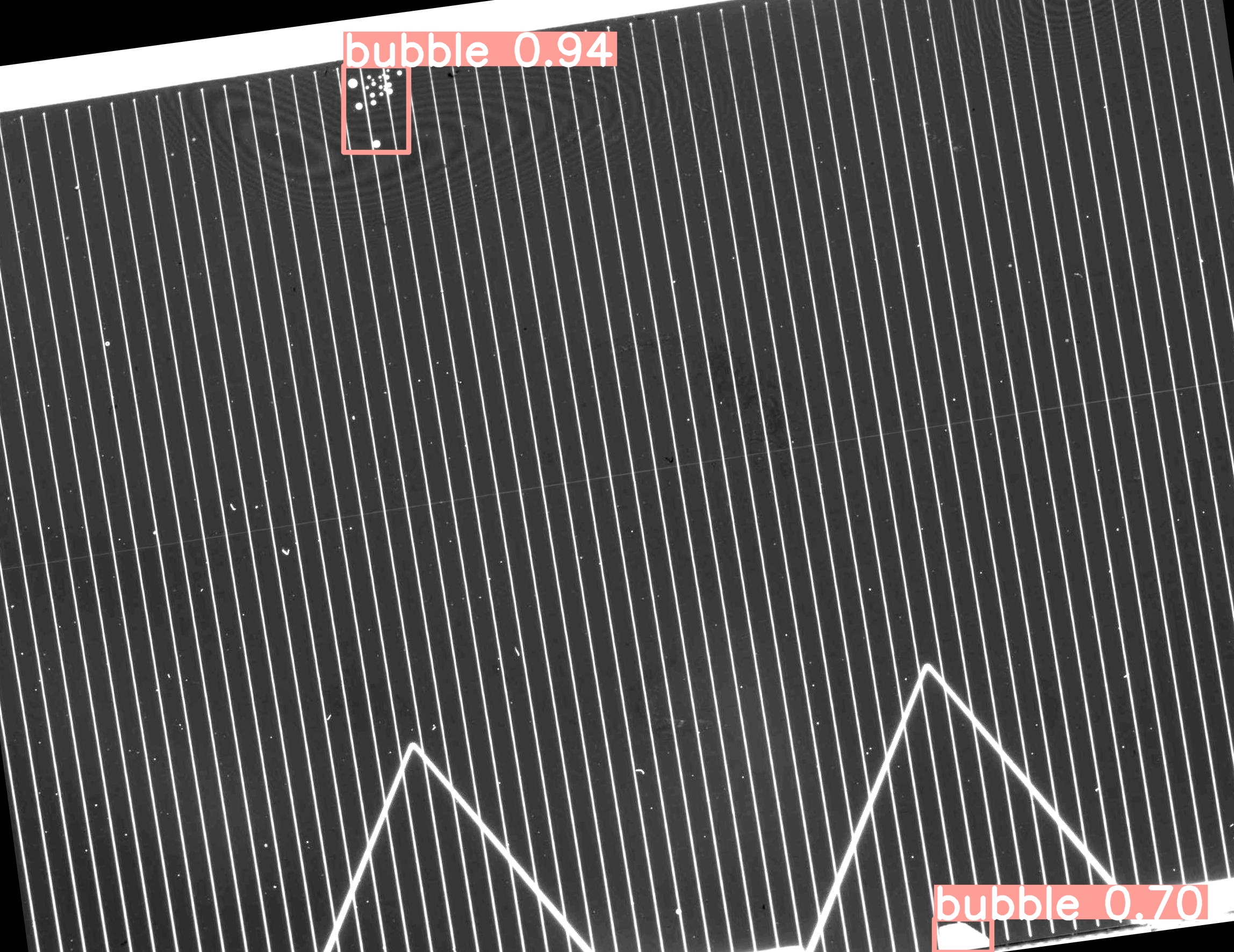

Supplement: S1 Dataset — (ZIP) [file pone.0304819.s001.zip › 9333.jpg]

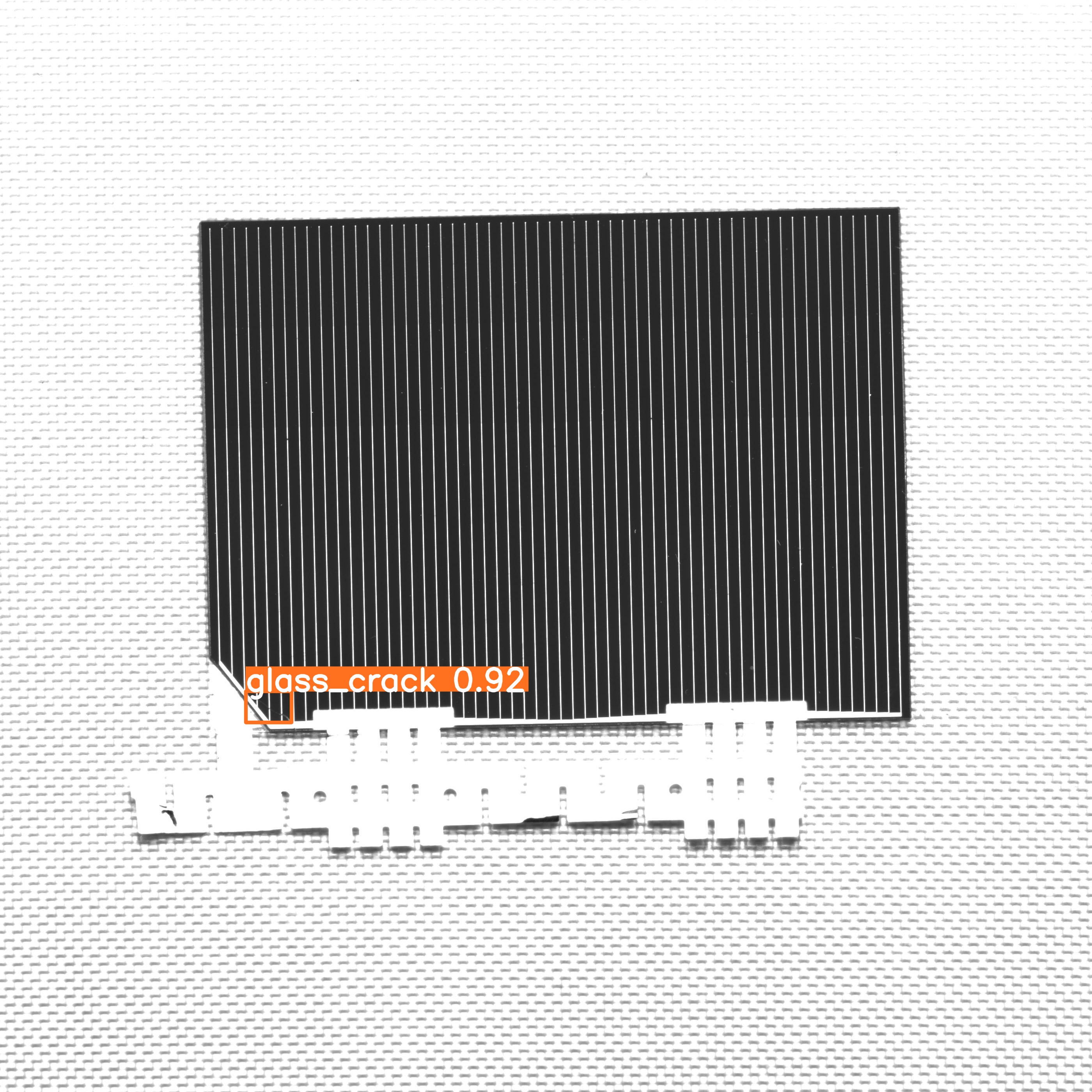

Supplement: S1 Dataset — (ZIP) [file pone.0304819.s001.zip › 9480.jpg]

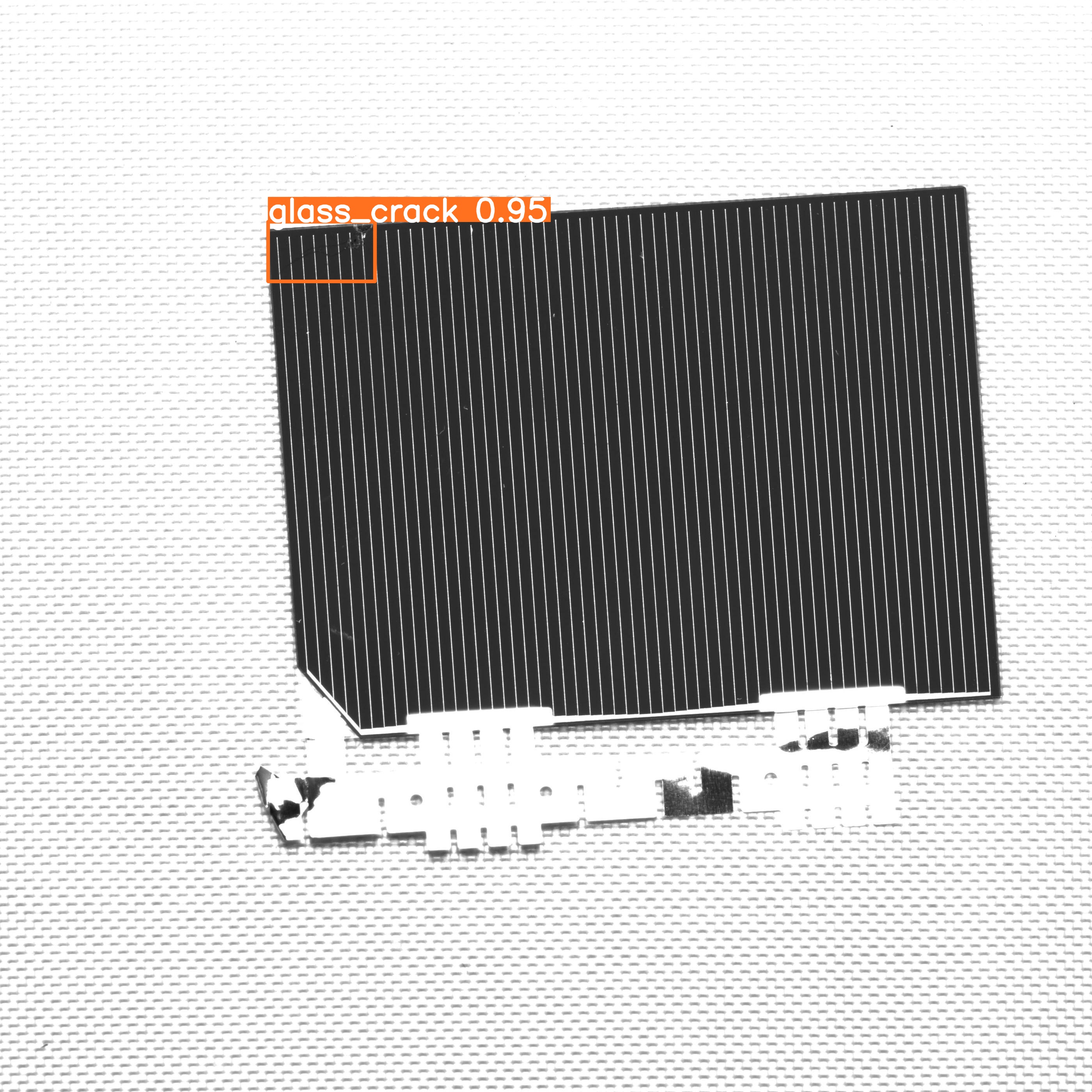

Supplement: S1 Dataset — (ZIP) [file pone.0304819.s001.zip › 9643.jpg]
