# Supplementary figures and images for: Research on multi-defects classification detection method for solar cells based on deep learning (part 1 of 2)
Source: PLoS One. 2024 Jun 21;19(6):e0304819. doi: 10.1371/journal.pone.0304819 (PMC11192367; doi:10.1371/journal.pone.0304819)

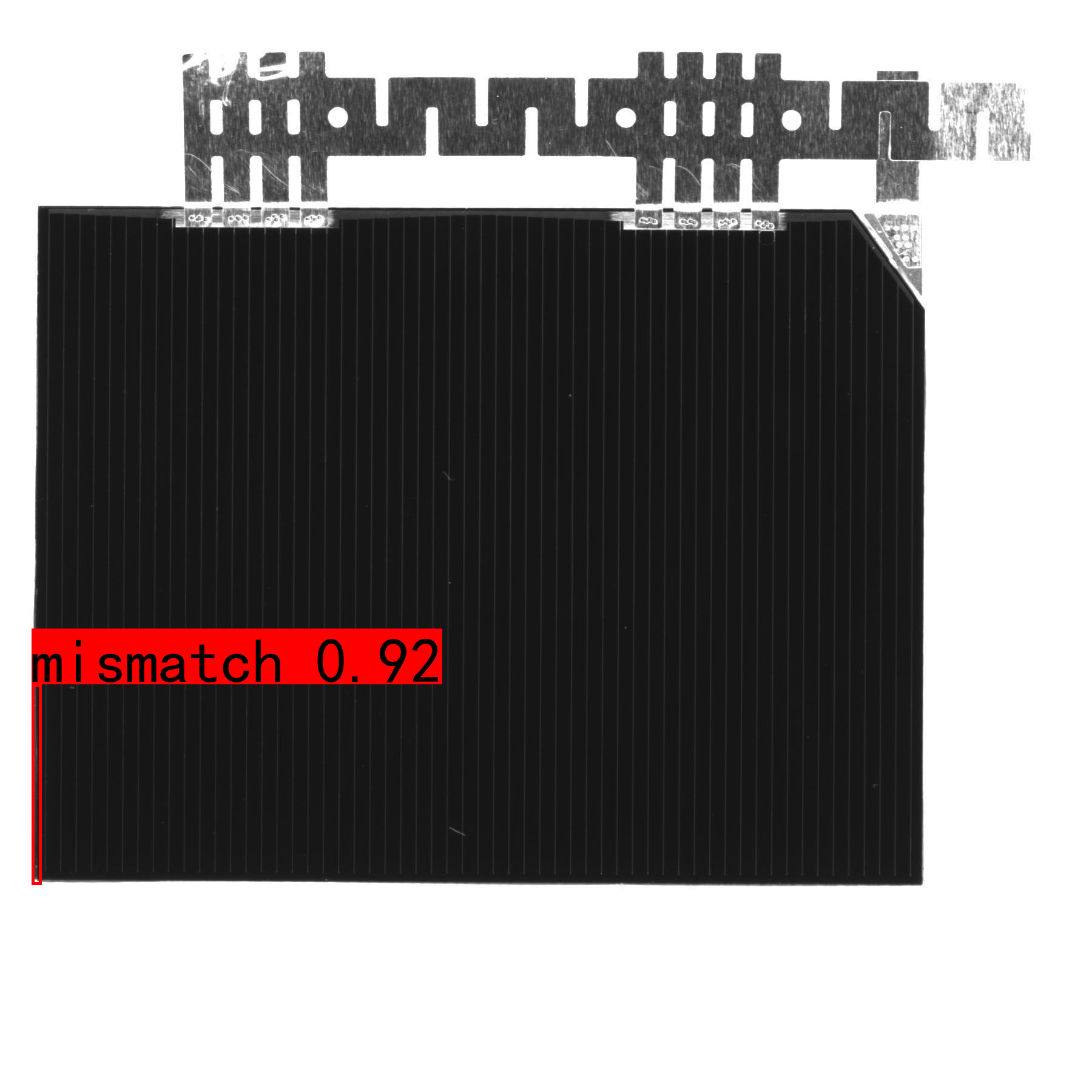

Supplement: S1 Dataset — (ZIP) [file pone.0304819.s001.zip › 00080mismatch_origin-copy_000001.png]

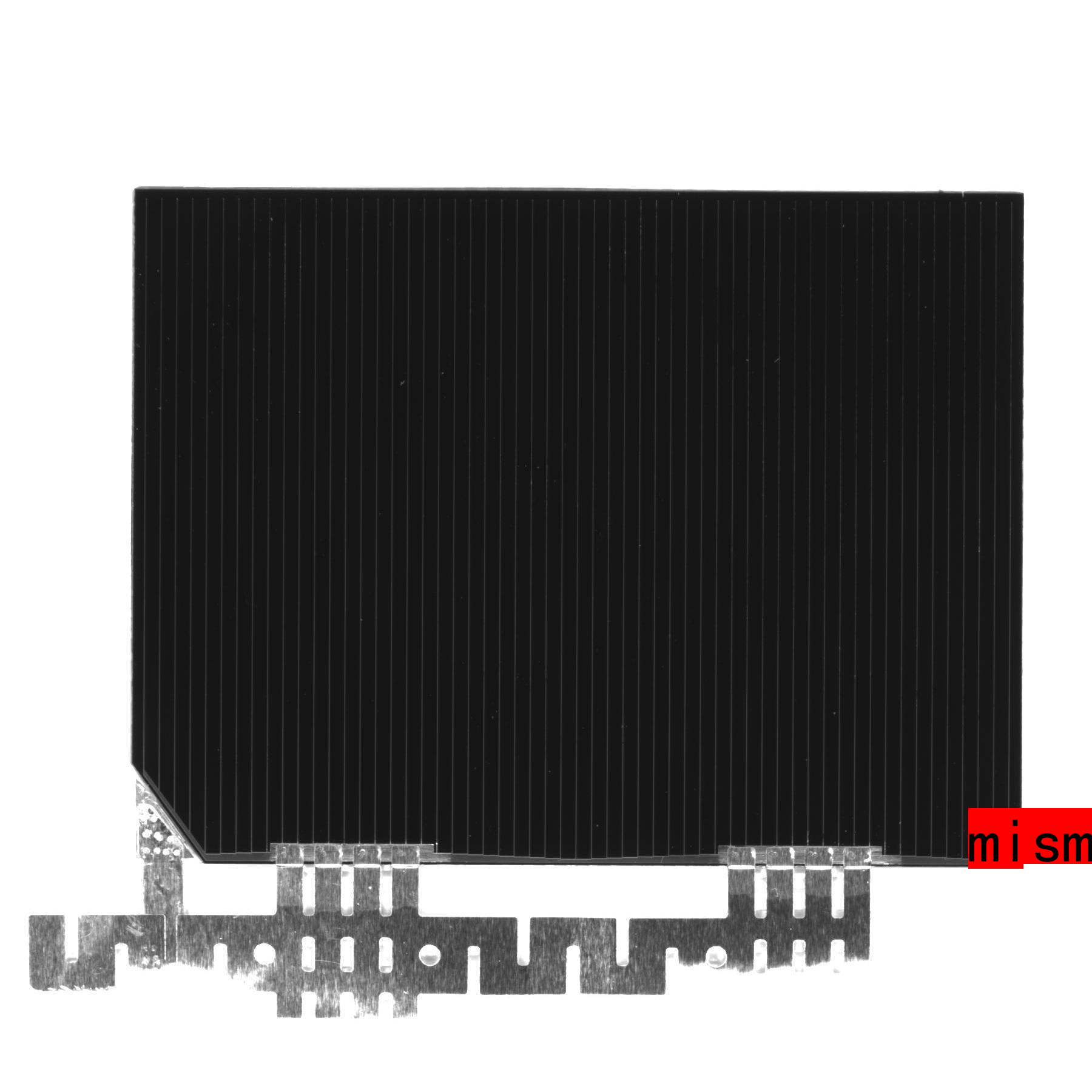

Supplement: S1 Dataset — (ZIP) [file pone.0304819.s001.zip › 00080mismatch_updown.png]

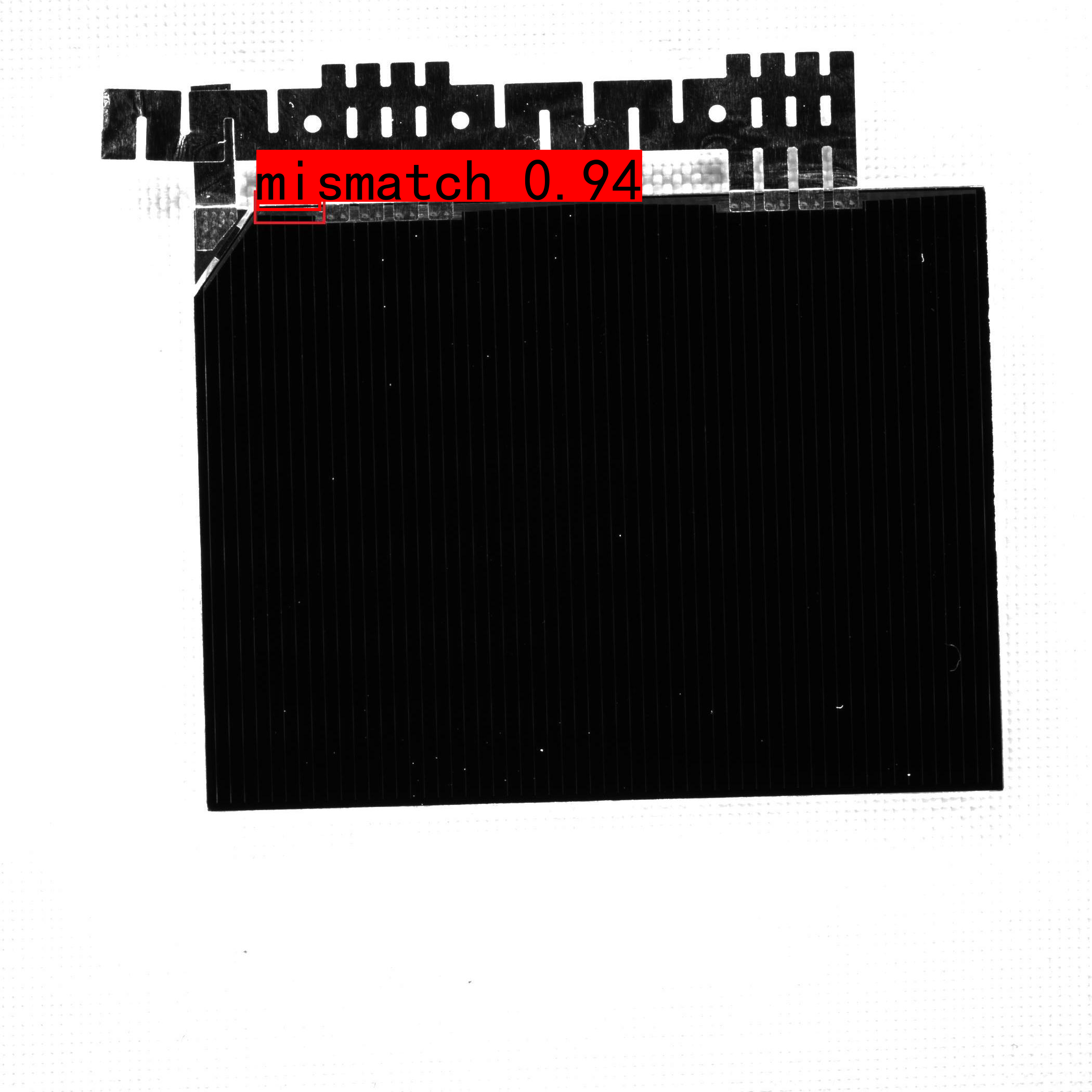

Supplement: S1 Dataset — (ZIP) [file pone.0304819.s001.zip › 00081mismatch_origin-copy_000001.png]

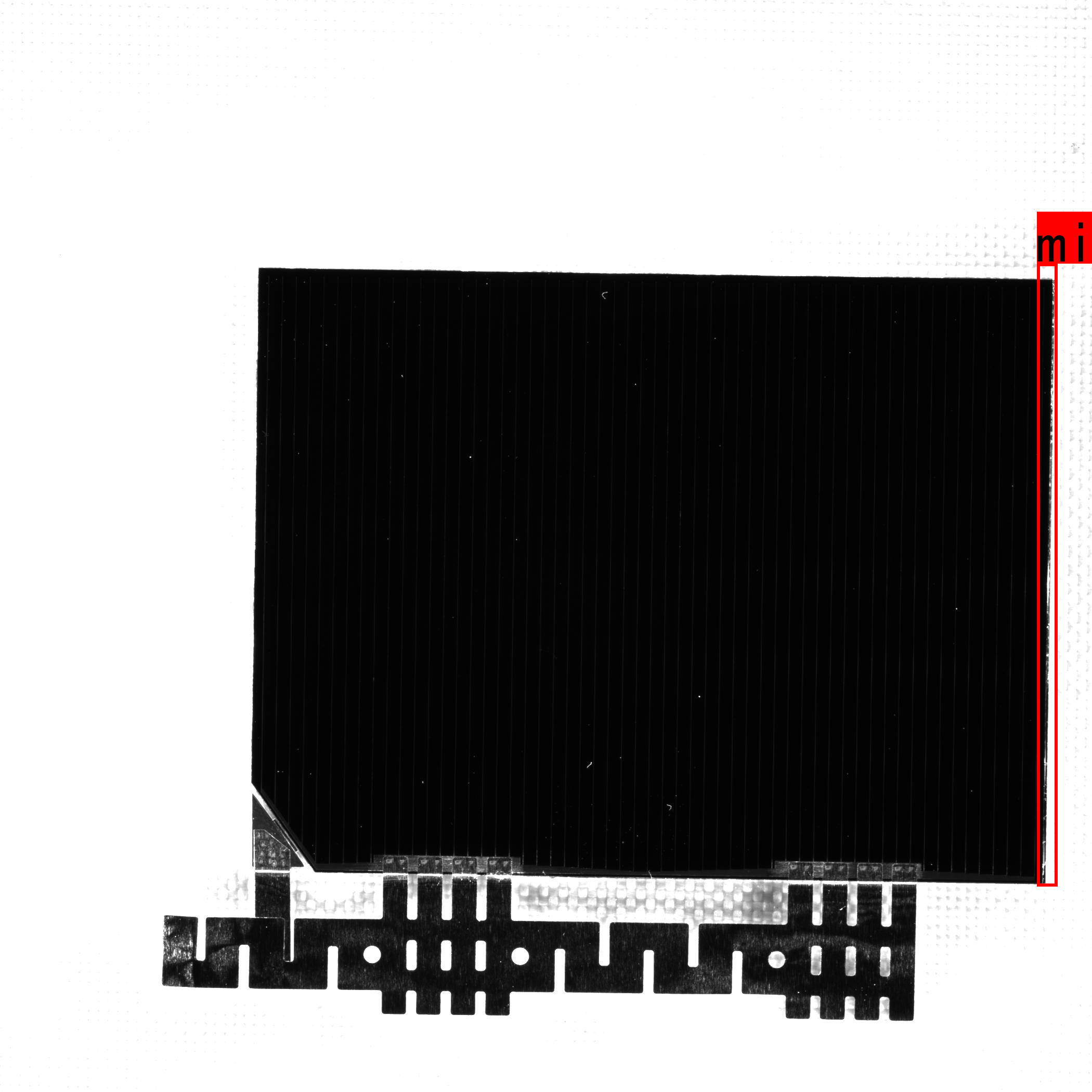

Supplement: S1 Dataset — (ZIP) [file pone.0304819.s001.zip › 00091mismatch_updown.png]

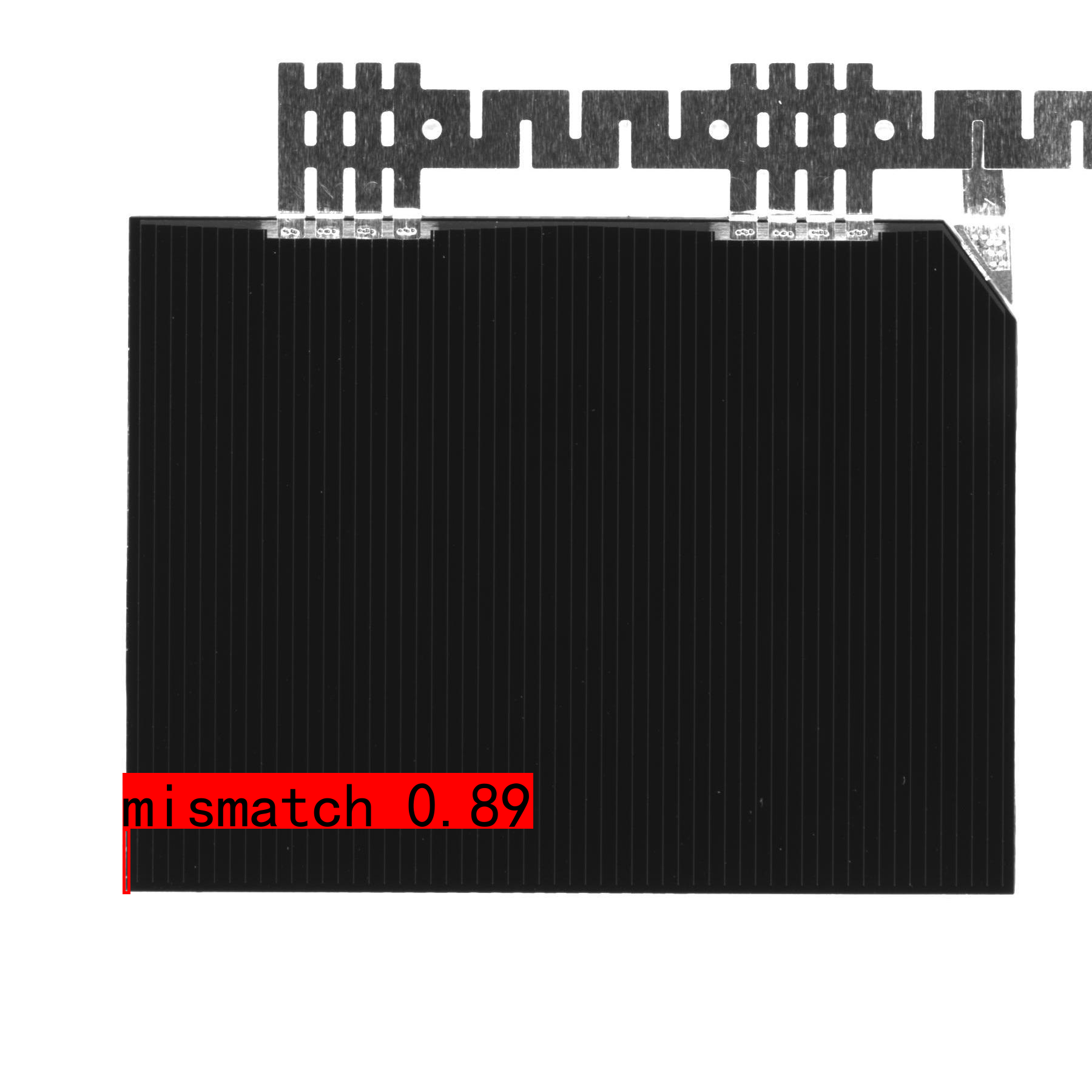

Supplement: S1 Dataset — (ZIP) [file pone.0304819.s001.zip › 00092mismatch_origin-copy_000001.png]

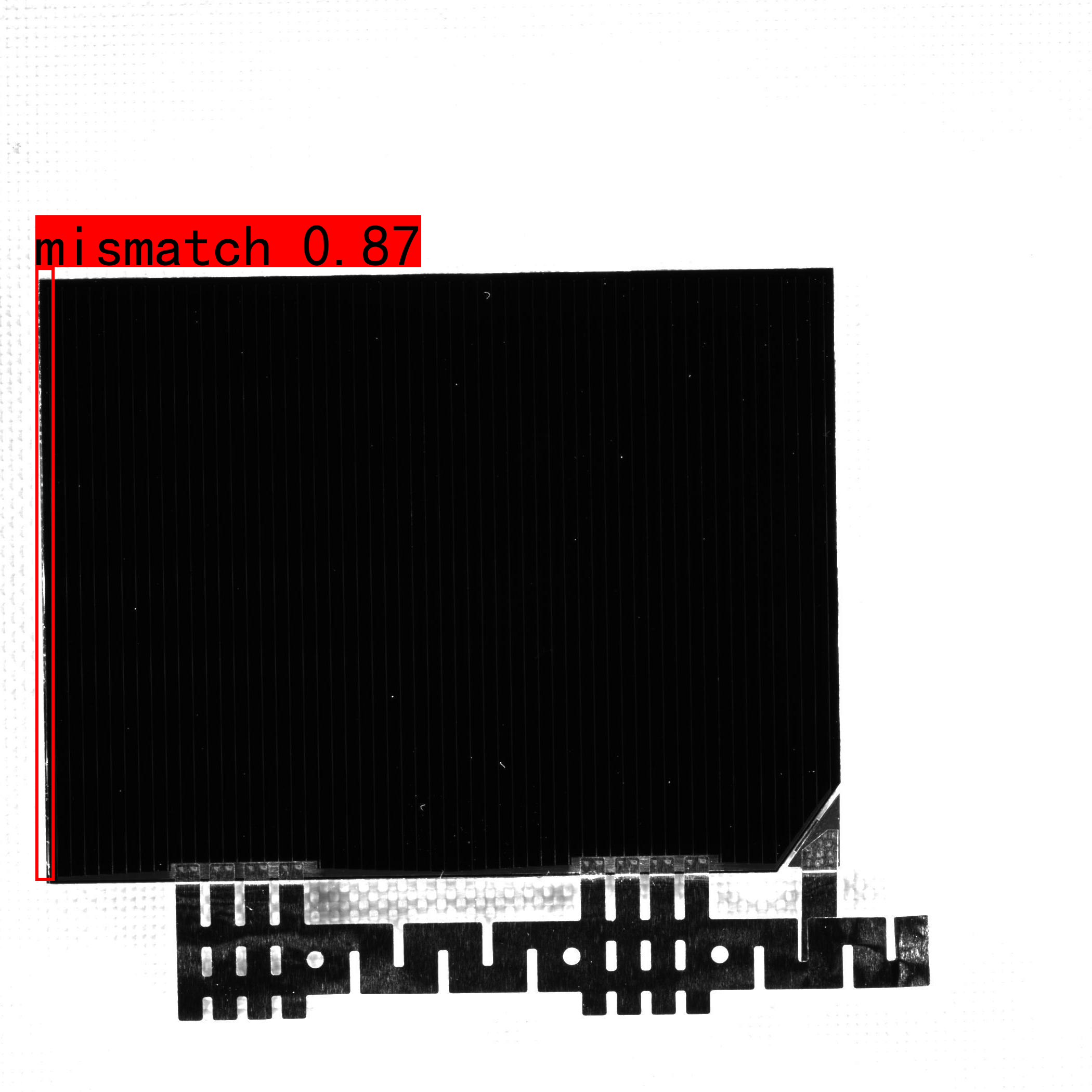

Supplement: S1 Dataset — (ZIP) [file pone.0304819.s001.zip › 00092mismatch_updown.png]

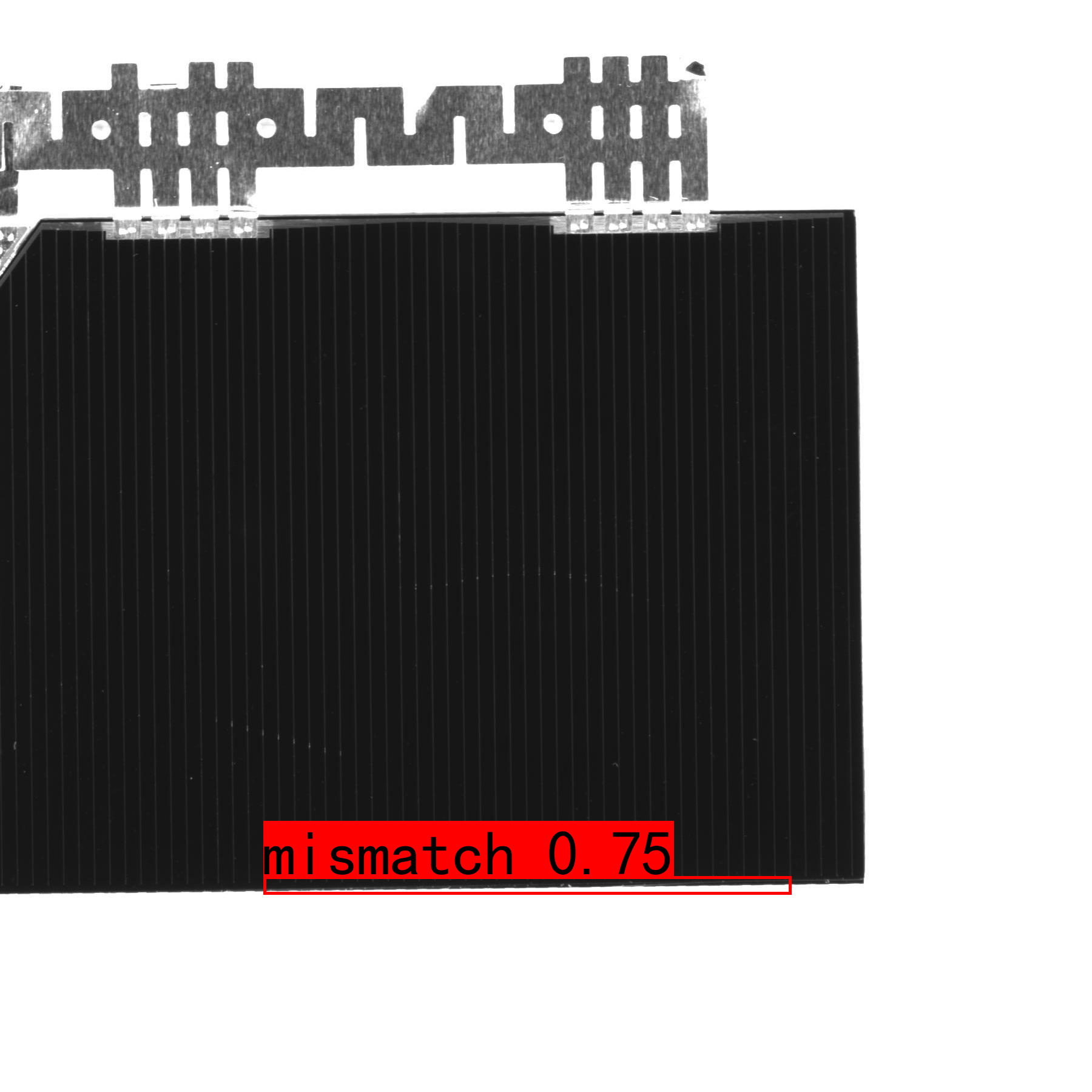

Supplement: S1 Dataset — (ZIP) [file pone.0304819.s001.zip › 00104mismatch_origin-copy_000001.png]

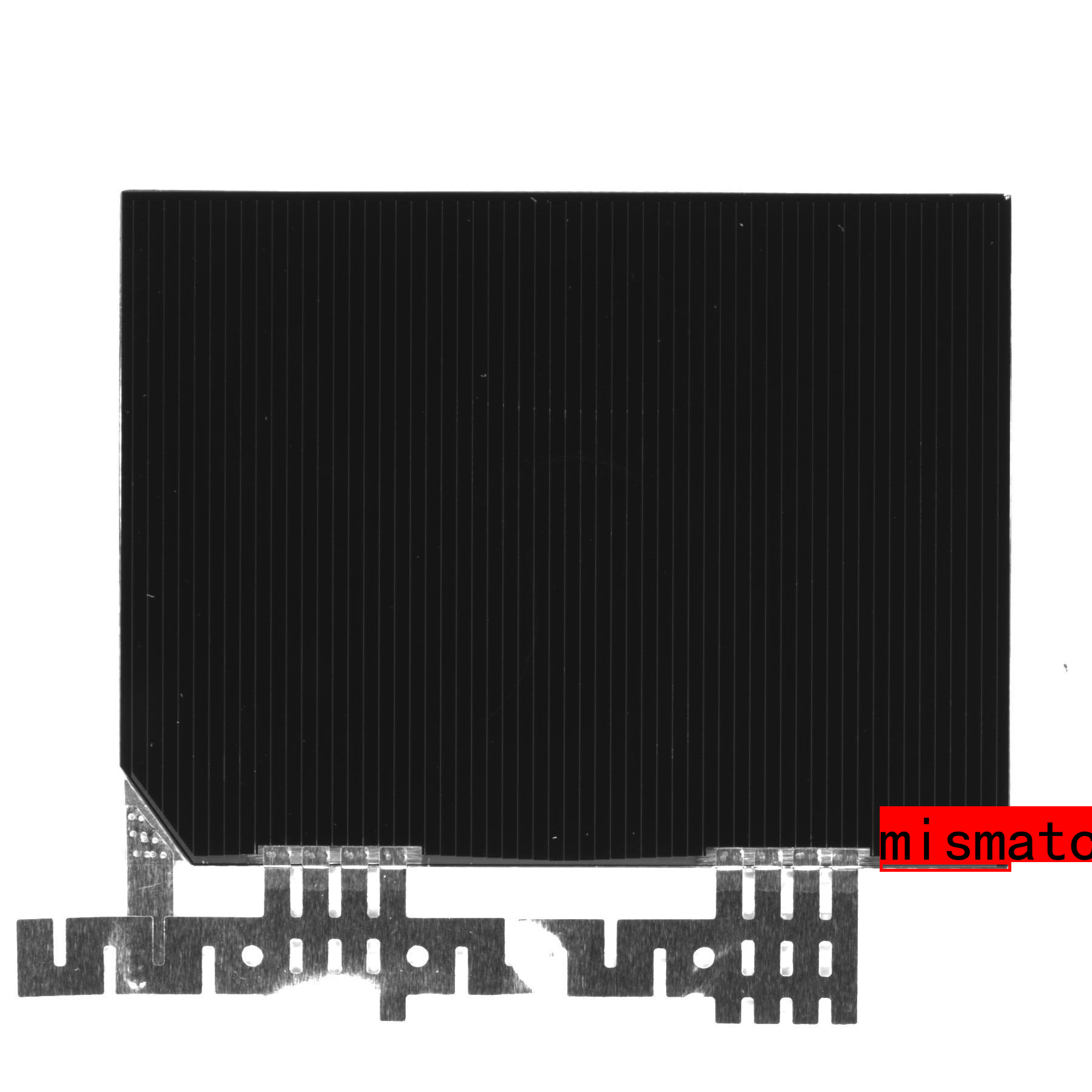

Supplement: S1 Dataset — (ZIP) [file pone.0304819.s001.zip › 00104mismatch_updown.png]

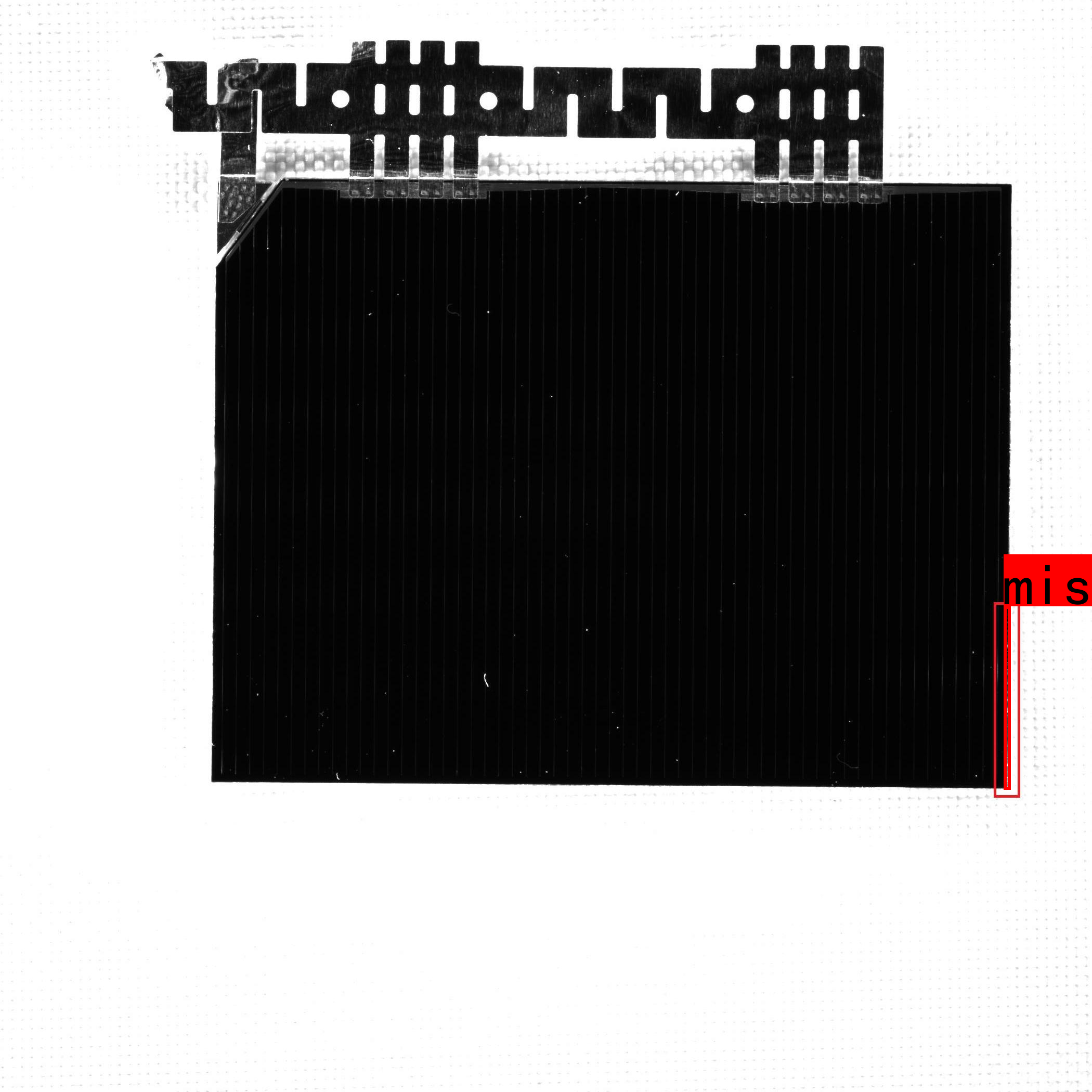

Supplement: S1 Dataset — (ZIP) [file pone.0304819.s001.zip › 00105mismatch_origin-copy_000001.png]

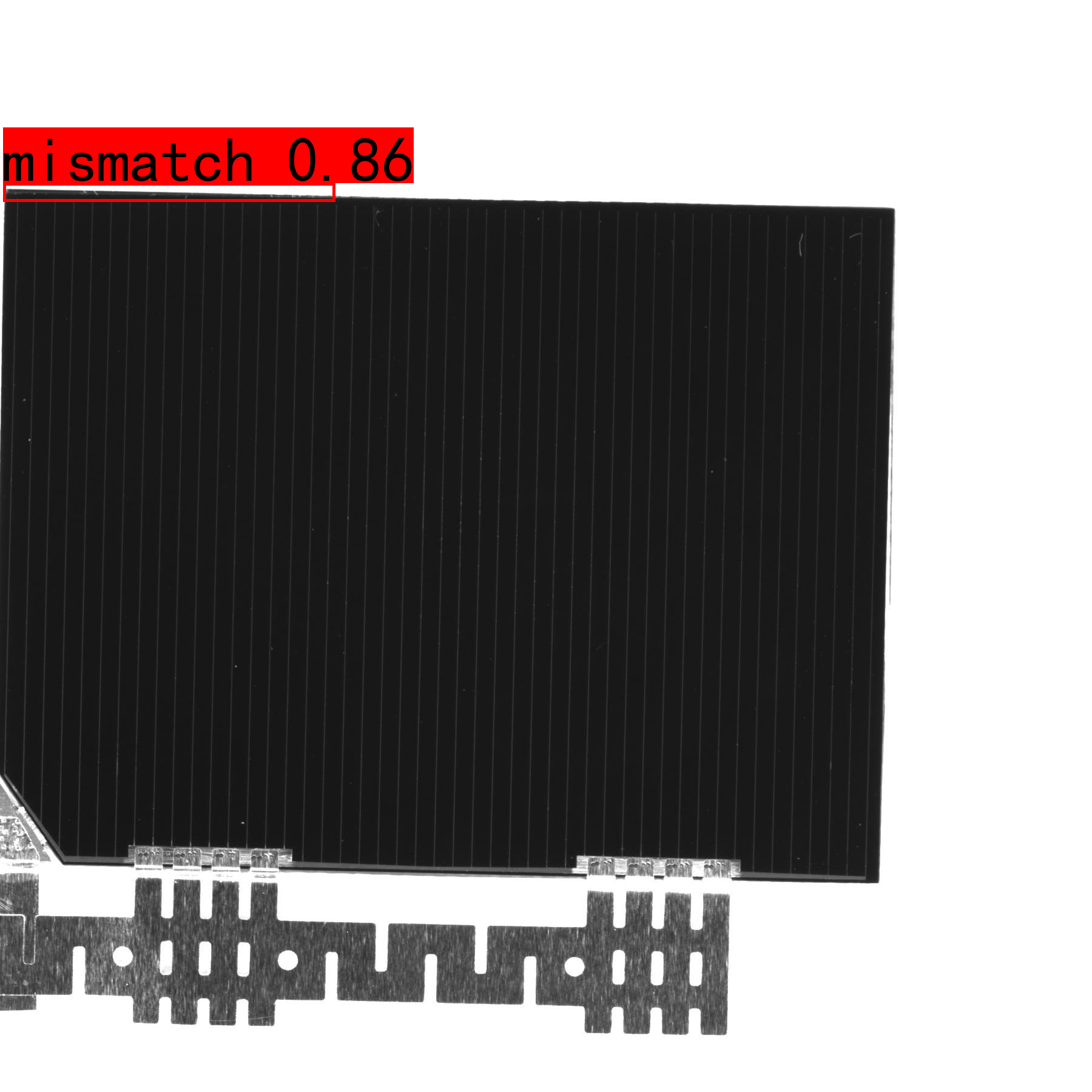

Supplement: S1 Dataset — (ZIP) [file pone.0304819.s001.zip › 00115mismatch_updown.png]

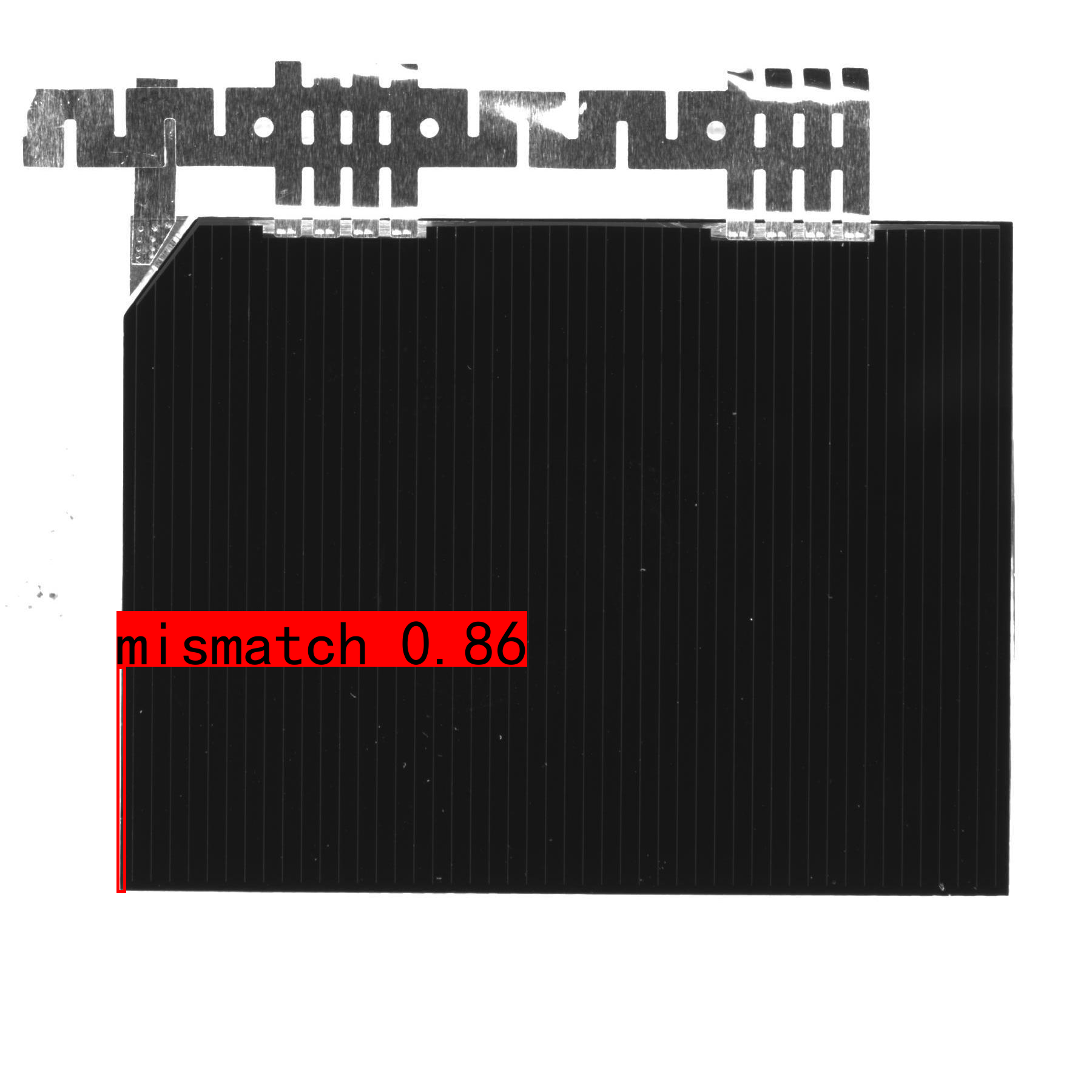

Supplement: S1 Dataset — (ZIP) [file pone.0304819.s001.zip › 00116mismatch_origin-copy_000001.png]

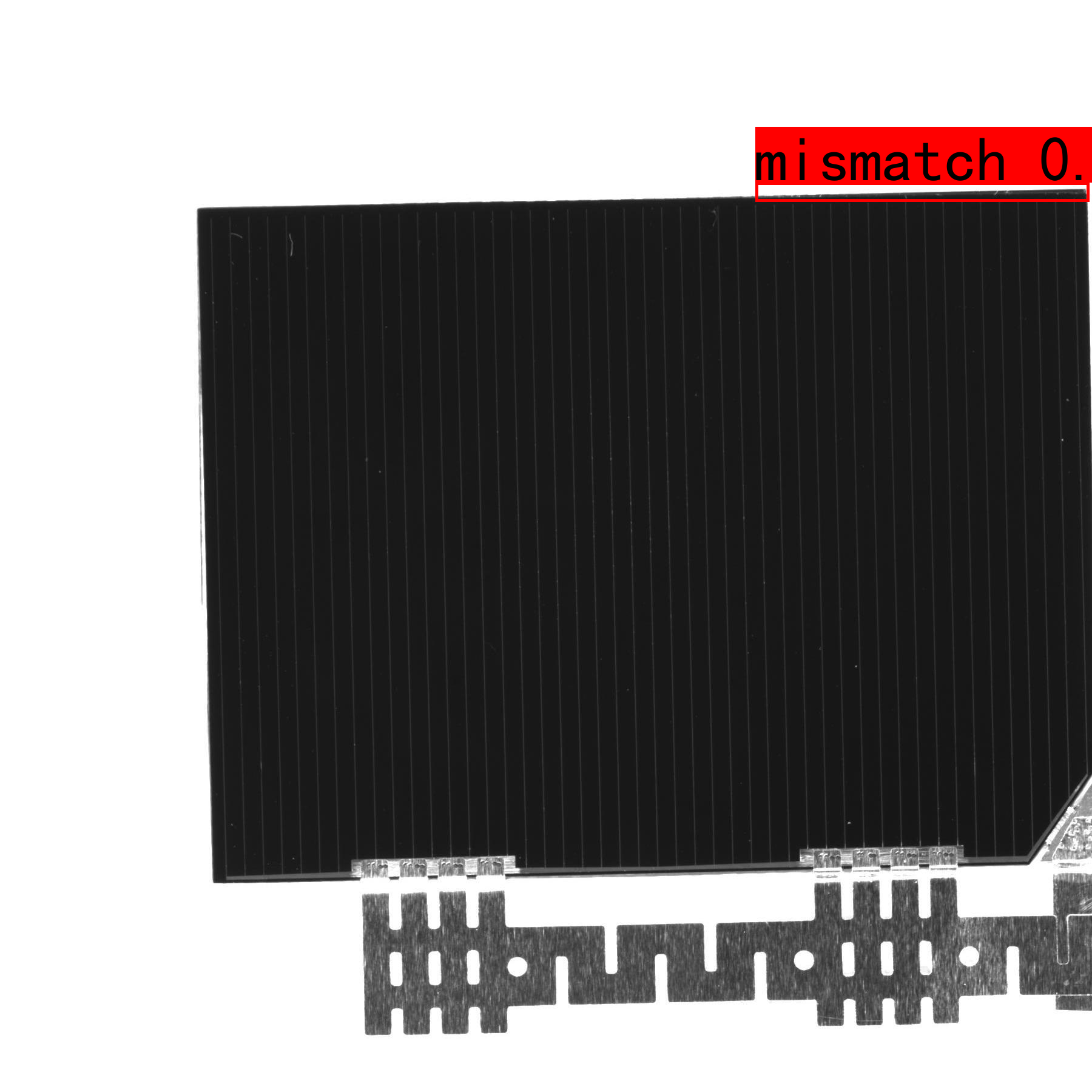

Supplement: S1 Dataset — (ZIP) [file pone.0304819.s001.zip › 00116mismatch_updown.png]

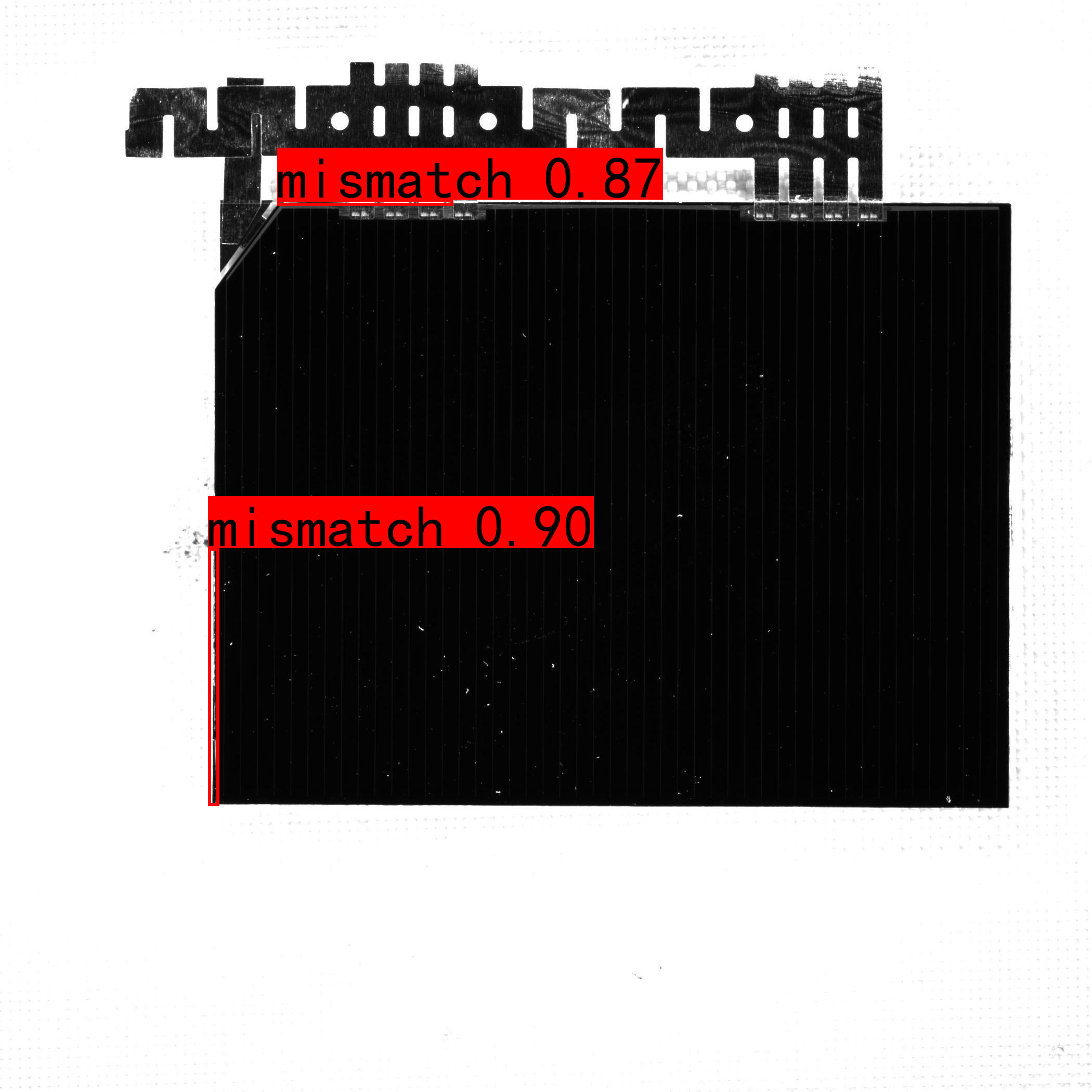

Supplement: S1 Dataset — (ZIP) [file pone.0304819.s001.zip › 00128mismatch_origin-copy_000001.png]

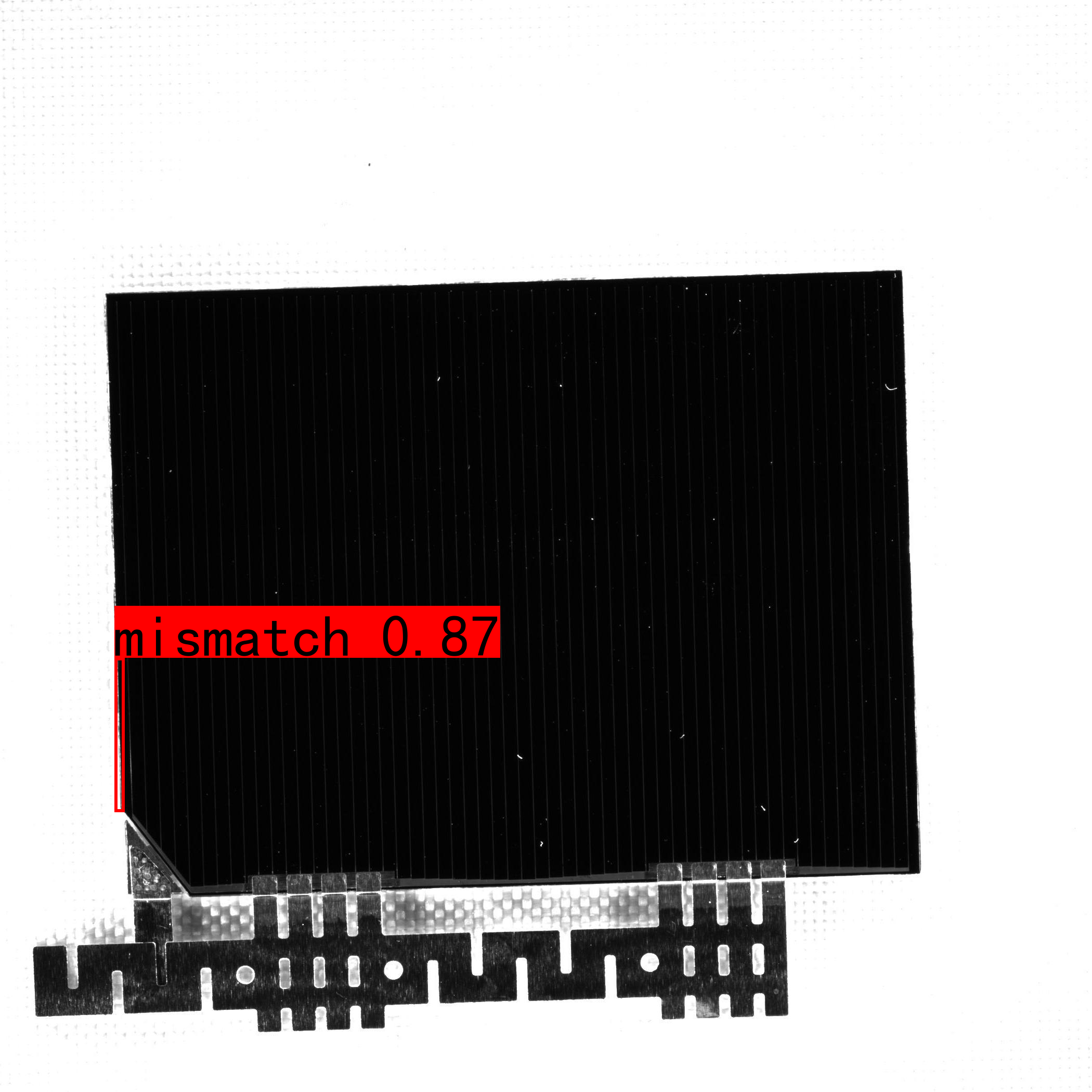

Supplement: S1 Dataset — (ZIP) [file pone.0304819.s001.zip › 00128mismatch_updown.png]

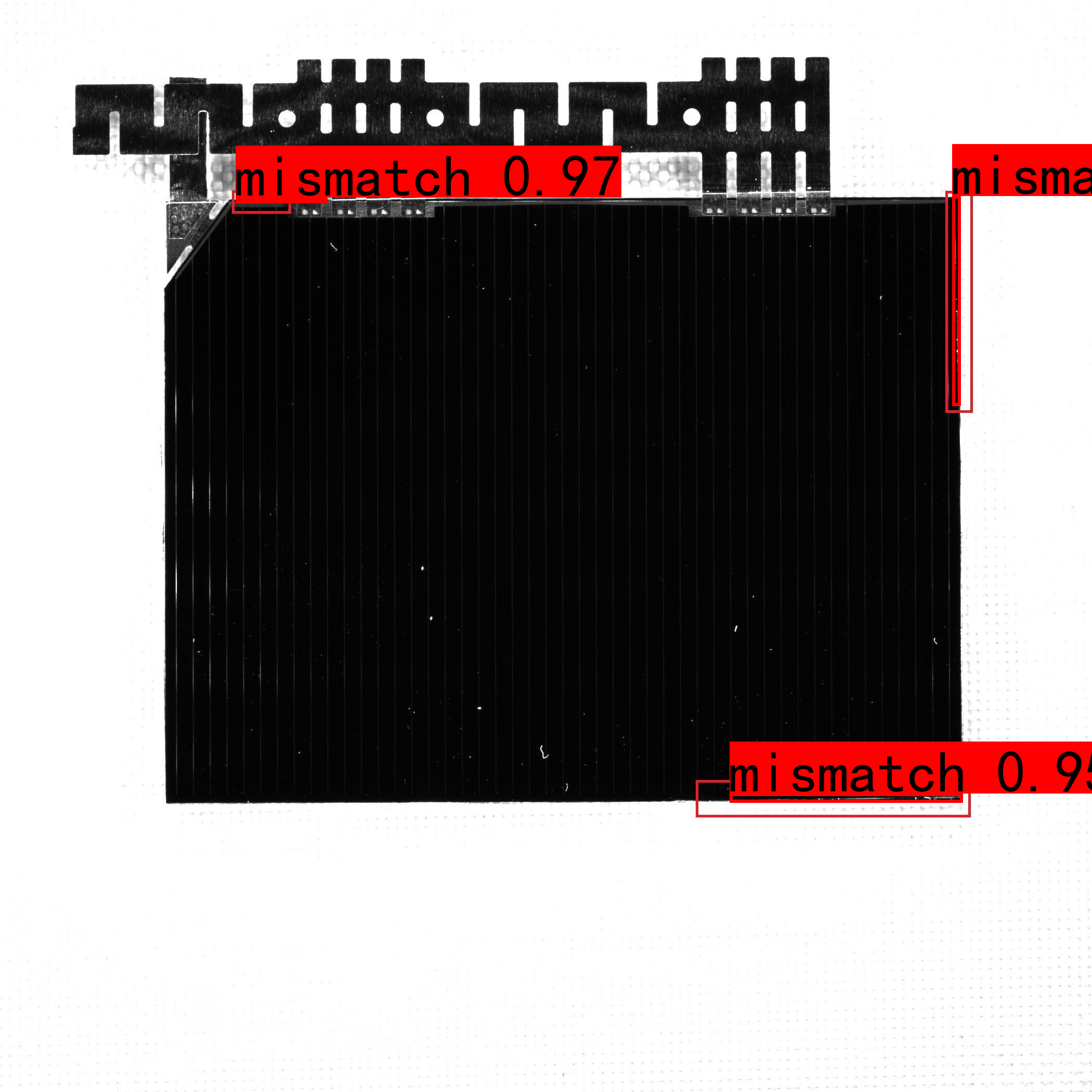

Supplement: S1 Dataset — (ZIP) [file pone.0304819.s001.zip › 00129mismatch_origin-copy_000001.png]

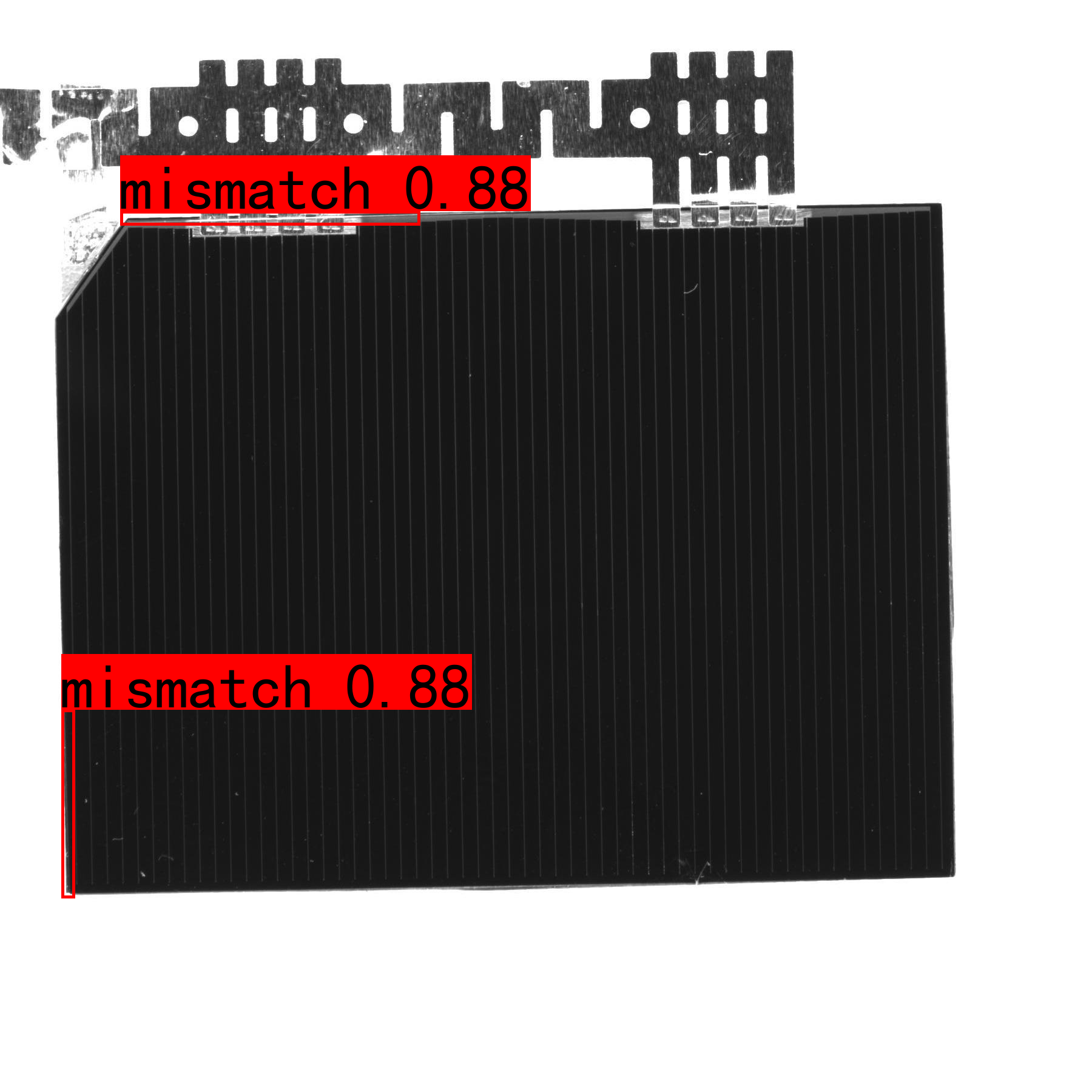

Supplement: S1 Dataset — (ZIP) [file pone.0304819.s001.zip › 00140mismatch_updown.png]

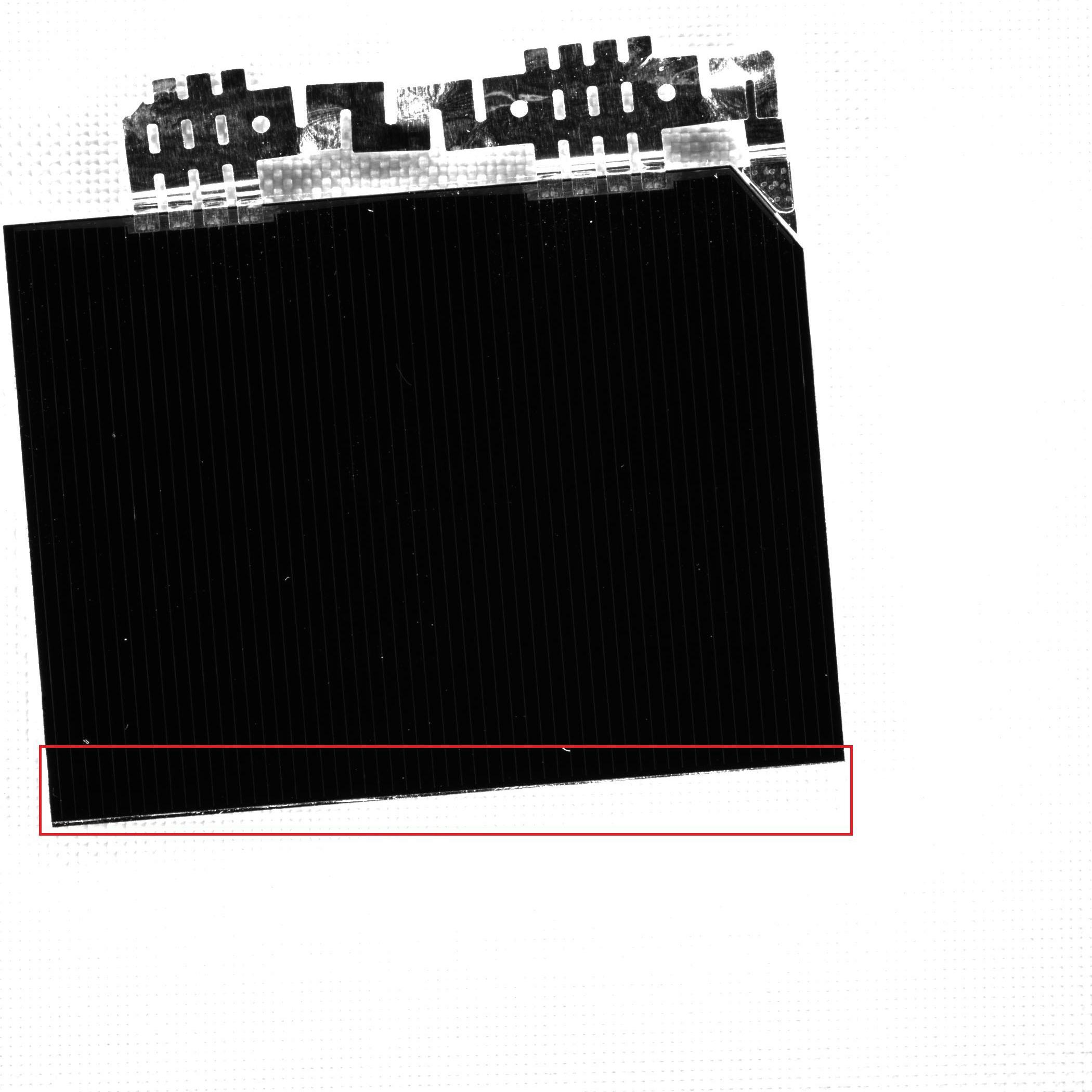

Supplement: S1 Dataset — (ZIP) [file pone.0304819.s001.zip › 00141mismatch_origin-copy_000001.png]

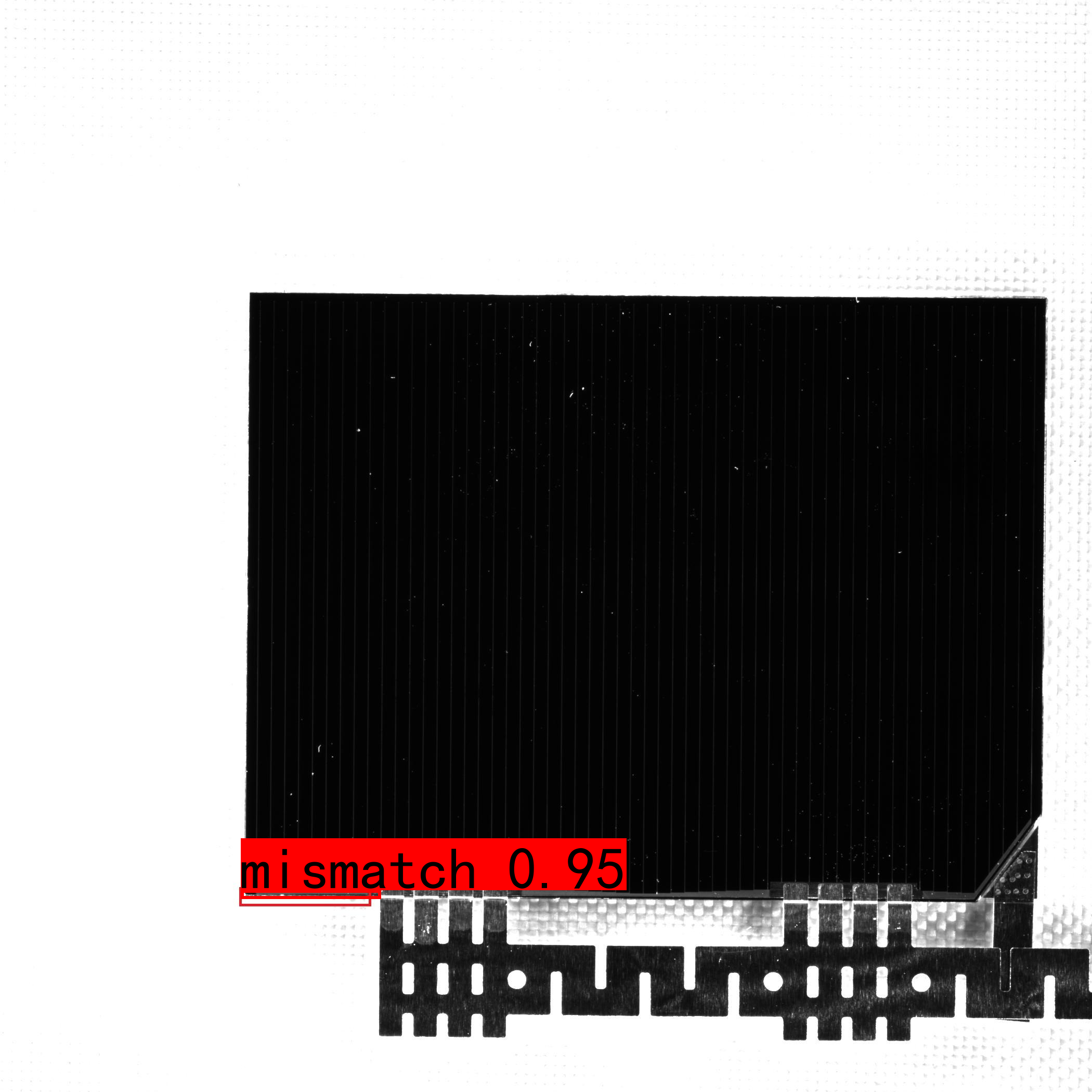

Supplement: S1 Dataset — (ZIP) [file pone.0304819.s001.zip › 00141mismatch_updown.png]

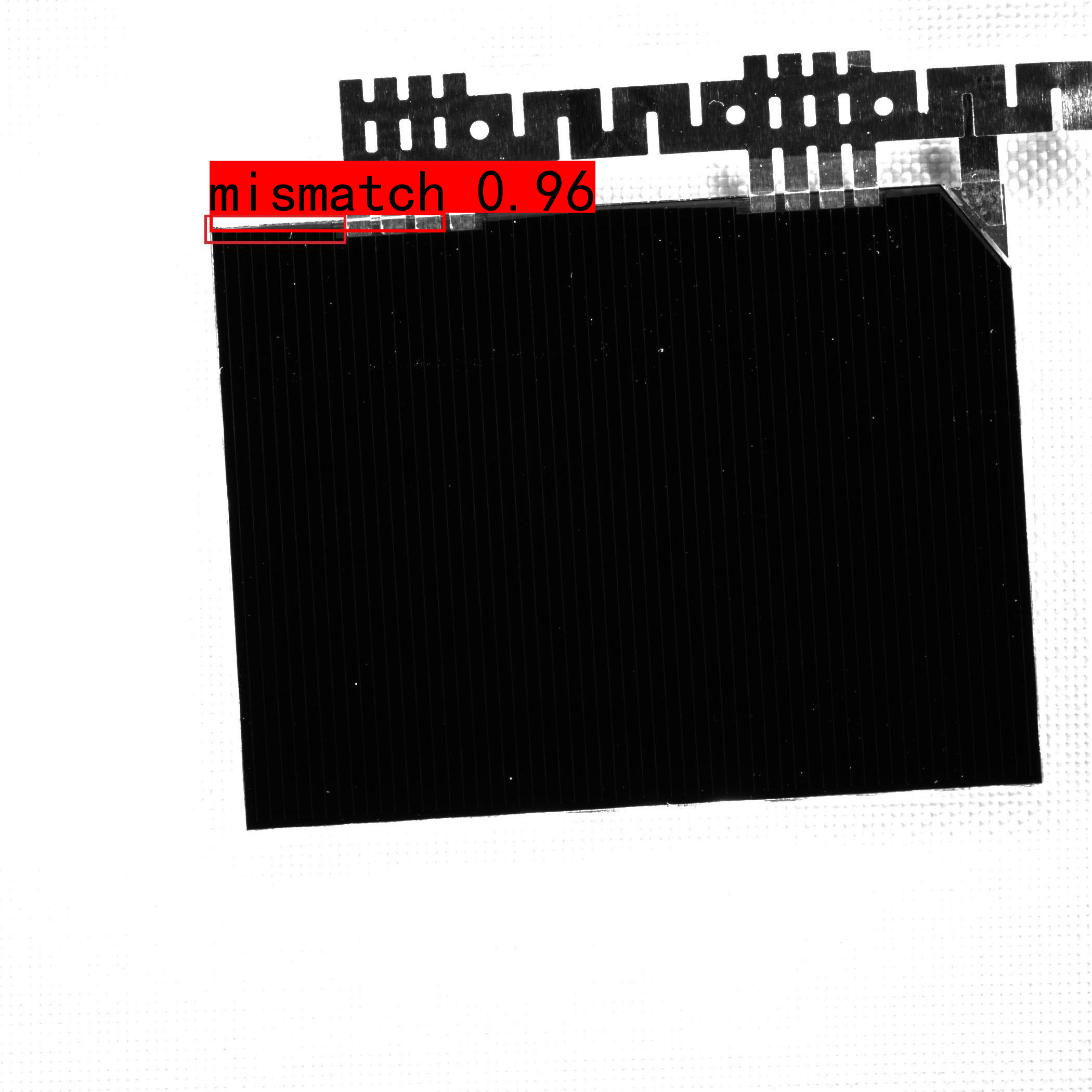

Supplement: S1 Dataset — (ZIP) [file pone.0304819.s001.zip › 00153mismatch_origin-copy_000001.png]

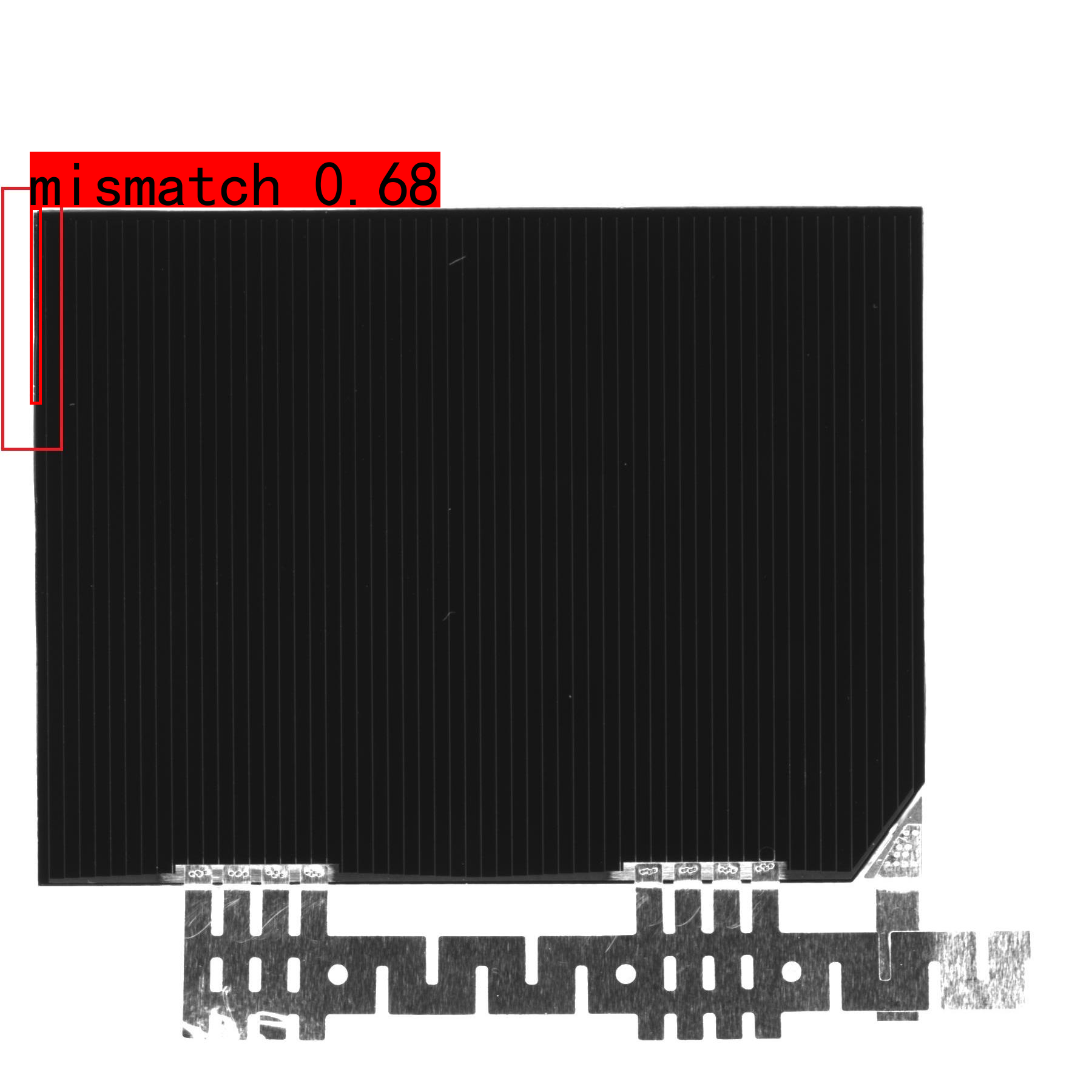

Supplement: S1 Dataset — (ZIP) [file pone.0304819.s001.zip › 00153mismatch_updown.png]

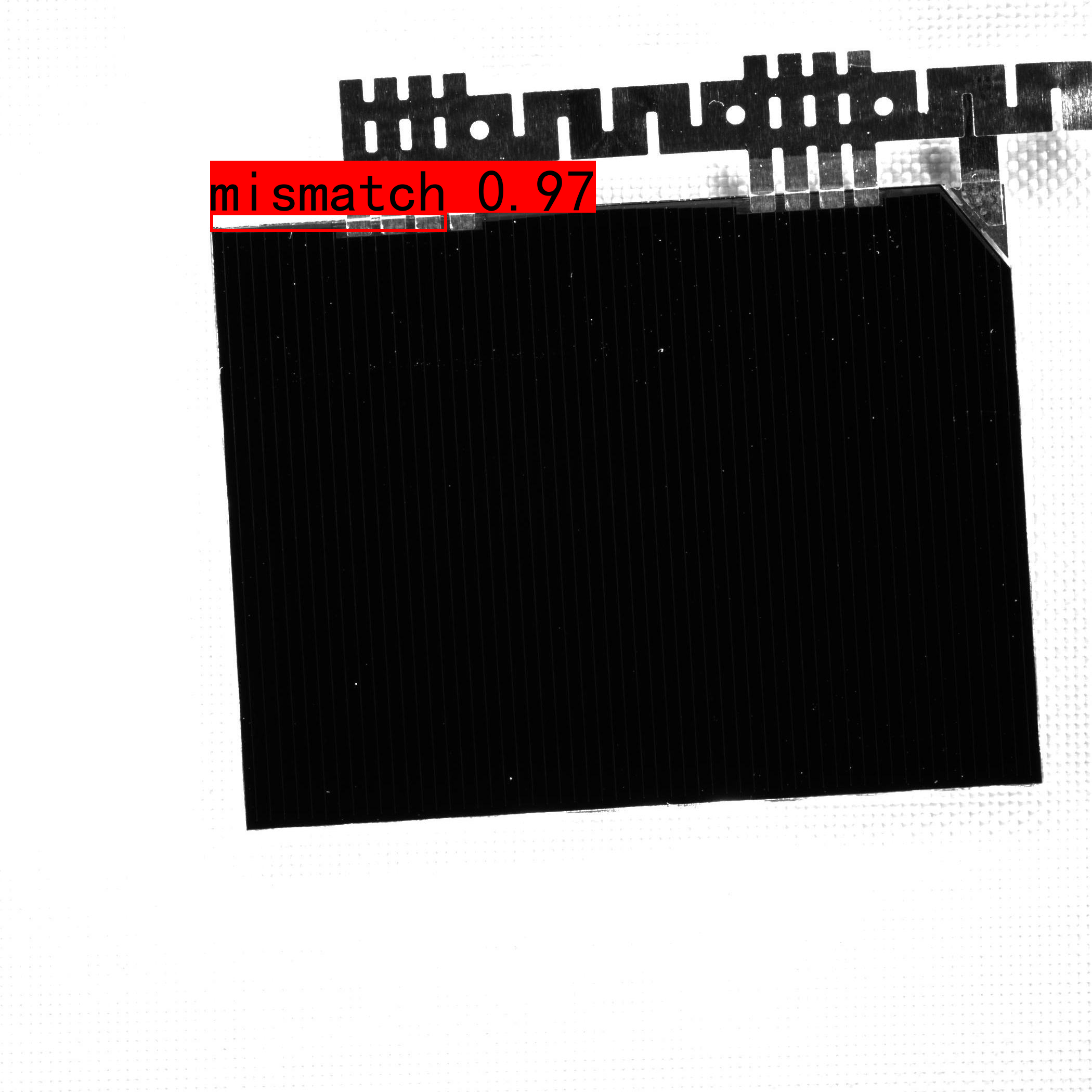

Supplement: S1 Dataset — (ZIP) [file pone.0304819.s001.zip › 00154mismatch_origin-copy_000001.png]

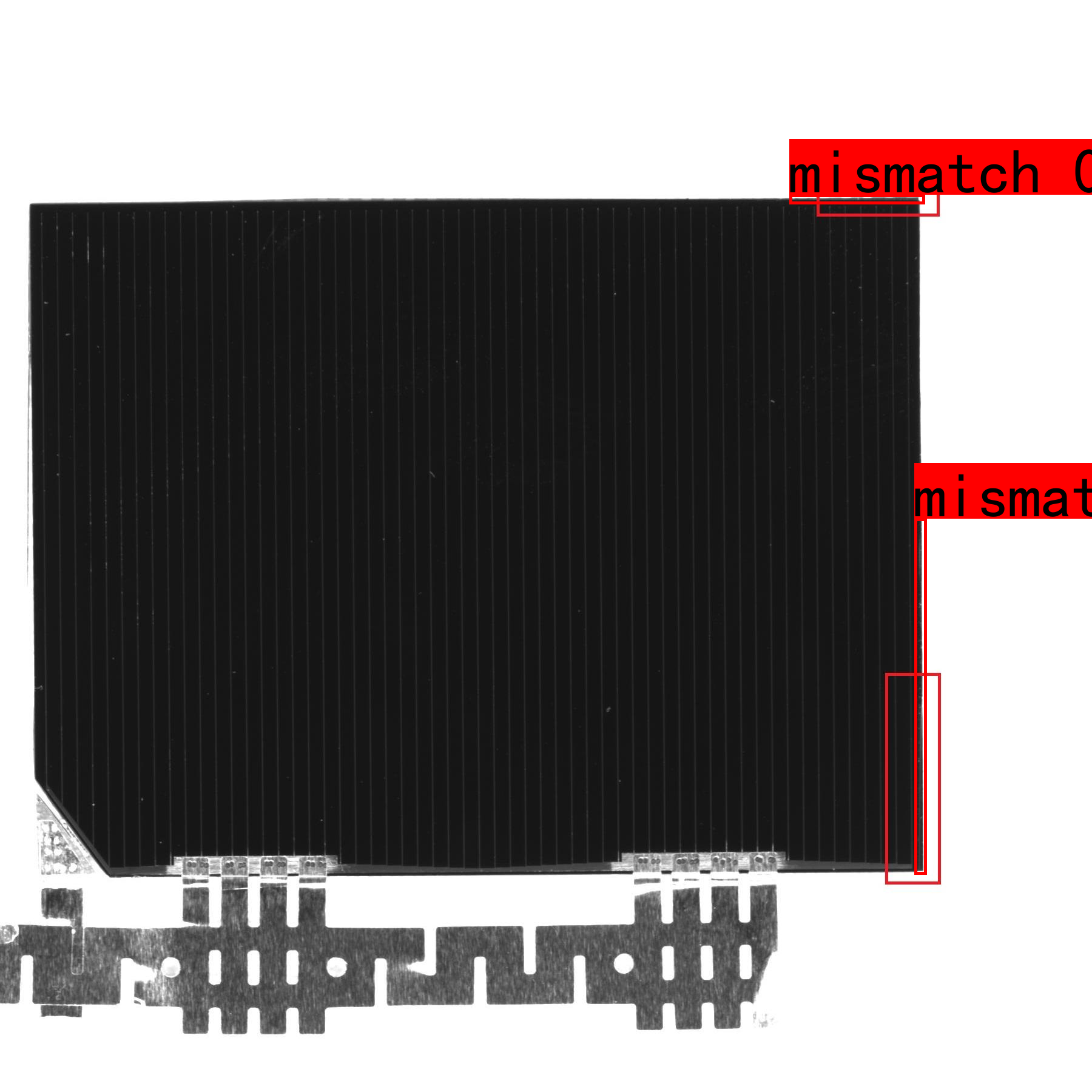

Supplement: S1 Dataset — (ZIP) [file pone.0304819.s001.zip › 00165mismatch_updown.png]

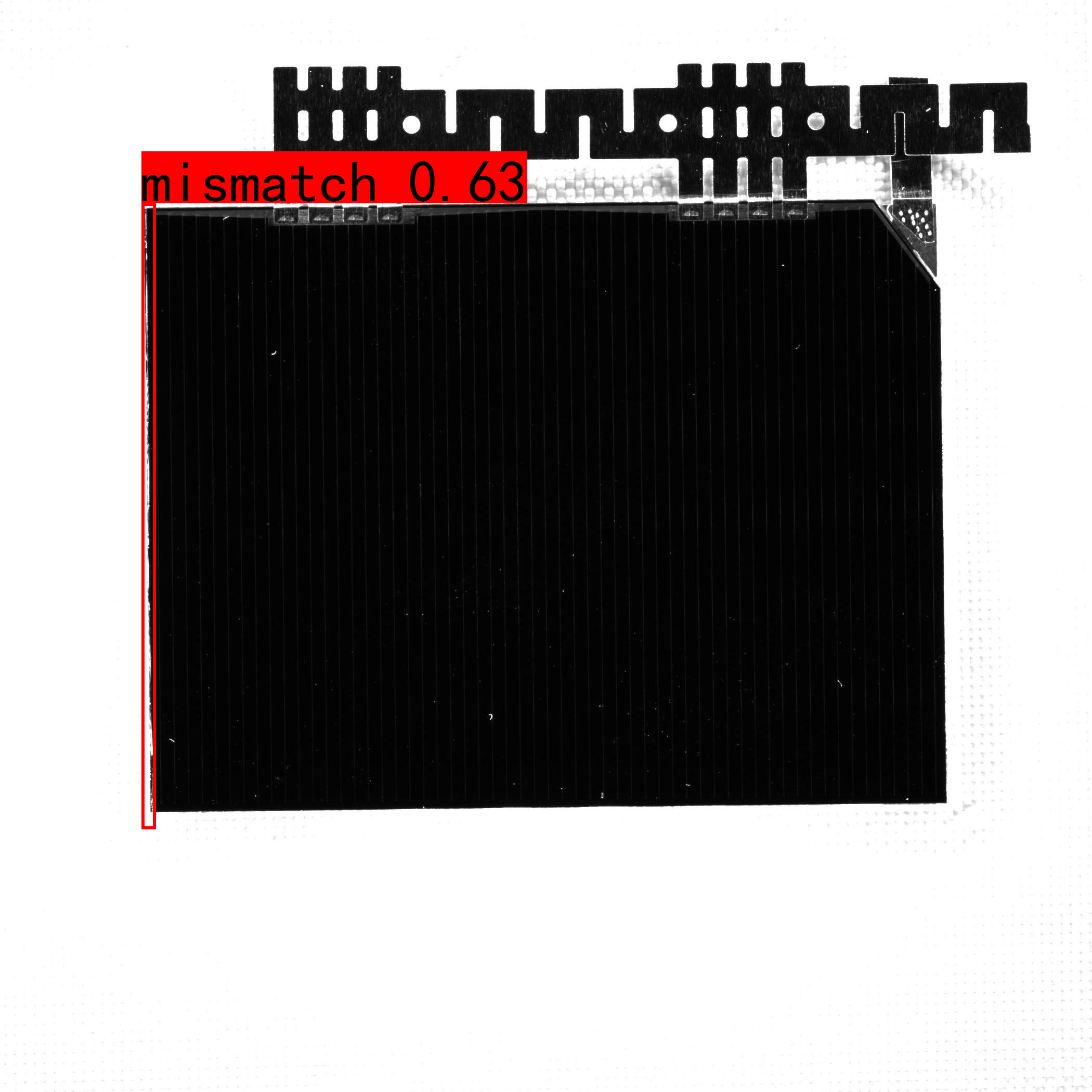

Supplement: S1 Dataset — (ZIP) [file pone.0304819.s001.zip › 00166mismatch_origin-copy_000001.png]

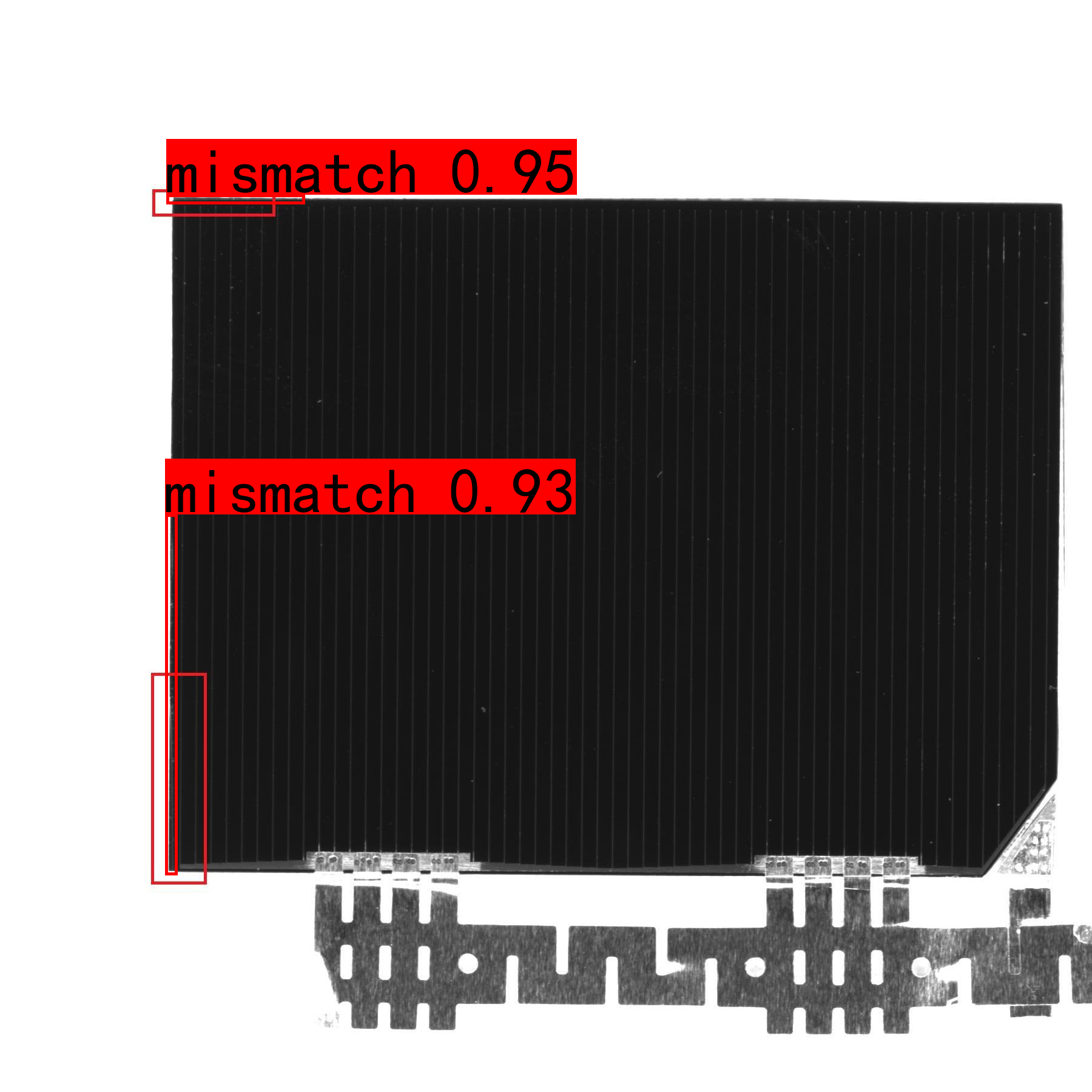

Supplement: S1 Dataset — (ZIP) [file pone.0304819.s001.zip › 00166mismatch_updown.png]

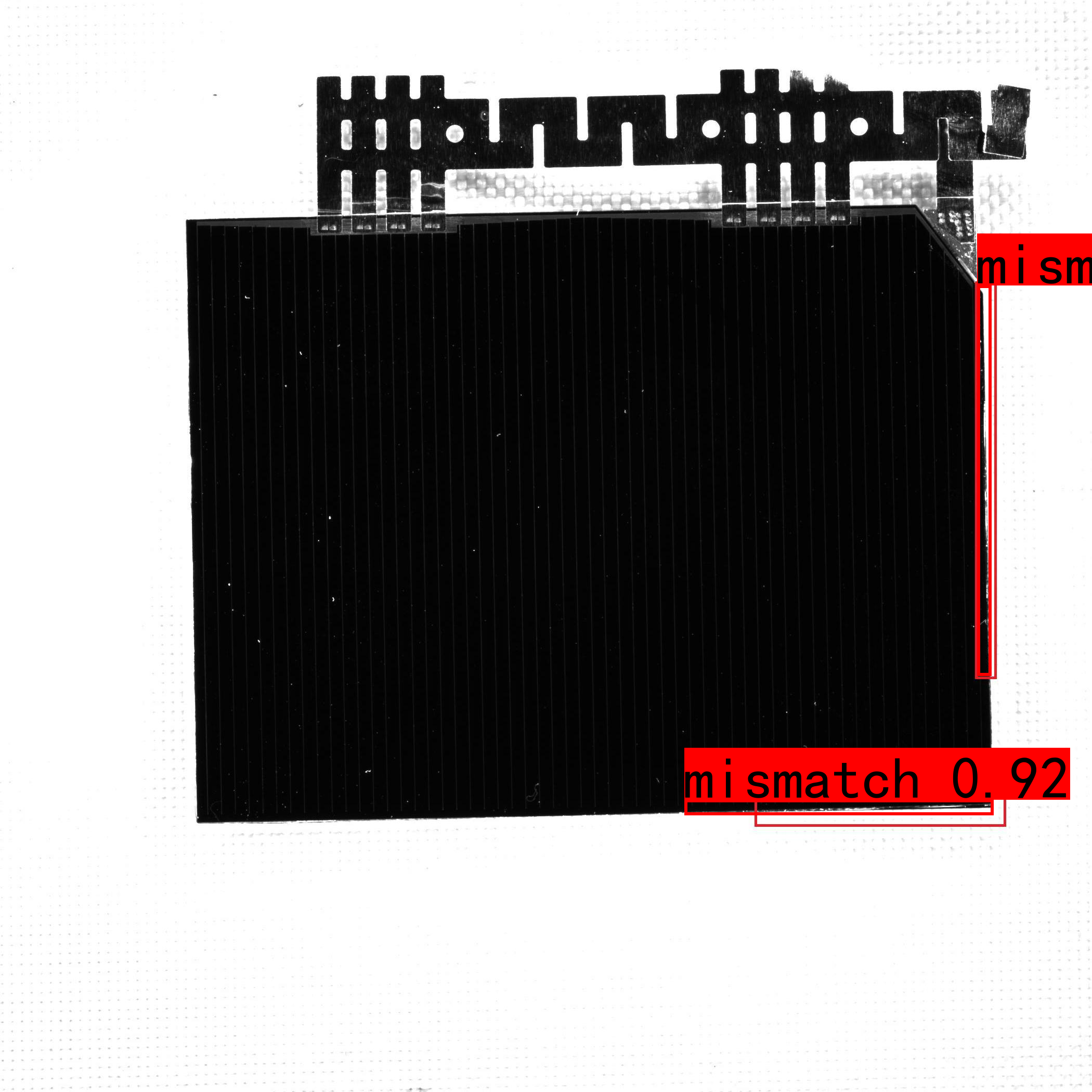

Supplement: S1 Dataset — (ZIP) [file pone.0304819.s001.zip › 00179mismatch_origin-copy_000001.png]

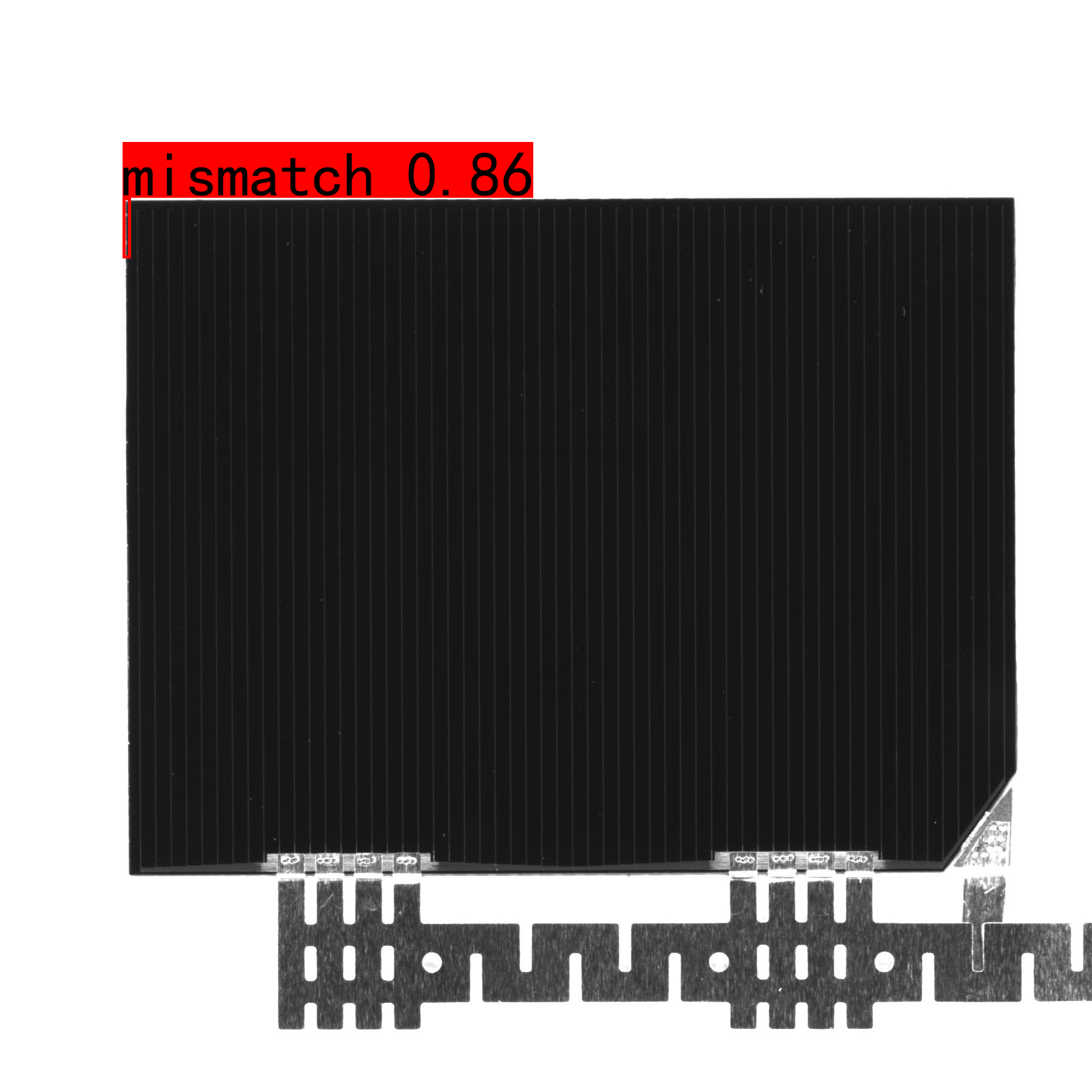

Supplement: S1 Dataset — (ZIP) [file pone.0304819.s001.zip › 00179mismatch_updown.png]

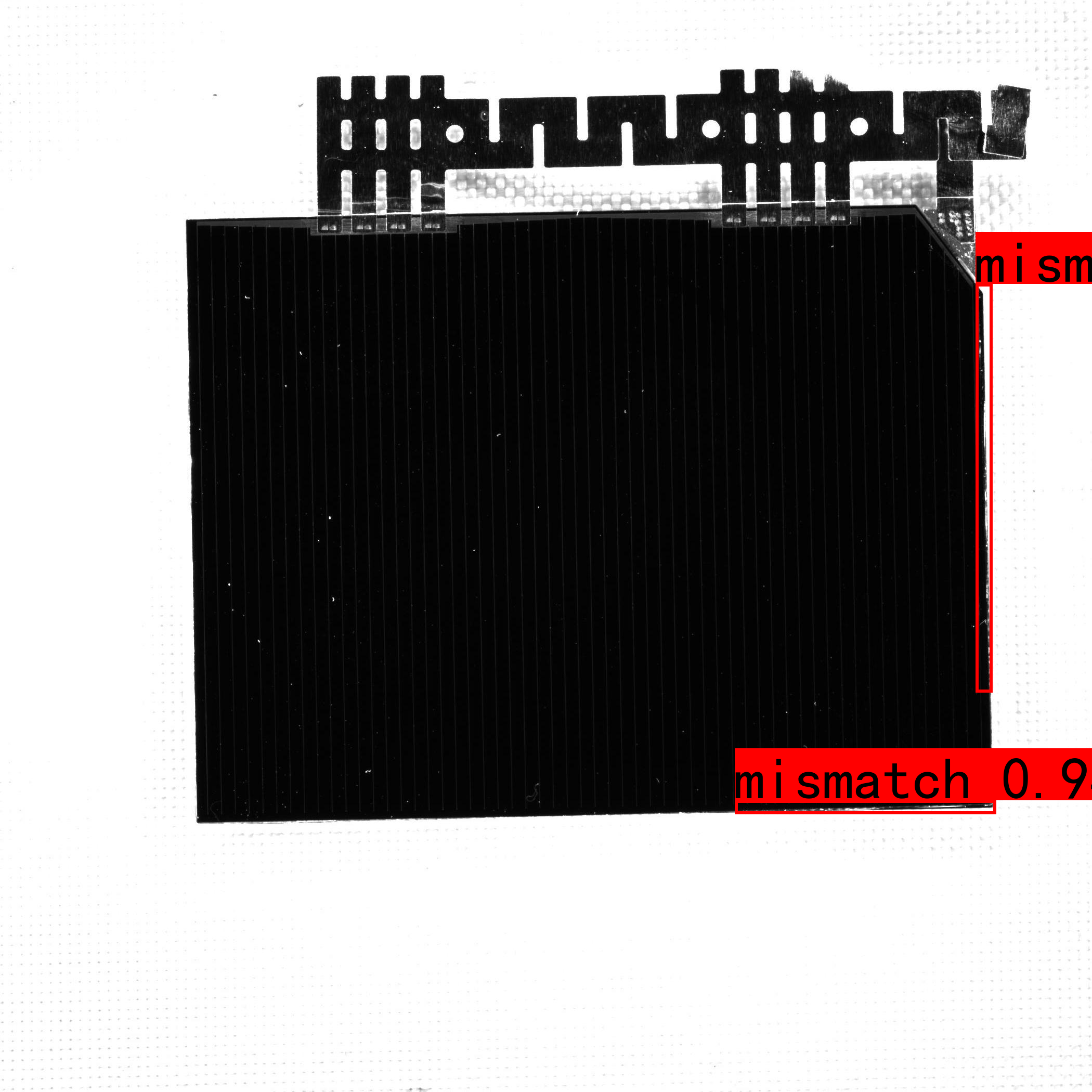

Supplement: S1 Dataset — (ZIP) [file pone.0304819.s001.zip › 00180mismatch_origin-copy_000001.png]

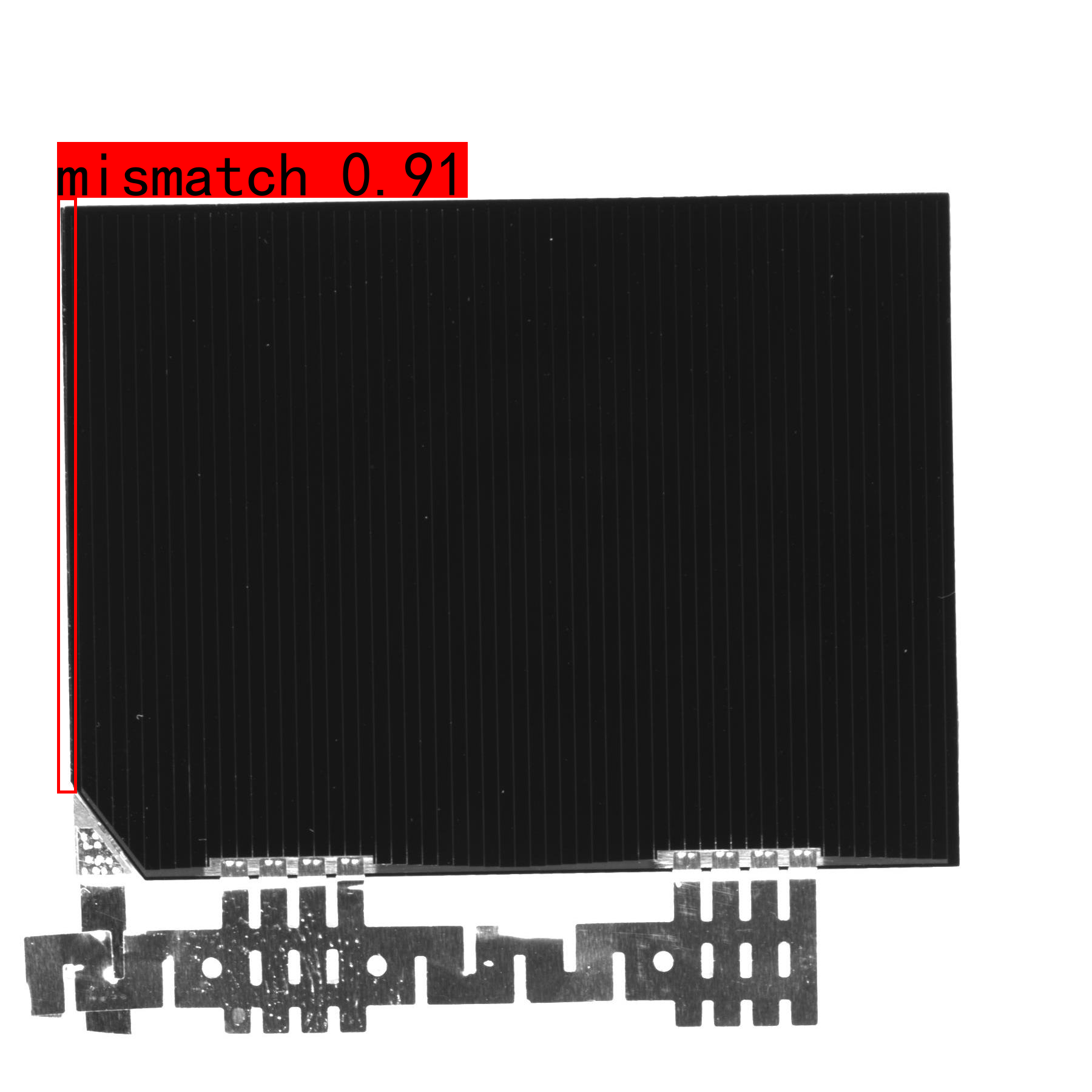

Supplement: S1 Dataset — (ZIP) [file pone.0304819.s001.zip › 00191mismatch_updown.png]

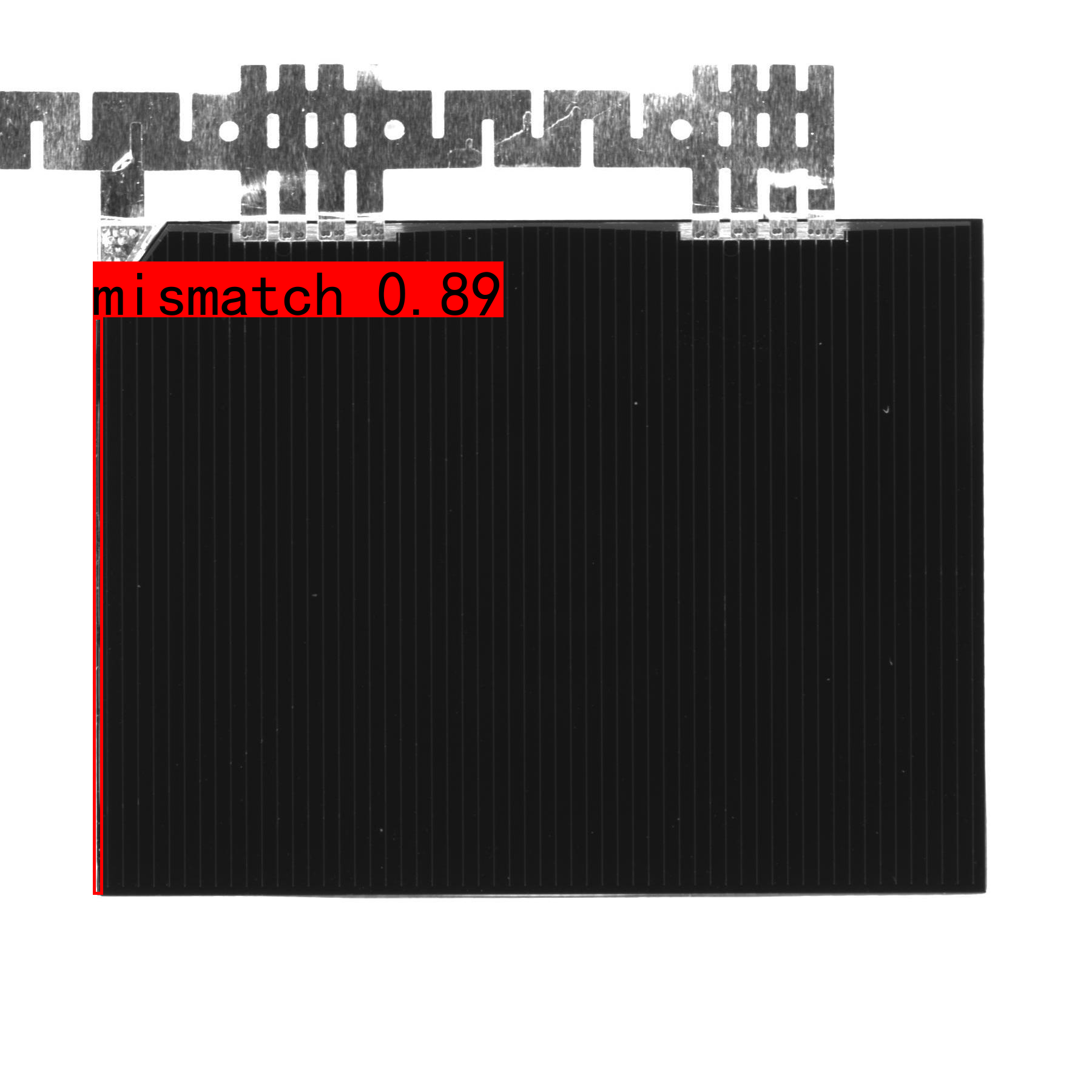

Supplement: S1 Dataset — (ZIP) [file pone.0304819.s001.zip › 00192mismatch_origin-copy_000001.png]

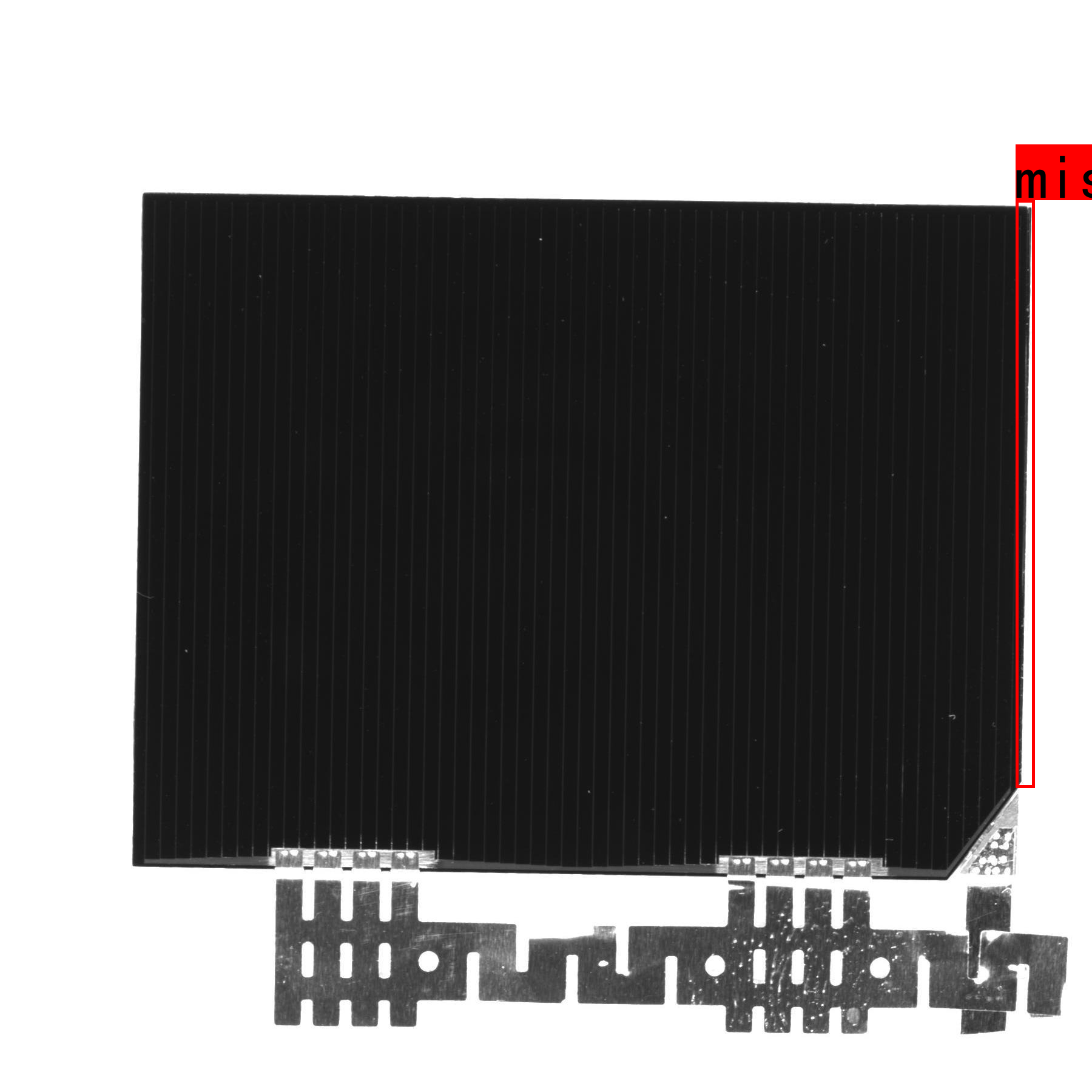

Supplement: S1 Dataset — (ZIP) [file pone.0304819.s001.zip › 00192mismatch_updown.png]

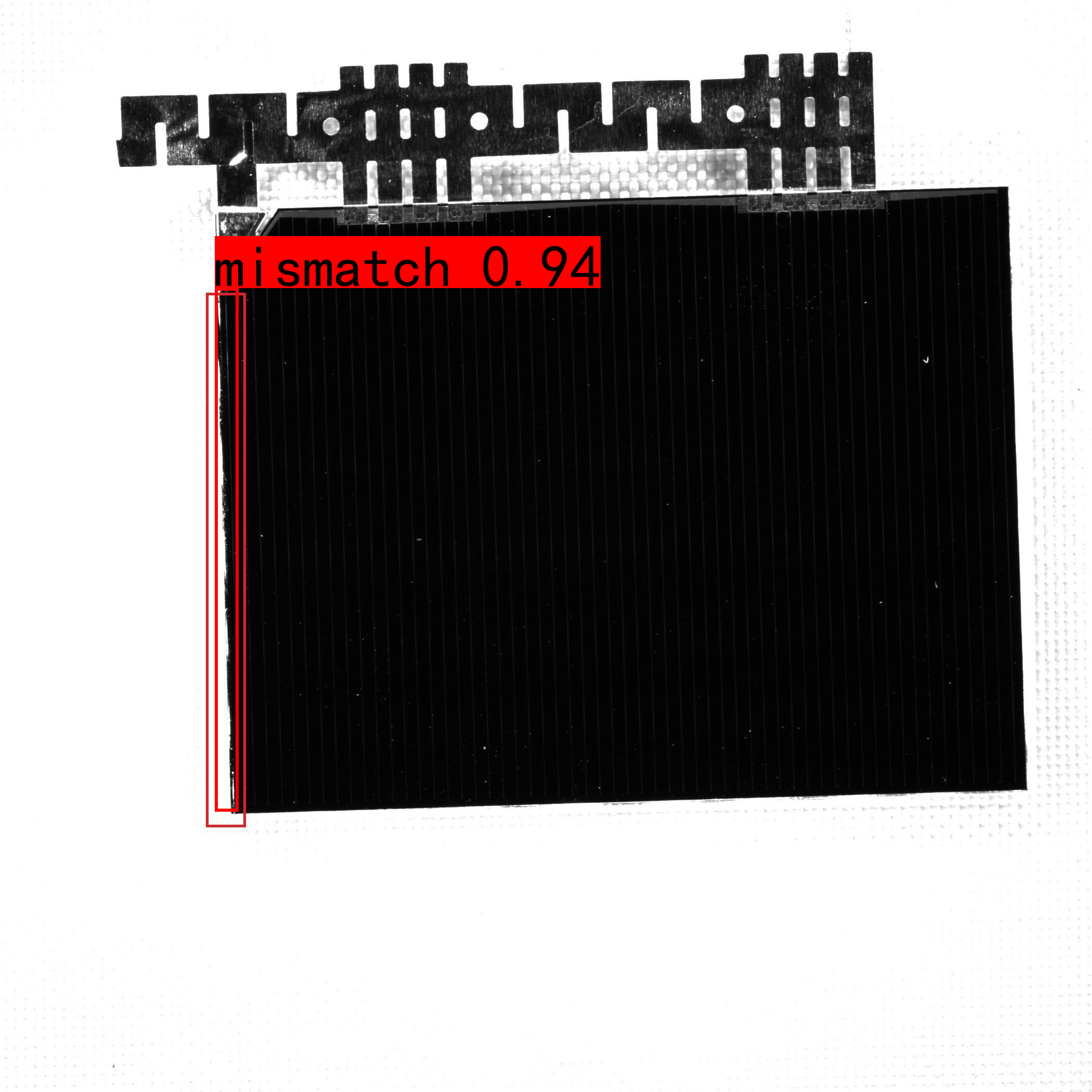

Supplement: S1 Dataset — (ZIP) [file pone.0304819.s001.zip › 00203mismatch_origin-copy_000001.png]

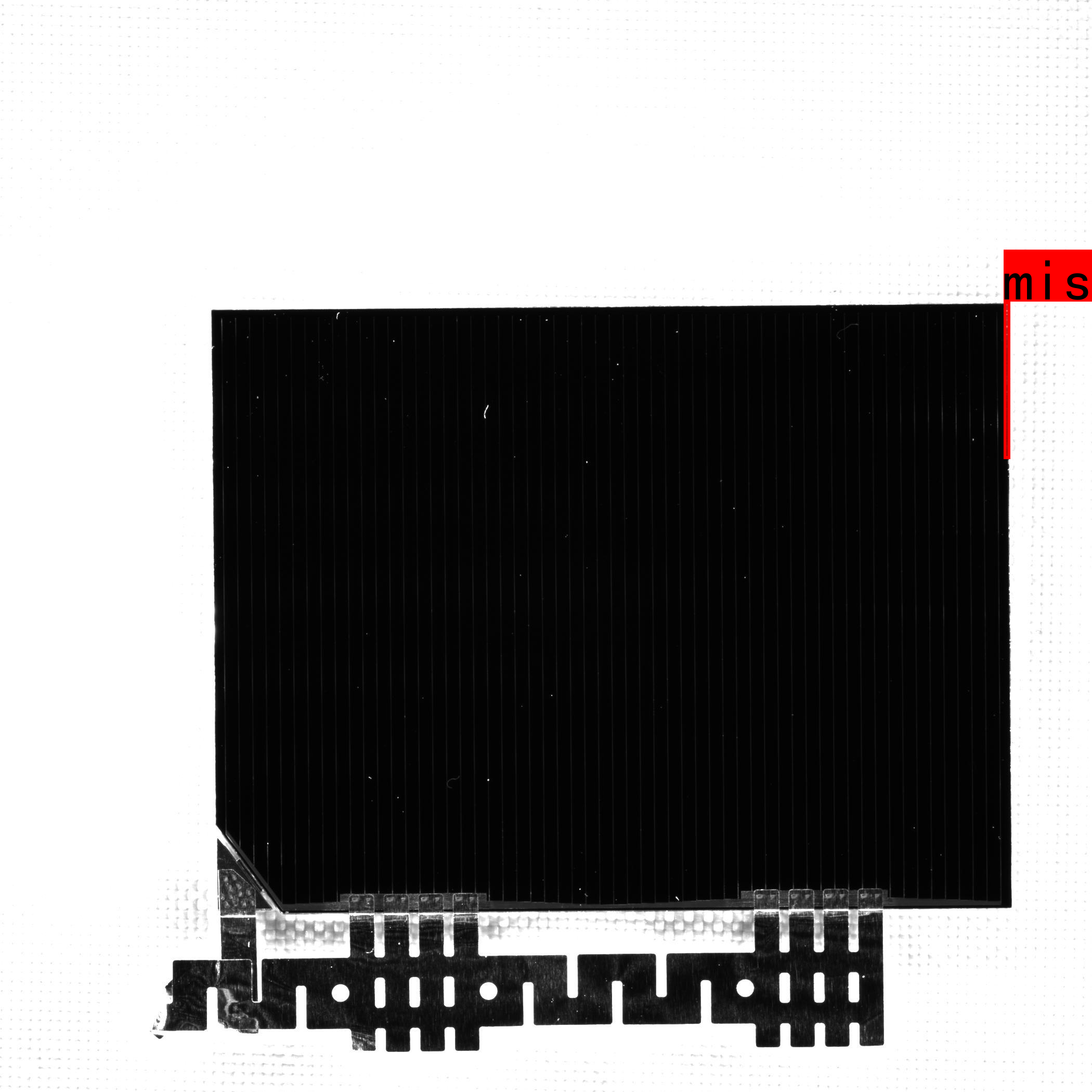

Supplement: S1 Dataset — (ZIP) [file pone.0304819.s001.zip › 00203mismatch_updown.png]

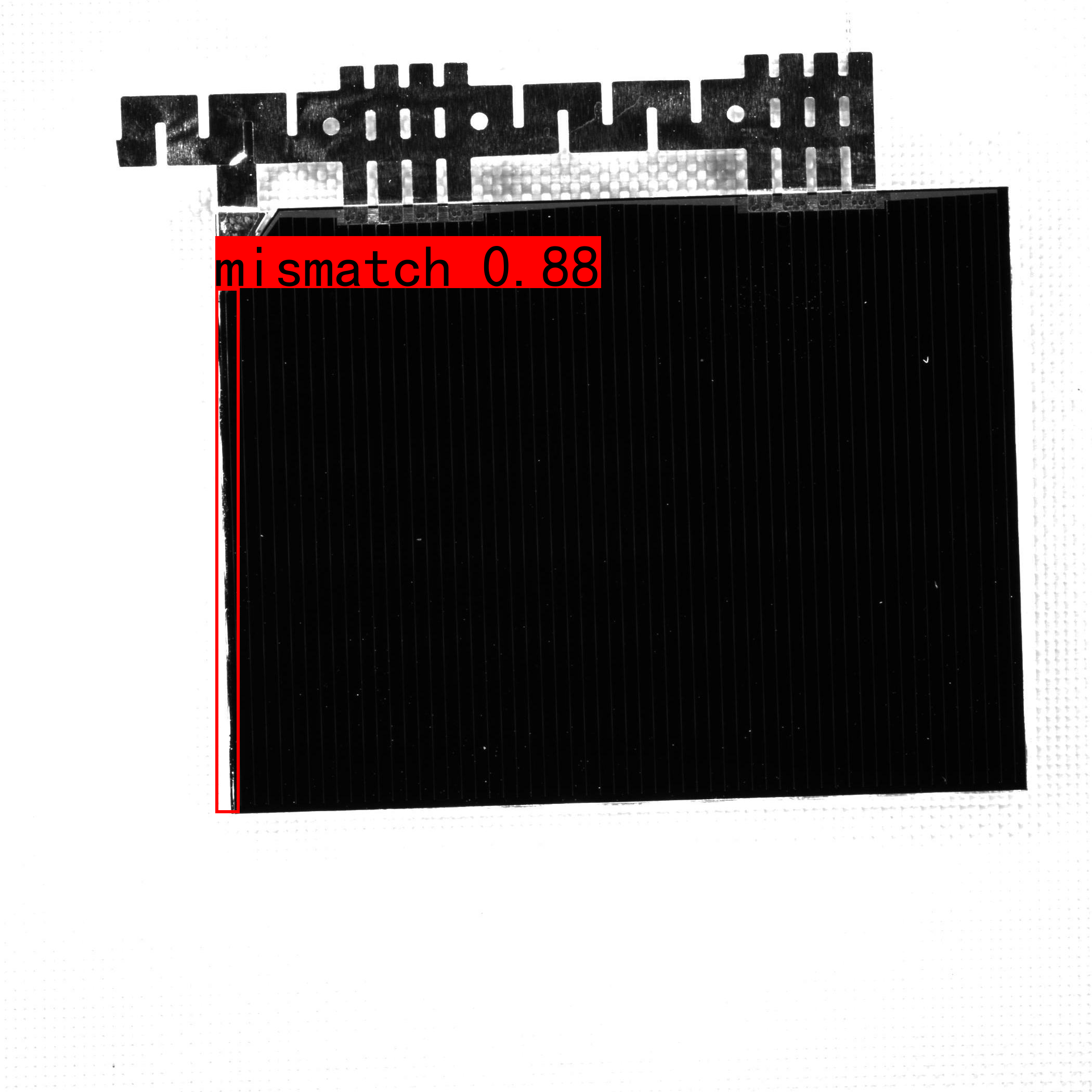

Supplement: S1 Dataset — (ZIP) [file pone.0304819.s001.zip › 00204mismatch_origin-copy_000001.png]

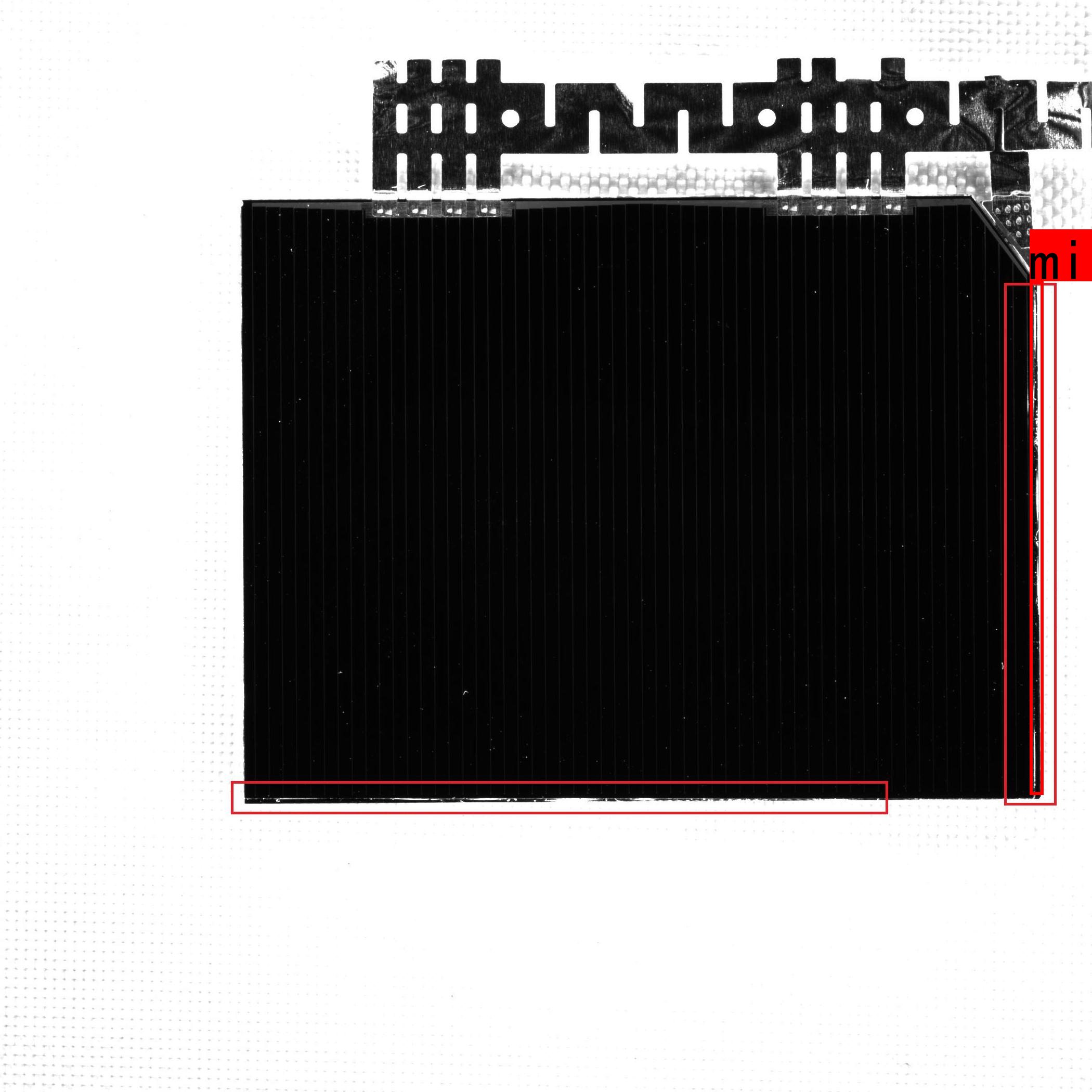

Supplement: S1 Dataset — (ZIP) [file pone.0304819.s001.zip › 00214mismatch_updown.png]

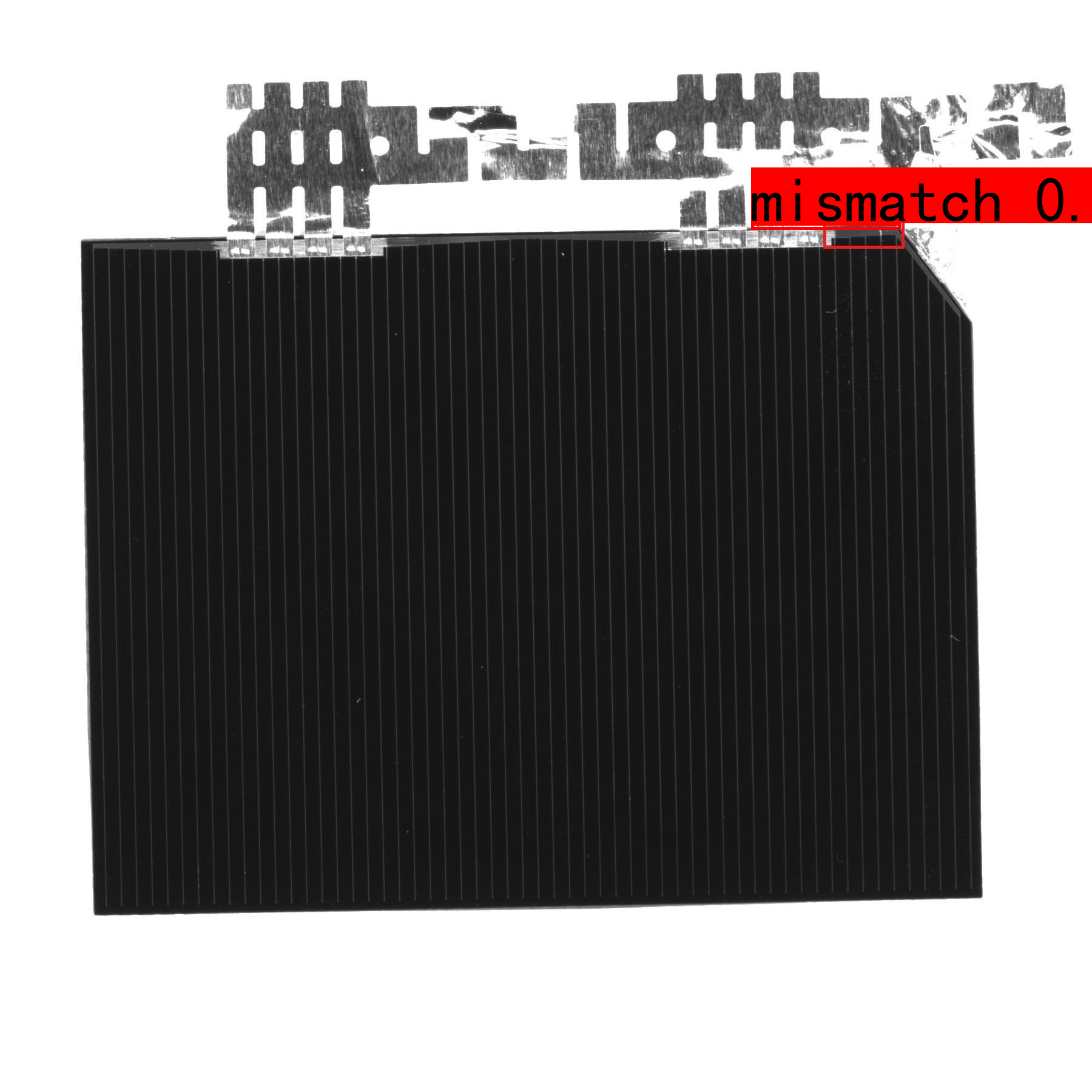

Supplement: S1 Dataset — (ZIP) [file pone.0304819.s001.zip › 00215mismatch_origin-copy_000001.png]

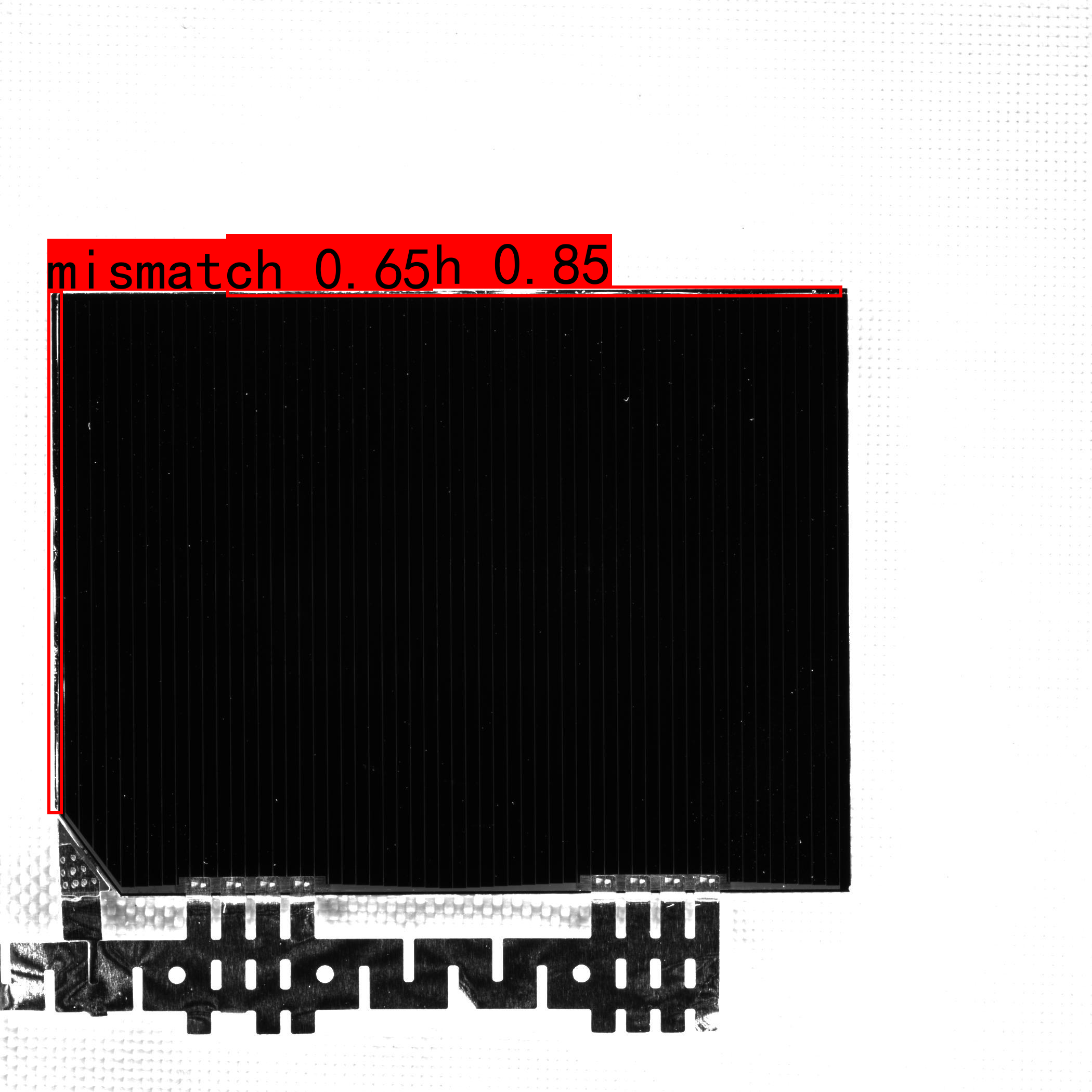

Supplement: S1 Dataset — (ZIP) [file pone.0304819.s001.zip › 00215mismatch_updown.png]

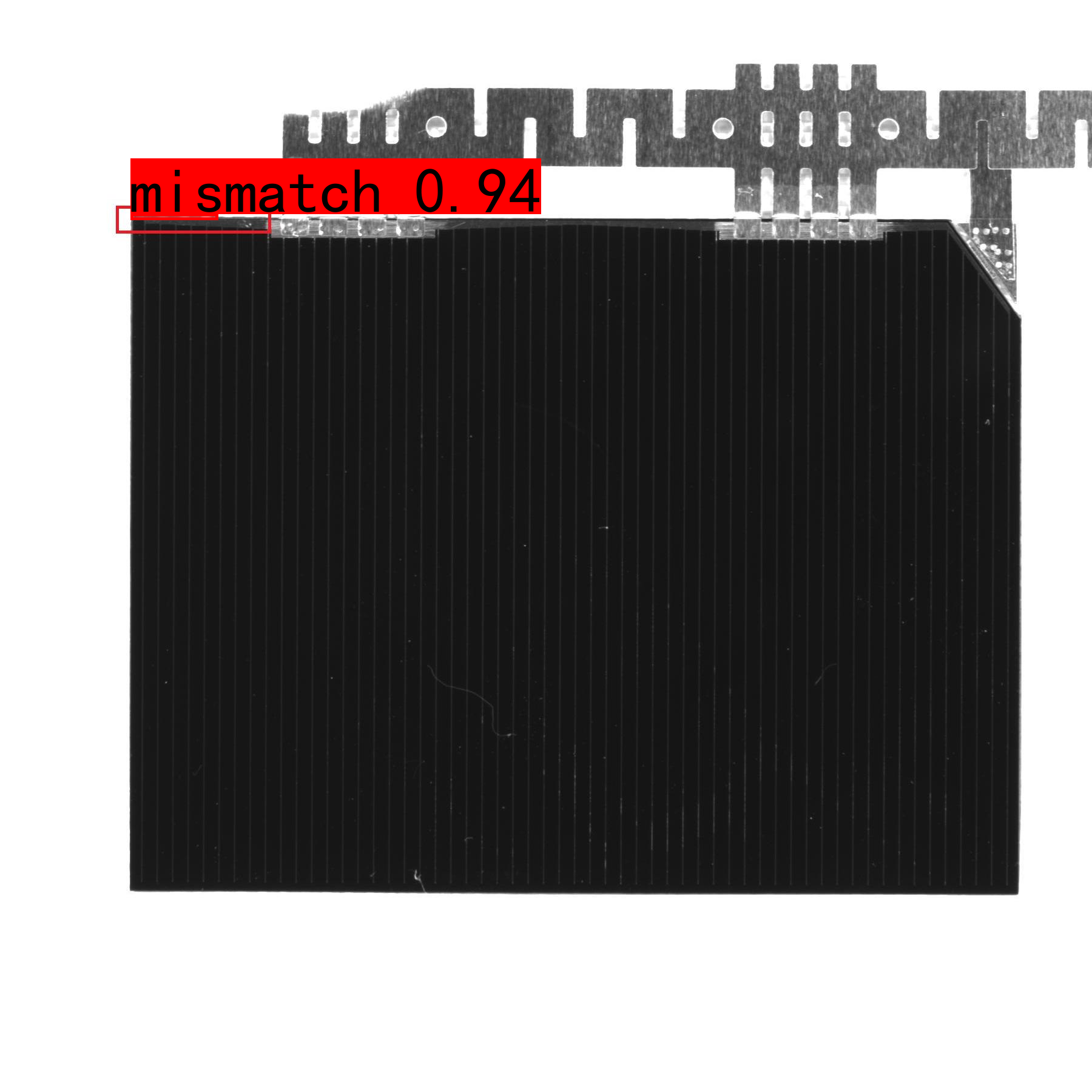

Supplement: S1 Dataset — (ZIP) [file pone.0304819.s001.zip › 00227mismatch_origin-copy_000001.png]

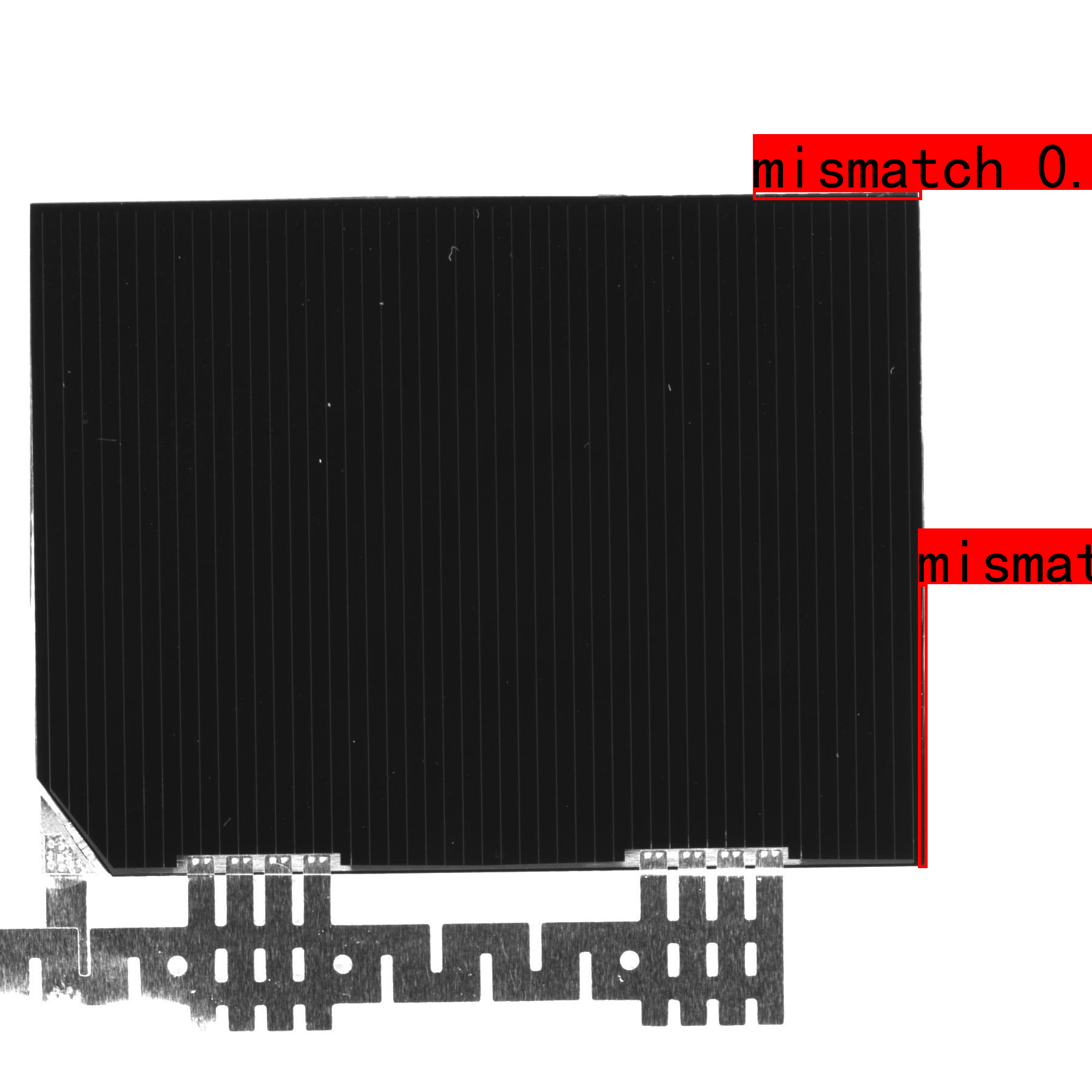

Supplement: S1 Dataset — (ZIP) [file pone.0304819.s001.zip › 00227mismatch_updown.png]

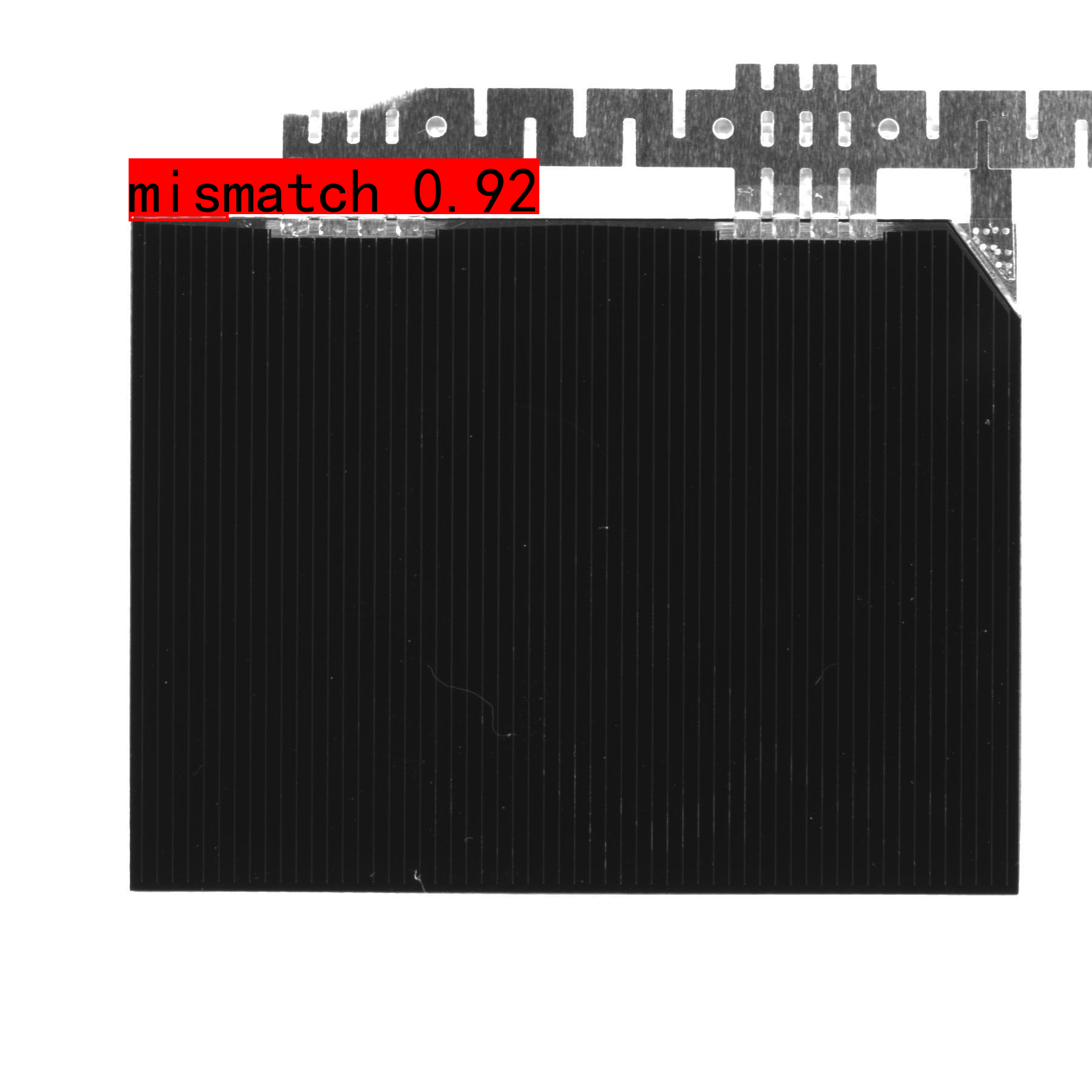

Supplement: S1 Dataset — (ZIP) [file pone.0304819.s001.zip › 00228mismatch_origin-copy_000001.png]

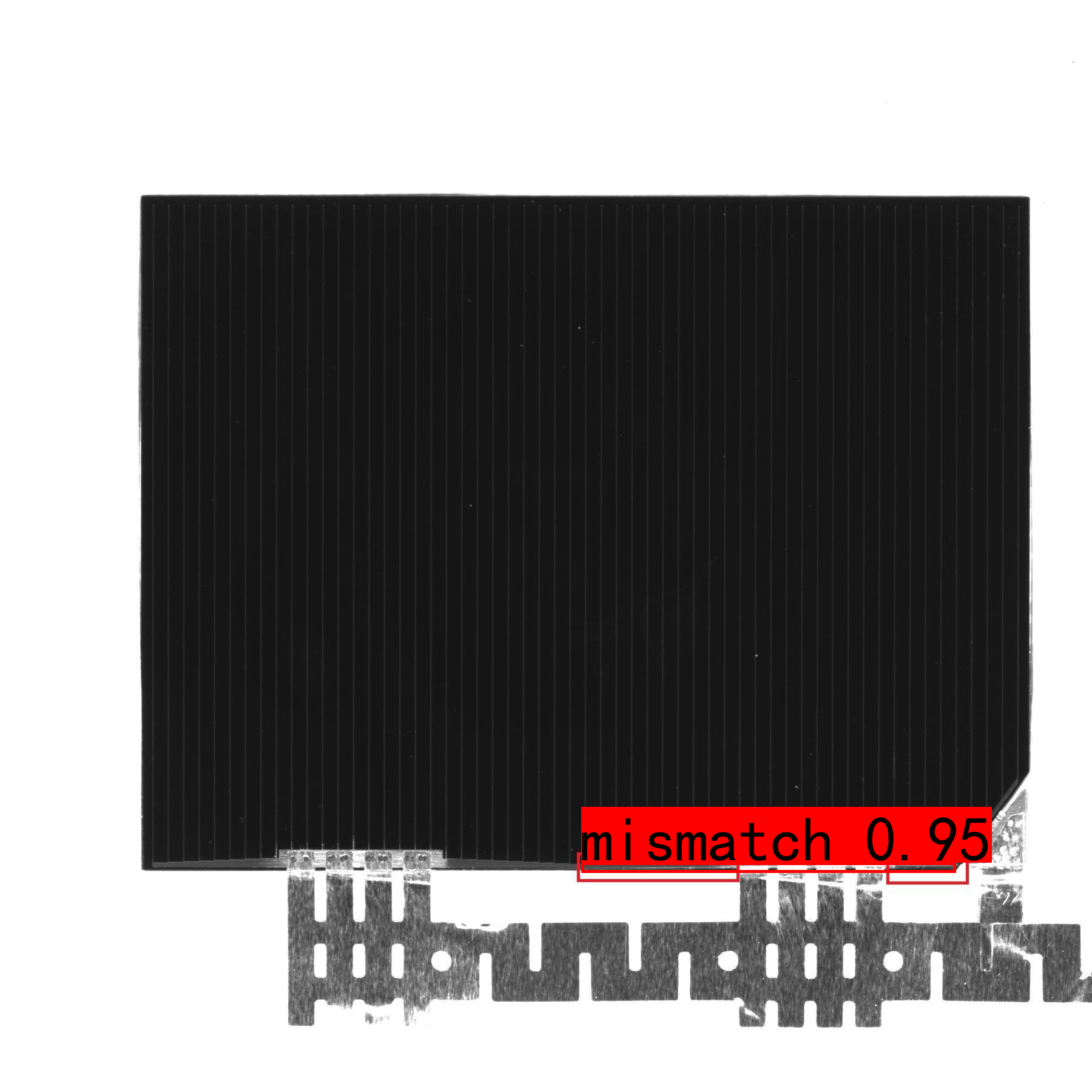

Supplement: S1 Dataset — (ZIP) [file pone.0304819.s001.zip › 00238mismatch_updown.png]

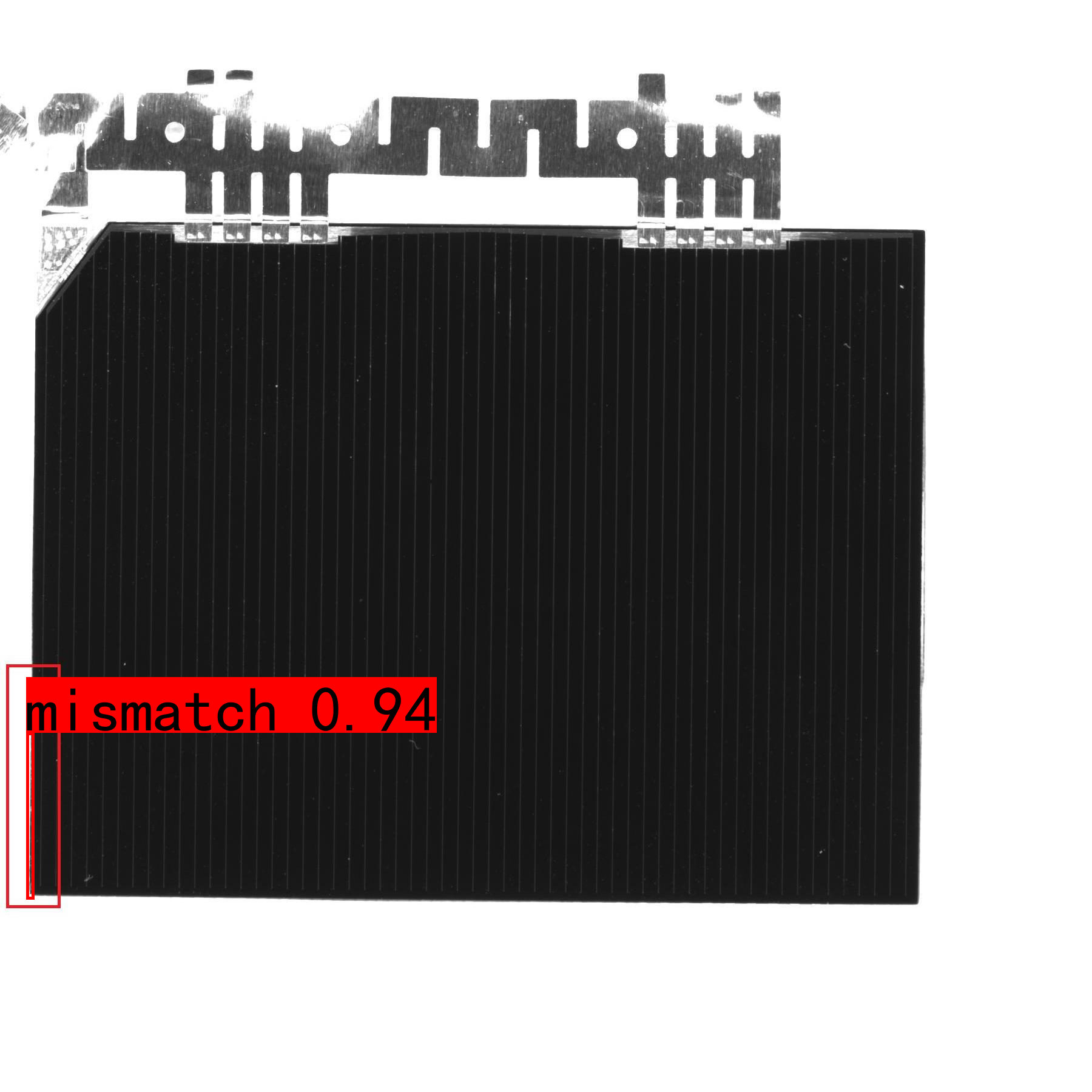

Supplement: S1 Dataset — (ZIP) [file pone.0304819.s001.zip › 00239mismatch_origin-copy_000001.png]

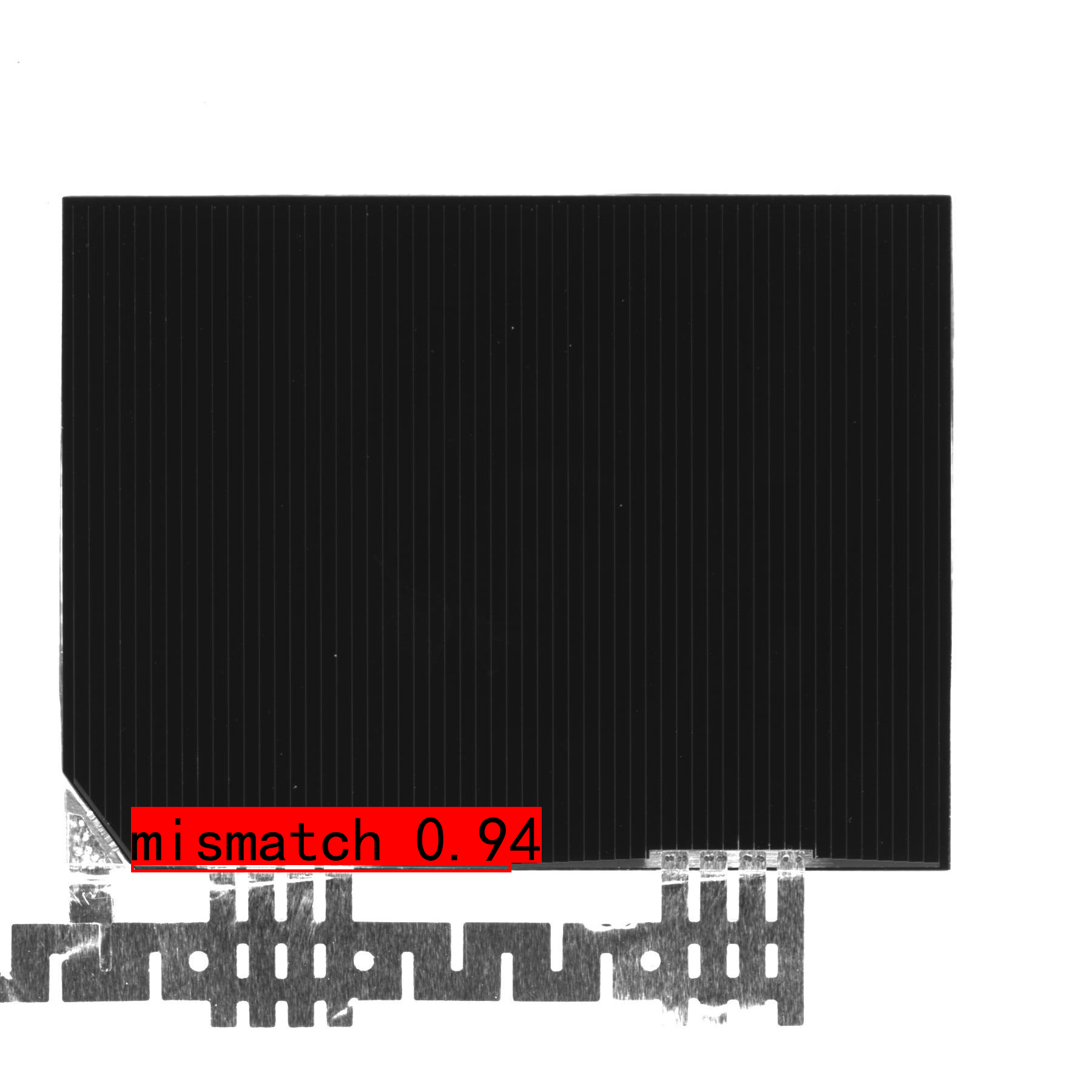

Supplement: S1 Dataset — (ZIP) [file pone.0304819.s001.zip › 00239mismatch_updown.png]

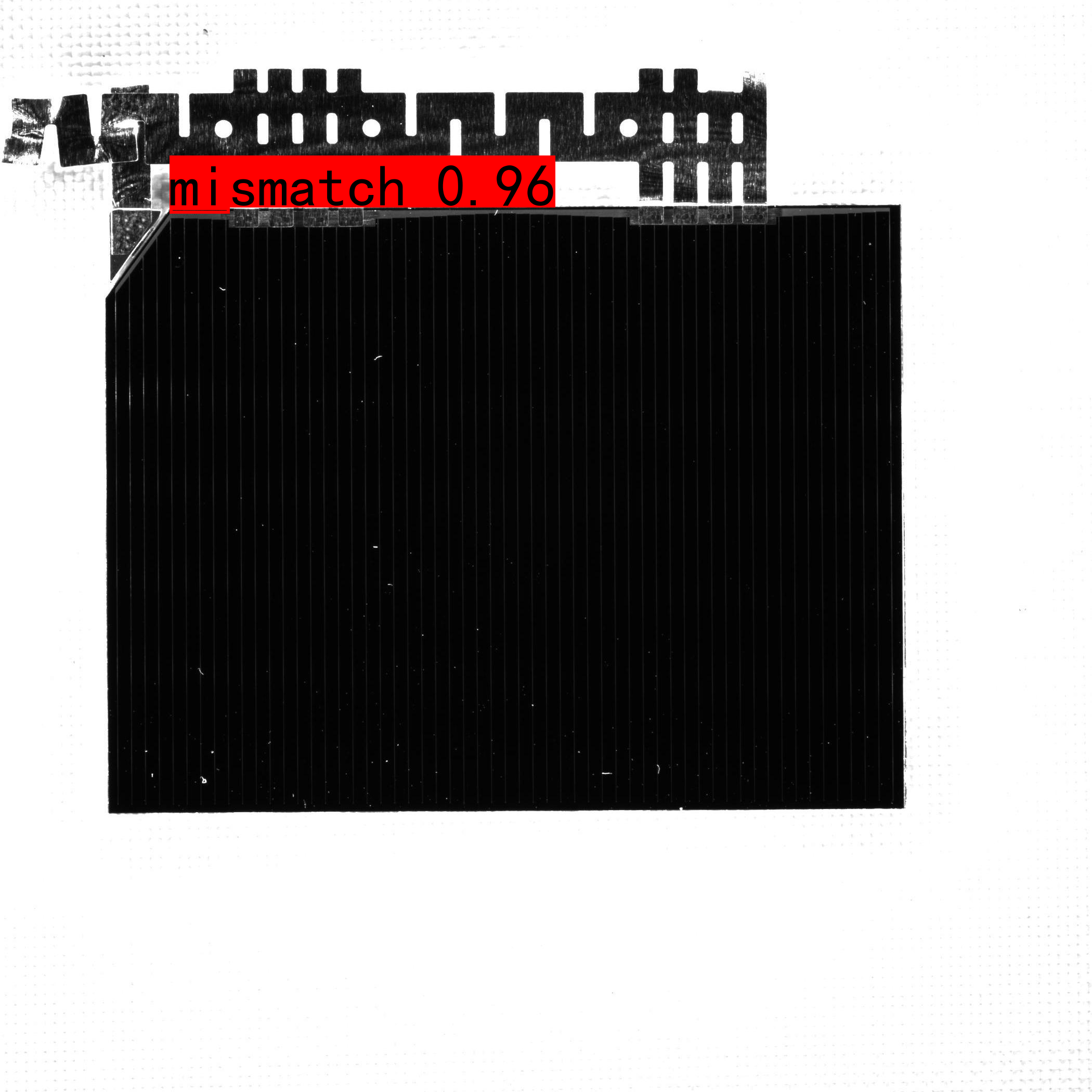

Supplement: S1 Dataset — (ZIP) [file pone.0304819.s001.zip › 00252mismatch_origin-copy_000001.png]

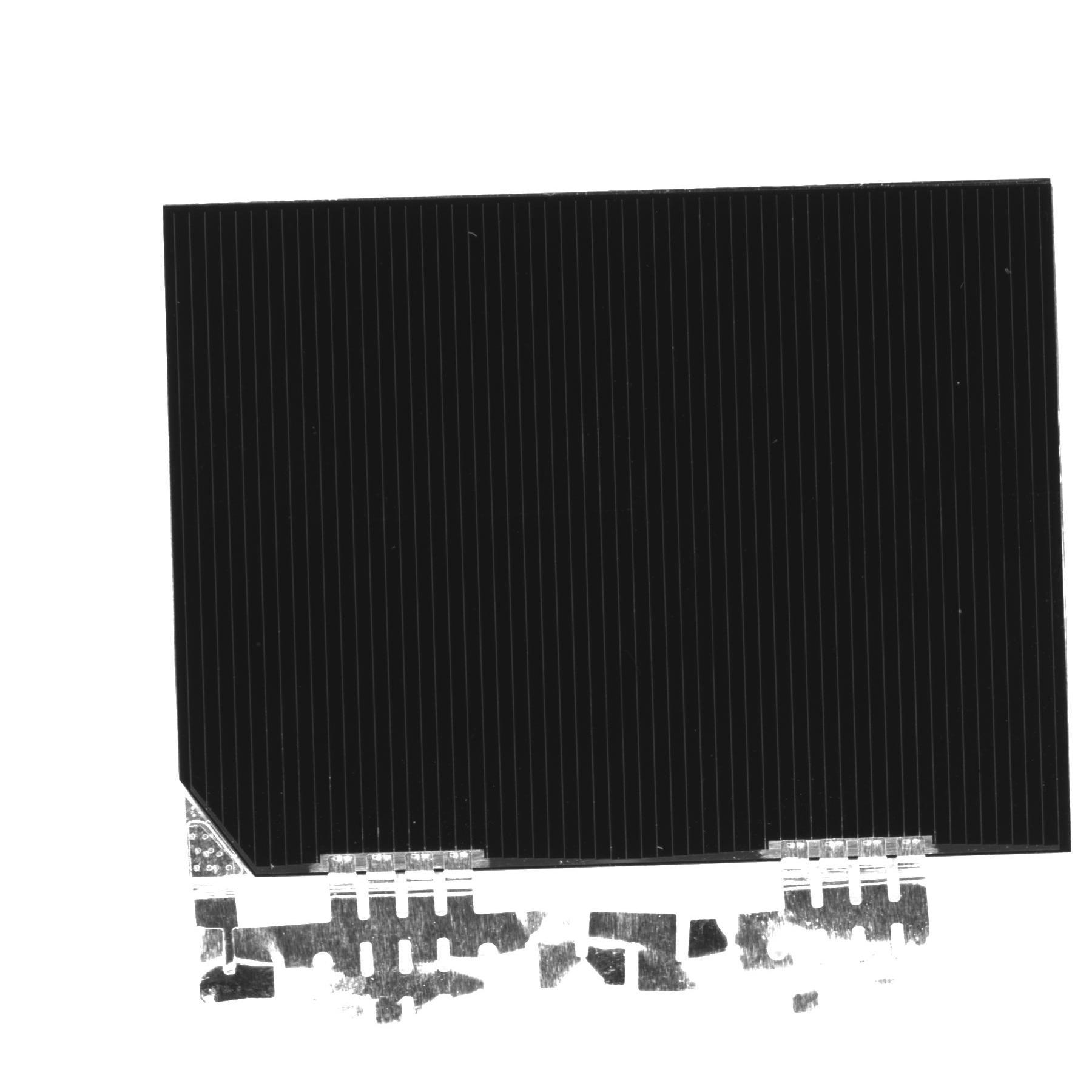

Supplement: S1 Dataset — (ZIP) [file pone.0304819.s001.zip › 00252mismatch_updown.png]

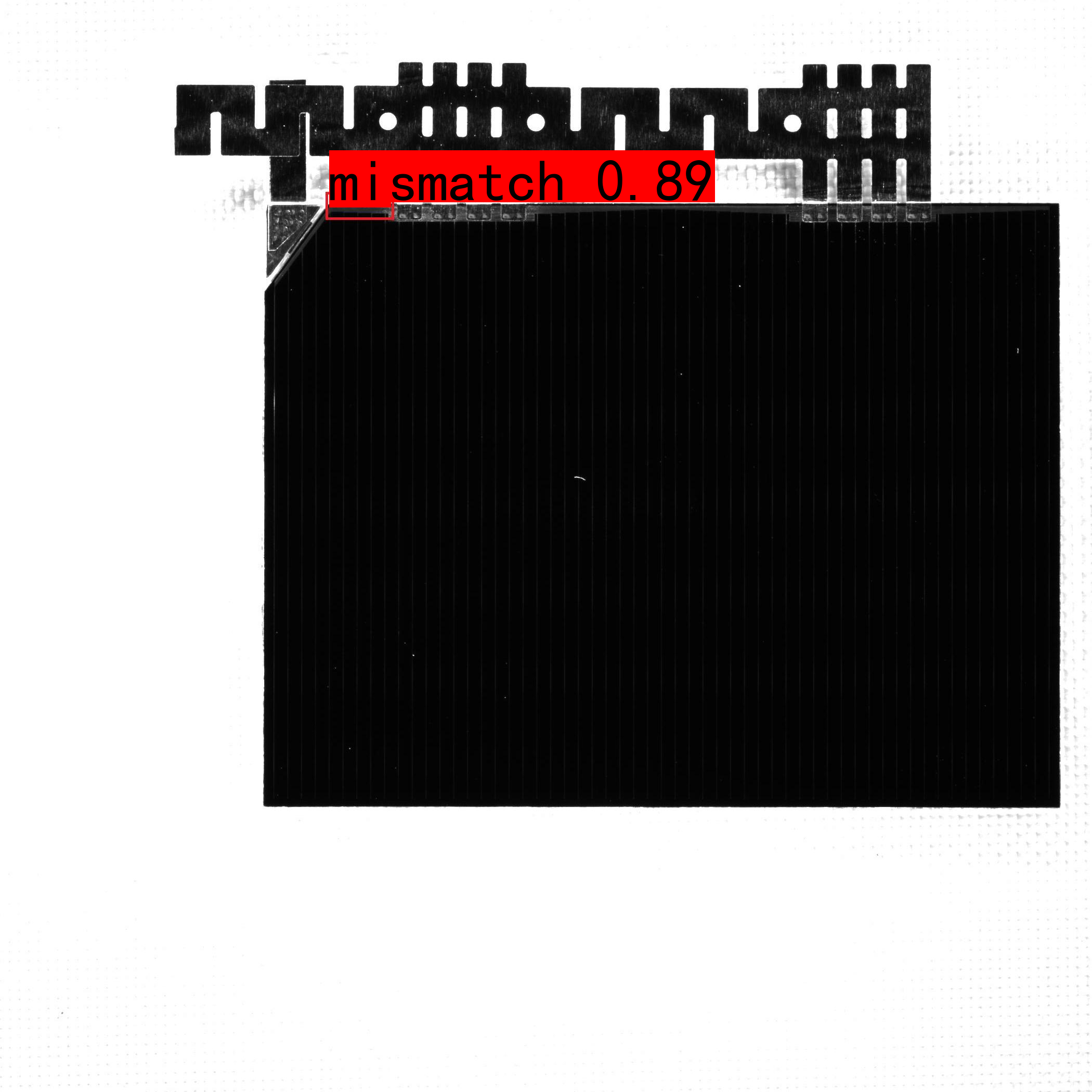

Supplement: S1 Dataset — (ZIP) [file pone.0304819.s001.zip › 00253mismatch_origin-copy_000001.png]

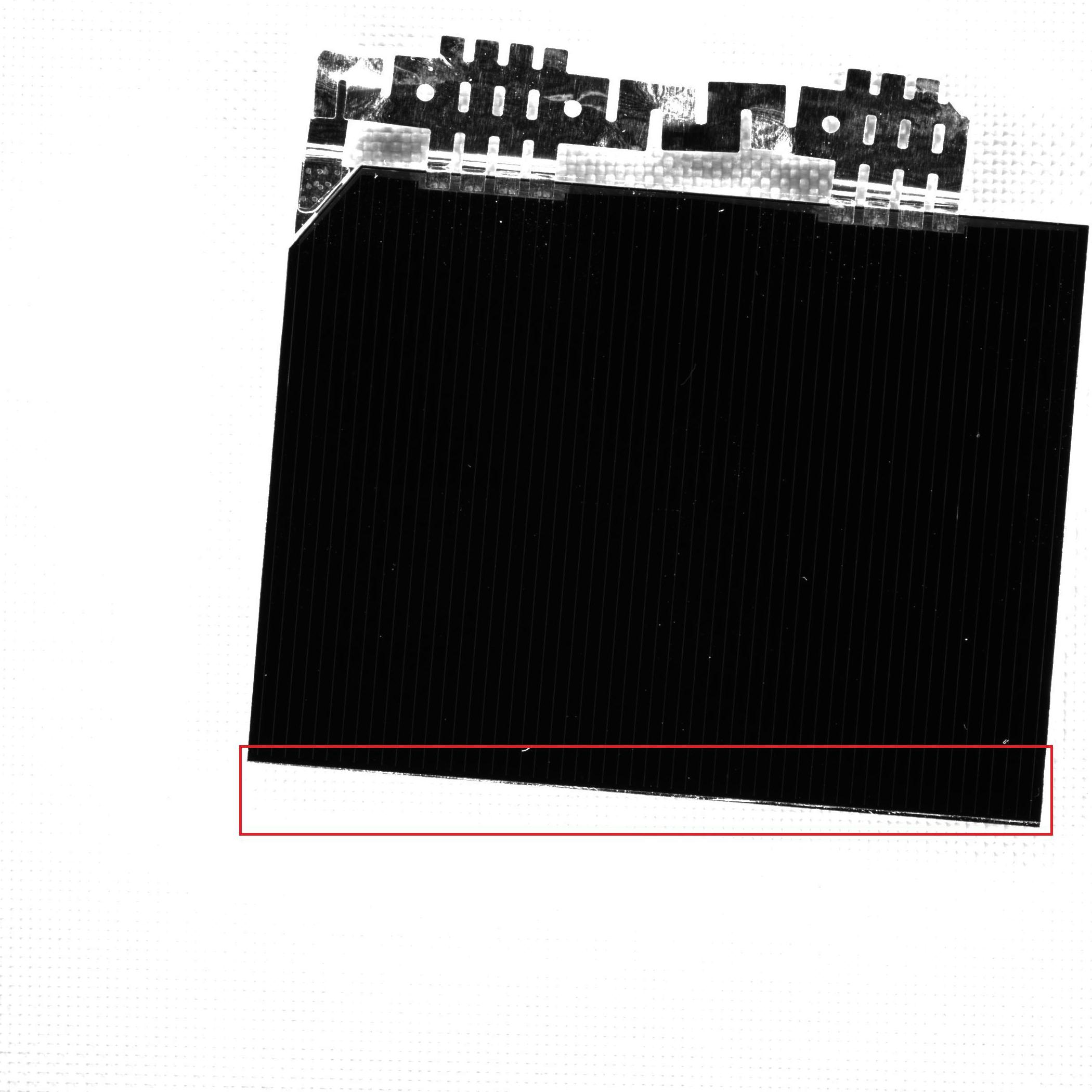

Supplement: S1 Dataset — (ZIP) [file pone.0304819.s001.zip › 00266mismatch_updown.png]

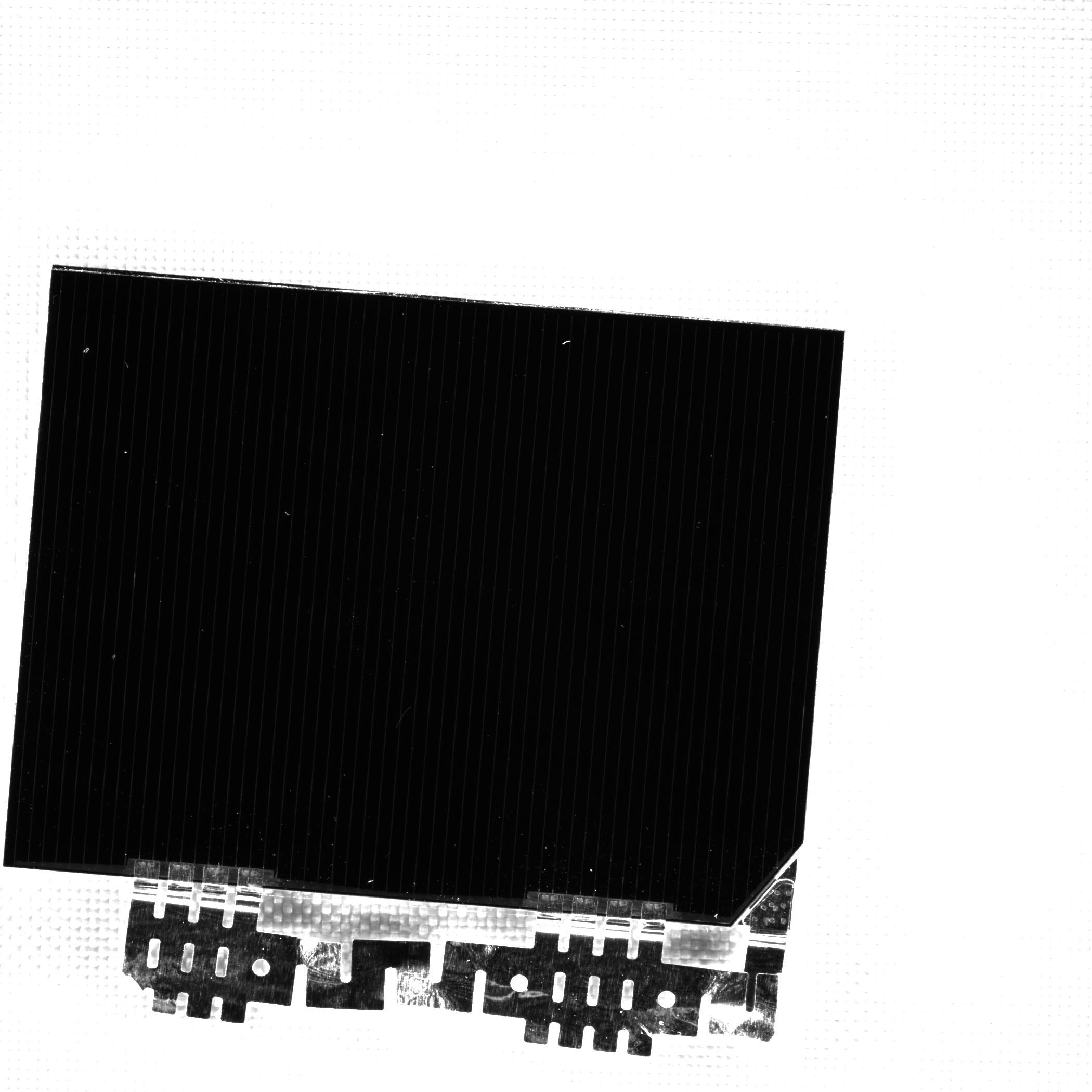

Supplement: S1 Dataset — (ZIP) [file pone.0304819.s001.zip › 00267mismatch_updown.png]

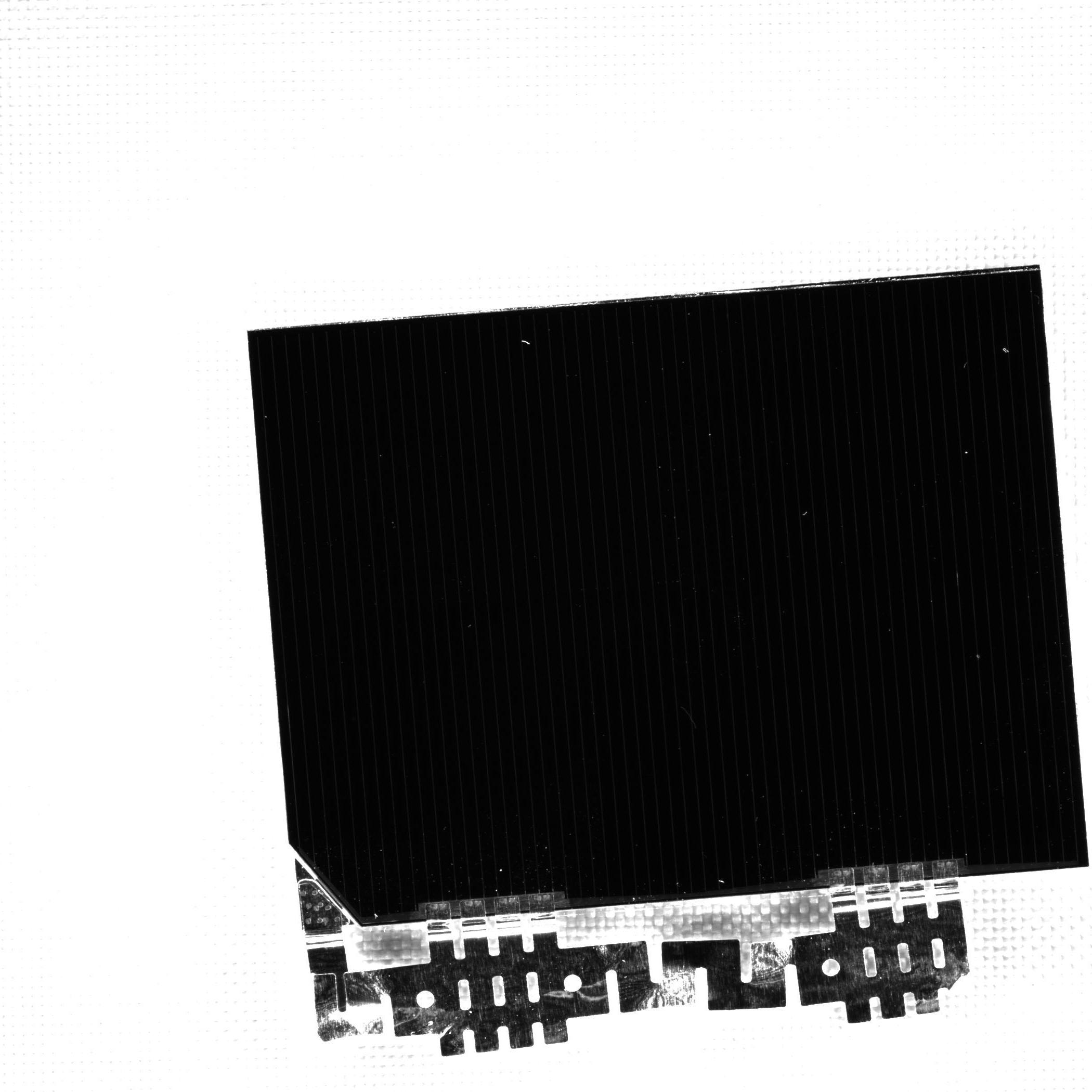

Supplement: S1 Dataset — (ZIP) [file pone.0304819.s001.zip › 00268mismatch_updown.png]

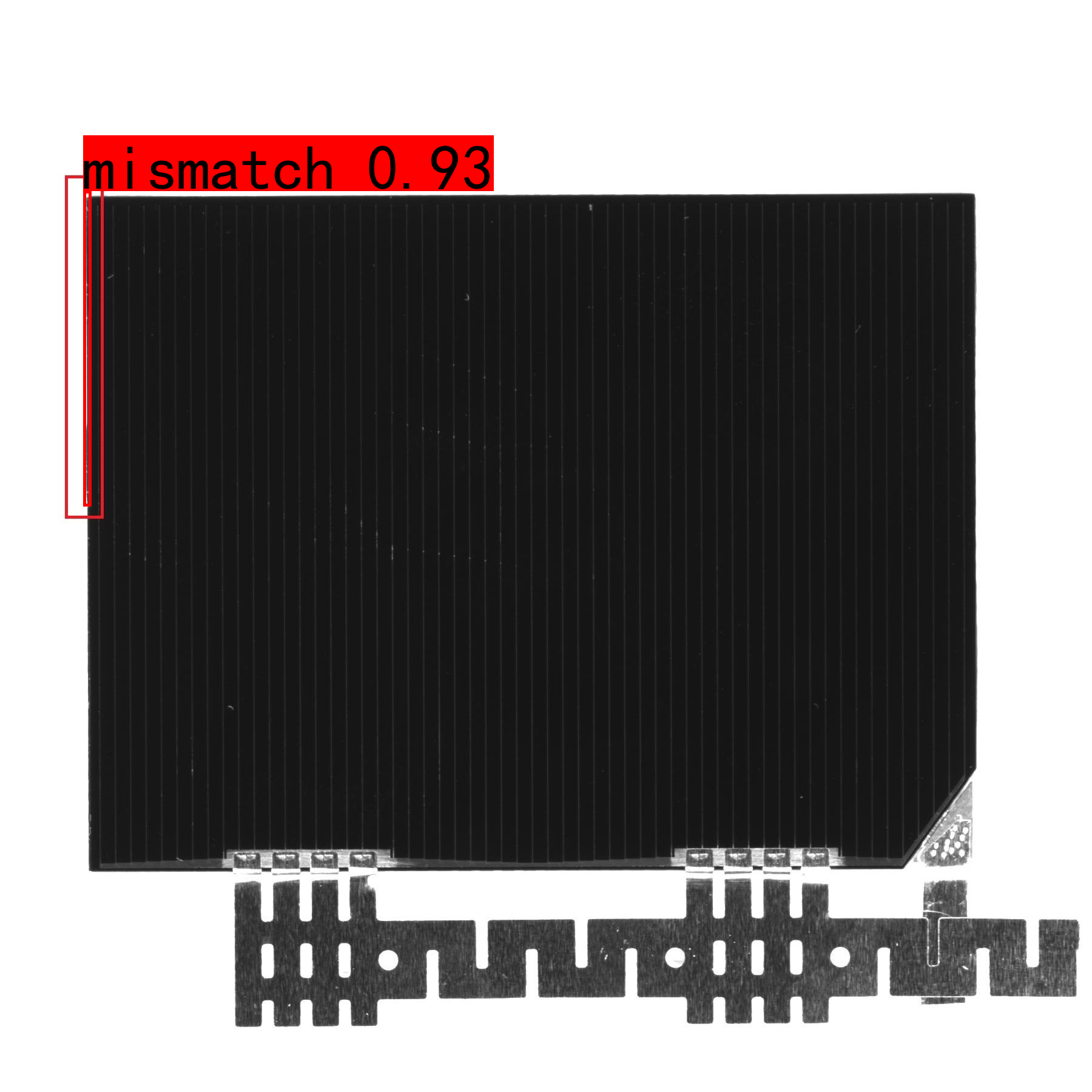

Supplement: S1 Dataset — (ZIP) [file pone.0304819.s001.zip › 00289mismatch_updown.png]

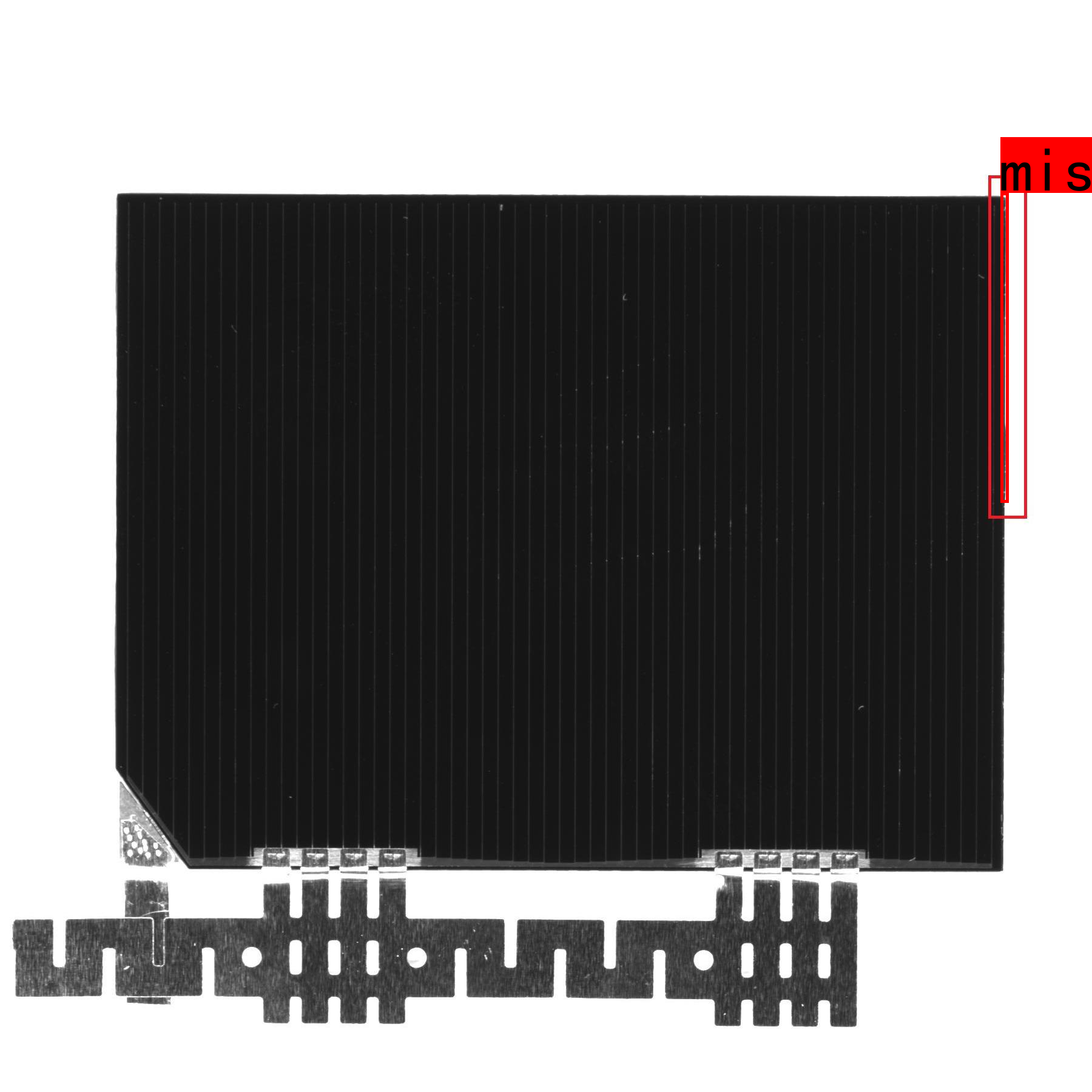

Supplement: S1 Dataset — (ZIP) [file pone.0304819.s001.zip › 00290mismatch_updown.png]

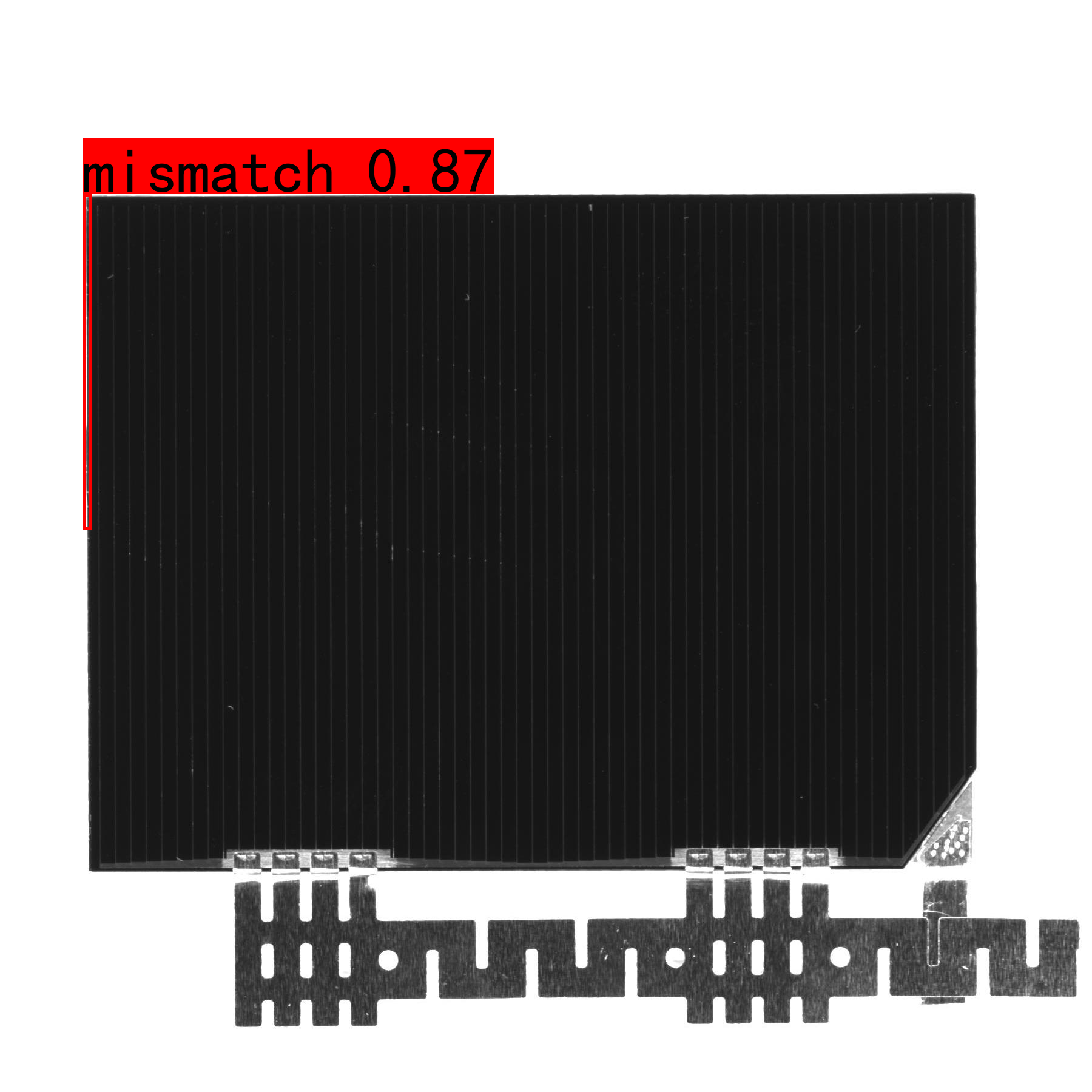

Supplement: S1 Dataset — (ZIP) [file pone.0304819.s001.zip › 00291mismatch_updown.png]

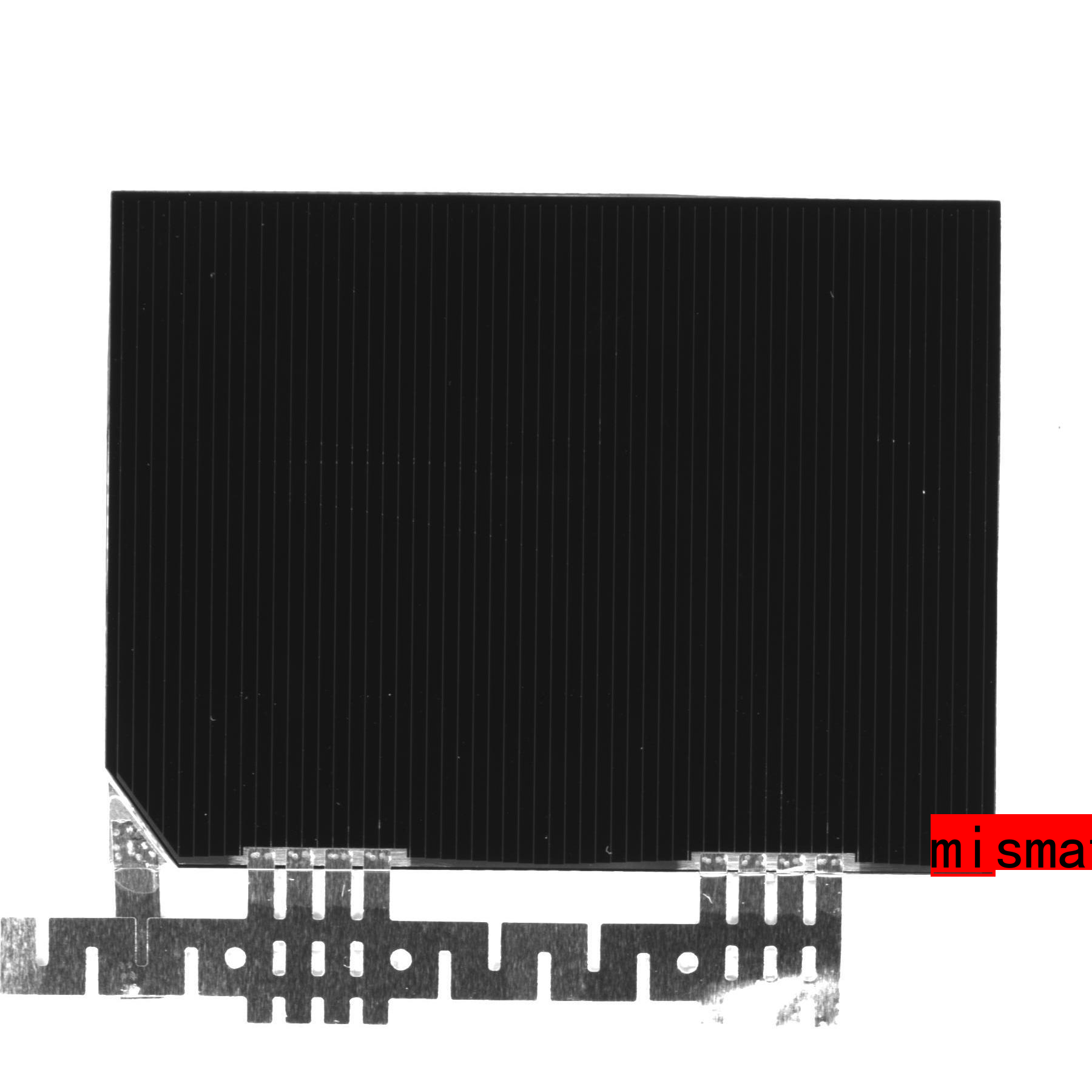

Supplement: S1 Dataset — (ZIP) [file pone.0304819.s001.zip › 00312mismatch_updown.png]

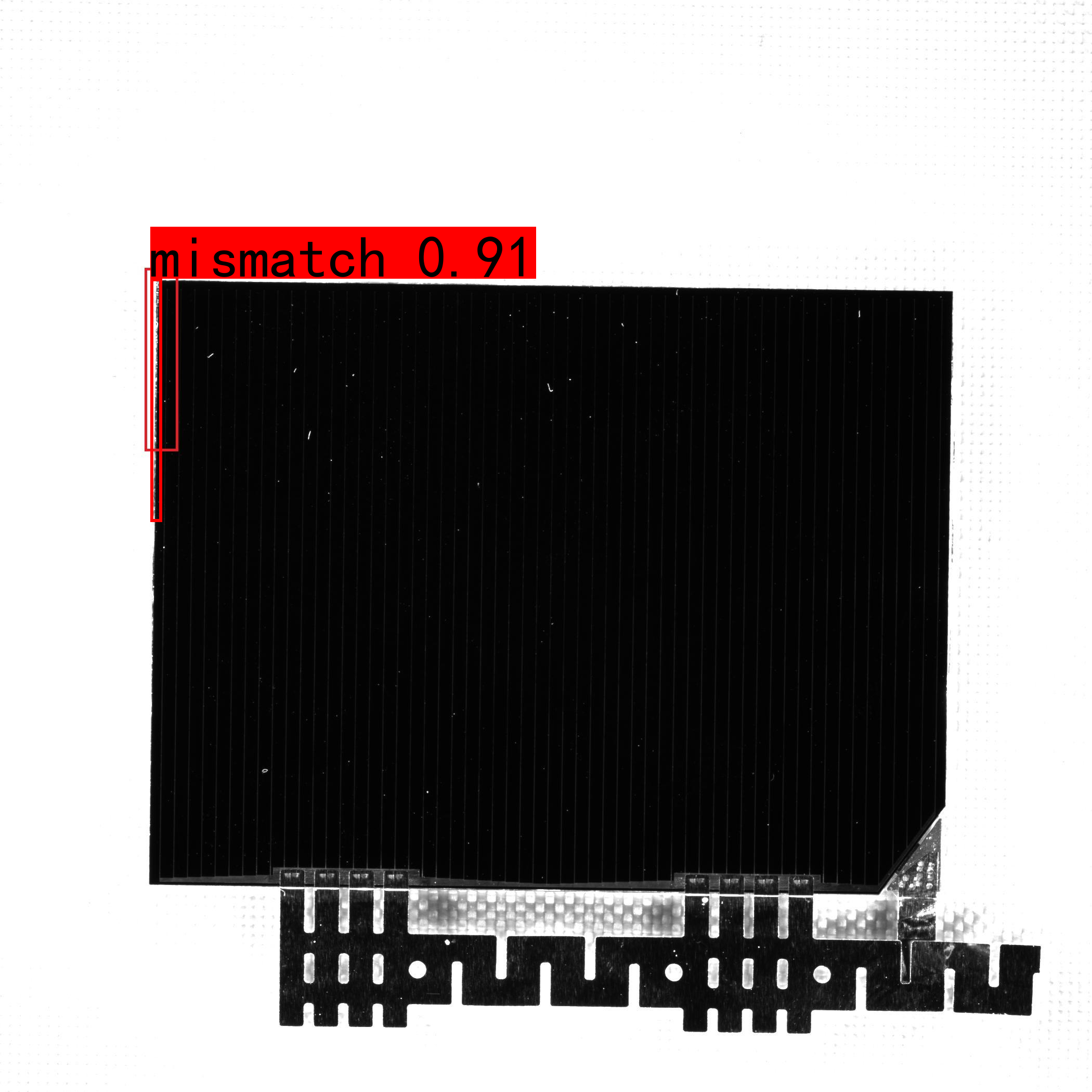

Supplement: S1 Dataset — (ZIP) [file pone.0304819.s001.zip › 00313mismatch_updown.png]

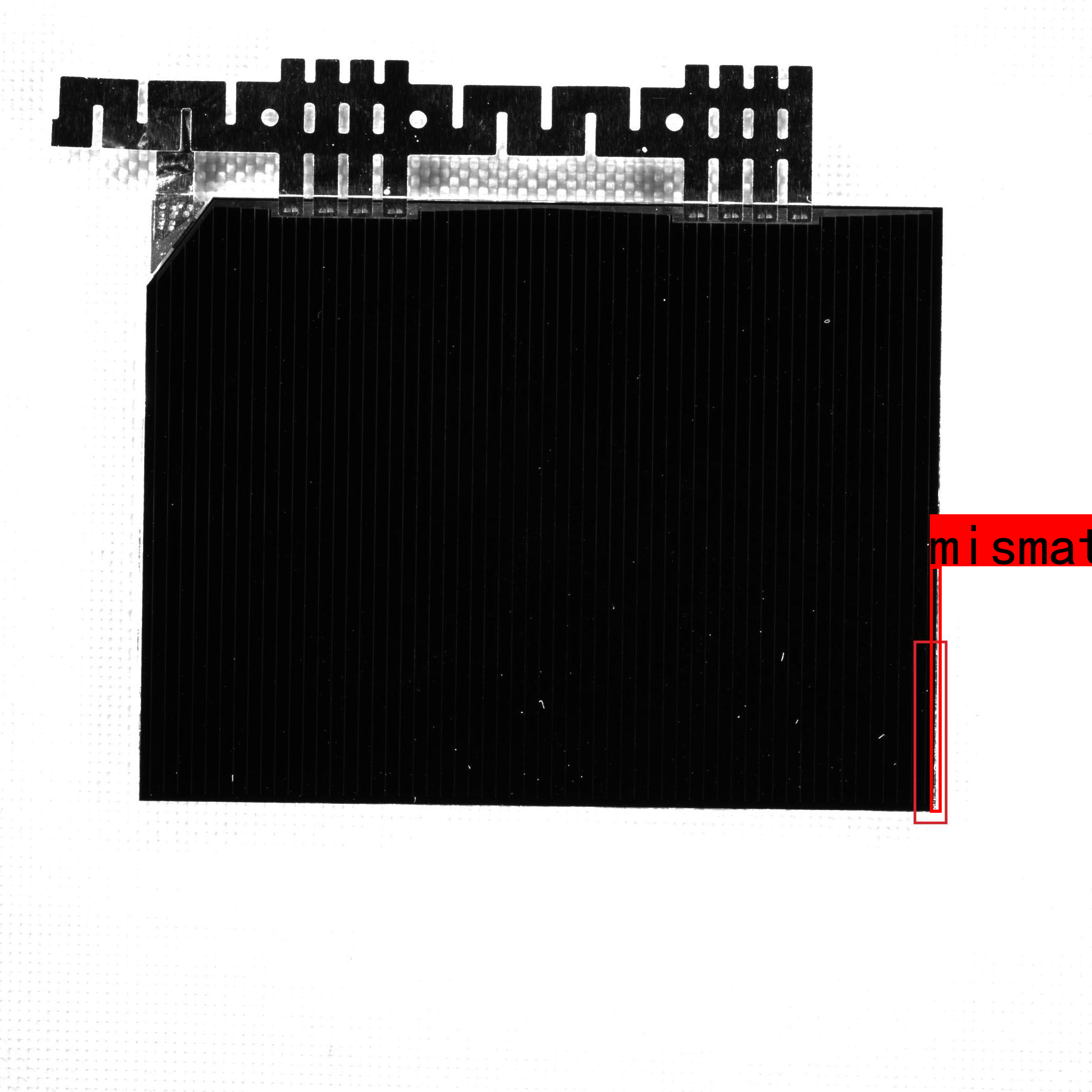

Supplement: S1 Dataset — (ZIP) [file pone.0304819.s001.zip › 00314mismatch_updown.png]

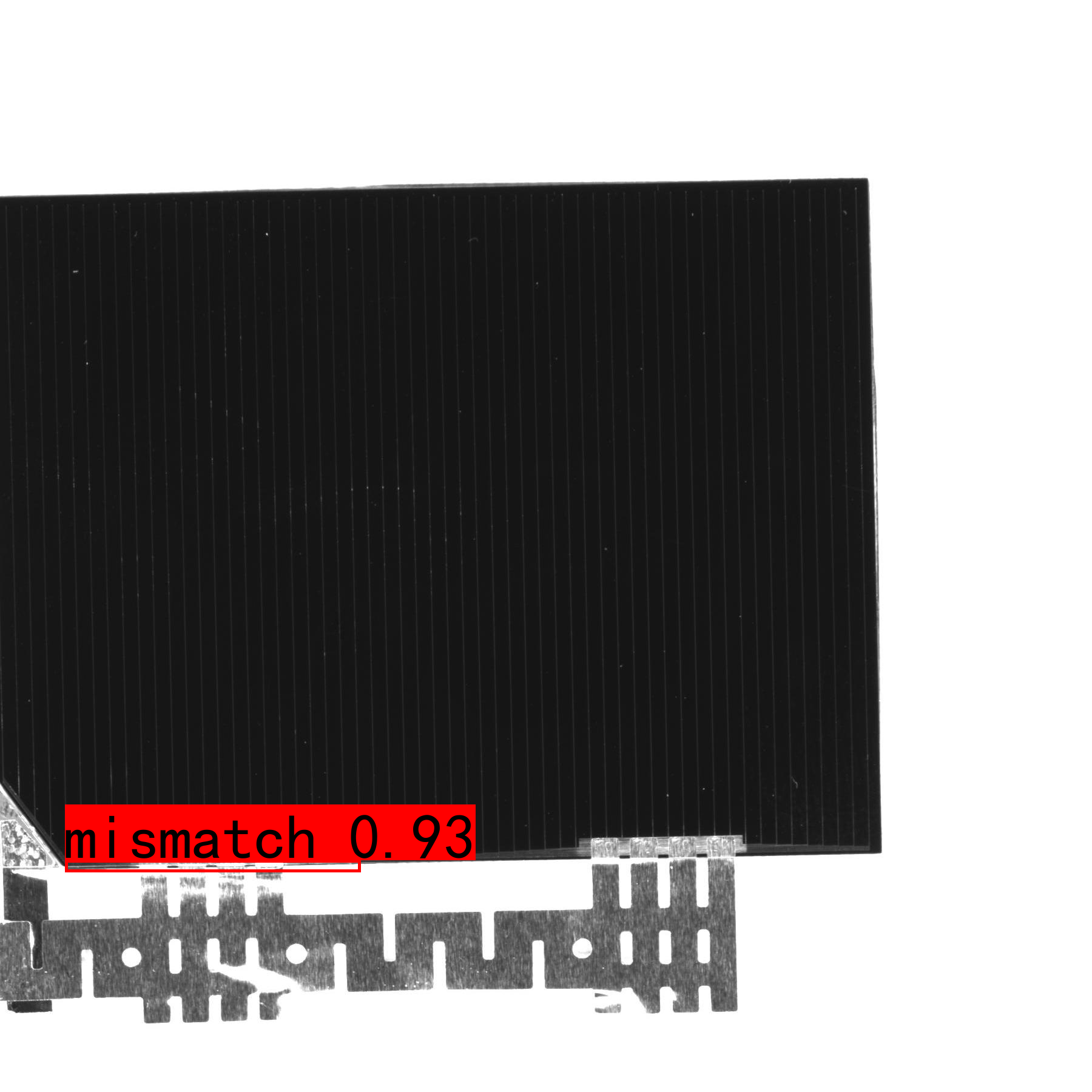

Supplement: S1 Dataset — (ZIP) [file pone.0304819.s001.zip › 00335mismatch_updown.png]

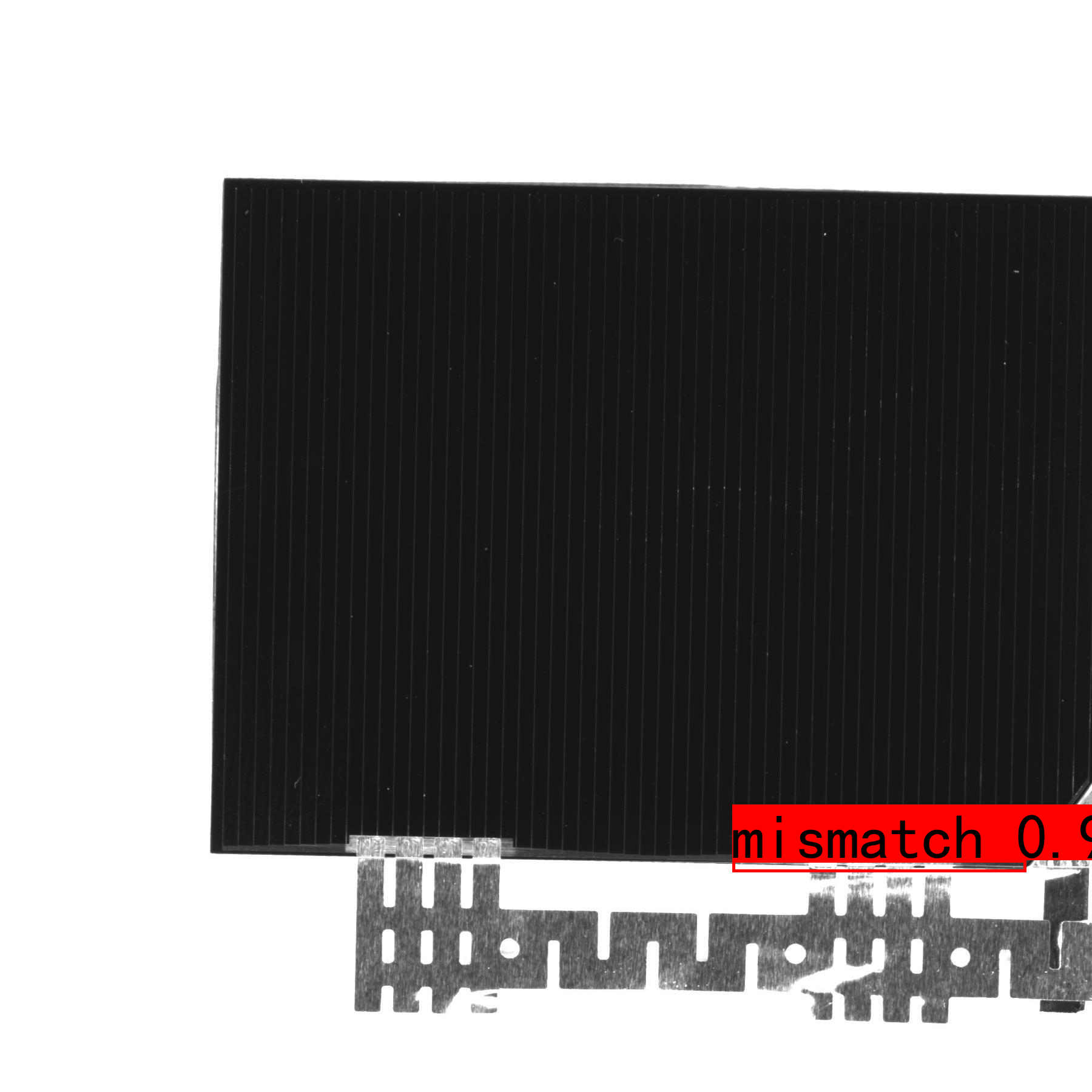

Supplement: S1 Dataset — (ZIP) [file pone.0304819.s001.zip › 00336mismatch_updown.png]

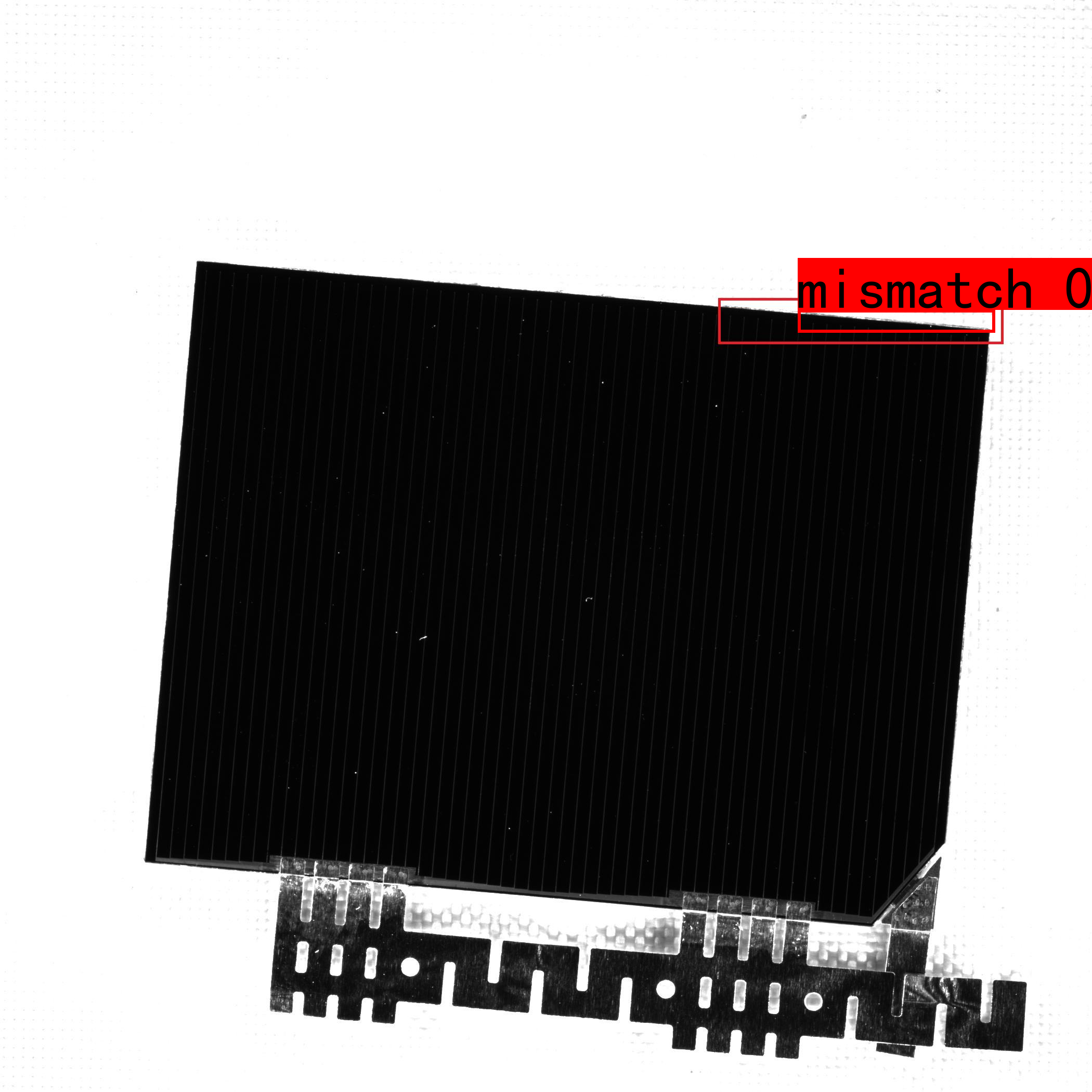

Supplement: S1 Dataset — (ZIP) [file pone.0304819.s001.zip › 00337mismatch_updown.png]

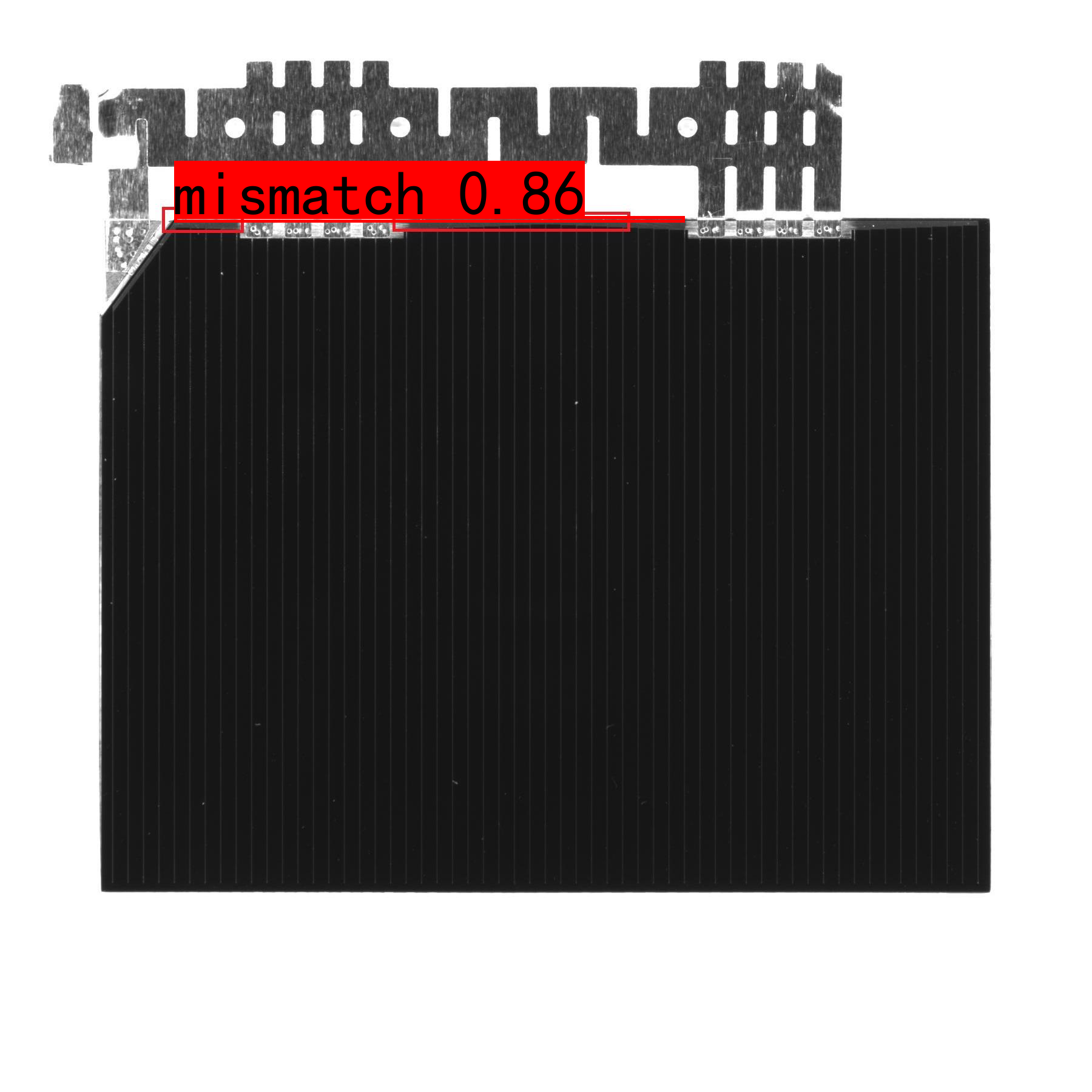

Supplement: S1 Dataset — (ZIP) [file pone.0304819.s001.zip › 00358mismatch_updown.png]

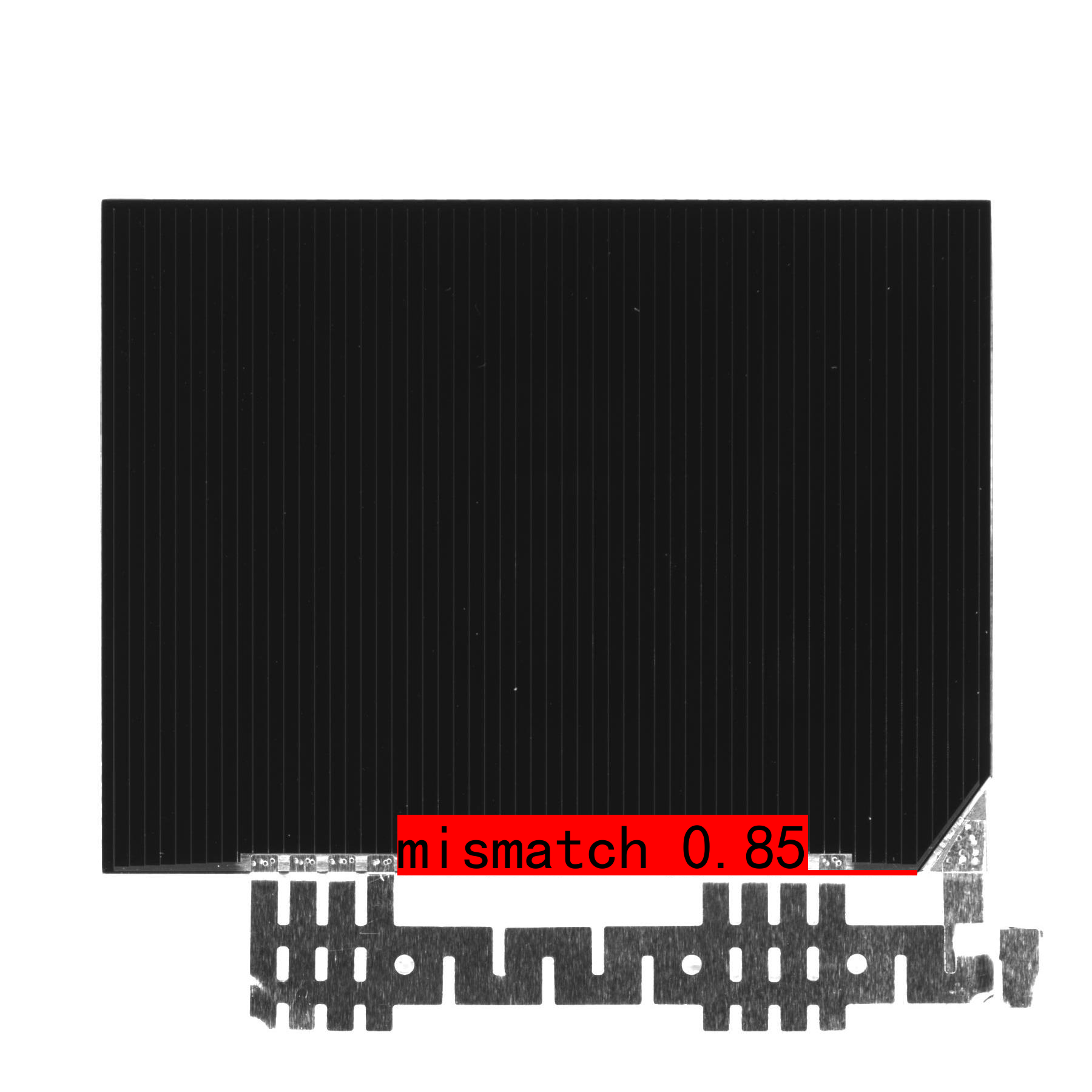

Supplement: S1 Dataset — (ZIP) [file pone.0304819.s001.zip › 00359mismatch_updown.png]

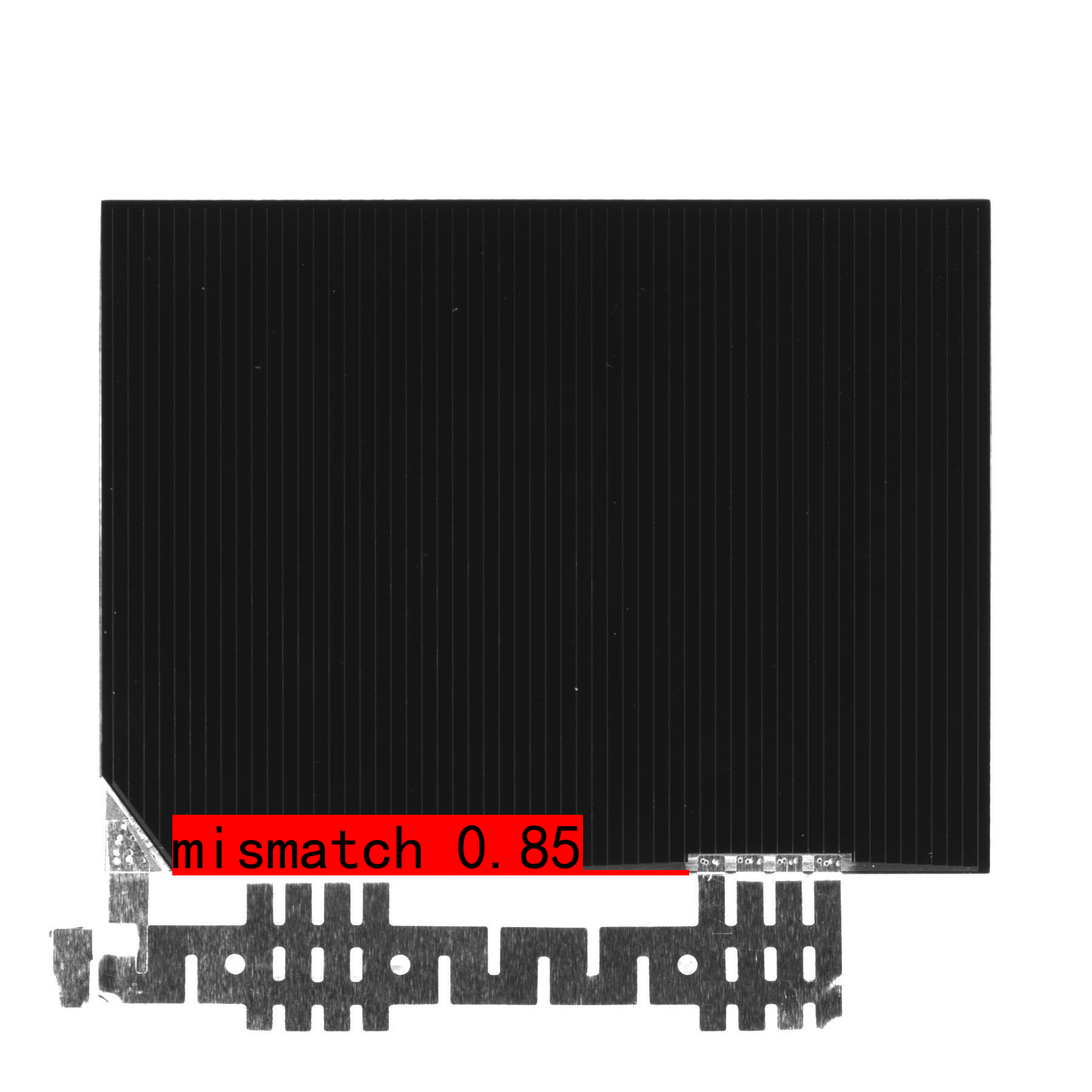

Supplement: S1 Dataset — (ZIP) [file pone.0304819.s001.zip › 00360mismatch_updown.png]

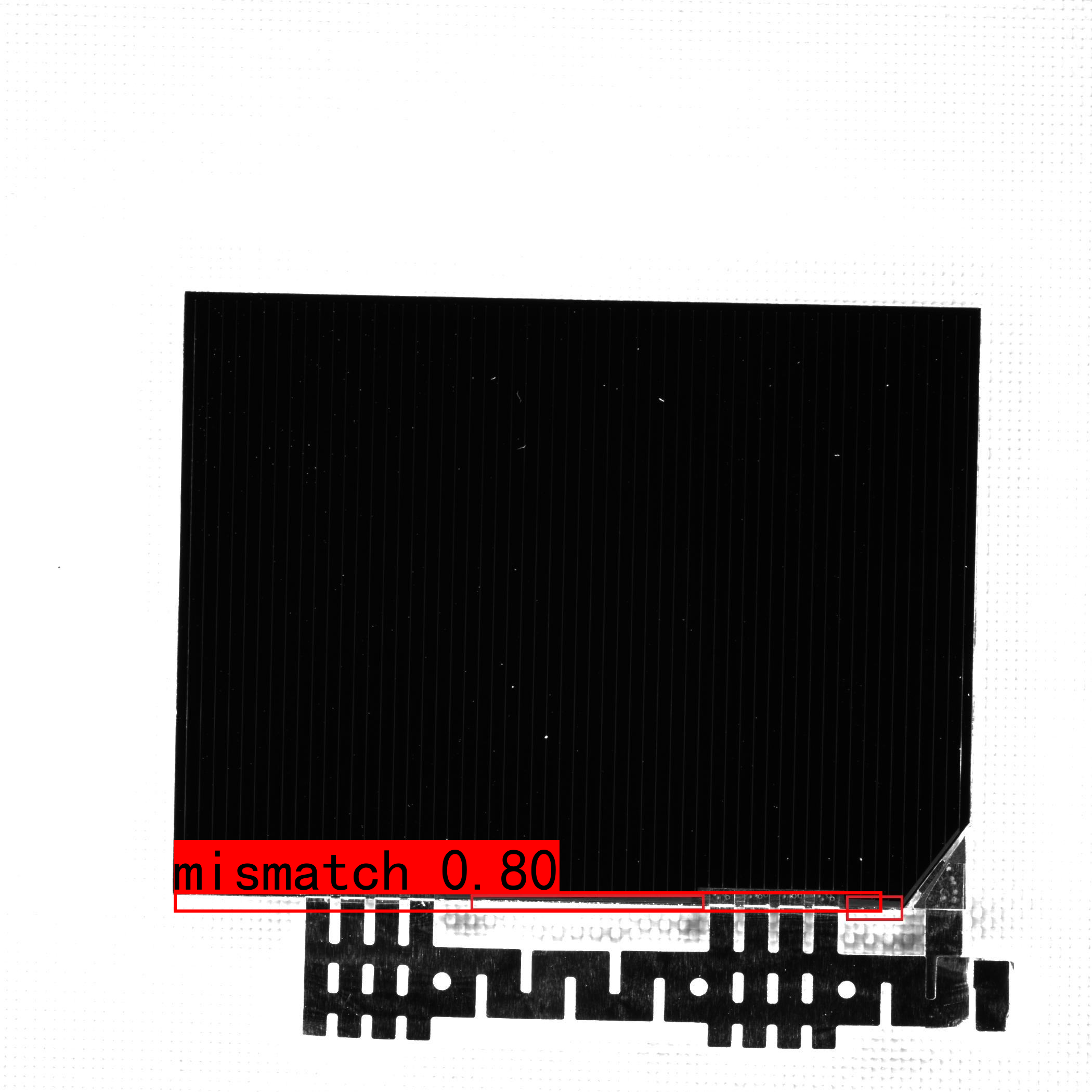

Supplement: S1 Dataset — (ZIP) [file pone.0304819.s001.zip › 00381mismatch_updown.png]

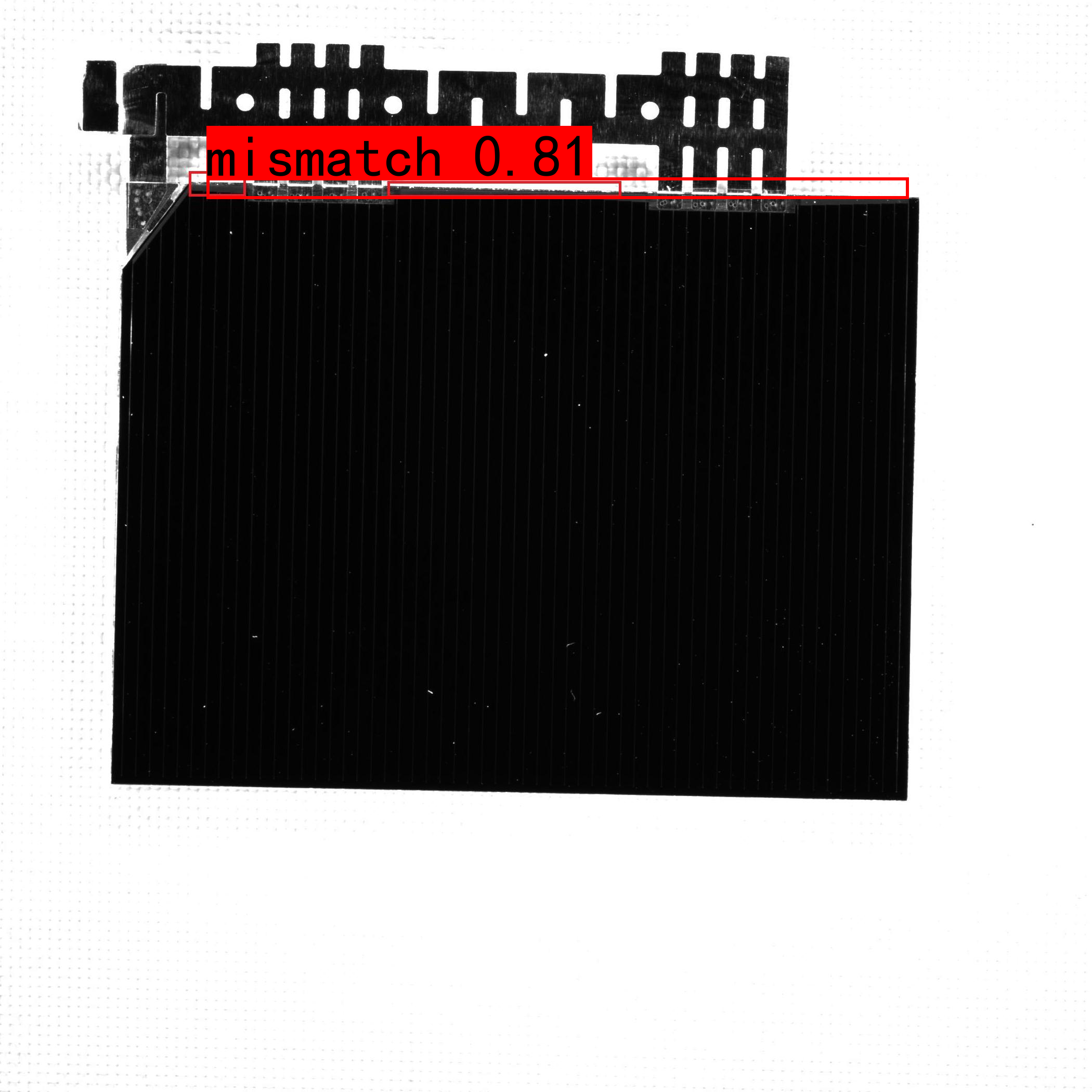

Supplement: S1 Dataset — (ZIP) [file pone.0304819.s001.zip › 00382mismatch_updown.png]

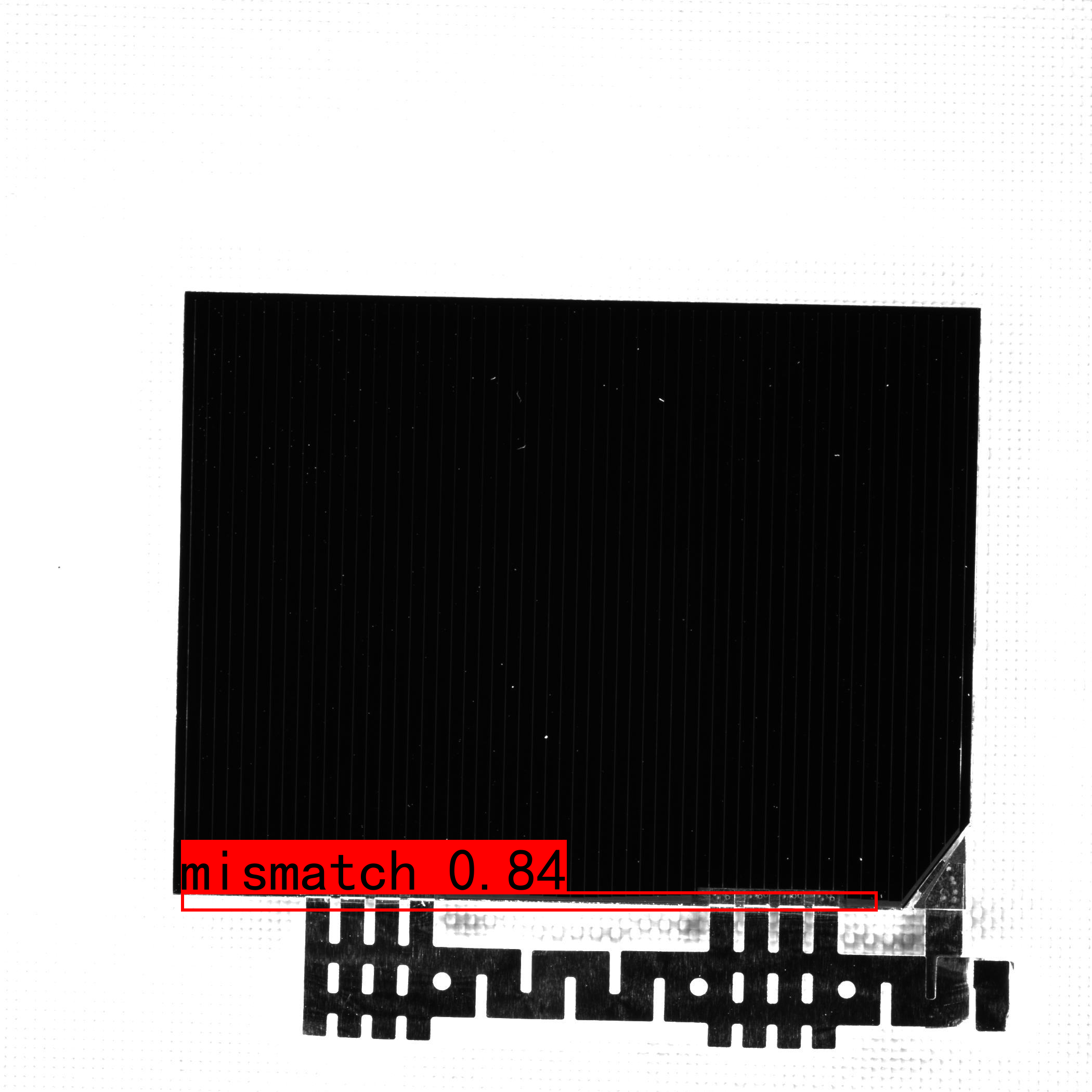

Supplement: S1 Dataset — (ZIP) [file pone.0304819.s001.zip › 00383mismatch_updown.png]

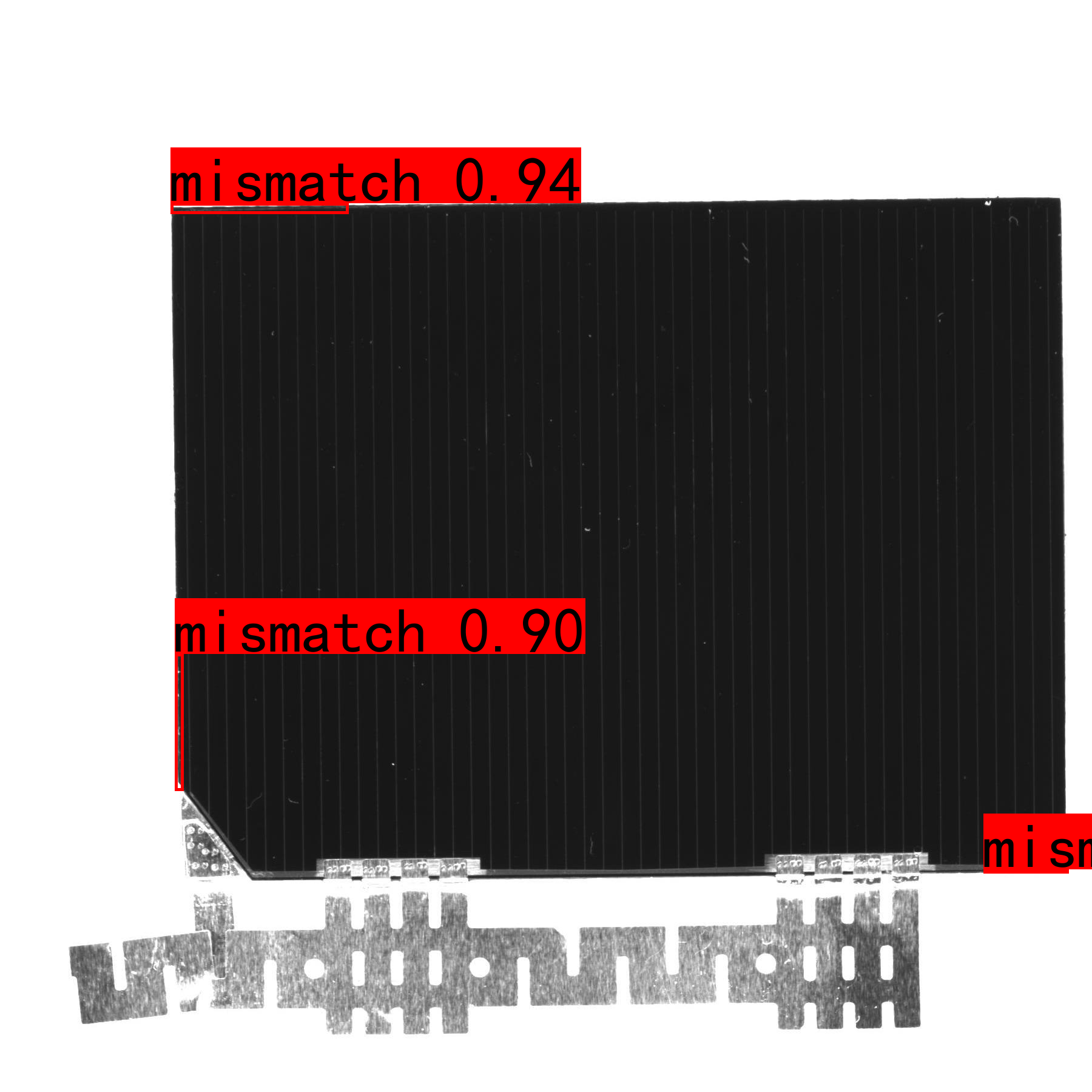

Supplement: S1 Dataset — (ZIP) [file pone.0304819.s001.zip › 00404mismatch_updown.png]

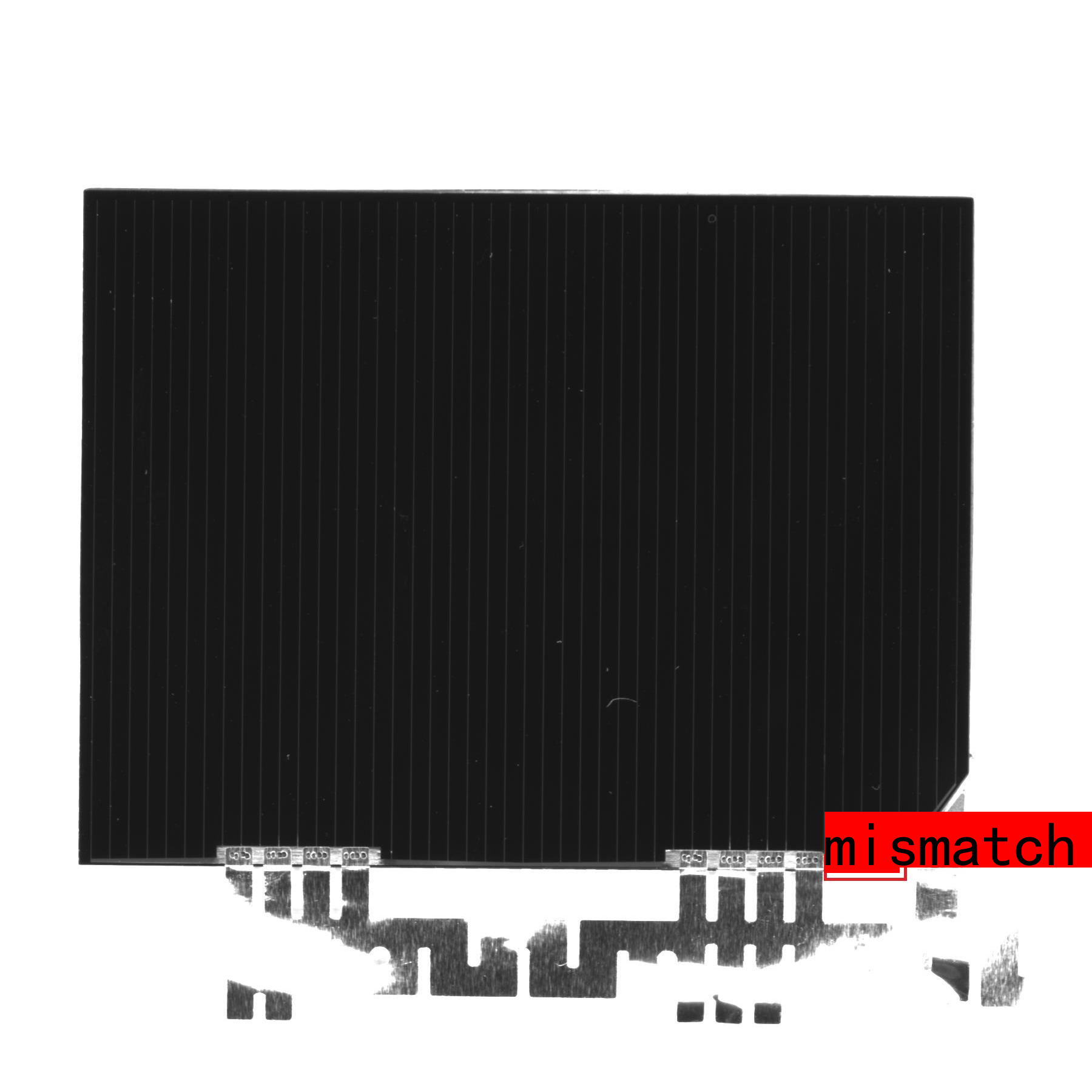

Supplement: S1 Dataset — (ZIP) [file pone.0304819.s001.zip › 00405mismatch_updown.png]

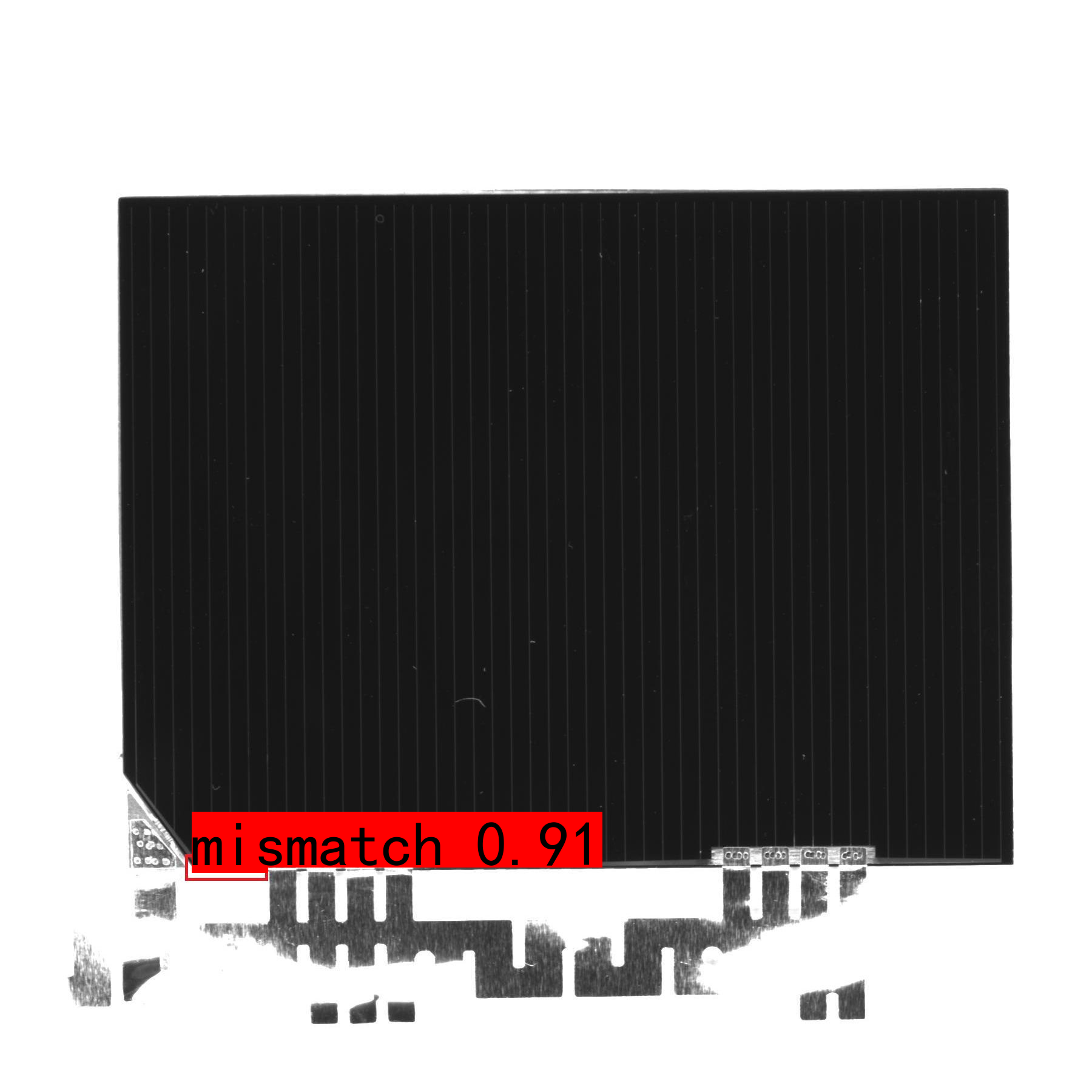

Supplement: S1 Dataset — (ZIP) [file pone.0304819.s001.zip › 00406mismatch_updown.png]

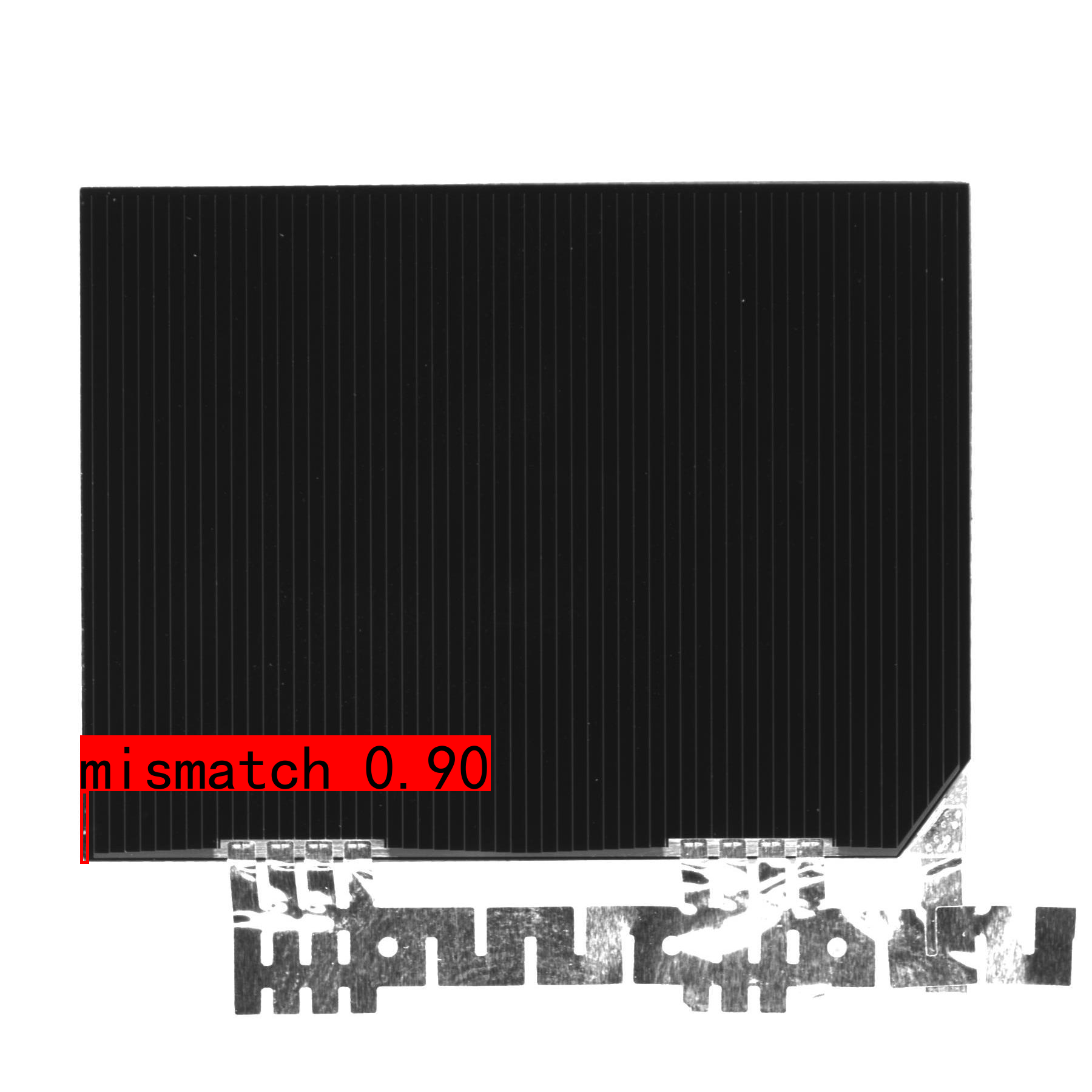

Supplement: S1 Dataset — (ZIP) [file pone.0304819.s001.zip › 00427mismatch_updown.png]

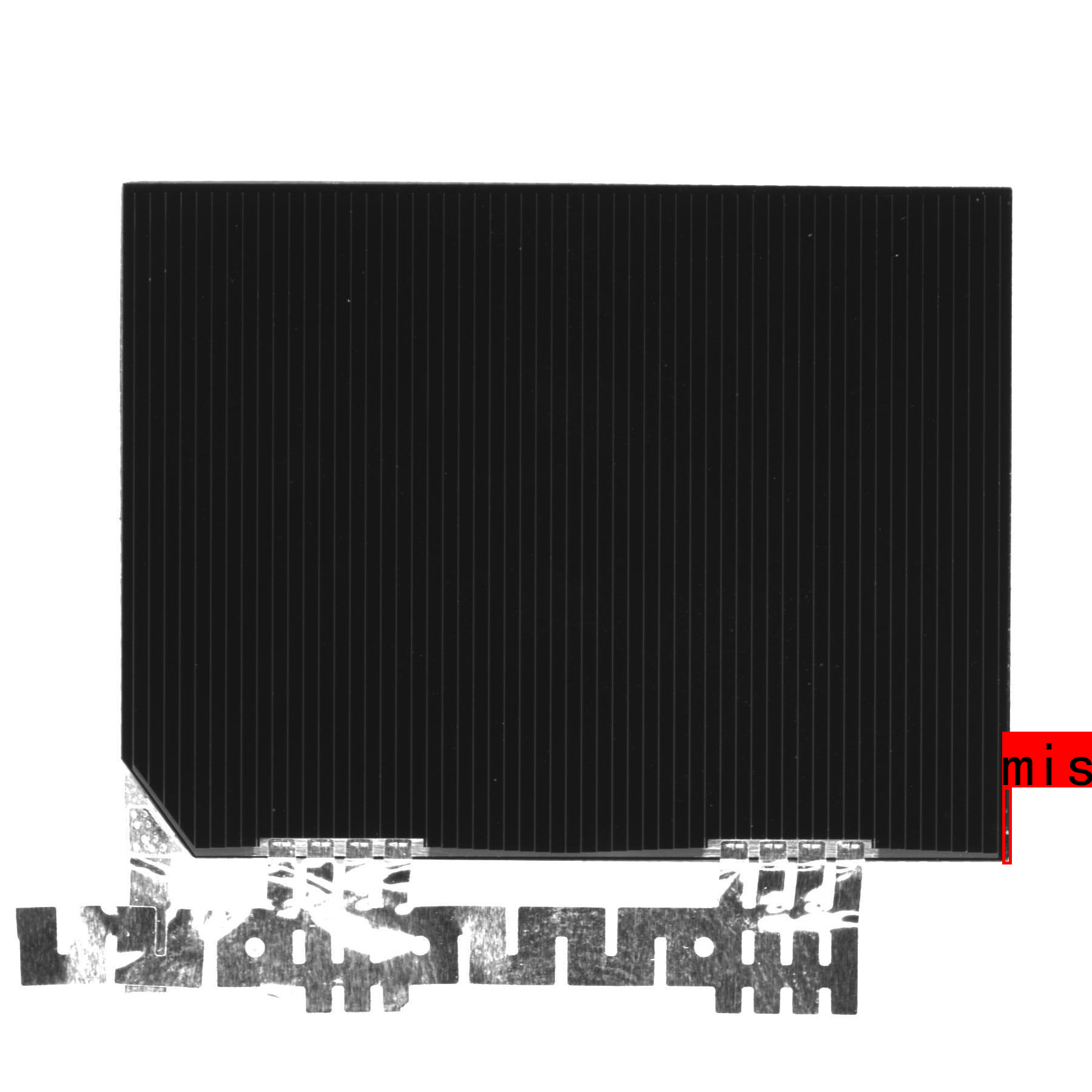

Supplement: S1 Dataset — (ZIP) [file pone.0304819.s001.zip › 00428mismatch_updown.png]

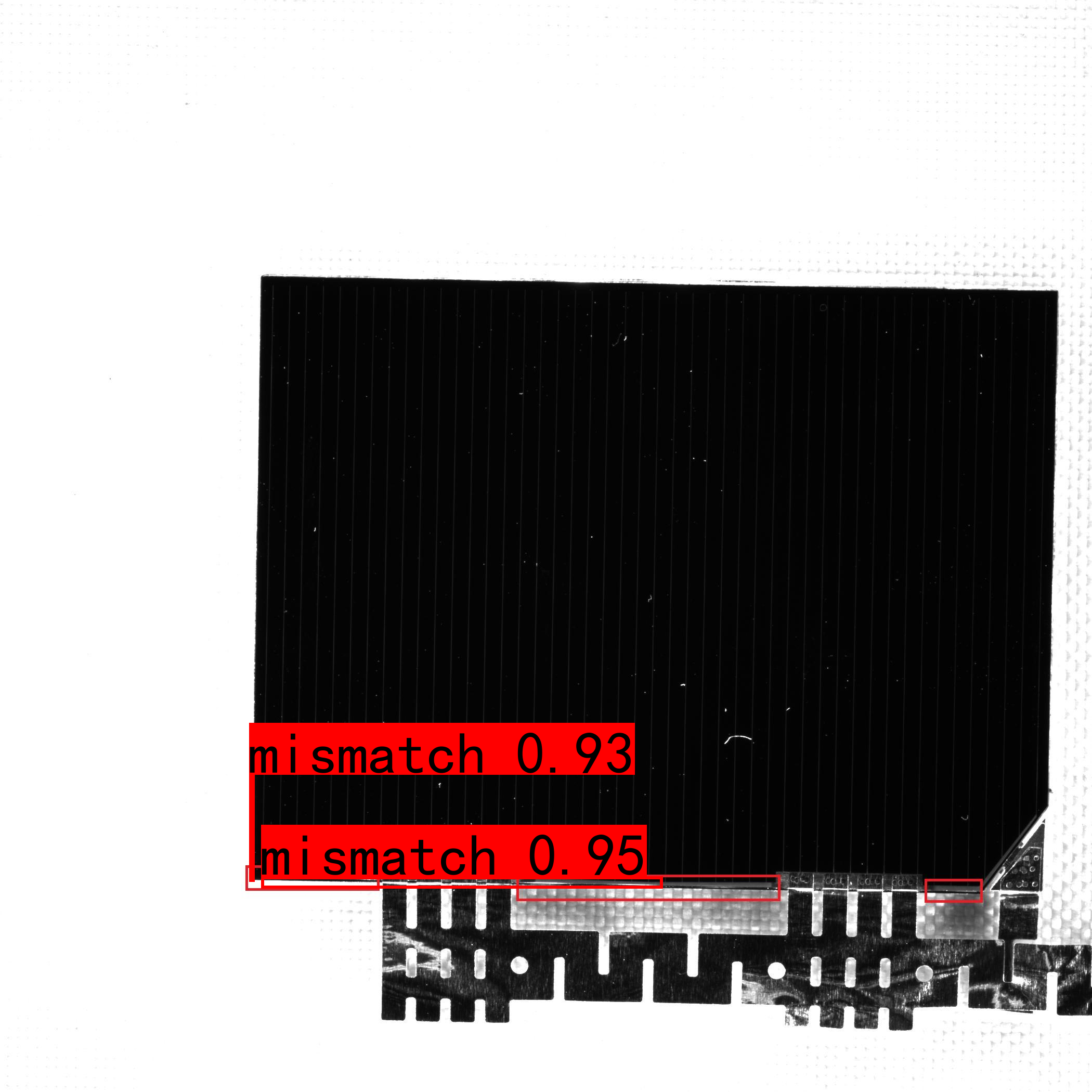

Supplement: S1 Dataset — (ZIP) [file pone.0304819.s001.zip › 00429mismatch_updown.png]

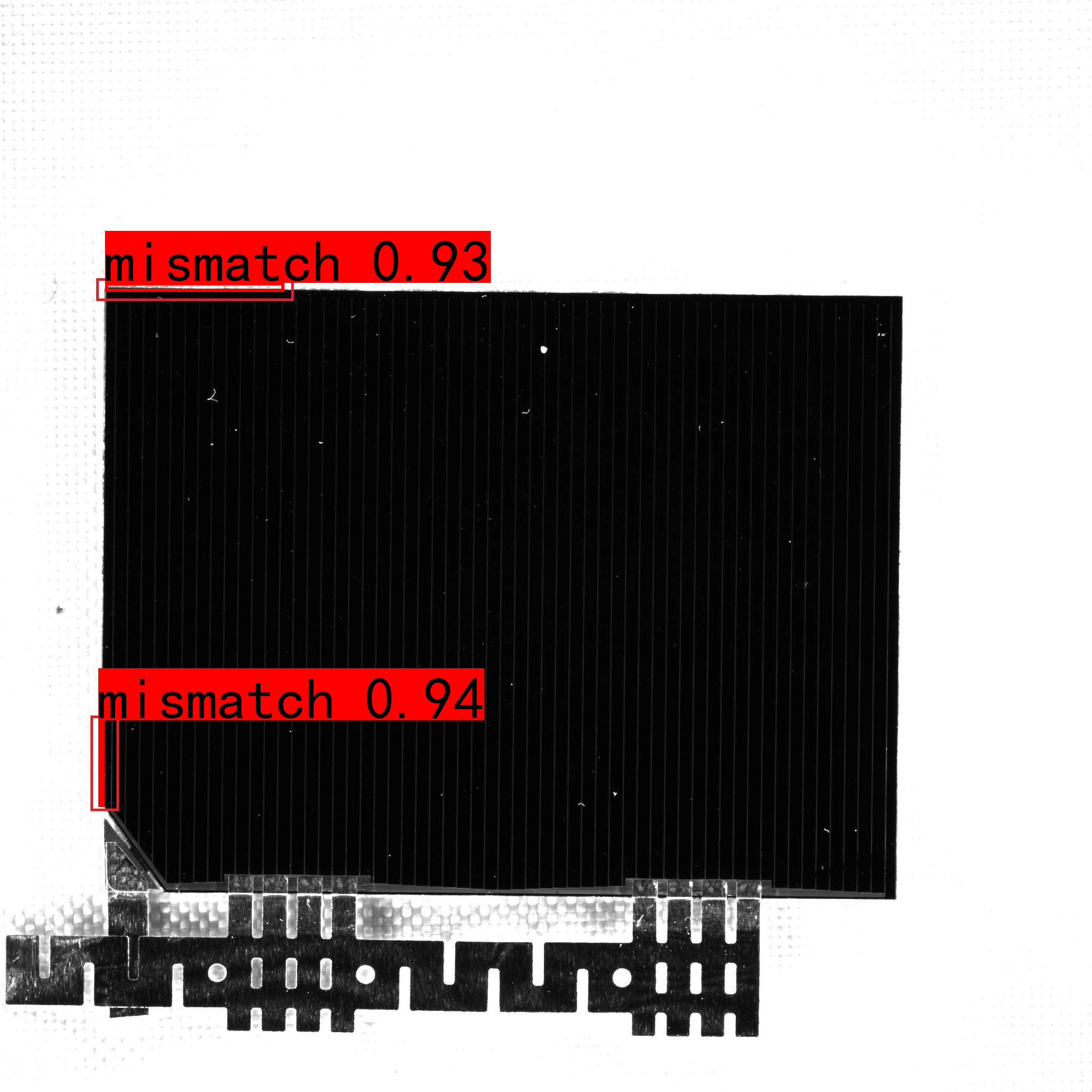

Supplement: S1 Dataset — (ZIP) [file pone.0304819.s001.zip › 00450mismatch_updown.png]

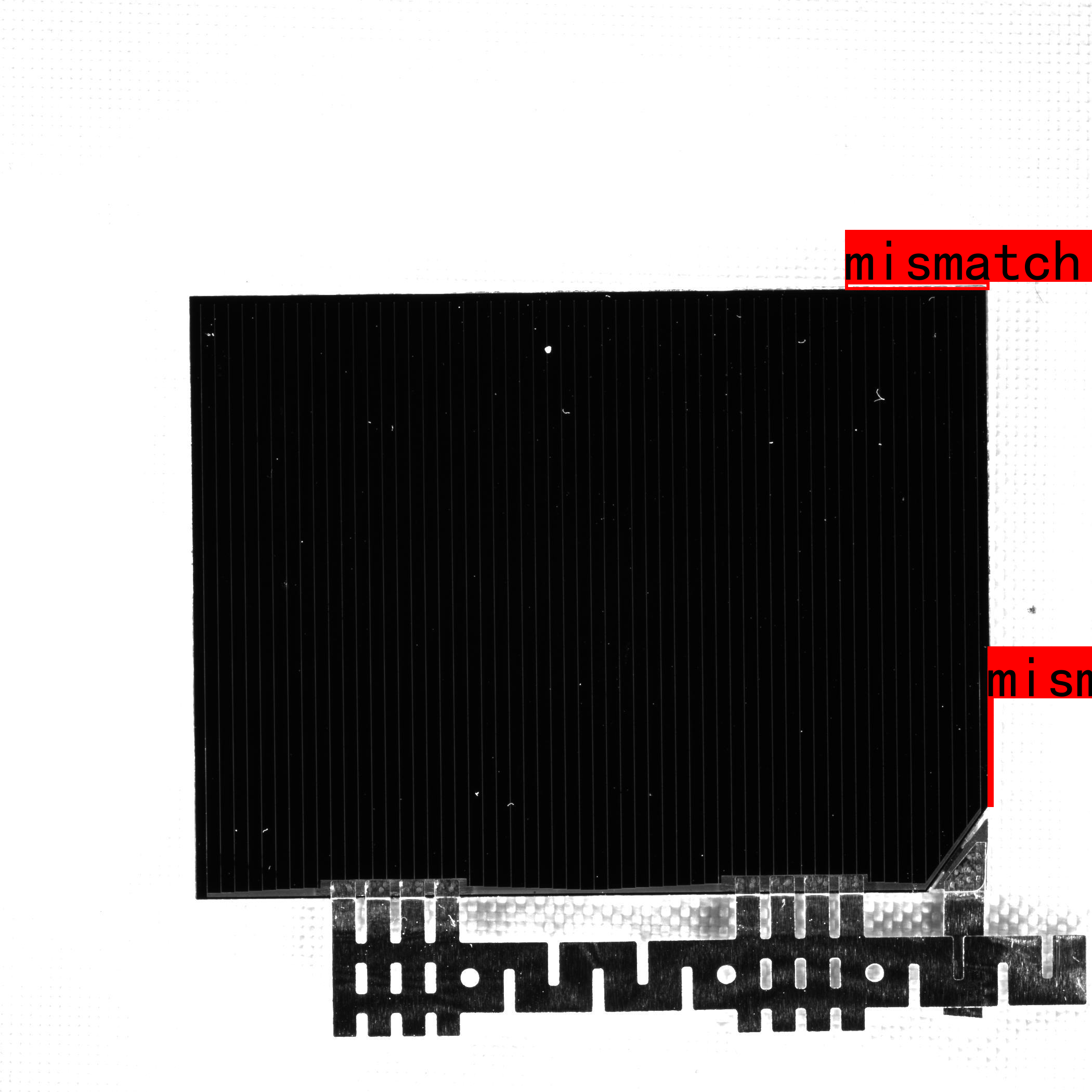

Supplement: S1 Dataset — (ZIP) [file pone.0304819.s001.zip › 00451mismatch_updown.png]

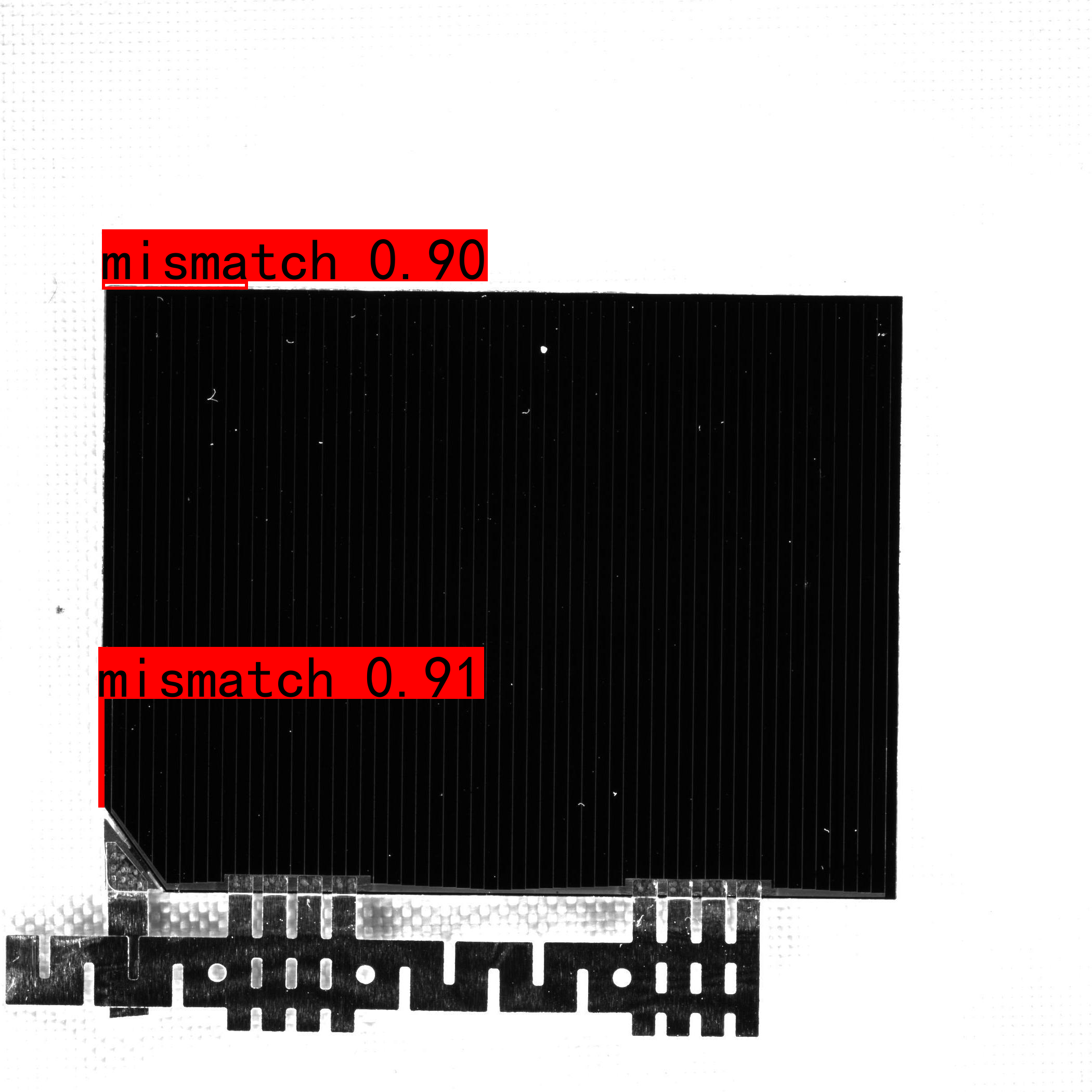

Supplement: S1 Dataset — (ZIP) [file pone.0304819.s001.zip › 00452mismatch_updown.png]

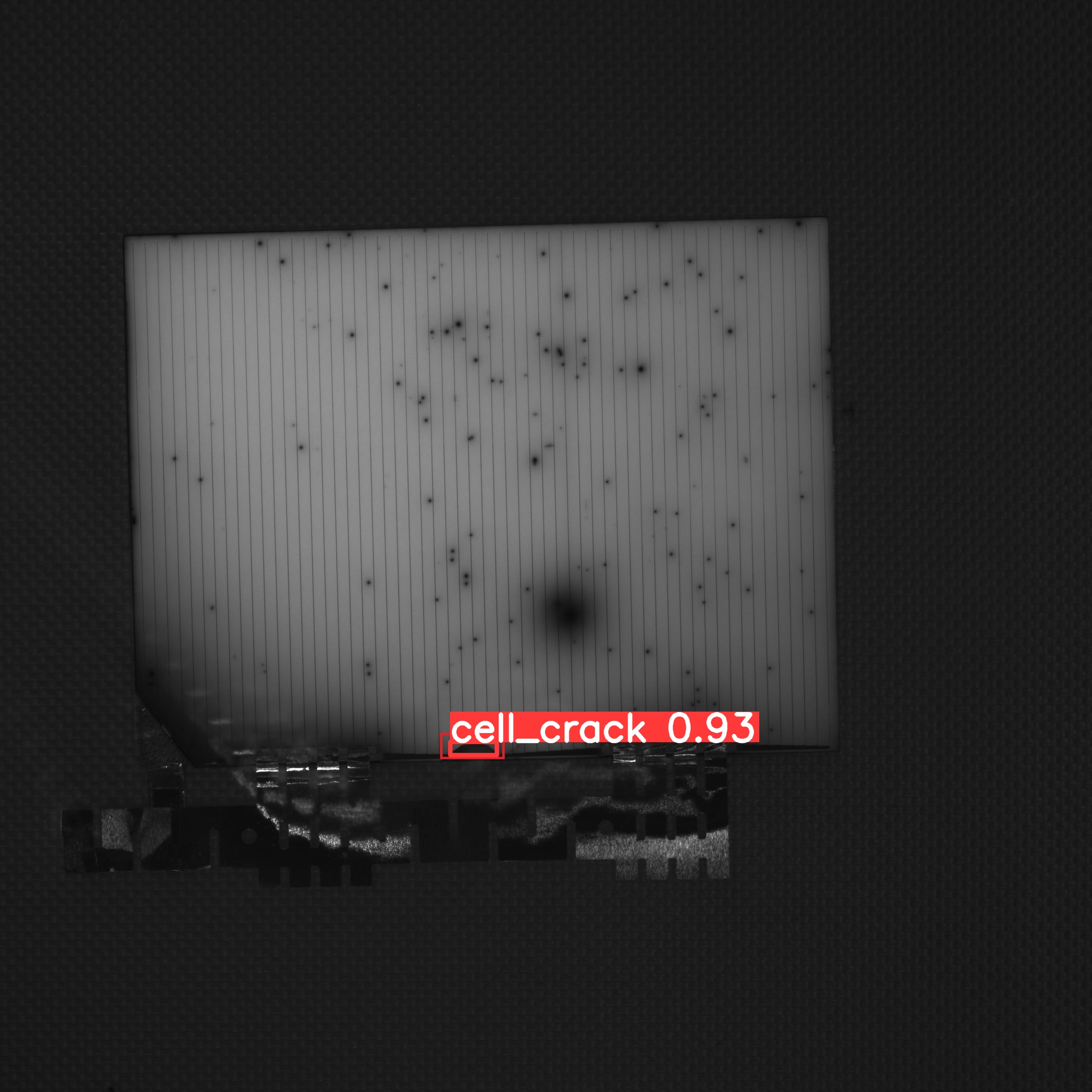

Supplement: S1 Dataset — (ZIP) [file pone.0304819.s001.zip › 0920.jpg]

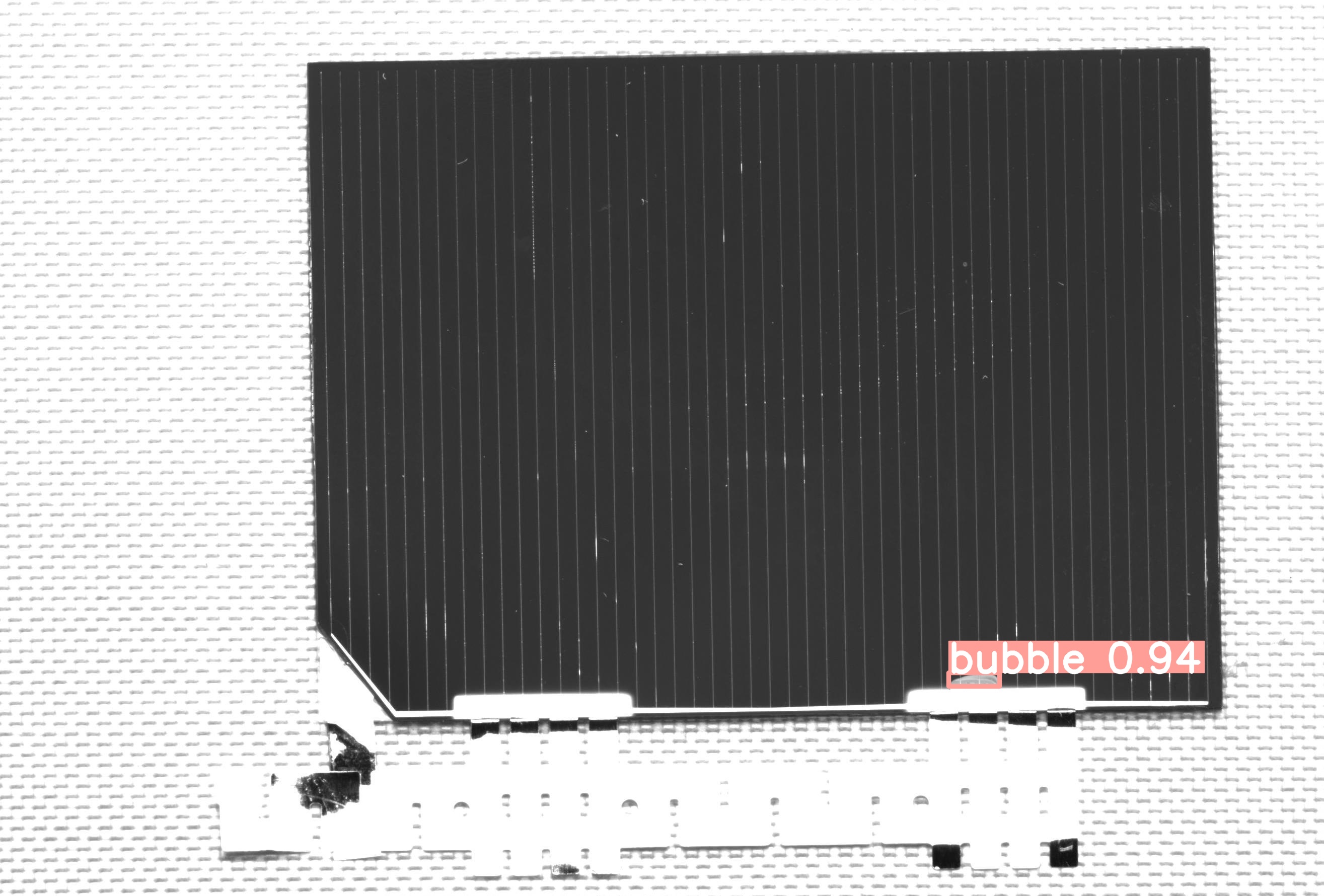

Supplement: S1 Dataset — (ZIP) [file pone.0304819.s001.zip › 1089.jpg]

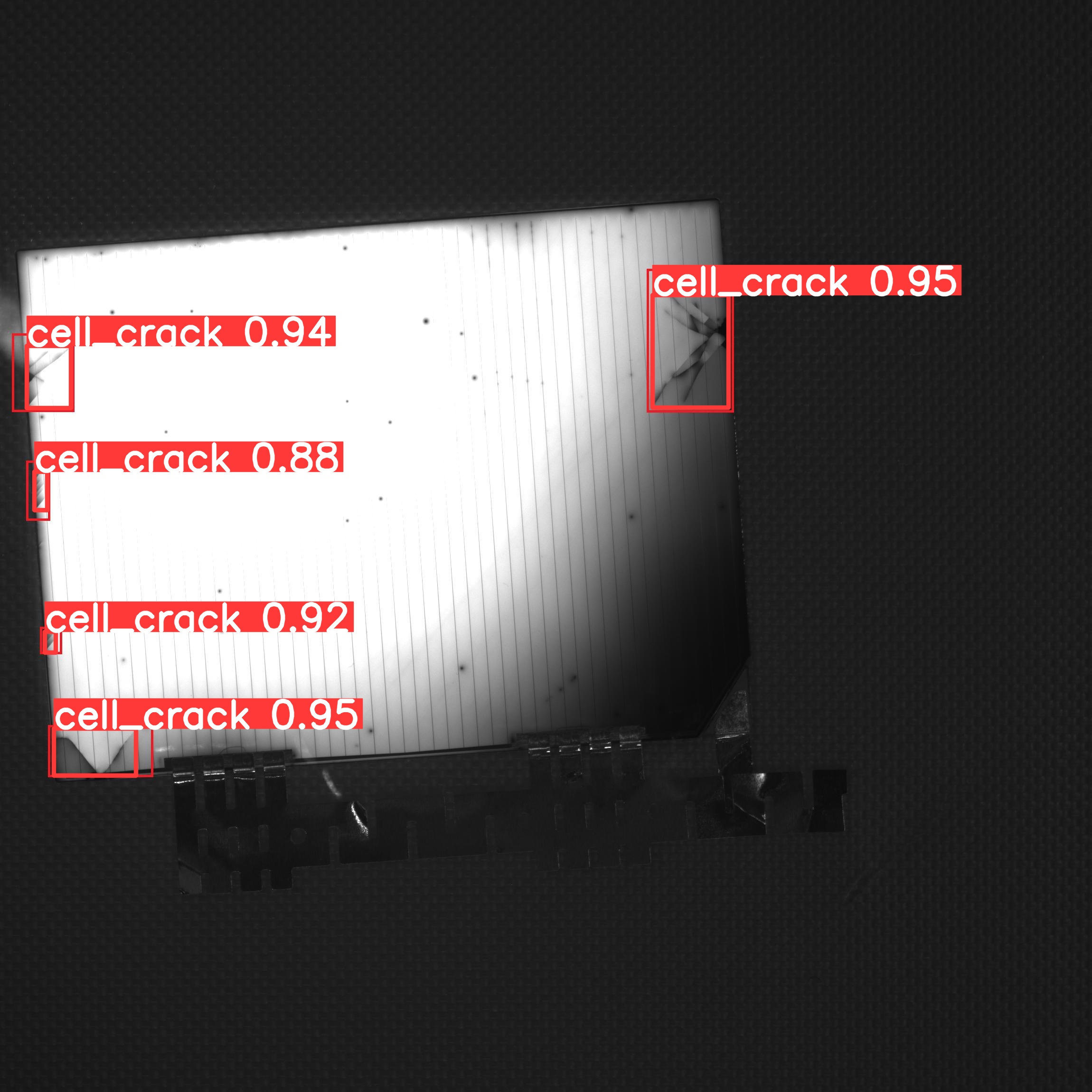

Supplement: S1 Dataset — (ZIP) [file pone.0304819.s001.zip › 1360.jpg]

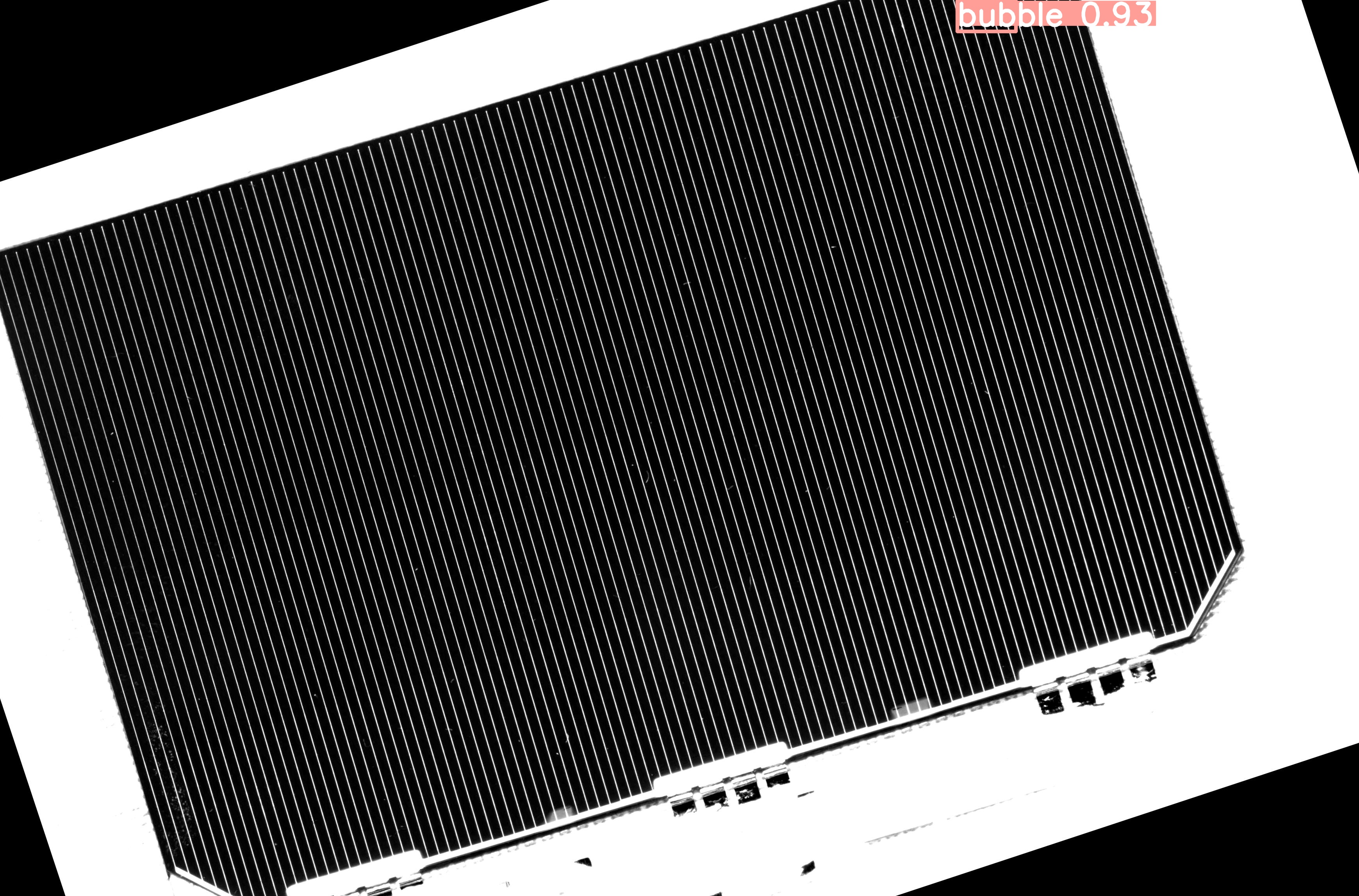

Supplement: S1 Dataset — (ZIP) [file pone.0304819.s001.zip › 2305.jpg]

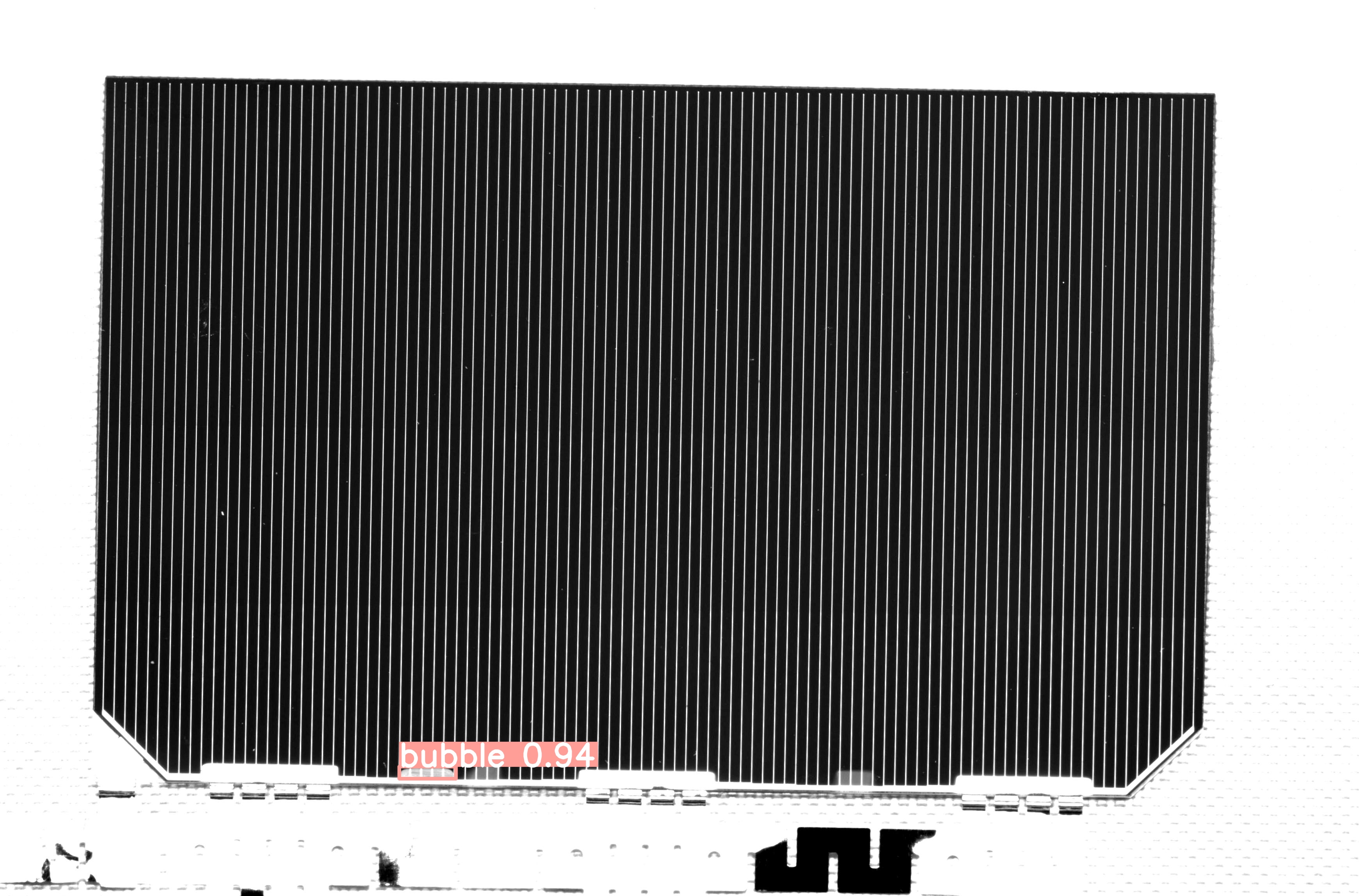

Supplement: S1 Dataset — (ZIP) [file pone.0304819.s001.zip › 3307.jpg]

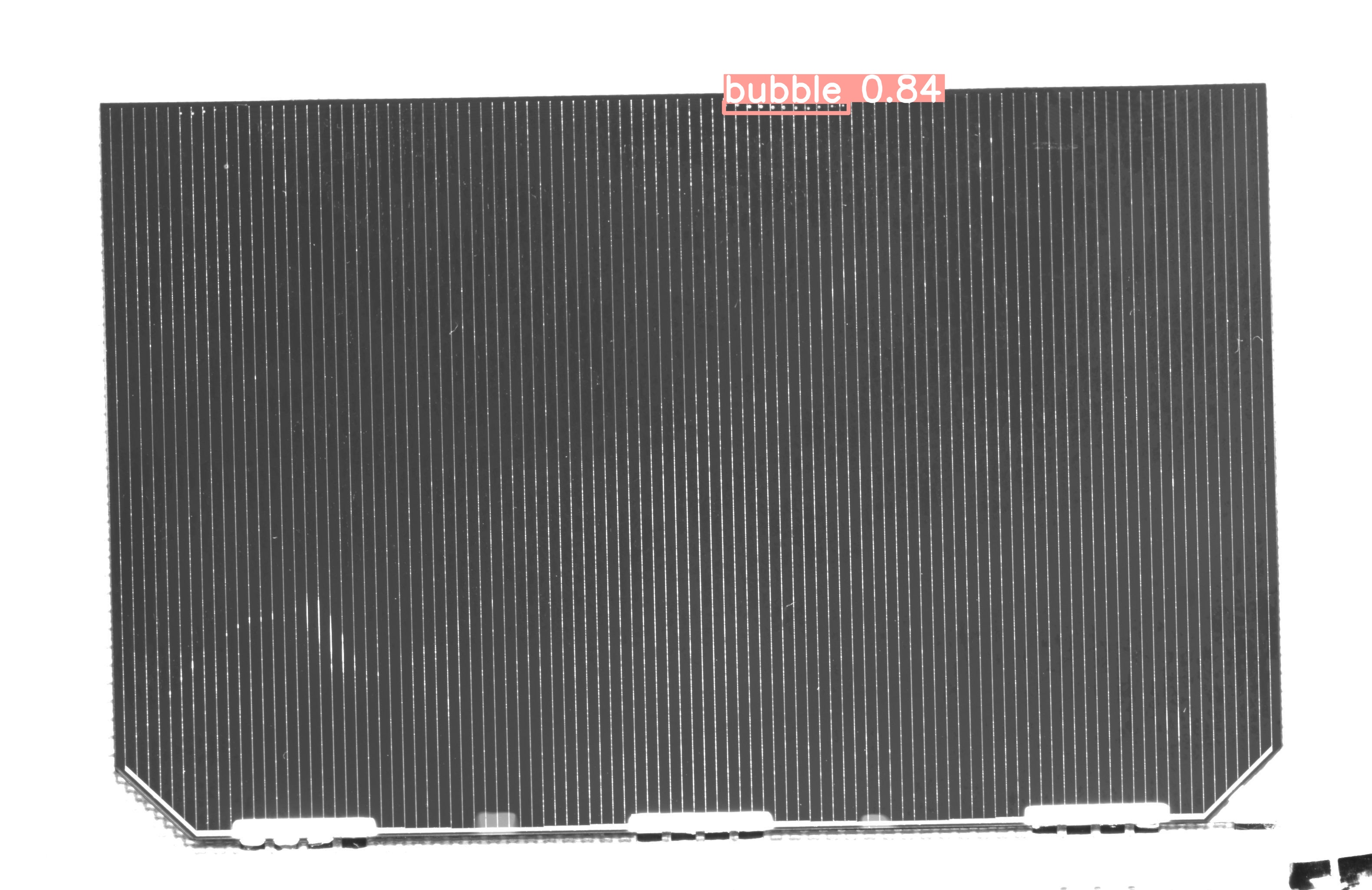

Supplement: S1 Dataset — (ZIP) [file pone.0304819.s001.zip › 3668.jpg]

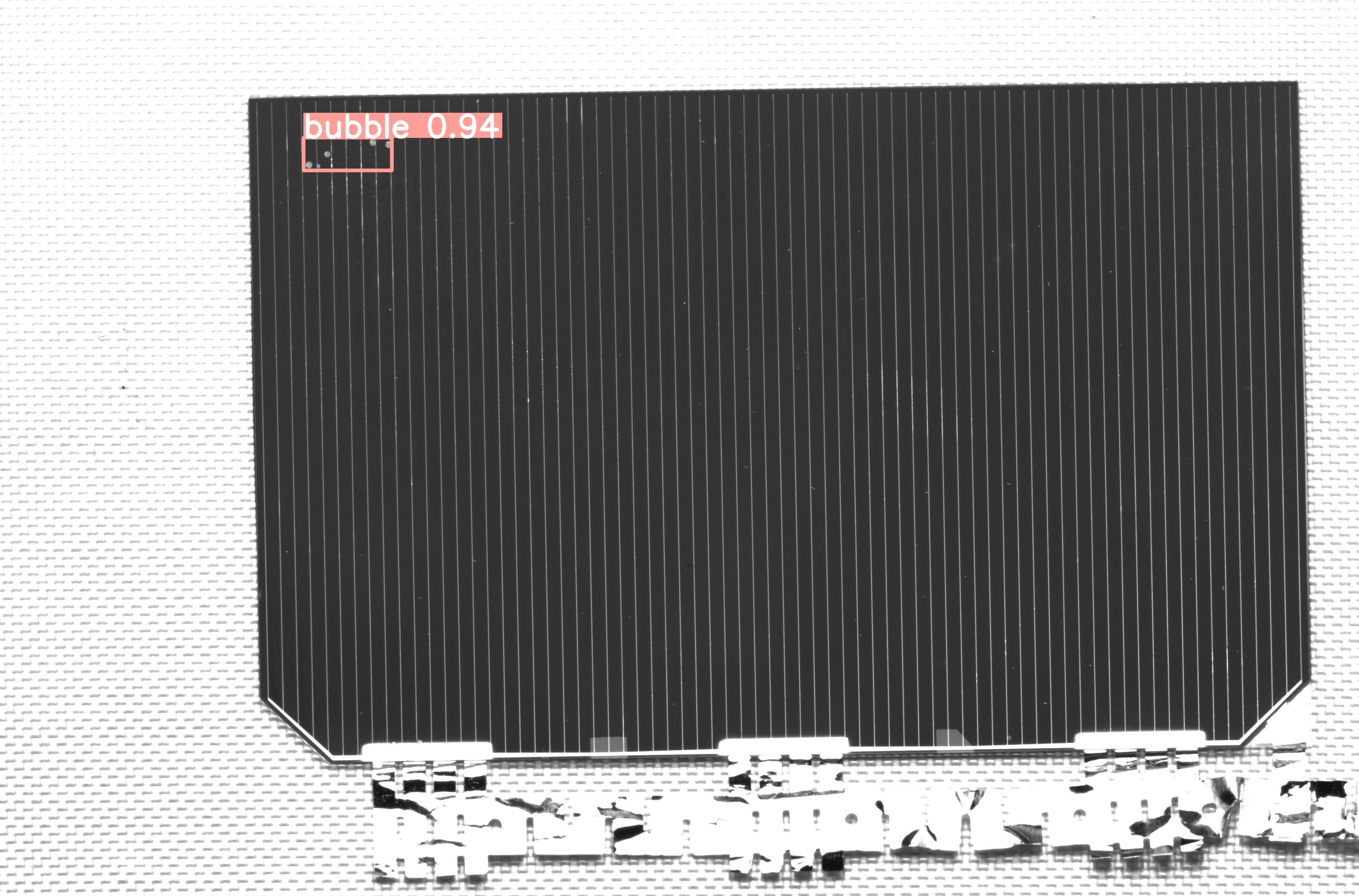

Supplement: S1 Dataset — (ZIP) [file pone.0304819.s001.zip › 3987.jpg]

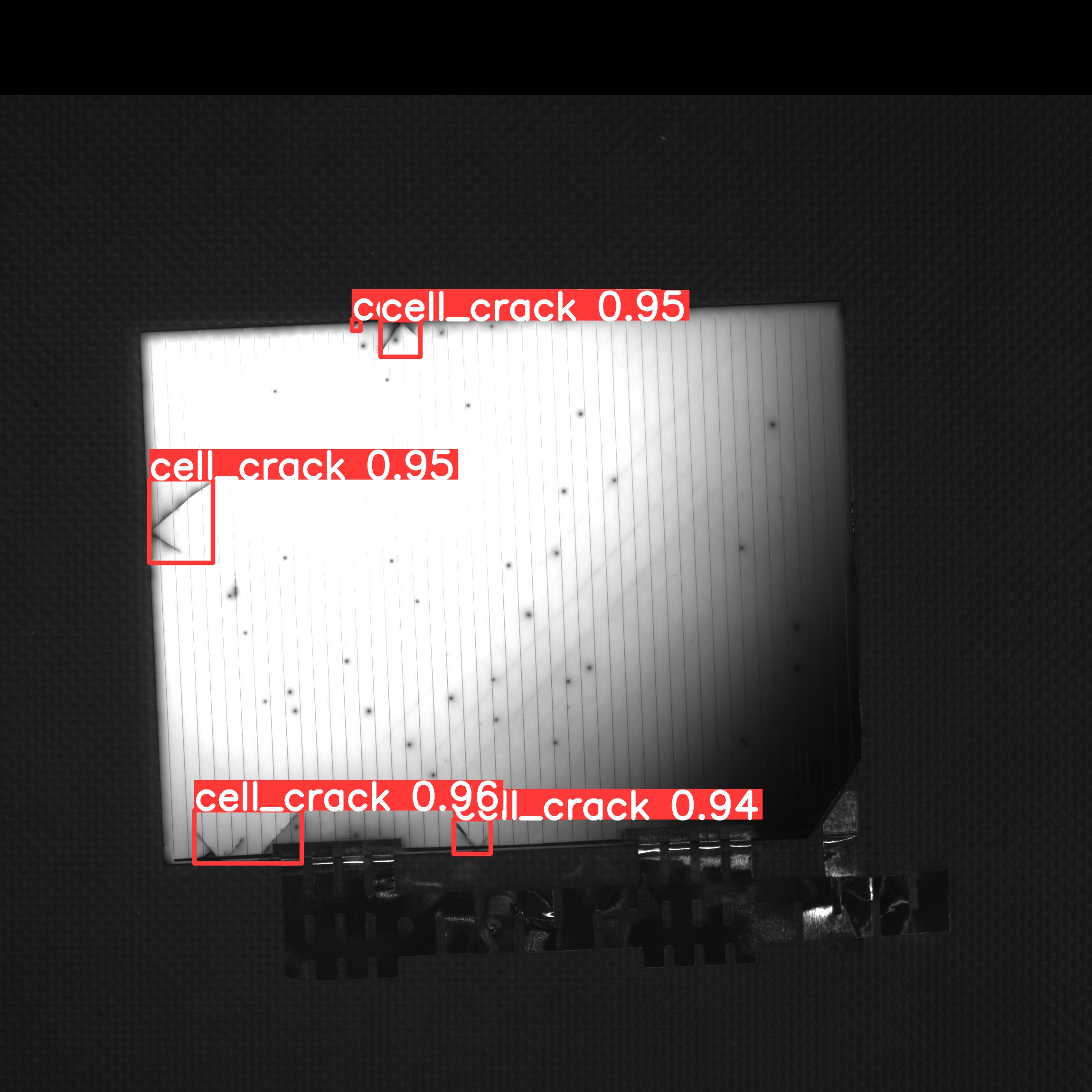

Supplement: S1 Dataset — (ZIP) [file pone.0304819.s001.zip › 4142.jpg]

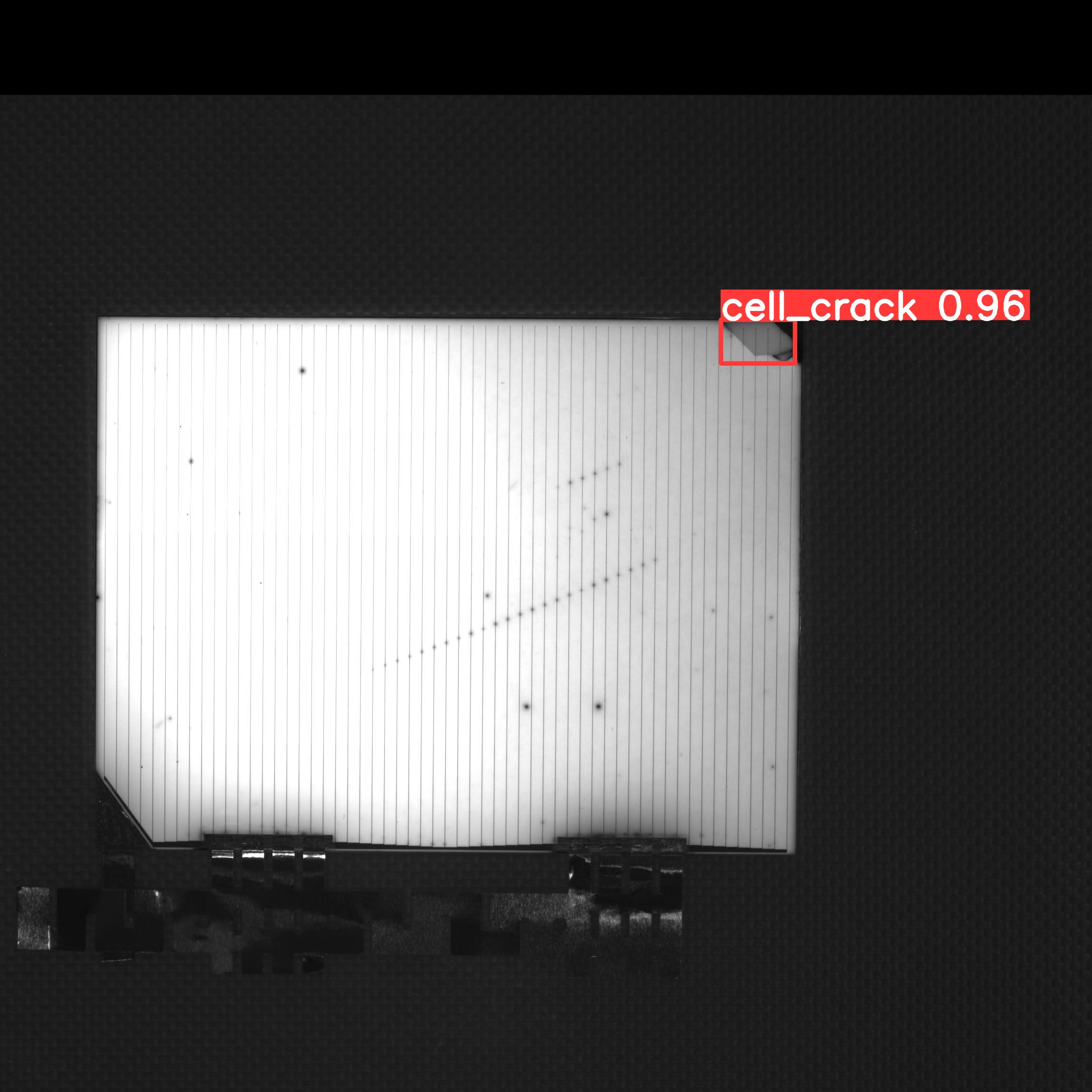

Supplement: S1 Dataset — (ZIP) [file pone.0304819.s001.zip › 4248.jpg]

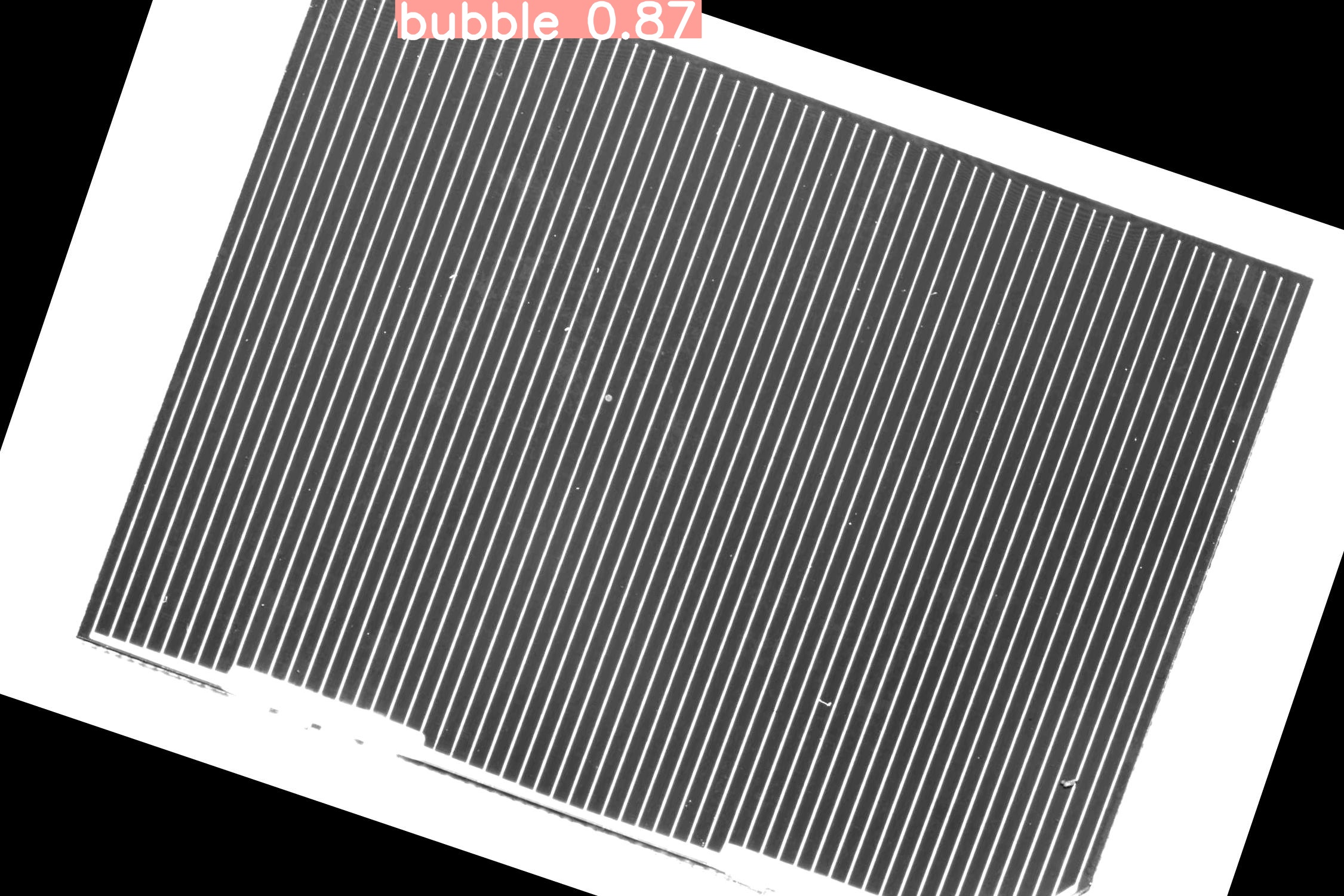

Supplement: S1 Dataset — (ZIP) [file pone.0304819.s001.zip › 4653.jpg]

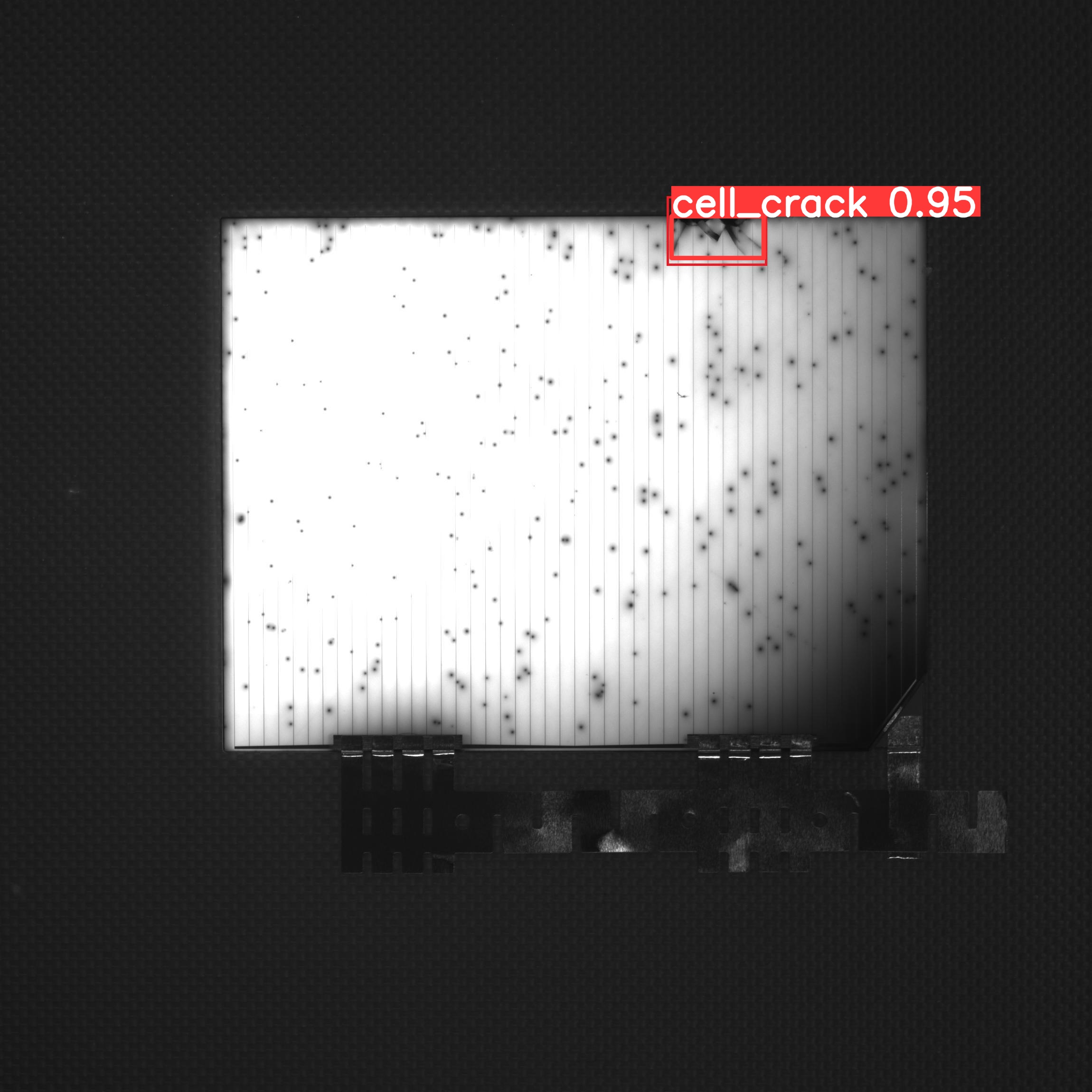

Supplement: S1 Dataset — (ZIP) [file pone.0304819.s001.zip › 4750.jpg]

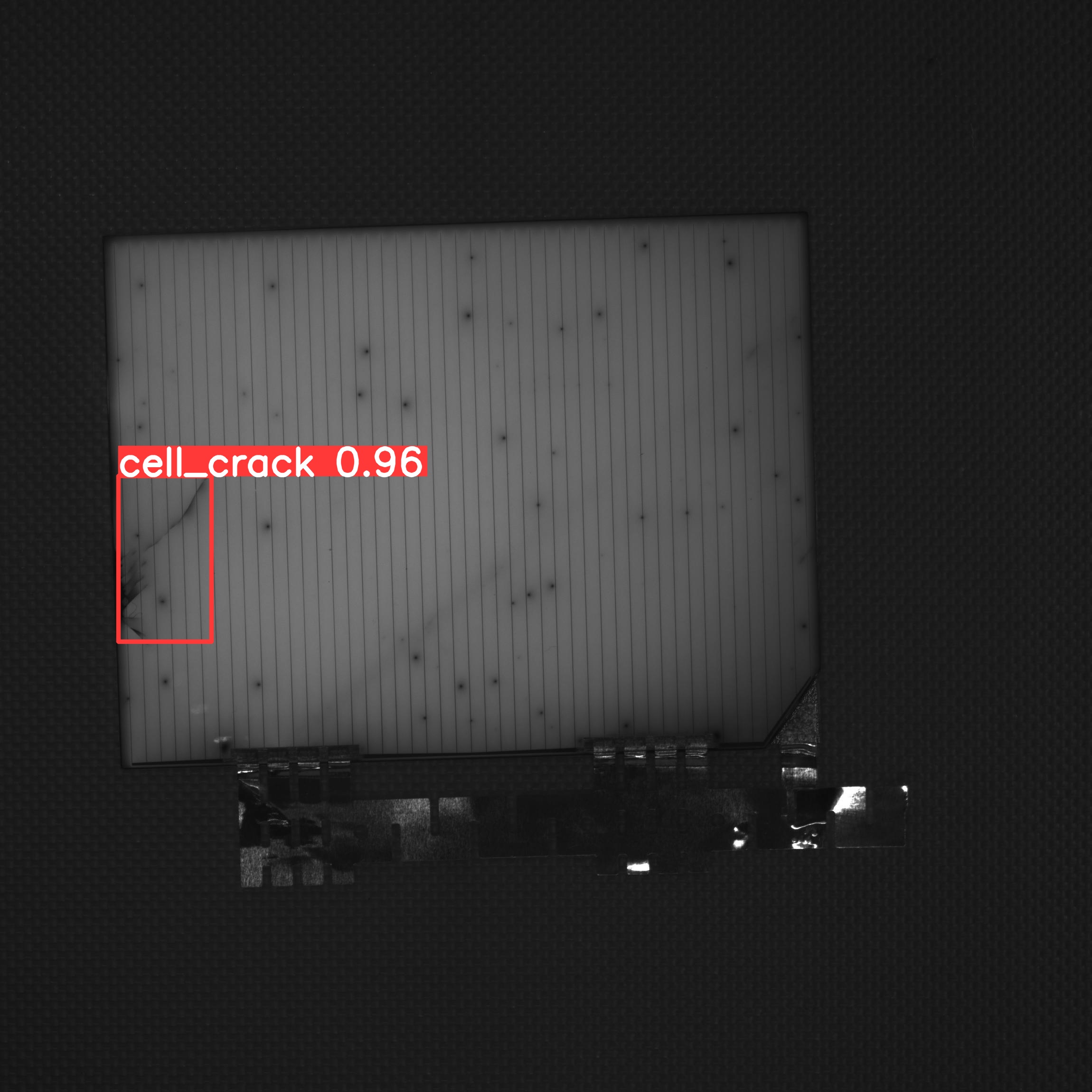

Supplement: S1 Dataset — (ZIP) [file pone.0304819.s001.zip › 5074.jpg]

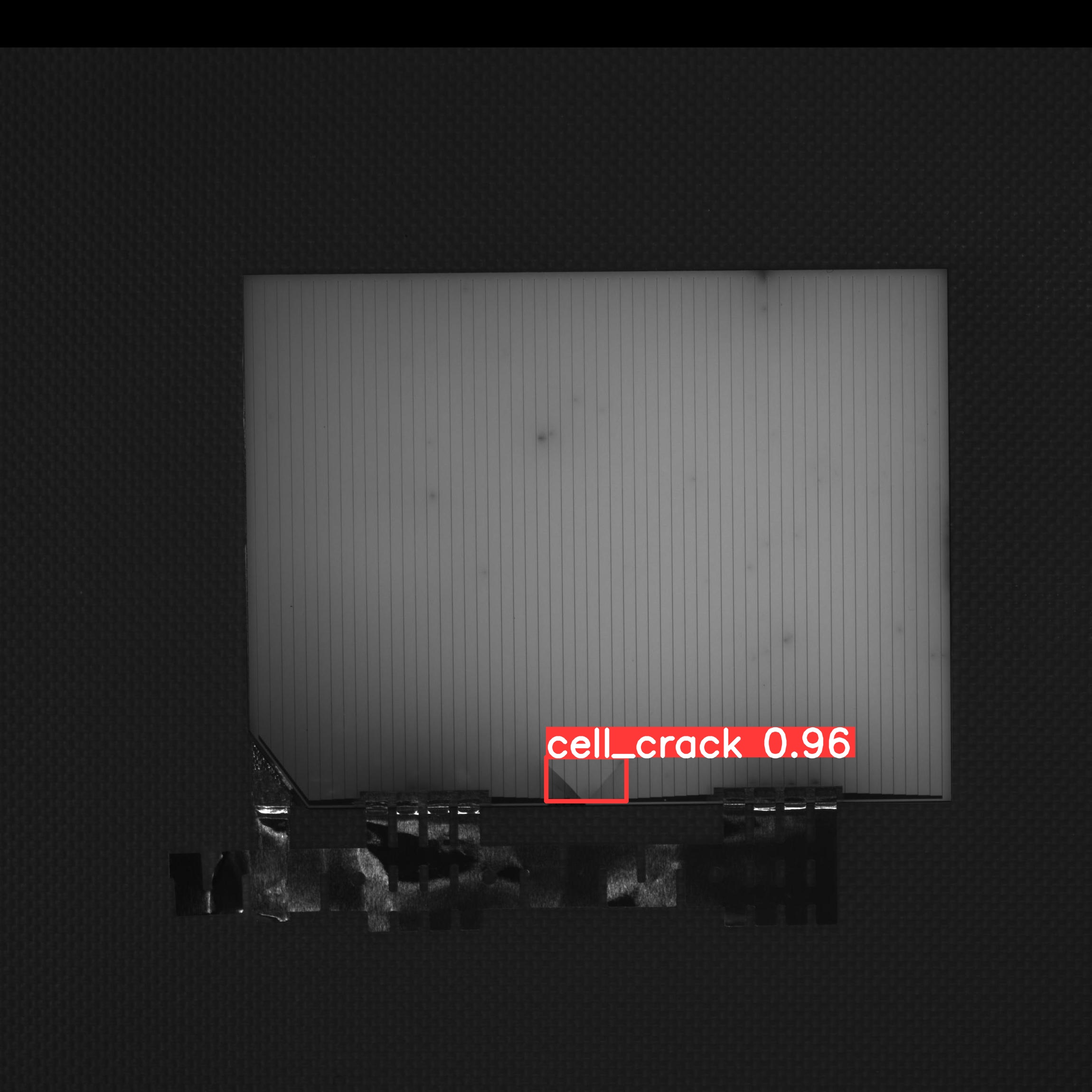

Supplement: S1 Dataset — (ZIP) [file pone.0304819.s001.zip › 5142.jpg]

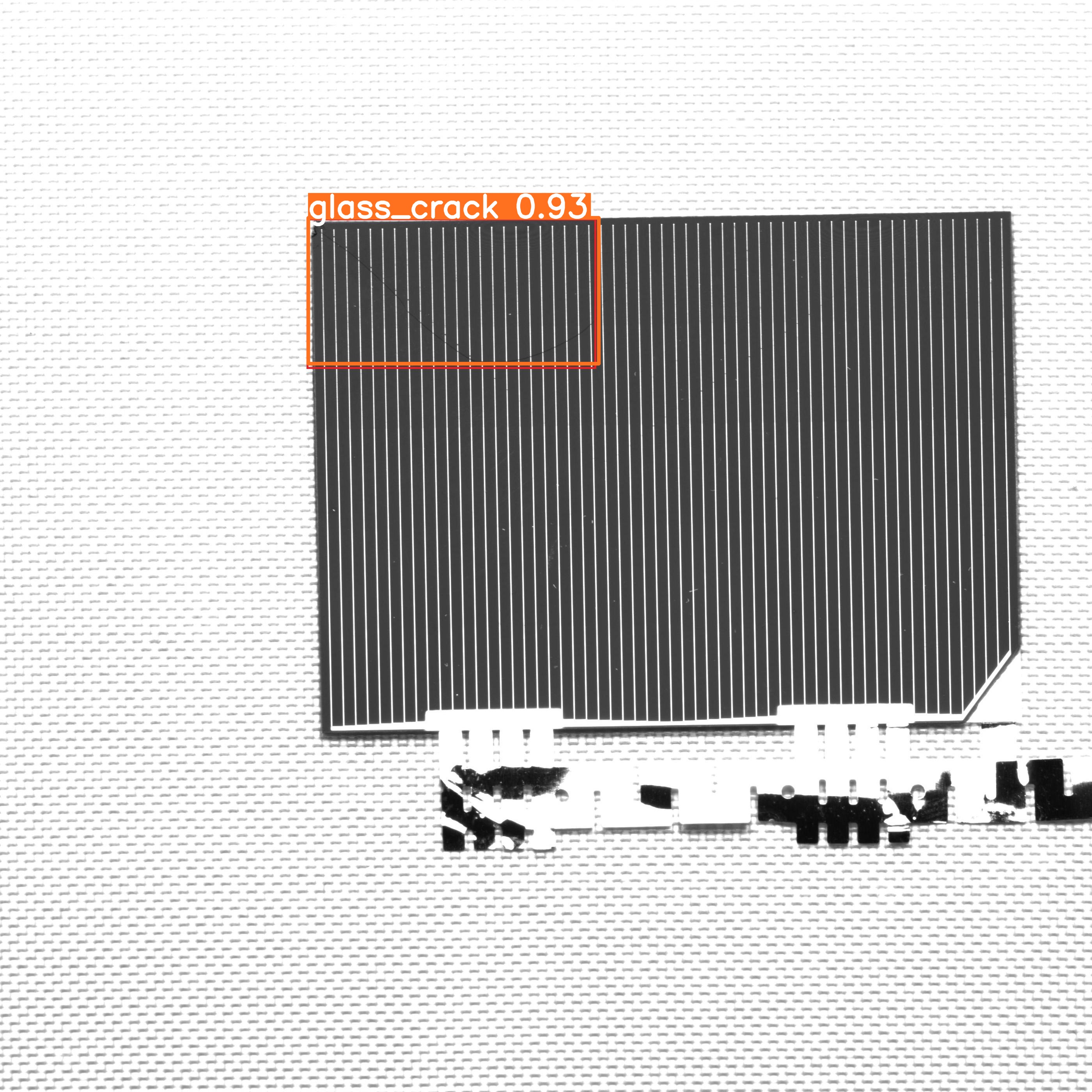

Supplement: S1 Dataset — (ZIP) [file pone.0304819.s001.zip › 5230.jpg]

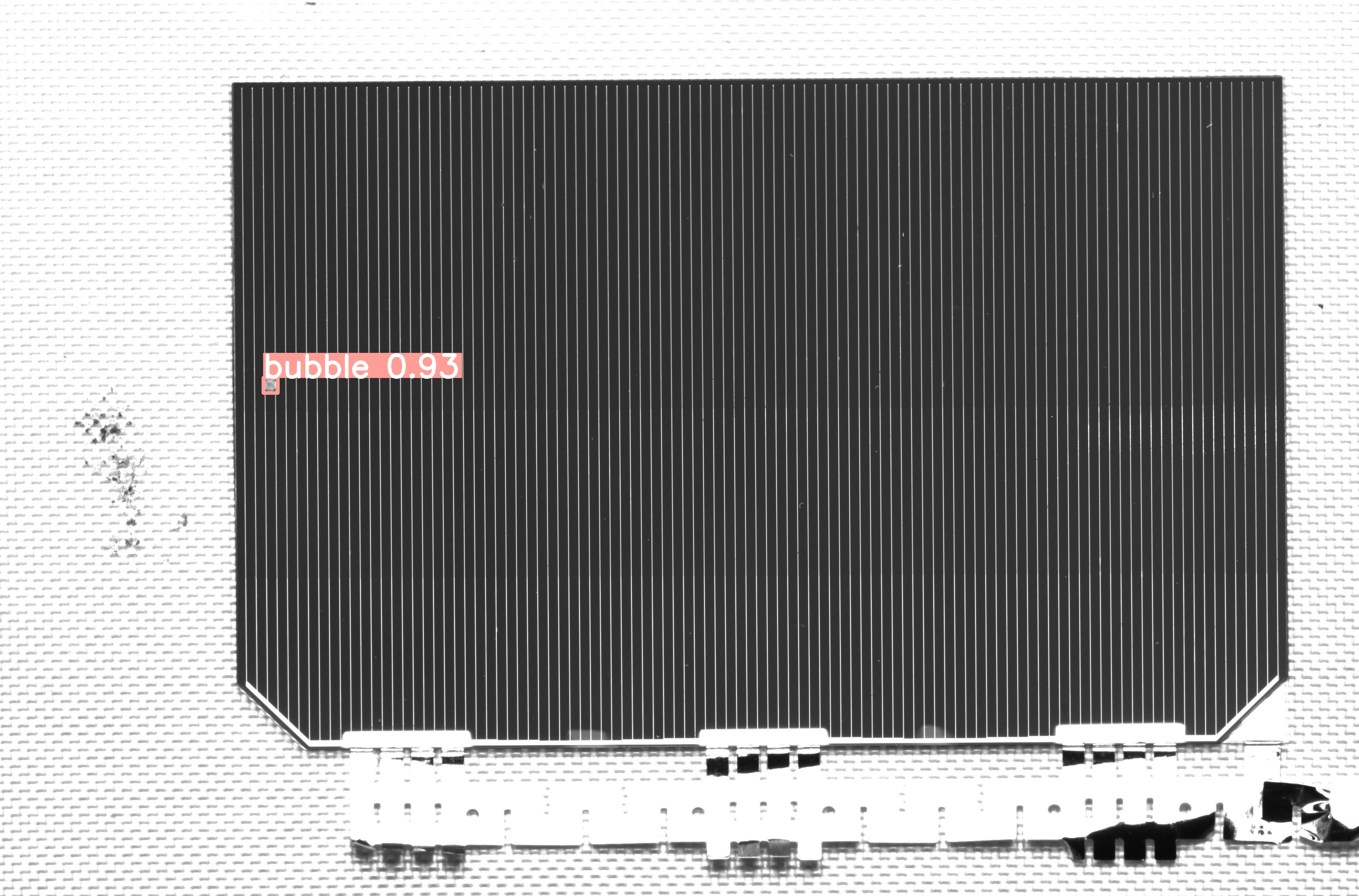

Supplement: S1 Dataset — (ZIP) [file pone.0304819.s001.zip › 5308.jpg]

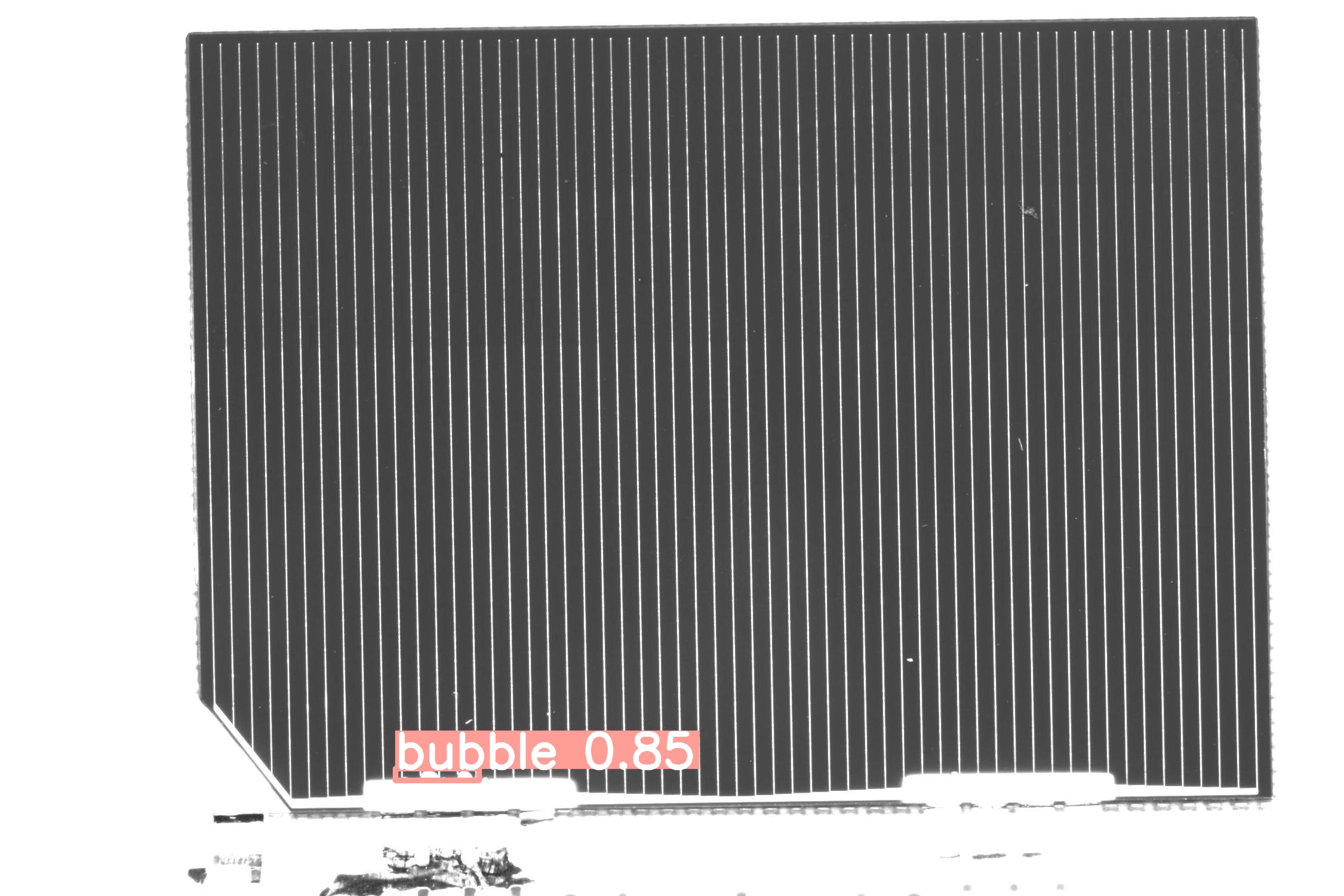

Supplement: S1 Dataset — (ZIP) [file pone.0304819.s001.zip › 5553.jpg]

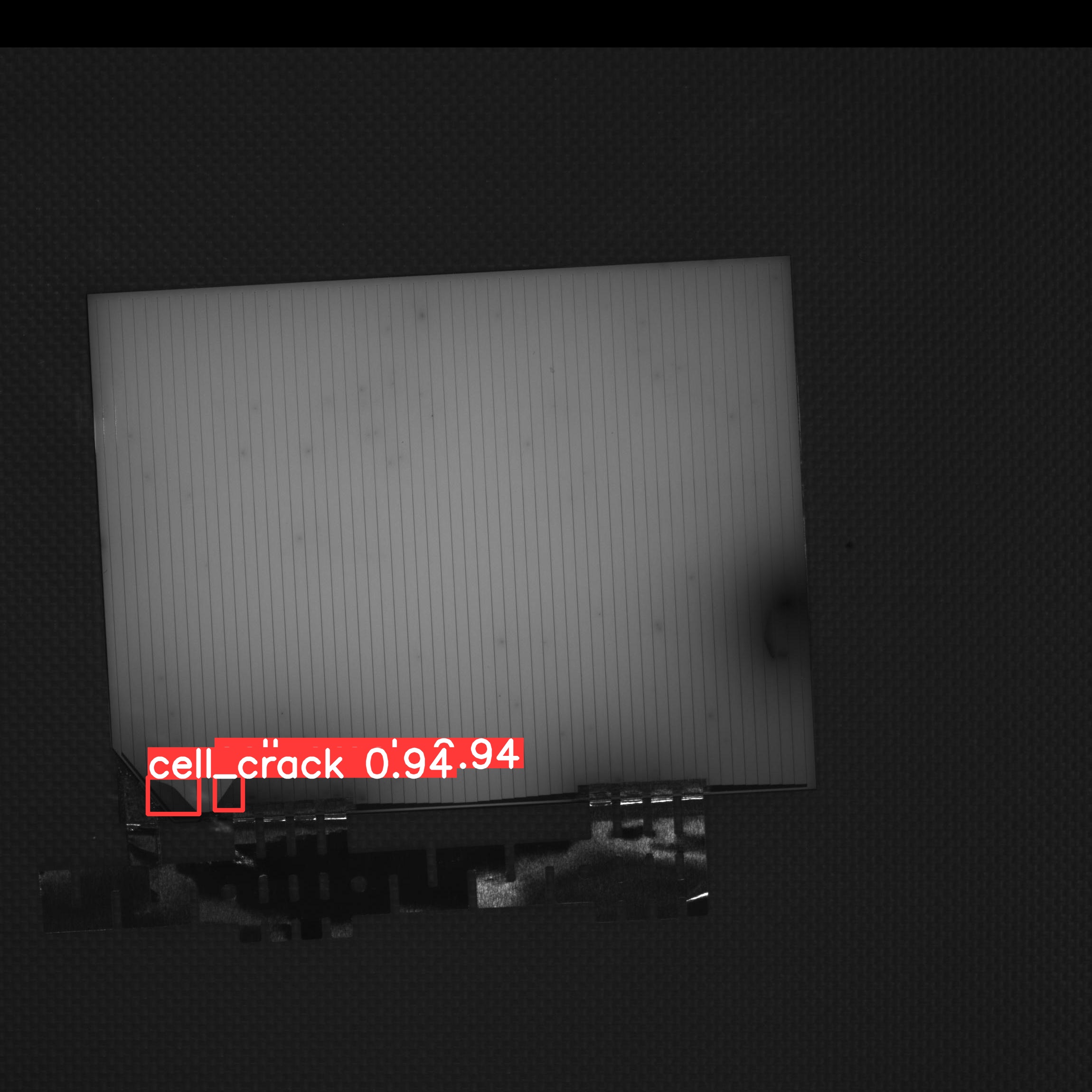

Supplement: S1 Dataset — (ZIP) [file pone.0304819.s001.zip › 5610.jpg]

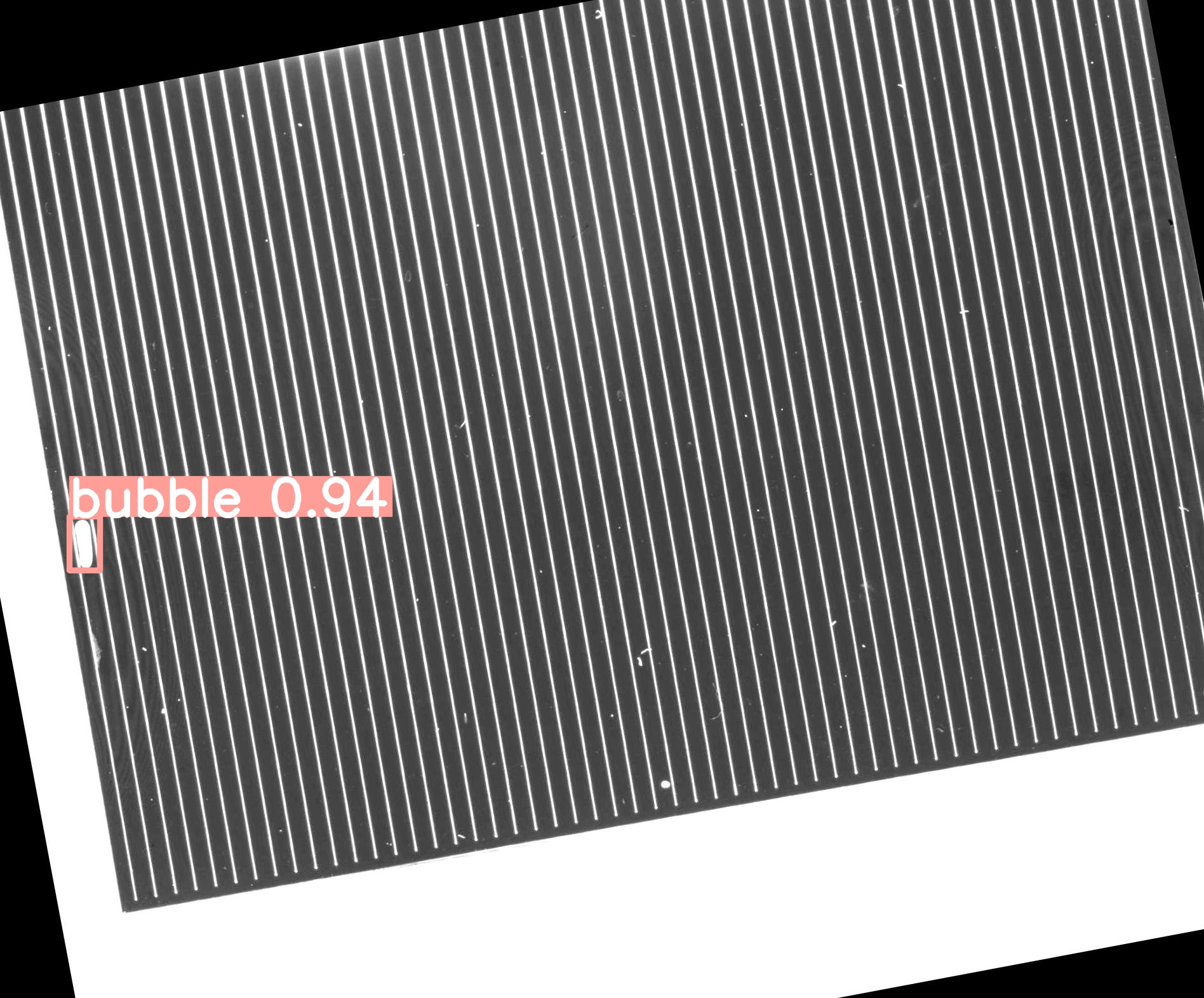

Supplement: S1 Dataset — (ZIP) [file pone.0304819.s001.zip › 5933.jpg]

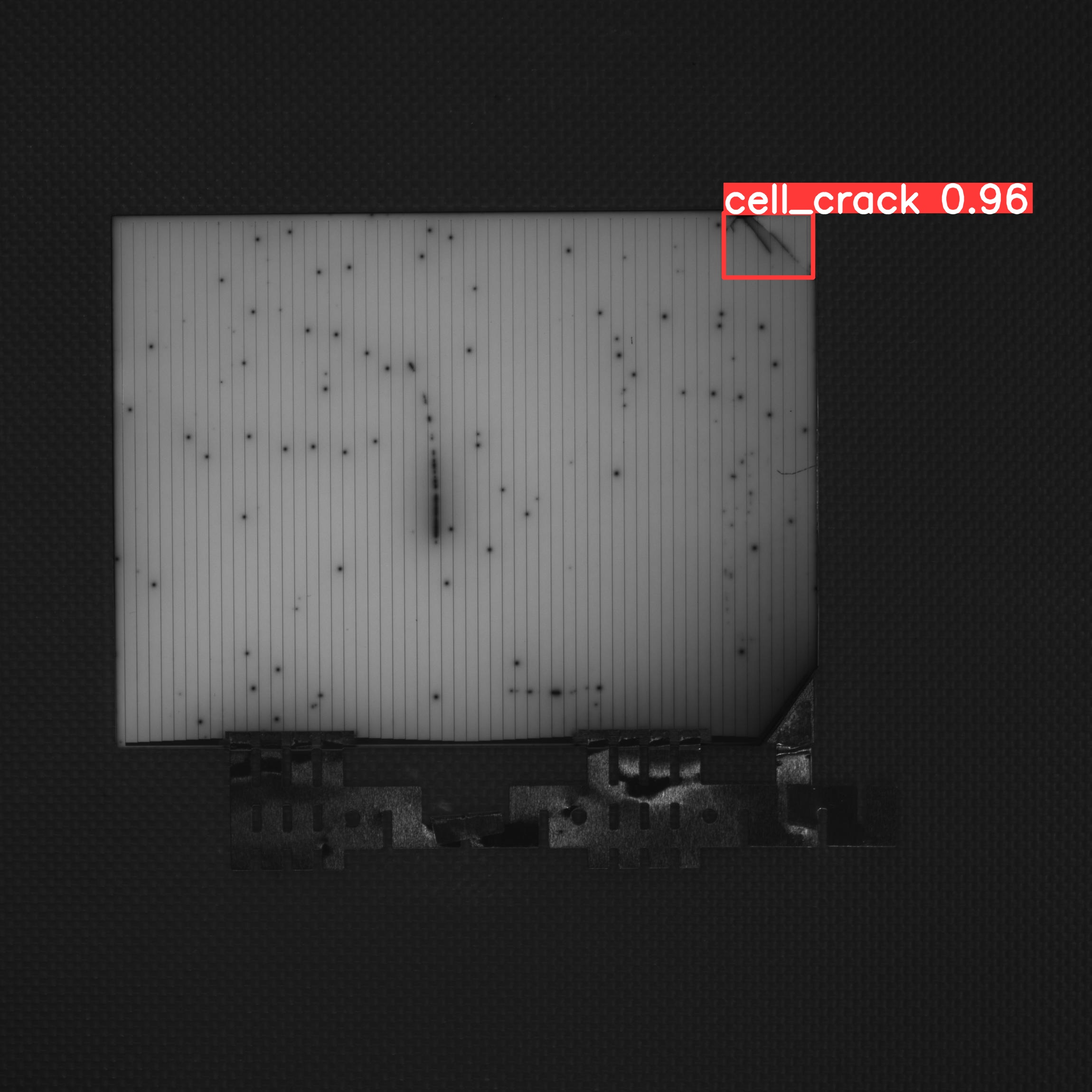

Supplement: S1 Dataset — (ZIP) [file pone.0304819.s001.zip › 5996.jpg]

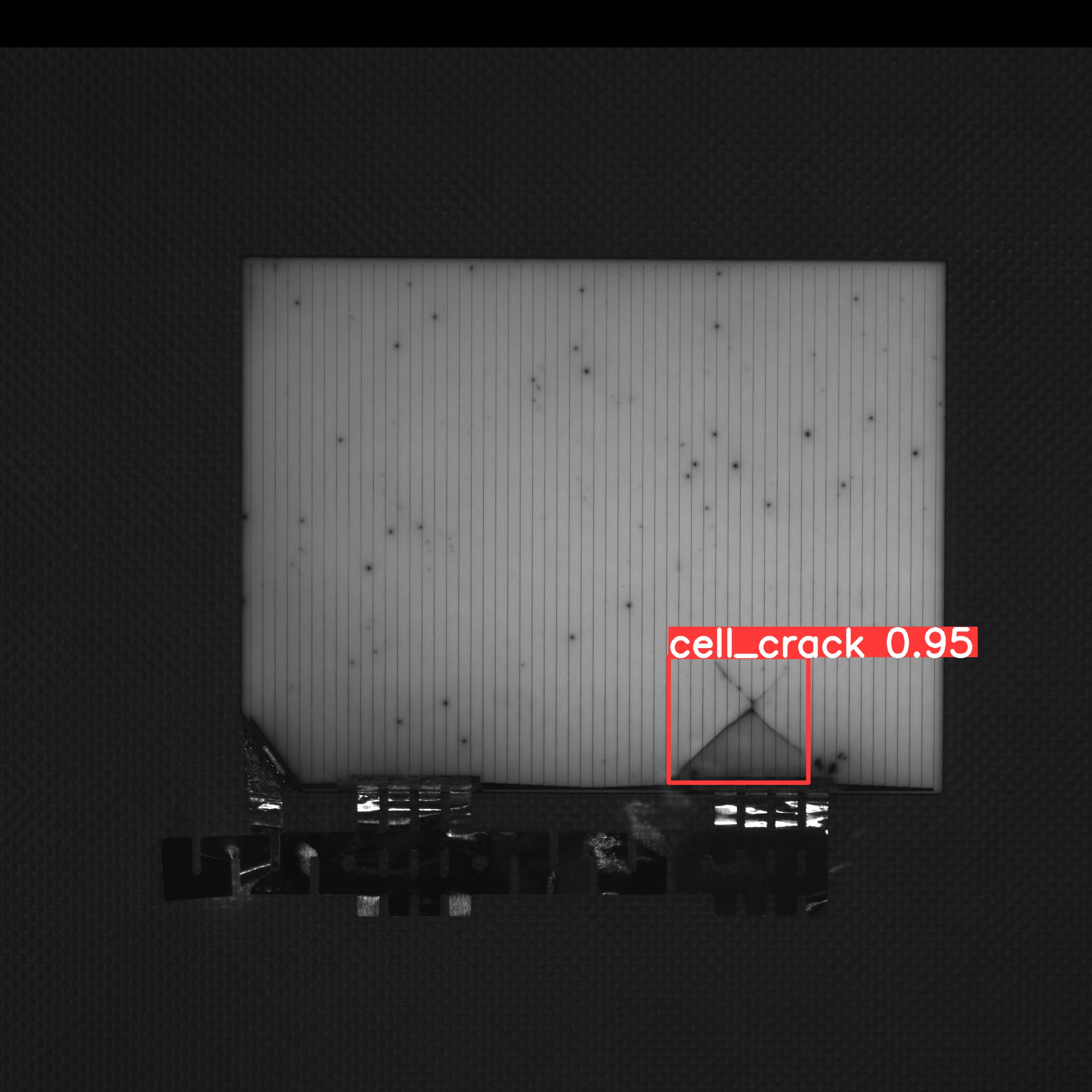

Supplement: S1 Dataset — (ZIP) [file pone.0304819.s001.zip › 6104.jpg]

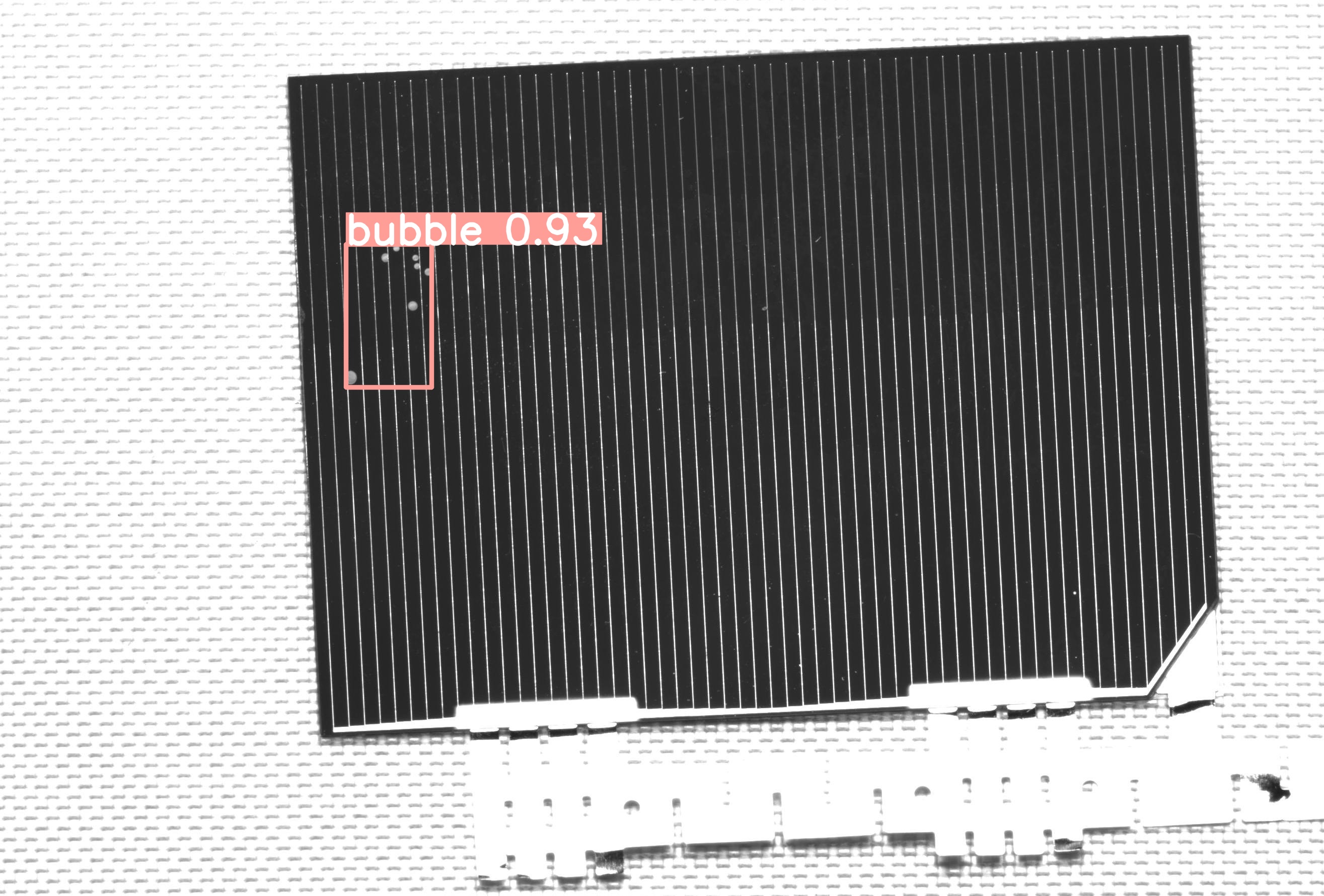

Supplement: S1 Dataset — (ZIP) [file pone.0304819.s001.zip › 6176.jpg]

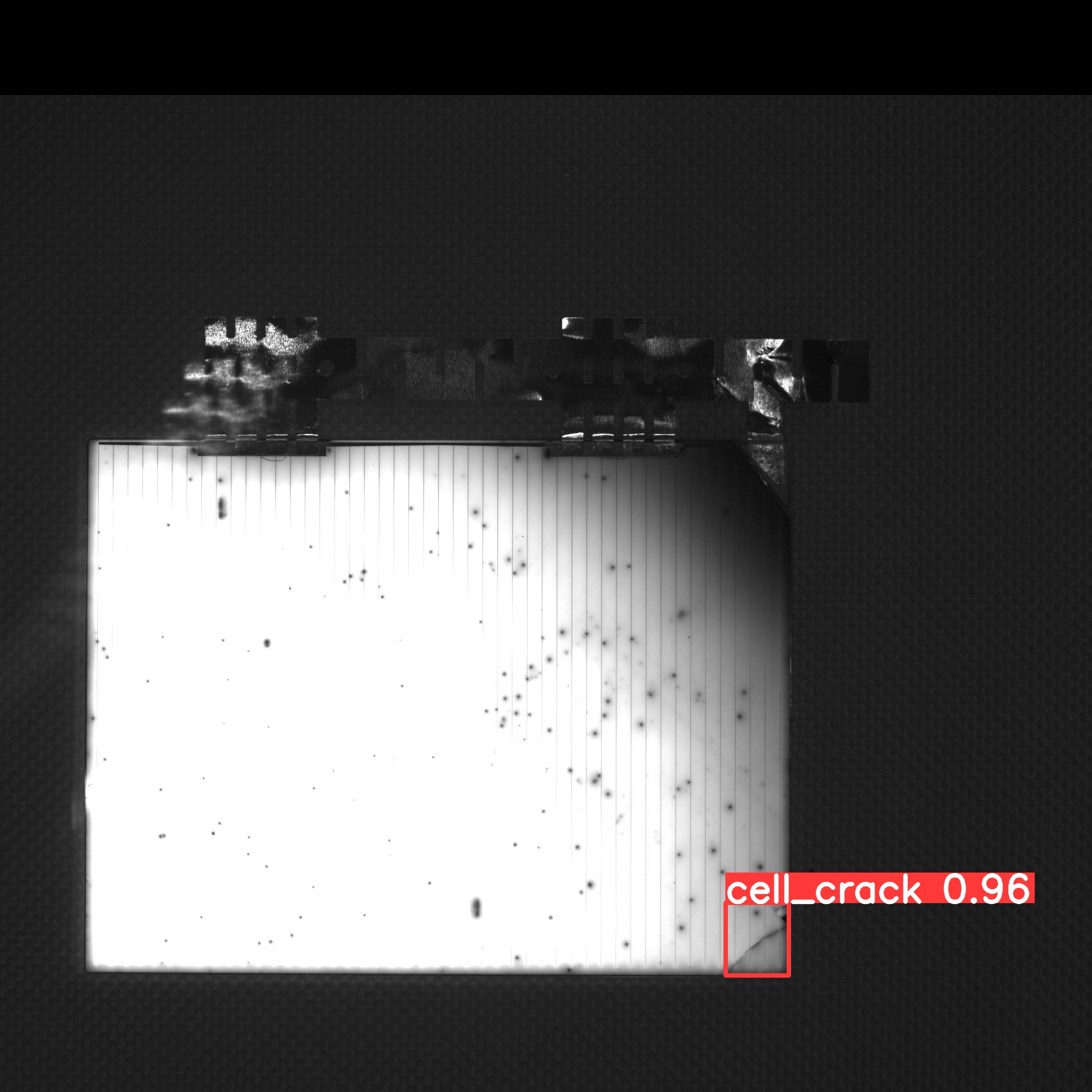

Supplement: S1 Dataset — (ZIP) [file pone.0304819.s001.zip › 6258.jpg]

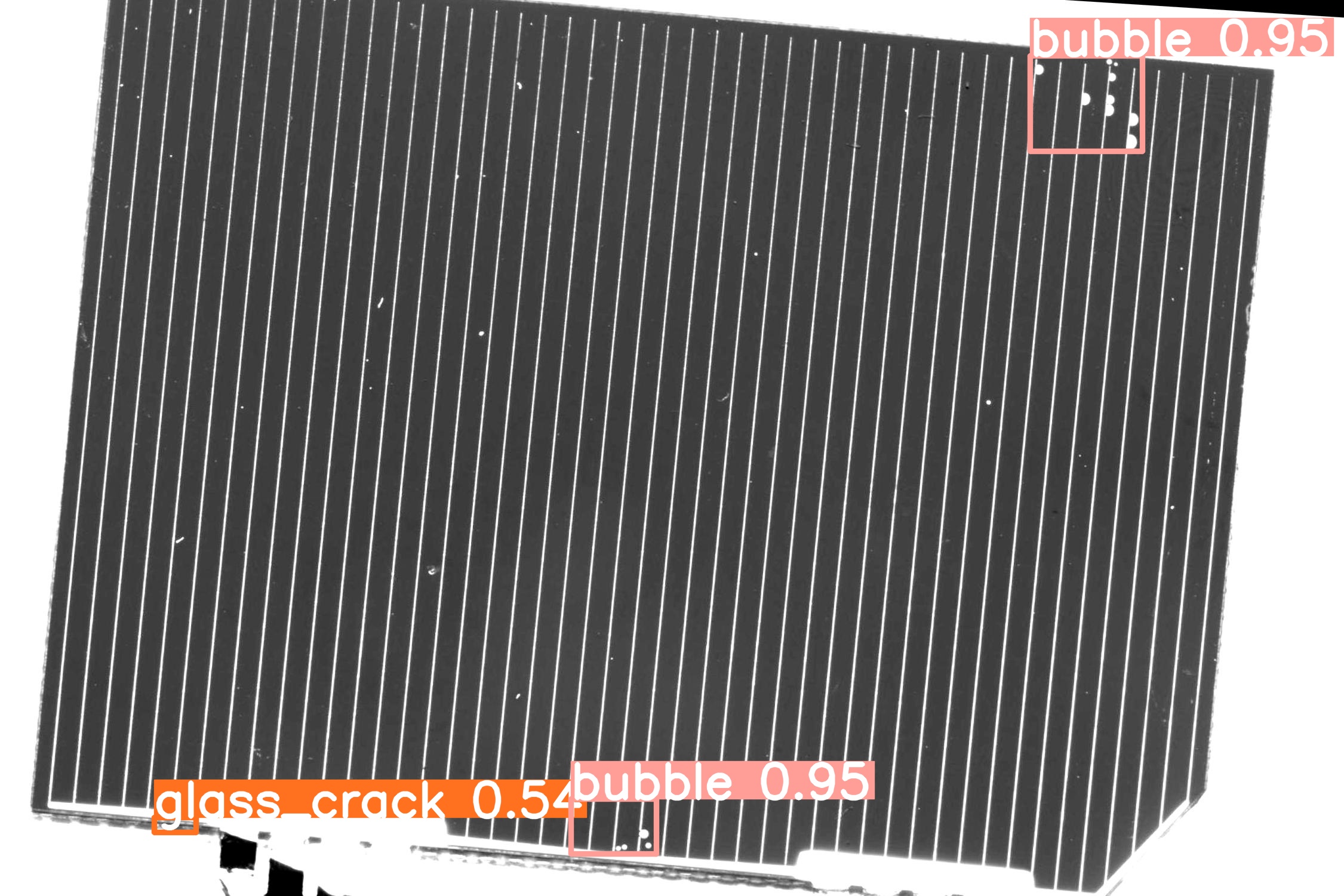

Supplement: S1 Dataset — (ZIP) [file pone.0304819.s001.zip › 6336.jpg]

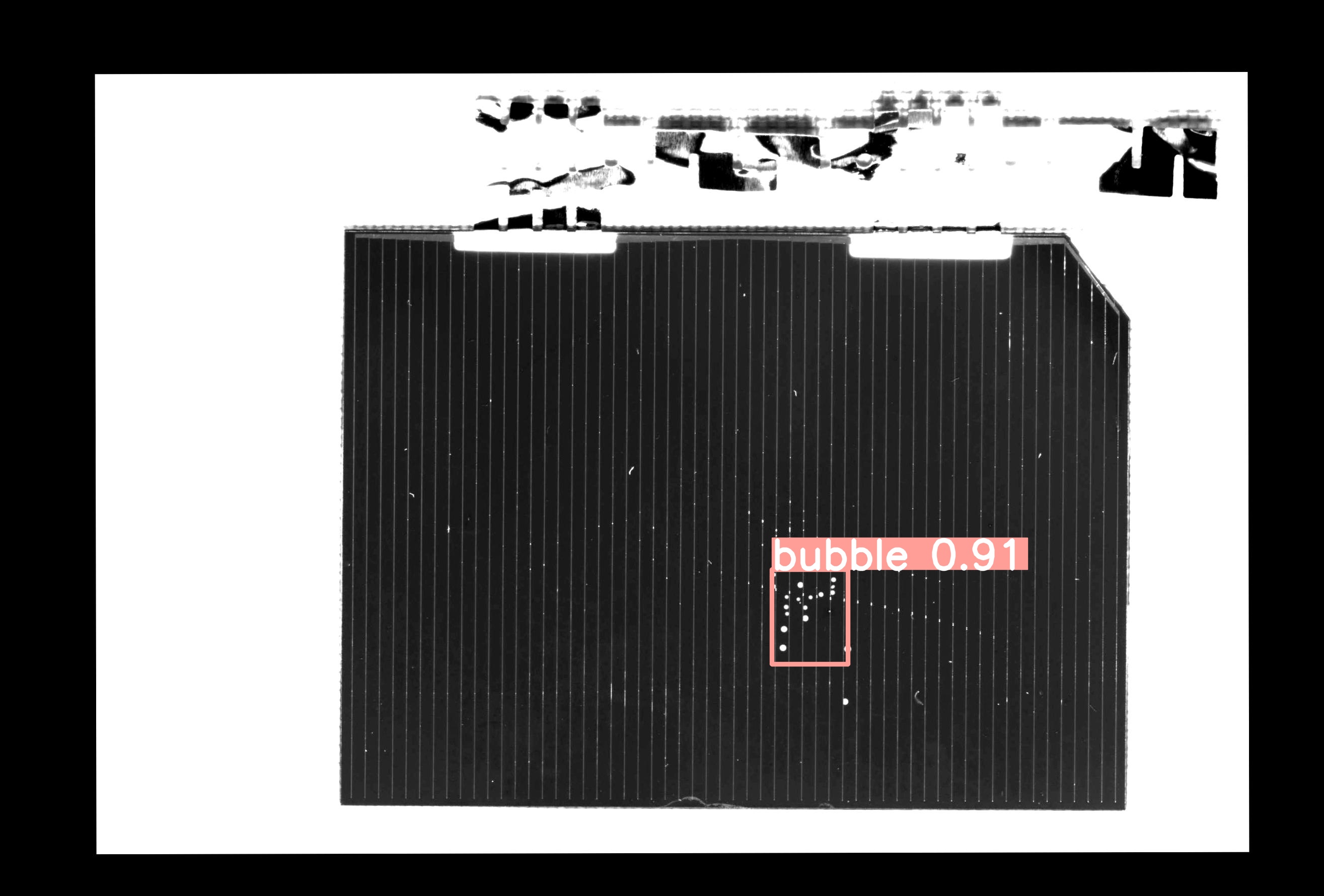

Supplement: S1 Dataset — (ZIP) [file pone.0304819.s001.zip › 6398.jpg]

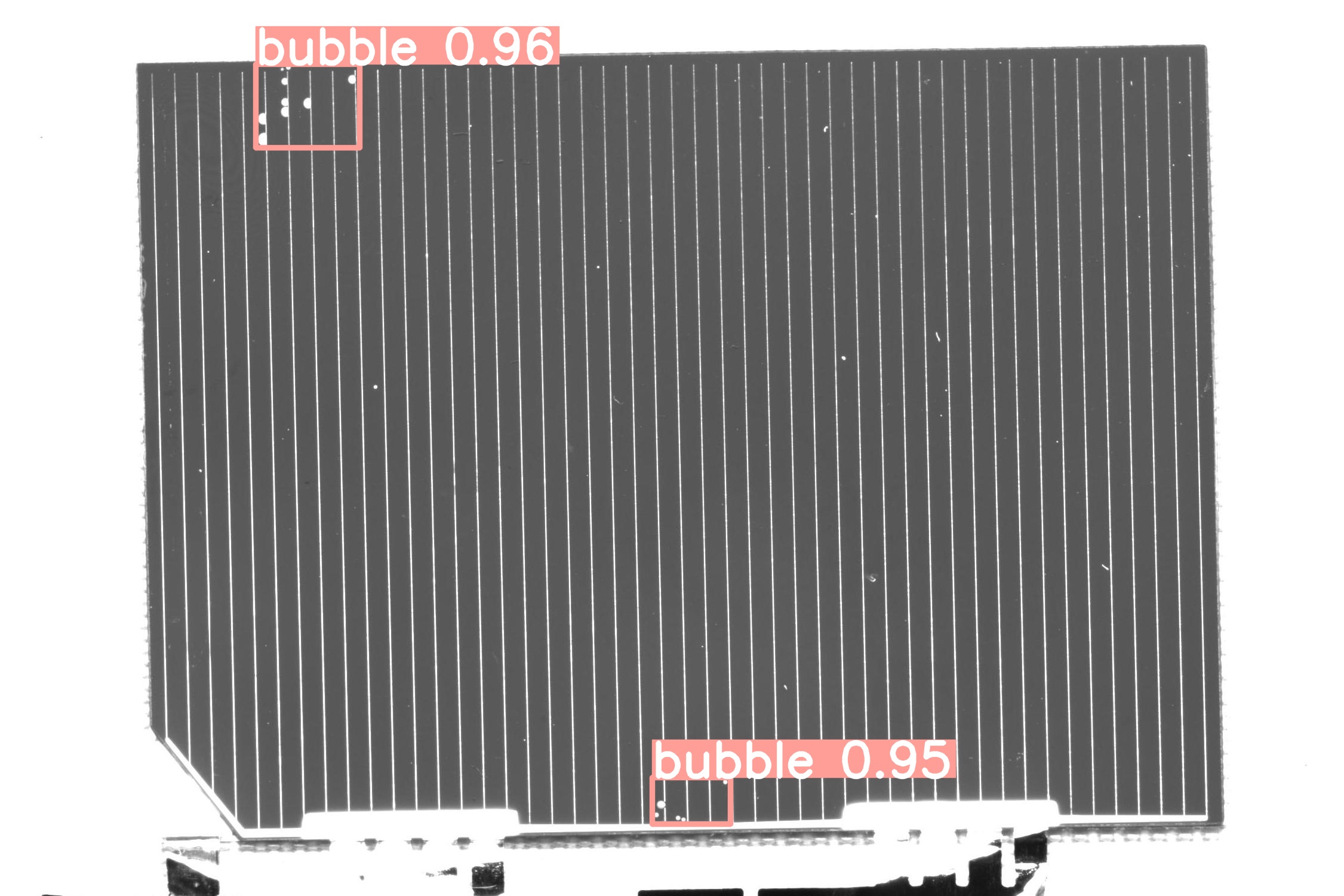

Supplement: S1 Dataset — (ZIP) [file pone.0304819.s001.zip › 6472.jpg]

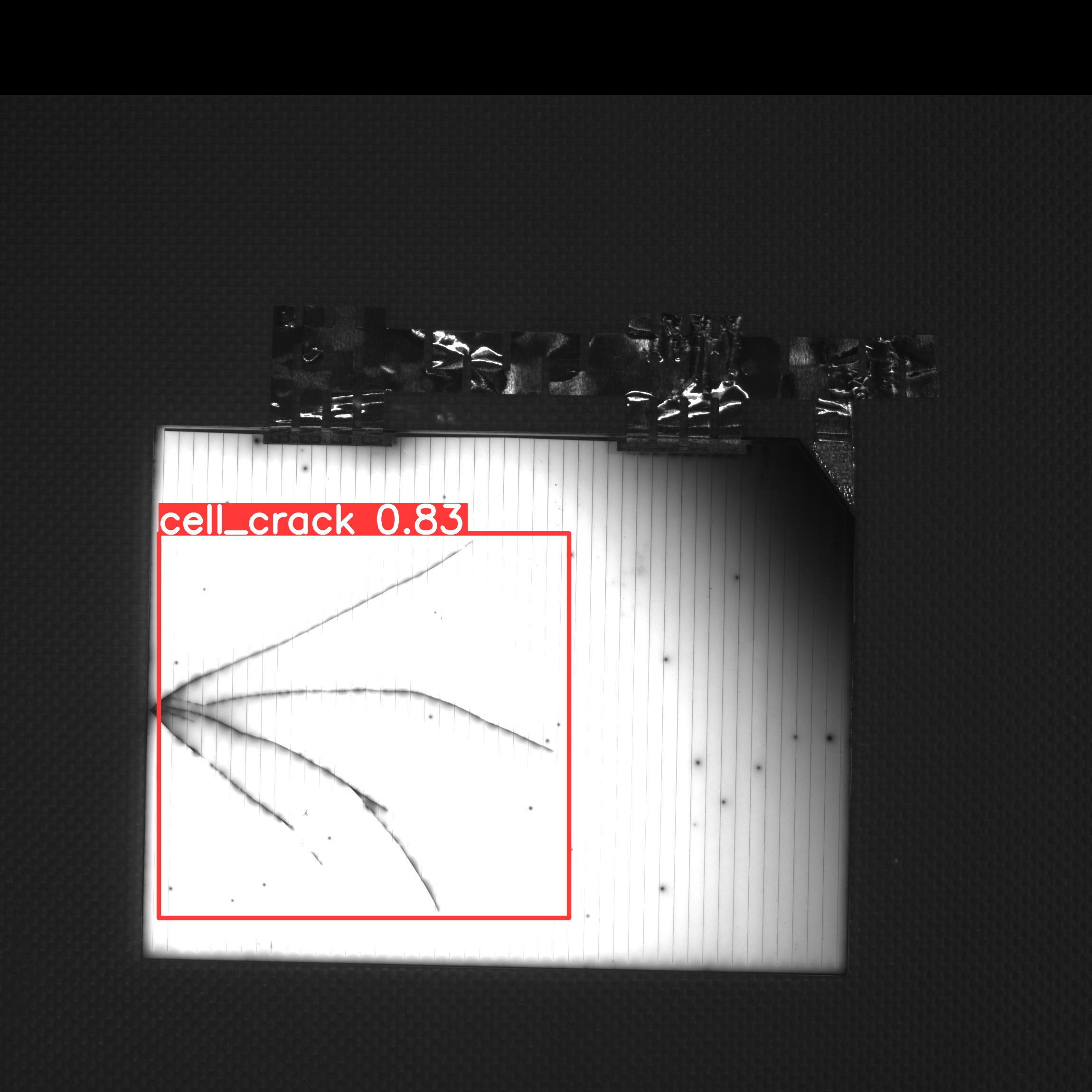

Supplement: S1 Dataset — (ZIP) [file pone.0304819.s001.zip › 6566.jpg]

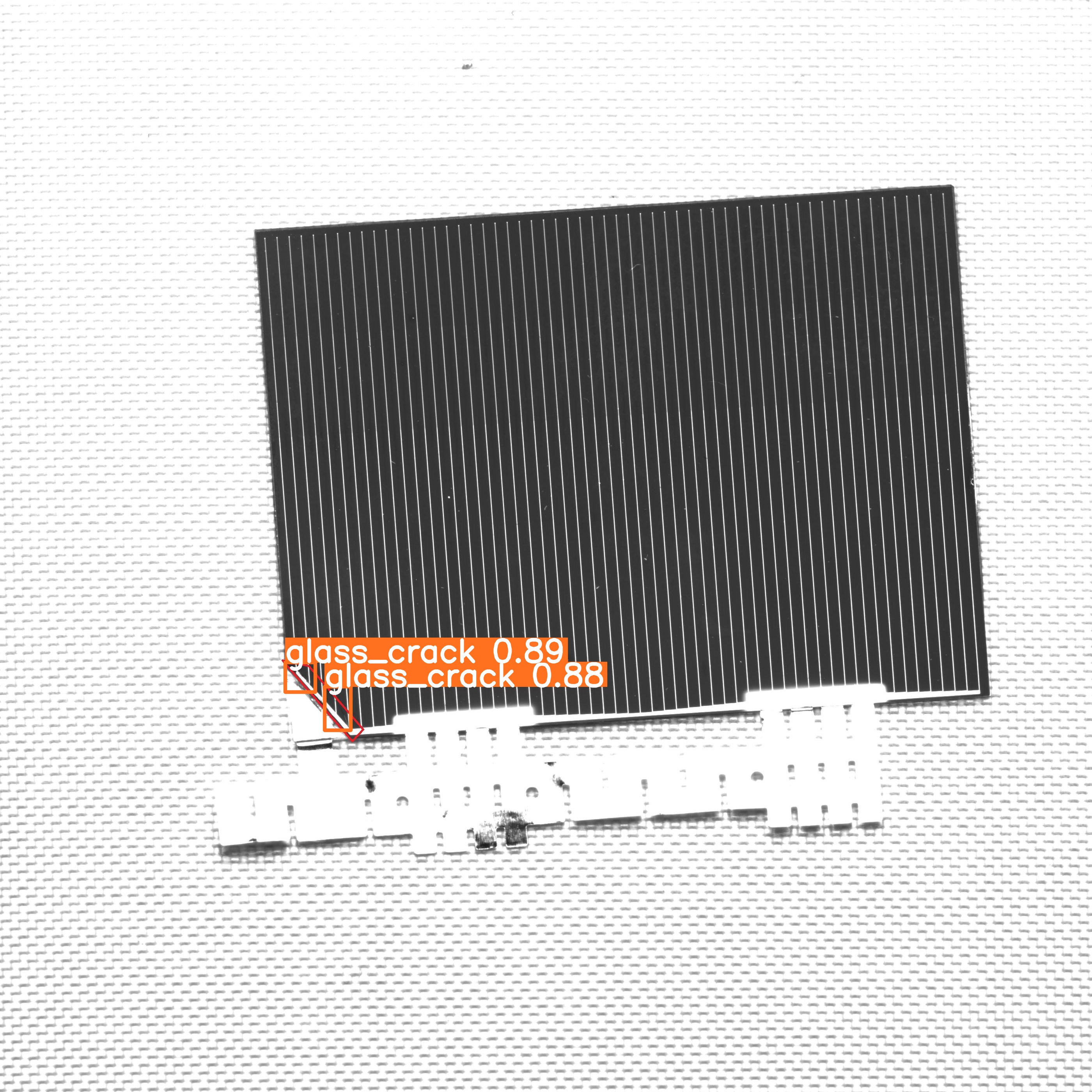

Supplement: S1 Dataset — (ZIP) [file pone.0304819.s001.zip › 6665.jpg]

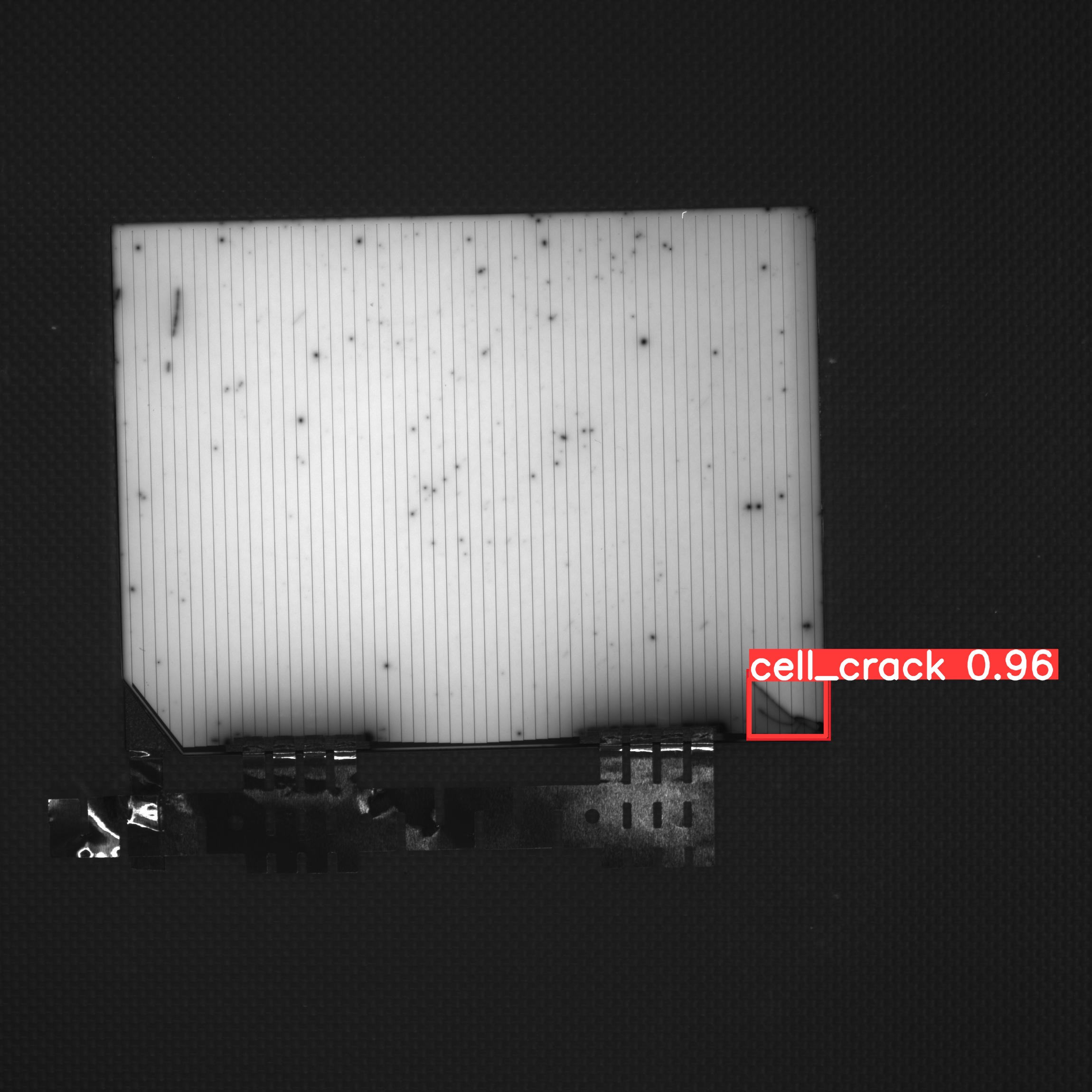

Supplement: S1 Dataset — (ZIP) [file pone.0304819.s001.zip › 6823.jpg]
